# Insights into angiosperm evolution, floral development and chemical biosynthesis from the *Aristolochia fimbriata* genome

**DOI:** 10.1038/s41477-021-00990-2

**Published:** 2021-09-02

**Authors:** Liuyu Qin, Yiheng Hu, Jinpeng Wang, Xiaoliang Wang, Ran Zhao, Hongyan Shan, Kunpeng Li, Peng Xu, Hanying Wu, Xueqing Yan, Lumei Liu, Xin Yi, Stefan Wanke, John E. Bowers, James H. Leebens-Mack, Claude W. dePamphilis, Pamela S. Soltis, Douglas E. Soltis, Hongzhi Kong, Yuannian Jiao

**Affiliations:** 1 State Key Laboratory of Systematic and Evolutionary Botany, Institute of Botany, the Chinese Academy of Sciences, Beijing, China; 2 University of Chinese Academy of Sciences, Beijing, China; 3 School of Life Sciences and Center for Genomics and Computational Biology, North China University of Science and Technology, Tangshan, China; 4 Institute of Botany, Dresden University of Technology, Dresden, Germany; 5 Department of Plant Biology, University of Georgia, Athens, GA USA; 6 Plant Genome Mapping Laboratory, University of Georgia, Athens, GA USA; 7 Department of Biology and Huck Institutes of the Life Sciences, The Pennsylvania State University, University Park, PA USA; 8 Florida Museum of Natural History, University of Florida, Gainesville, FL USA; 9 Department of Biology, University of Florida, Gainesville, FL USA

**Keywords:** Genome evolution, Plant evolution, Genome duplication, Secondary metabolism

## Abstract

*Aristolochia*, a genus in the magnoliid order Piperales, has been famous for centuries for its highly specialized flowers and wide medicinal applications. Here, we present a new, high-quality genome sequence of *Aristolochia fimbriata*, a species that, similar to *Amborella trichopoda*, lacks further whole-genome duplications since the origin of extant angiosperms. As such, the *A. fimbriata* genome is an excellent reference for inferences of angiosperm genome evolution, enabling detection of two novel whole-genome duplications in Piperales and dating of previously reported whole-genome duplications in other magnoliids. Genomic comparisons between *A. fimbriata* and other angiosperms facilitated the identification of ancient genomic rearrangements suggesting the placement of magnoliids as sister to monocots, whereas phylogenetic inferences based on sequence data we compiled yielded ambiguous relationships. By identifying associated homologues and investigating their evolutionary histories and expression patterns, we revealed highly conserved floral developmental genes and their distinct downstream regulatory network that may contribute to the complex flower morphology in *A. fimbriata*. Finally, we elucidated the genetic basis underlying the biosynthesis of terpenoids and aristolochic acids in *A. fimbriata*.

## Abstract (web summary)

A high-quality genome of *Aristolochia fimbriata* illuminates its unique history of whole-genome duplication similar to *Amborella*, the genomic basis of its complex flower morphology and chemical biosynthesis, and the phylogenetic placement of magnoliids.

## Main

Angiosperms, or flowering plants, are by far the largest group of land plants and comprise more than 350,000 living species (http://www.theplantlist.org/). Among extant angiosperms, Amborellales, Nymphaeales and Austrobaileyales (the so-called ANA grade) are followed by the rapid diversification of the remaining angiosperms or mesangiosperms^1,2^. The major mesangiosperm lineages are the eudicot, monocot and magnoliid clades, which make up approximately 75, 22 and 3% of angiosperm species diversity, respectively, and are the product of an ancient, rapid radiation^1,3^. Despite the availability of numerous sequenced nuclear genomes from eudicots and monocots, as well as the recently sequenced genomes of several magnoliids^4–12^, there remain many unanswered questions about early mesangiosperm diversification and molecular mechanisms that have contributed to within-lineage diversification and evolution. In spite of much attention, the phylogenetic relationships among eudicots, monocots and magnoliids remain uncertain and strongly debated^4–20^.

**Fig. 1:**
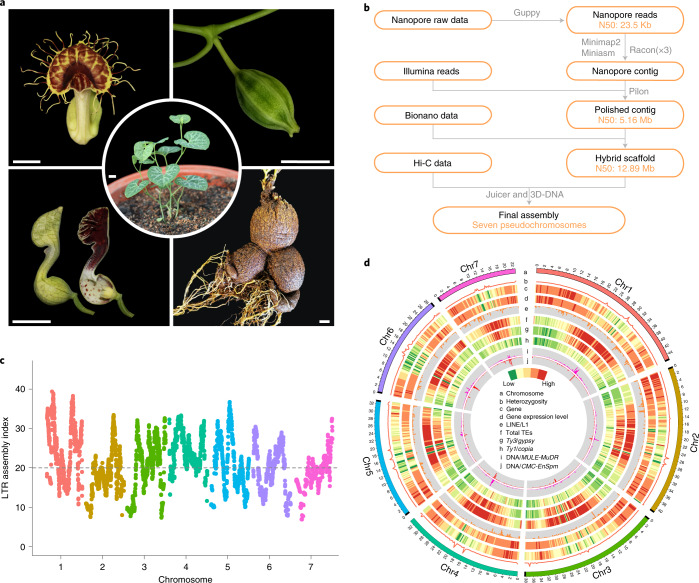
Overview of the *A. fimbriata* genome assembly and features. **a**, Morphology of the seedlings, flowers, fruit and root of *A. fimbriata*. Scale bars, 1 cm. **b**, Genome assembly pipeline used for the *A. fimbriata*. **c**, LAI assessment for each assembled *A. fimbriata* chromosome. The average LAI is about 21, indicating the high quality of our assembly. Dashed line (LAI = 20) indicates the gold standard quality level of the assembly. **d**, Distribution of *A. fimbriata* genomic features. Track ‘a’ represents the assembled seven chromosomes and the black boxes at the end of each chromosome represent the assembled telomere regions. Tracks ‘b–j’ represent the other genomic features as indicated in the centre of the Circos plot. The colours represent the density of genomic features in each 300-kb sliding window on the chromosomes. Source data

**Extended Data Fig. 1:**
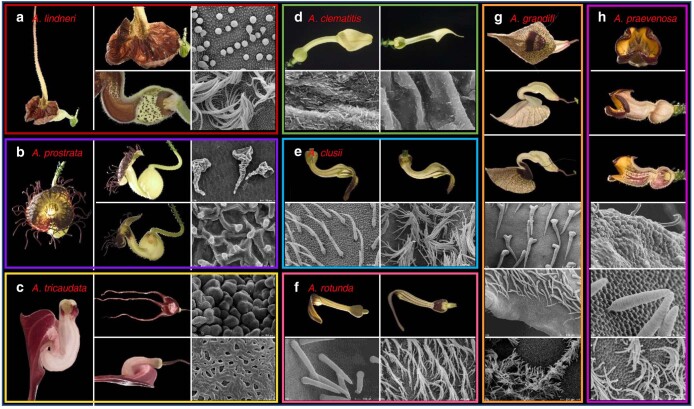
Flower morphologies of eight other *Aristolochia* species. For each selected species, the front and side views and longitudinal section of flowers at anthesis, as well as the scanning electron micrographs of the inner epidermis of perianth, are shown.

The magnoliid family Aristolochiaceae (Piperales; APG IV) comprises ~550 species, most of which are members of the large genus *Aristolochia* (450 species)^21,22^. *Aristolochia* species usually have a highly specialized flower morphology^23,24^. Whereas most ANA grade species and magnoliids have radial floral symmetry (and indeed, radial symmetry has been reconstructed as the ancestral state in angiosperms^25^), the flowers of *Aristolochia* comprise a petaloid, sepal-derived perianth that is monosymmetric (often tubular and dull purple-brown) and a gynostemium formed by the congenital fusion between stamens and the stigmatic region of the carpels^21^ (Fig. 1a and Extended Data Fig. 1). The peculiar floral structure of ‘pipevine’ or ‘Dutchman’s pipe’, together with the extensive floral modifications including scents, nectaries and trichomes, may have facilitated the evolution of deceptive pollination systems in *Aristolochia* that include attraction, imprisonment and release of specific pollinators^24,26^. In addition to their unique flower morphology, many *Aristolochia* species are important resources of traditional medicines^27^. Recent studies have demonstrated that a class of nitrophenanthrene carboxylic acids, known as aristolochic acids (AAs), naturally produced by *Aristolochia* species are highly nephrotoxic and carcinogenic to humans^28–30^. Yet, the exact biosynthesis pathway of AAs remains unknown. Collectively, these features warrant increased appreciation of *Aristolochia* species as valuable model systems for plant evolutionary developmental biology (evo-devo) and medicinal plant studies.

Here, we report the de novo genome assembly of a species in the genus *Aristolochia*, *A. fimbriata*, which has enormous potential as a useful genetic model system for magnoliids, as proposed previously^21^, because of its short life cycle, ease of large-scale cultivation and small genome size (~0.87 pg 2C value). Our most striking finding is that, unlike nearly all other ~200 angiosperm genomes sequenced to date, *A. fimbriata* has not undergone any whole-genome duplications (WGDs) beyond the ancestral WGD that predated diversification of all living angiosperm lineages^31^. The only other angiosperm for which this is known to be the case is *Amborella trichopoda* (Amborellaceae; hereafter simply *Amborella*), the sister to all other living angiosperms^32^. The absences of WGDs and subsequent subgenome rearrangement make *Aristolochia* an exceptionally powerful evolutionary genomic resource that we use to improve understanding of WGDs in magnoliids and early angiosperm diversification and to decipher molecular developmental genetics underlying both flower development and natural products (terpenoids and AAs) biosynthesis.

## Results

### High-quality genome assembly and annotation of *A. fimbriata*

**Extended Data Fig. 2:**
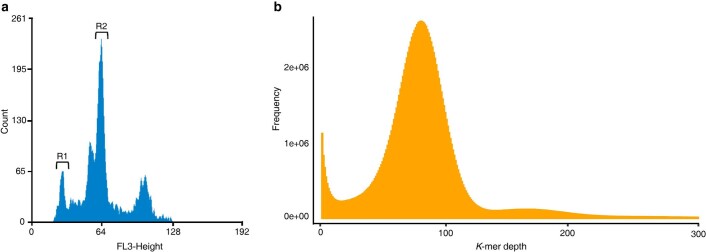
Genome size estimation of *A. fimbriata*. **a**, Genome size estimation for *A. fimbriata* (R2) based on flow cytometry using *A. thaliana* (R1, 125 Mb/2 C) as an internal reference. The genome size of *A. fimbriata* was estimated to be approximately 289.50 Mb. **b**, 17-mer-based analysis of estimation of genome size. The total number of *k*-mer is ~20,853,344,487, and the peak of the *k*-mer depth is ~83; therefore, the estimated genome size is approximately 251 Mb.

**Extended Data Fig. 3:**
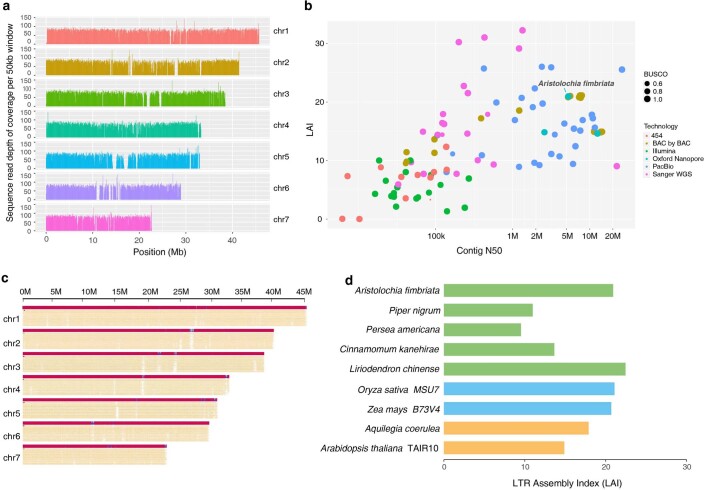
Genome assembly quality assessments. **a**, Mapping profile of the Nanopore clean reads to the final *A. fimbriata* assembly. **b**, Comparison of the length of contig N50 and the LTR Assembly Index (LAI) for the 105 published plant genome assemblies. **c**, Alignments of the Bionano molecules to the assembled chromosomes of *A. fimbriata*. **d**, Comparison of LAIs for several representative plant genomes. All stats indicate that the *A. fimbriata* assembly quality is outstanding. Source data

The genome of *A. fimbriata* was sequenced and assembled using Oxford Nanopore Technologies, Bionano optical mapping and Hi-C sequencing (Fig. 1b). The final nuclear genome assembly is about 258 megabases (Mb) and consists of 283 scaffolds with an N50 of 12.9 Mb (Supplementary Tables 1.6 and 1.7). The assembled genome size is similar to the estimated genome size based on flow cytometry and *k*-mer analyses (Extended Data Fig. 2). Using the Hi-C contact information, these scaffolds were further anchored onto seven pseudochromosomes, which cover ~95% of the assembled sequences (Supplementary Note 1.3 and Supplementary Fig. 1.3). Probably due to propagation via selfing over ~20 yr in cultivation, the sequenced *A. fimbriata* accession has extremely low heterozygosity (~0.07%) simplifying genome assembly (Fig. 1). The overall read-mapping rates for transcriptomes (for example, those from leaves, flowers, roots and seedlings with and without stress treatments) and for genomic sequences exceeded 93 and 99%, respectively (Supplementary Tables 1.9 and 1.10). Moreover, 96.8% of the Plantae BUSCO (Benchmarking Universal Single-Copy Orthologs)^33^ genes were identified in the genome (Supplementary Table 1.11). The long terminal repeat (LTR) Assembly Index (LAI)^34^ of the genome assembly is ~21 (Fig. 1c and Extended Data Fig. 3b,d). These results, as well as those from other genome quality assessments (Supplementary Note 1.4 and Extended Data Fig. 3), suggest that the *A. fimbriata* genome assembly is of high quality.

We annotated 21,751 protein-coding gene models from the *A. fimbriata* genome, 19,582 of which were classified as high-confidence genes on the basis of whether they have support from the aforementioned transcriptomes and whether they exhibit overlapping with TEs (Supplementary Note 2). Gene family classification and comparison showed that most of the commonly shared orthogroups comprise annotated *A. fimbriata* genes and that *A. fimbriata* has fewer species-specific orthogroups than many other flowering plants (Supplementary Fig. 2.3). Transposable elements (TEs) occupy ~52.1% of the *A. fimbriata* genome and the LTR retrotransposons represent 38.2% of the assembly (Supplementary Table 2.2). *Ty3*/*Gypsy* elements account for 21.3%, while the *Ty1*/*Copia* elements cover 4.6% of the genome (Supplementary Table 2.2). DNA transposons *MULE-MuDR* and *CMC-EnSpm* are enriched in centromeric regions but are absent from the rest of the genome (Fig. 1d). Notably, and clearly distinct from reports for the other published magnoliid genomes^5,6^, LINE/L1 elements have expanded substantially in *A. fimbriata*; these elements tend to be located outside of the centromeric regions and are especially evident in genic regions (Fig. 1d and Supplementary Fig. 2.1b,c). We also observed an elevation in the expression levels of genes with the insertion of LINE/L1 elements in the intron regions as compared to the much larger set of genes lacking such insertions (Supplementary Fig. 2.1d).

### A genome sequence free of lineage-specific WGD

**Fig. 2:**
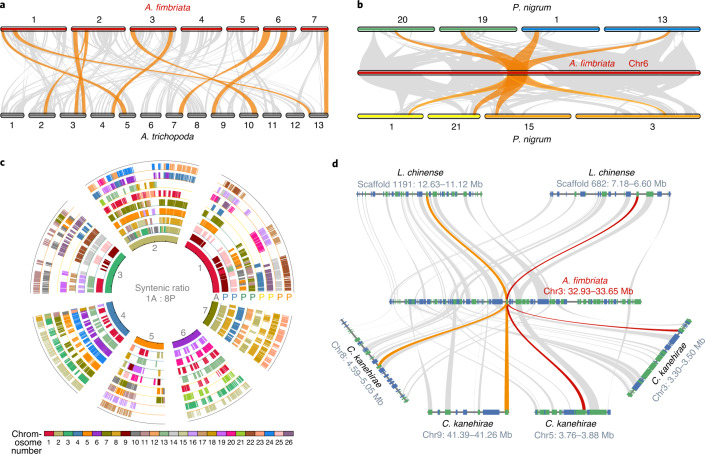
Intergenomic comparisons revealed that *A. fimbriata* lacks any WGD after the shared WGD in the common ancestor of all angiosperms and identified two novel WGDs in *P. nigrum*. **a**, Syntenic comparison between *A. fimbriata* and *A. trichopoda*^37^ revealed a 1:1 ratio that suggests no lineage-specific WGD in *A. fimbriata* after its divergence from *A. trichopoda*. Syntenic blocks with more than ten genes are linked by grey lines; the largest ten syntenic blocks are highlighted in orange. **b**, Three rounds of WGDs in *P. nigrum* were identified via syntenic comparison to *A. fimbriata*, a finding in contrast to the single WGD in a previous report^4^. Exemplar syntenic relationships of eight regions in *P. nigrum* matching a single genomic region in *A. fimbriata* are highlighted in orange. **c**, Chromosome-level syntenic alignments of *P. nigrum* to the *A. fimbriata* reference genome further support three WGDs. The inner circle represents the seven chromosomes of *A. fimbriata* (marked with A) and the outer eight circles illustrate the corresponding syntenic regions in *P. nigrum* (marked with P). The corresponding circles with the same colour of ‘P’ represent synteny from the most recent WGD (Pn-α); the circles where ‘P’ is coloured blue, green, yellow or orange were duplicated in the Pn-β event. **d**, Microsynteny comparisons clarified the timing of other previously reported WGDs in magnoliids. Representative synteny relationship shows that one *A. fimbriata* region matches two regions in *L. chinense* and four regions in *C. kanehirae*. Rectangles represent annotated genes with orientation on the same strand (blue) or reverse strand (green) and the grey lines connect syntenic gene pairs, with one set highlighted in colour.

WGDs have occurred frequently throughout the evolutionary history of angiosperms^15,31,35^ and a genome sequence lacking lineage-specific WGD could facilitate the studies of genome evolution and inference of the WGD history in other species^36^. Until now, only *Amborella* is known to lack any lineage-specific WGD; it only possesses evidence for a WGD that occurred in an ancestor of all extant flowering plants^32^. It is therefore noteworthy that an intragenomic comparison of the genome of *A. fimbriata* revealed very sparse self-synteny blocks, indicating absence of any recent WGDs in *A. fimbriata* (Supplementary Fig. 3.1). We further conducted intergenomic comparisons against *Amborella*^32,37^ and also against a water lily (*Nymphaea colorata*) that has one lineage-specific WGD^38^. The corresponding syntenic depth ratios are 1:1 and 1:2 (Fig. 2a and Supplementary Figs. 3.2 and 3.3), respectively, which strongly support the lack of further WGD in *A. fimbriata* since the earliest diversification of extant angiosperm lineages (Supplementary Note 3.1). Notably, *A. fimbriata* is thus only the second flowering plant species with a sequenced genome that has a genomic evolutionary history that is similar to that of *Amborella* in having no additional lineage-specific WGD.

Comparing the genomes of *Amborella* and *A. fimbriata*, we identified 450 intergenomic syntenic blocks comprising 6,378 anchor genes in each genome, of which ten syntenic blocks have >50 anchor gene pairs (Supplementary Table 3.1). The longest syntenic block, which is between *A. fimbriata* chromosome 3 and *Amborella* chromosome 4, has 77 anchor gene pairs, suggestive of high conservation (Fig. 2a, Supplementary Fig. 3.2a and Supplementary Table 3.1). In contrast, we only detected three syntenic regions with >50 anchor gene pairs between *A. fimbriata* and *N. colorata* (Supplementary Table 3.1), which suggests extensive chromosomal rearrangements in *Nymphaea*, perhaps following WGD^36,38^. These results suggest that the *A. fimbriata* genome could serve as another exceptional reference for evolutionary genomic studies of angiosperms.

Using the *A. fimbriata* genome as a reference, we were able to identify new WGDs in Piperales and clarify the timing of the previously proposed WGDs in Laurales and Magnoliales. By comparing the genome of *A. fimbriata* with that of black pepper (*Piper nigrum*; Piperaceae), we found one-to-eight well-preserved intergenomic syntenic blocks, suggesting three successive rounds of lineage-specific WGDs in black pepper (Fig. 2b,c and Supplementary Fig. 3.4). Further synonymous substitutions per site (Ks) analyses of the anchor gene pairs in the self-synteny blocks of black pepper also provide estimates of these same three duplication events (Ks peaks around 0.11, 0.69 and 0.91; we named them Pn-α, Pn-β and Pn-γ, respectively), all of which occurred after the divergence of black pepper and *A. fimbriata* (Supplementary Figs. 3.5 and 3.6). However, only the most recent lineage-specific WGD (Pn-α) was reported in the previous analysis of the black pepper genome^4^.

In addition, we identified a 1:2 syntenic depth ratio between *A. fimbriata* and *Liriodendron chinense* (Magnoliaceae) and a 1:4 ratio between *A. fimbriata* and *Cinnamomum kanehirae* (Lauraceae) (Fig. 2d and Supplementary Figs. 3.7 and 3.8), thereby confirming the previously reported single WGD in *L. chinense*^5^ and two rounds of WGD in *C. kanehirae*^6^ since the divergence of magnoliids. Ks-based analyses could possibly verify these WGDs; however, owing to the variable evolutionary rates of different species, it is hard to confidently conclude whether any of the WGDs were shared among magnoliid species^39^. Using integrated phylogenomic and synteny analyses^40,41^, we found that, of the two WGDs identified in *C. kanehirae*, the more ancient one was shared with *L. chinense* whereas the recent one was shared with *Persea americana* (Lauraceae) (Supplementary Note 3.3).

### Structural variation and angiosperm phylogeny

Recently, several other genome sequencing and phylogenomic studies have proposed discordant phylogenetic relationships among the mesangiosperm clades of eudicots, monocots and magnoliids^4–12^, which is probably due in part to the different and sparse taxon sampling used, rapid diversification and true variation in the phylogenetic histories of nuclear genes and the plastid genome^14^. A recent phylogenetic study based on genome-wide synteny network data suggested the magnoliids as a sister lineage to monocots^42^. Other phylogenetic studies, which combined nuclear genome sequences and transcriptomes from large-scale species sampling, recovered a sister relationship between magnoliids and eudicots^15–18^. Analyses using chloroplast genomes, however, seem to strongly support magnoliids as a sister to the clade of monocots and eudicots^19^. Here, we attempted to investigate these phylogenetic discrepancies through comparisons of genomic structural features.

Specifically, after comparing the *A. fimbriata* genome to those of the other angiosperms, we identified several large chromosomal rearrangements that probably occurred during the early evolution of angiosperms (Supplementary Note 3.4). Through intergenomic comparisons between the *A. fimbriata* genome and those of *Amborella* and *N. colorata* from the ANA grade, we found that regions of *A. fimbriata* chromosome 6 (Af6) are orthologous with segments of *Amborella* chromosomes 7 or 9 and *N. colorata* chromosomes 4 and 12 or chromosomes 2 and 9 (Supplementary Fig. 3.10). Similarly, we also found that chromosome 7 of *A. fimbriata* (Af7) has non-overlapped orthologous syntenic regions in *Amborella*, as well as in *N. colorata* (Supplementary Fig. 3.10). These structural comparisons indicate that chromosomes 6 and 7 of *A. fimbriata* might have formed via fusion events in an ancestor of *A. fimbriata*.

**Extended Data Fig. 4:**
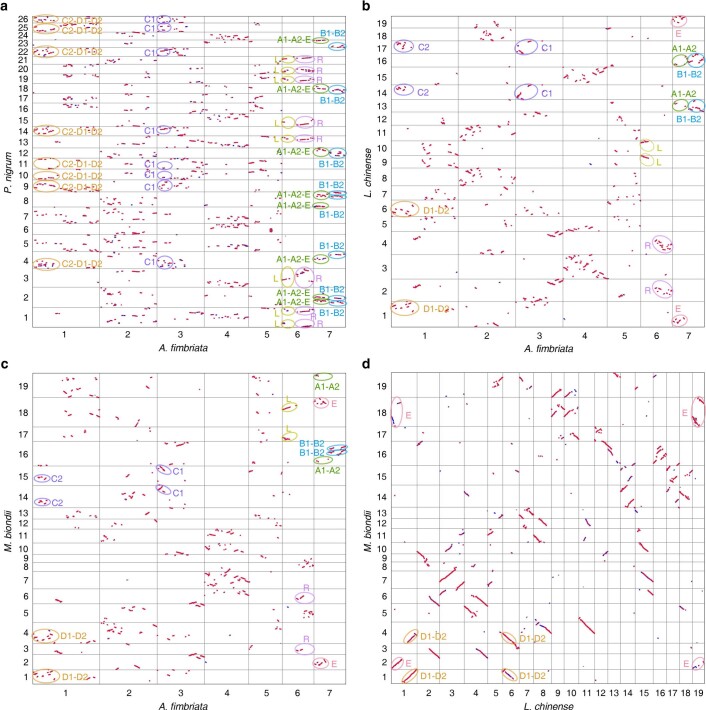
Genomic comparison of the *A. fimbriata* and *L. chinense*, with the *P. nigrum* and *M. biondii* genomes, respectively. **a**, Syntenic dotplot between the *A. fimbriata* and *P. nigrum* genomes. **b**, Syntenic dotplot between the *A. fimbriata* and *L. chinense* genomes. **c**, Syntenic dotplot between the *A. fimbriata* and *M. biondii* genomes. **d**, Syntenic dotplot between the *L. chinense* and *M. biondii* genomes. Given the orthologous D and E regions in *L. chinense* remain in ancestral status (not merged with C2 or A), we could use the *L. chinense* as another comparing reference to clearly infer the A1-A2/E and the D1-D2/C2 orthologous regions in other genomes. The names of these circled syntenic blocks in **d** were inferred based on the D and E genomic regions in *L. chinense* that exhibiting orthologous relationships to these defined D and E regions in *A. fimbriata* respectively.

**Extended Data Fig. 5:**
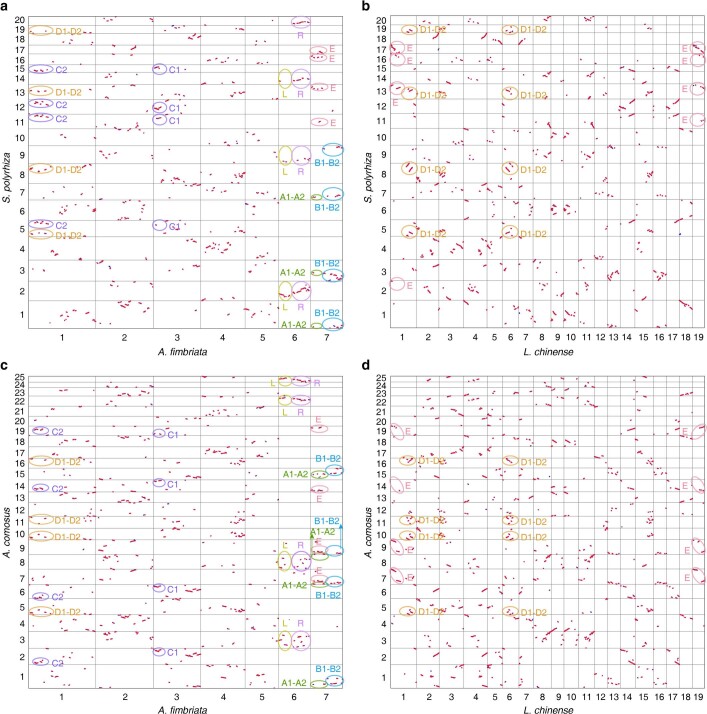
Genomic comparisons of the *A. fimbriata* and *L. chinense* with the *S. polyrhiza* and *A. comosus* genomes, respectively. **a**, Syntenic dotplot between the *A. fimbriata* and *S. polyrhiza* genomes. **b**, Syntenic dotplot between the *L. chinense* and *S. polyrhiza* genomes. **c**, Syntenic dotplot between the *A. fimbriata* and *A. comosus* genomes. **d**, Syntenic dotplot between the *L. chinense* and *A. comosus* genomes. The orthologous region of the D1-D2 and E in *S. polyrhiza* and *A. comosus* could be further verified by the syntenic relationship to the corresponding D1-D2 and E regions in *L. chinense*.

We further compared the *A. fimbriata* genome to those of representative magnoliid, eudicot and monocot species to determine whether or not the associated genomic rearrangements are shared by two or all three mesangiosperm clades. Chromosome Af6 has integrated orthologous regions in the other published Piperales genome of *P. nigrum* and the monocot genomes of *Ananas comosus* (Bromeliaceae)*, Asparagus setaceus* (Asparagaceae)*, Spirodela polyrhiza* (Lemnaceae) and *Elaeis guineensis* (Arecaceae) (Extended Data Figs. 4a and 5a,c and Supplementary Fig. 3.12a,c). In contrast, when compared to the eudicot genomes of *Vitis vinifera* (Vitaceae), *Acer yangbiense* (Aceraceae), *Tetracentron sinense* (Trochodendraceae) and *Aquilegia coerulea* (Ranunculaceae) and the other magnoliid genomes of *L. chinense*, *Magnolia biondii*, *C. kanehirae* and *Litsea cubeba*, we found that Af6 has syntenic orthologous regions on two or more homoeologous chromosome sets in these species (Extended Data Fig. 4b,c and Supplementary Figs. 3.11a,c and 3.13–3.15). Moreover, the locations of these breakpoints inferred between *A. fimbriata* and these eudicots and magnoliid species in Laurales and Magnoliales are similar to those between *A. fimbriata*, *Amborella* and *N. colorata* (Supplementary Figs. 3.15 and 3.16). Given that *Amborella* and *Nymphaea* exhibit similar genome organization patterns that differ from those of the *A. fimbriata* genome, we propose that the separated genomic regions were ancestral and either a fusion event occurred before the divergence of monocots and magnoliids followed by a further fission event in the common ancestor of Laurales and Magnoliales (scenario I) or parallel evolution in the Piperales and monocots led to similar fusions (scenario II). Scenario I would support the magnoliids and monocots as sister clades and eudicots as their sister lineage, while the scenario II could not provide evidence for the phylogenetic placement of magnoliids.

**Fig. 3:**
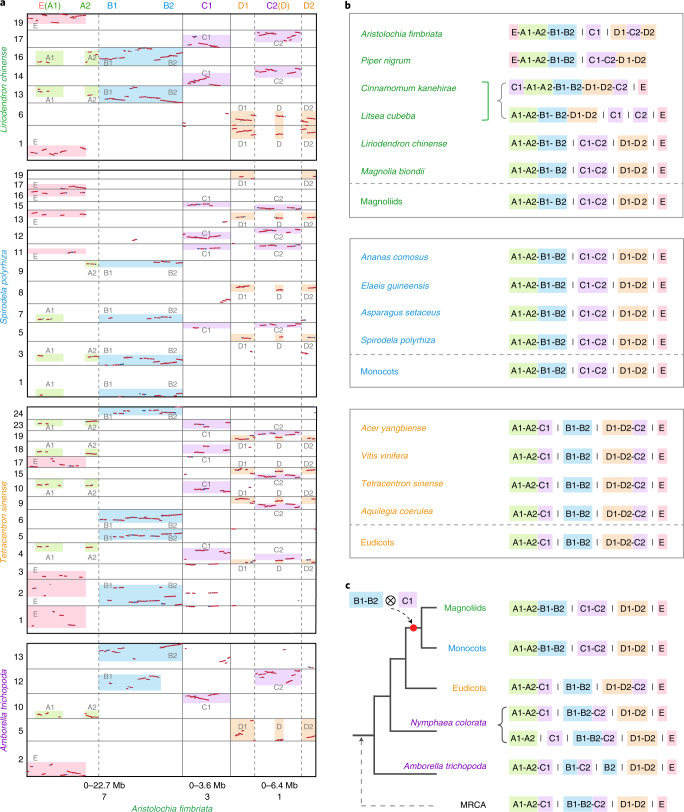
Common genomic rearrangements present in magnoliids and monocots but absent from eudicots and the two representative species of the ANA grade. **a**, The local syntenic blocks identified between the *A. fimbriata* genome and the genomes of *A. trichopoda, T. sinense, S. polyrhiza* and *L. chinense*. The specific genomic regions associated with the Af7 fusion were named regions of E, A1, A2, B1, B2, C1, C2, D, D1 and D2 as marked on top of the plot. The A1 region seems to be embedded in the E region, and is indicated as E(A1). Similarly, the D region seems to be embedded in the C2 region and is indicated as C2(D). Highlighted regions represent syntenic blocks among the compared genomes. The grey dotted lines in **a** indicate the fusion point in chromosome 7 of *A. fimbriata*. **b**, The connection patterns of the orthologous regions in the representative genomes of magnoliids, monocots and eudicots. There are two different connection patterns for the paralogous regions in the *C. kanehirae* and *L. cubeba* genomes and both patterns were presented. **c**, The inferred topology of angiosperms based on a common genomic exchange event shared by monocots and magnoliids. MRCA here represents the most recent common ancestor of extant angiosperms.

**Extended Data Fig. 6:**
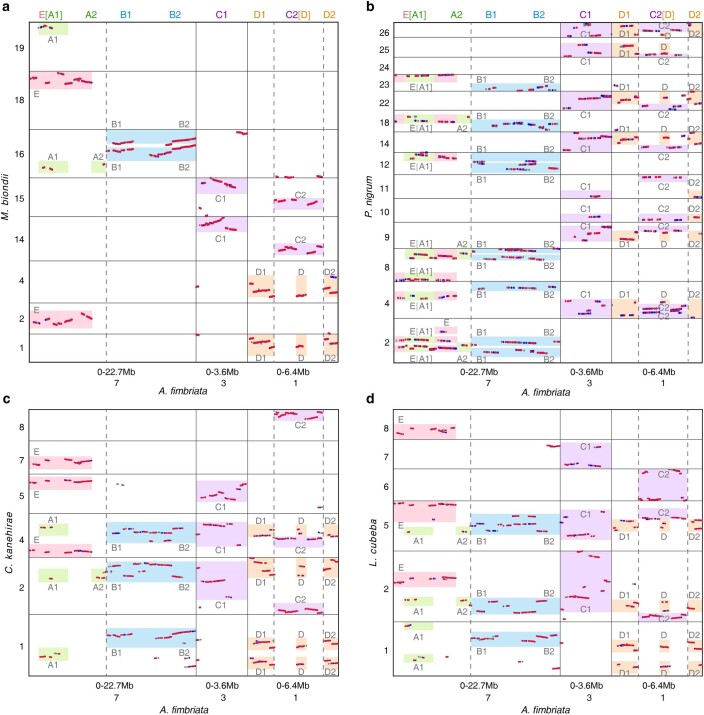
Local syntenic relationships among the selected genomic regions that associated with the structural rearrangements of *A. fimbriata* chromosome 7. **a**, The local syntenic blocks identified between the *A. fimbriata* and *M. biondii* genomes, **b**, The local syntenic blocks identified between the *A. fimbriata* and *P. nigrum* genomes, **c**, The local syntenic blocks identified between the *A. fimbriata* and *C. kanehirae* genomes, **d**, The syntenic blocks identified between the *A. fimbriata* and *L. cubeba* genomes. Similar to the Fig. 3a, the specific genomic regions associated with the *A. fimbriata* chromosome 7 fusion were named regions of E, A1, A2, B1, B2, C1, C2, D, D1 and D2 as marked on top of the plot.

Comparative analysis of Af7 provides even clearer evidence for an ancestral chromosomal fusion before the divergence of the magnoliids and monocots that is not shared with eudicots (Supplementary Note 3.4). Comprehensive genomic comparisons revealed that this event involved several other genomic regions of chromosomes 1, 3 and 7, thus we separated Af7 into the regions of E(A1)-A2-B1-B2 and also defined the region of Chr3: 0–3.6 Mb as C1 and region of Chr1: 0–6.4 Mb as D1-C2-D2 (Fig. 3a). We found that the fusion pattern of the A1-A2 and B1-B2 is common in magnoliids and monocots, while the A1-A2 is connected with C1 in the genomes of *Amborella*, *N. colorata* and eudicots (Fig. 3, Extended Data Figs. 4–6 and Supplementary Figs. 3.10–3.14 and 3.17–3.21). We also detected several lineages-specific structural changes, such as the Piperales-specific translocation of E region to the A1-A2, *A. fimbriata*-specific insertion of C2 into D1 and D2 and the separation of B1-B2 found in *Amborella* (Supplementary Note 3.4). After comprehensive examination of the connection pattern of these defined regions in the selected species, we reconstructed the most parsimonious ancestral patterns for the three major angiosperm clades, which are (A1-A2-B1-B2, C1-C2, D1-D2 and E) for magnoliids, (A1-A2-B1-B2, C1-C2, D1-D2 and E) for monocots and (A1-A2-C1, B1-B2, D1-D2-C2 and E) for eudicots (Fig. 3b). Together with the synteny patterns between *A. fimbriata* and the *Amborella* and *N. colorata* genomes, we predicted the structure of the homologous chromosome in the last common ancestor of extant angiosperms was (A1-A2-C1, B1-B2-C2, D1-D2 and E) (Fig. 3c). The reconstructions of ancestral chromosome structure imply a genomic exchange between regions of B1-B2 and C1 that occurred just before the divergence of monocots and magnoliids (Fig. 3c). This shared, derived (synapomorphic) chromosomal arrangement in magnoliids and monocots, but missing in eudicots, provides support for a magnoliid + monocot clade with eudicots as their sister lineage (Fig. 3c).

**Fig. 4:**
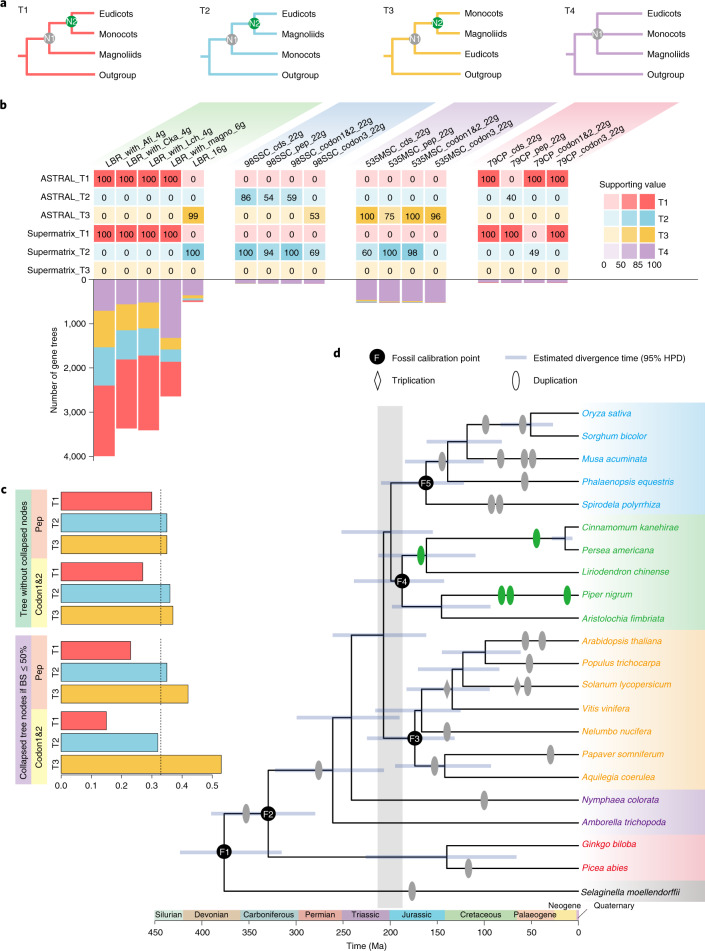
Challenges in using a phylogenomic approach to resolve relationships among the major angiosperm groups. **a**, Possible topologies among magnoliids, monocots and eudicots. **b**, The discordant topologies inferred from various taxon sampling using ASTRAL- and supermatrix-based approaches and individual gene trees. Numbers in the coloured boxes are the supporting values of the LPP or BS for the ‘N2’ nodes of the different topologies as shown in **a**. The bottom histogram shows the numbers of individual low-copy gene trees supporting the respective topologies. Species and clades are abbreviated as: *A. fimbriata*, Afi; *C. kanehirae*, Cka; *L. chinense* and Lch; magnoliids, Magno. **c**, Effect of gene tree resolution on the quartet frequencies of the 535 MSC gene families. Note that use of ML gene trees resulted in similar support levels for T2 and T3, whereas collapsing of nodes with BS values <50% in the ML trees resulted in strongest support for T3. Dashed lines show mean quartet frequencies at 0.33. **d**, The inferred phylogeny of representative angiosperms, shown with estimated divergence times. Blue bars at the nodes represent 95% confidence intervals of the estimated divergence time. WGD events are also shown on the species tree. The rapid divergence of eudicots, monocots and magnoliids at ~200 Ma is highlighted in grey.

**Extended Data Fig. 7:**
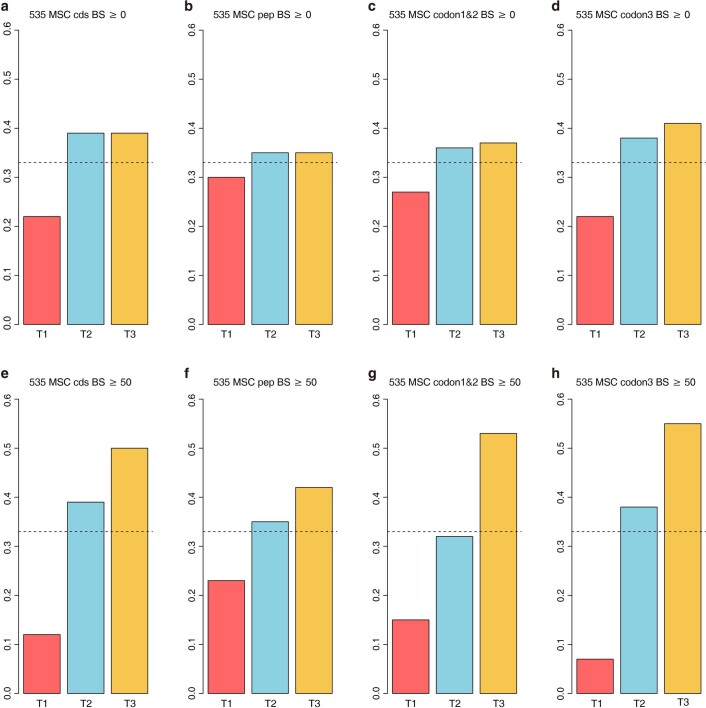
Gene tree quartet frequencies of 535 MSC gene families for different topologies. Here we inputted individual genes trees (**a-d**), and also ran with collapsed trees if BS was less than 50% (**e-h**). The x-axis labels T1, T2, and T3 refer to the quartet support for the topologies of T1 (red), T2 (blue), and T3 (yellow) in Fig. 4a respectively. The dashed line refers to a proportion of 0.33.

We also performed phylogenomic analyses using different taxon sampling datasets to investigate the reasons for the discordant topologies of monocots, eudicots and magnoliids (Supplementary Note 4). We identified 98 strictly single-copy (SSC) and 535 mostly single-copy (MSC) gene families from 22 representative species and maximum likelihood trees were constructed (Fig. 4 and Supplementary Table 2.8). Notably, we found that most of the individual nuclear gene trees show weak or no resolution regarding the phylogenetic relationships of the magnoliids, monocots and eudicots (Fig. 4a,b and Supplementary Table 4.2). In fact, a polytomy null hypothesis could not be rejected (the node of magnoliids, eudicots and monocots is a polytomy) (Supplementary Table 4.3). Gene tree quartet frequencies of the 98 SSC datasets slightly supported T2 (magnoliids and eudicots are sister clades; Fig. 4a), whereas the three topologies were almost equally supported from the 535 MSC datasets (Supplementary Note 4.1 and Extended Data Fig. 7), lending support for the polytomy hypothesis or rapid diversification with a high degree of incomplete lineage sorting (ILS) between successive bifurcations. Interestingly, strongly skewed quartet frequencies were recovered for one alternative tree (T2) relative to the other (T1) in ASTRAL analyses of the 535 MSC gene trees suggesting that processes other than ILS (for example, gene flow or gene duplication and loss of paralogous copies) may be contributing to gene tree discordance.

Analyses of concatenated nuclear gene alignments does not account for variation in gene histories due to ILS of ancestral sequence diversity but they can yield trees with identical branching orders if ILS is weak. The concatenation-based inferences using the various datasets of amino acid sequences and protein-coding sequences, as well as the partitioned codons, from the 98 SSC and 535 MSC gene families consistently supported magnoliids and eudicots as sister lineages (T2; Supplementary Note 4.1, Fig. 4a,b and Supplementary Fig. 4.1). Coalescent-based phylogenetic analyses of the 535 MSC nucleotide dataset also weakly supported T2 using ASTRAL and MP-EST (Supplementary Figs. 4.2a and 4.4a,c). However, if we used 535 MSC individual trees with collapsed nodes setting gradient bootstrap support (BS) values for coalescent analyses, the resulting topologies changed from T2 to T3, magnoliids as sister to monocots (Supplementary Fig. 4.2). Moreover, if we input the trees with nodes collapsed when their BS values were <50% to ASTRAL, the quartet frequency of T3 is much higher than the other two topologies (Fig. 4c). In addition, we used multicopy gene tree summary methods ASTRAL-Pro and STAG with 22,563 gene families and the results both support magnoliids and eudicots as sister groups (T2) (Supplementary Fig. 4.8). Therefore, our results showed that most of the individual gene trees exhibited low resolution regarding the topology of monocots, magnoliids and eudicots, while the resolution of individual gene trees has a great effect on the inferred topology for coalescent-based analyses.

Combining the genome structural evidence and the phylogenomic results, we propose the T3 topology (magnoliids and monocots are sister clades) as a possible relationship worthy of further study and we further performed molecular dating (Supplementary Note 4.5 and Fig. 4d). The crown age of angiosperms was inferred to be 190–315 million years ago (Ma). The split between monocots and magnoliids was estimated at 138–241 Ma and the divergence time between the magnoliid + monocot clade and eudicots was at 143–249 Ma. As noted in a previous study^1^, the temporal proximity of the split among magnoliids, monocots and eudicots (within ~7 Ma) and broadly overlapping divergence time confidence intervals indicate that rapid divergence, is probably responsible for the great difficulty in reconstructing the relationship using a phylogenomic approach based on sequence data (Supplementary Note 4.5).

### The genetic basis of unique floral features in *Aristolochia*

**Fig. 5:**
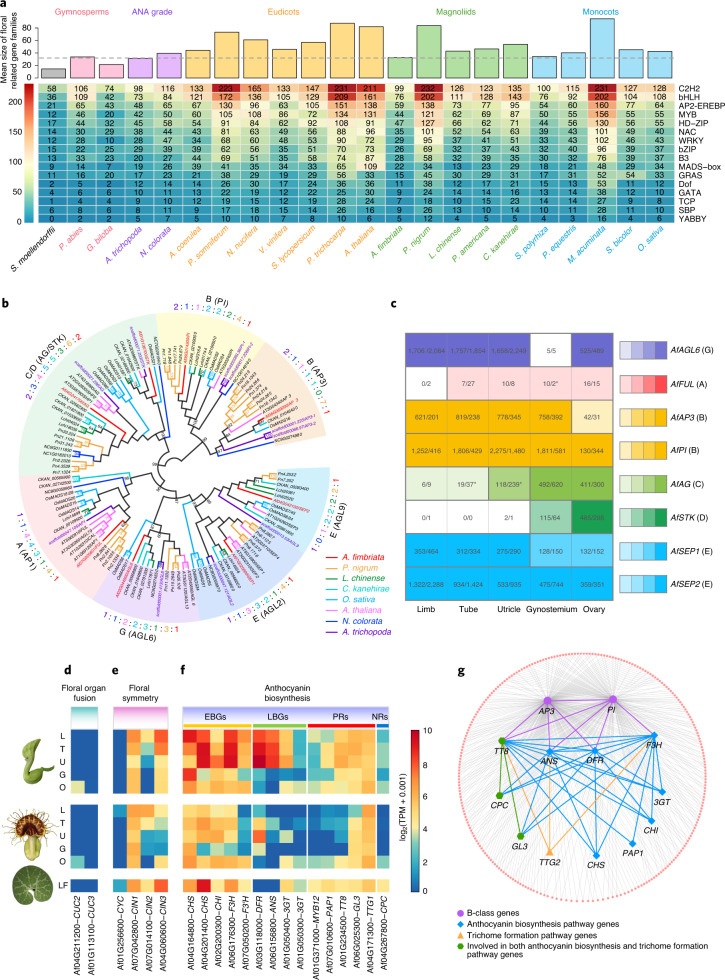
Using the *A. fimbriata* genome to elucidate the molecular developmental genetics of a highly specialized flower. **a**, Variation in the copy numbers of flowering-associated transcription factors during land plant evolution. *A. fimbriata* and *A. trichopoda* exhibit the lowest mean size for the investigated gene families. **b**, Phylogenetic inference of floral organ identity genes. Branches of the maximum likelihood tree were coloured on the basis of the species colour scheme (on the right). BS > 50% are shown. The numbers of floral organ identity genes are also shown and coloured according to the species colour scheme. **c**, The expression patterns of the floral organ identity genes. The numbers in the boxes are the TPM expression values for each gene at the pre-anthesis and anthesis stages. The relative expression levels were further normalized by calculating the ratio of their TPM expression values to that of the functionally conserved *AfAP3* gene in the gynostemium. The ratios were illustrated by four colour gradations representing 0.01–0.1, 0.1–0.25, 0.25–0.5 and >0.5. No colour was filled if the gene has no expression. The asterisks indicate genes with different relative expression levels between the two examined developmental stages; heatmap colours correspond to the relative expression levels in pre-anthetic flowers. **d**–**f**, Expression levels of the putative candidate genes involved in floral organ fusion (**d**), floral symmetry (**e**) and anthocyanin biosynthesis (**f**), in late pre-anthetic and anthetic flowers and leaves (LF). L, limb; T, tube; U, utricle; G, gynostemium; O, ovary; EBGs, early biosynthesis genes; LBGs, late biosynthesis genes; PRs, positive regulators; and NRs, negative regulators. **g**, Co-expression network reconstruction identified MADS-box B-class genes clustered with the genes involved in anthocyanin biosynthesis, as well as several trichome formation genes, suggesting that floral organ identity genes have expanded their regulatory networks. Source data

**Extended Data Fig. 8:**
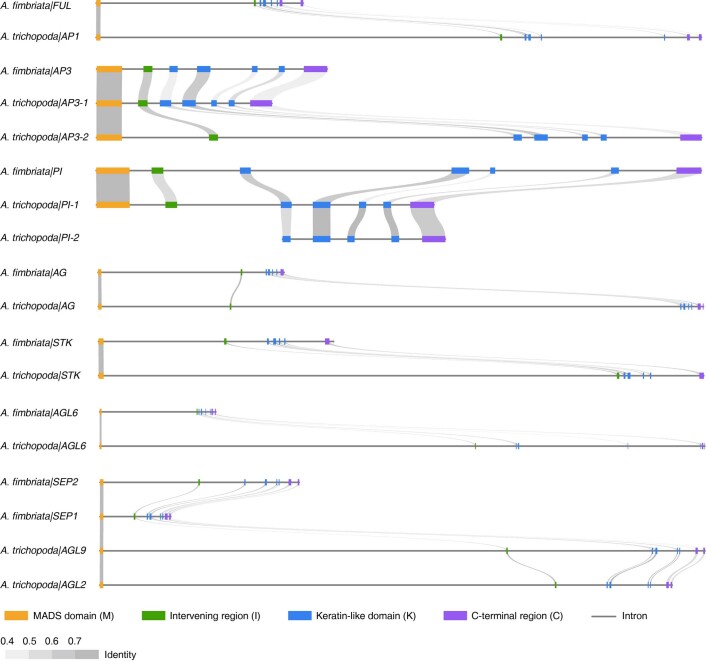
Comparisons of gene structure and exon sequence similarity of the floral organ identity genes in *A. fimbriata* and *A. trichopoda*. The *A. trichopoda* genes tend to have longer introns than that of *A. fimbriata*.

*Aristolochia* has a unique floral morphology that consists of a monosymmetric, trumpet-shaped, petaloid perianth and a gynostemium formed by the congenital fusion between stamens and the stigmatic region of the carpels (Fig. 1a and Extended Data Fig. 1). The *A. fimbriata* genome contains a relatively small number of floral regulatory genes (Supplementary Note 5.1, Fig. 5a, Supplementary Fig. 5.1 and Supplementary Table 5.2) and only one homologue for each of the eight classes of floral organ identity genes with high similarity to their corresponding orthologues in *Amborella* (Fig. 5b and Extended Data Fig. 8). Among the floral organ identity genes, *AfAP3* and *AfPI* are highly expressed in the perianth, suggesting that the petaloidy of the perianth was caused by outward expansion of the expression domains of B-function genes and supporting the hypothesis of a sepal-derived perianth in *Aristolochia*. Also, both B-function genes and *AfAG* are expressed in the gynostemium, supporting the hypothesis that the gynostemium is a fused structure (Supplementary Note 5.3 and Fig. 5c). Two *CUP-SHAPED COTYLEDON* genes, *AfCUC1* and *2*, whose orthologues in other species specify the boundaries between floral organs^43,44^, were also identified in *A. fimbriata* (Supplementary Note 5.4 and Supplementary Fig. 5.5). Consistent with the formation of the trumpet-shaped perianth and the fusion of stamens and the stigmatic region of the carpels, neither of these genes is expressed in the perianth or gynostemium (Fig. 5d). Notably, the *A. fimbriata* genome contains one *CYCLOIDEA* (*CYC*) and three *CINCINNATA* (*CIN*) genes (Supplementary Fig. 5.6), which are orthologues of the flower symmetry establishment and leaf-like organs morphogenesis genes in other species^45–47^. While the expression levels of *AfCYC* are very low in all of the tissues examined, the three *CIN* genes (that is, *AfCIN1*, *2* and *3*) show differential expression basipetally, with the highest expression being found in the limb region (Fig. 5e). This evidence, together with the observation of their expression profiles in *Aristolochia arborea* and *A. fimbriata*^48,49^, strongly suggests that the *CIN* genes are responsible for the heterogeneous growth and morphological deformation of the perianth in *Aristolochia*.

*Aristolochia* flowers often exhibit a dull, purple-brown colour in different parts of the perianth, probably related to pollinator attraction^24^. In the *A. fimbriata* genome, we identified 13 putative anthocyanin biosynthetic genes, consistent with the previously known pigmentation stages^50^, several key enzyme-encoding genes, such as *CHALCONE SYNTHASE* (*CHS*), *FLAVANONE 3-HYDROXYLASE* (*F3H*), *DIHYDROFLAVONOL 4-REDUCTASE* (*DFR*) and *ANTHOCYANIDIN SYNTHASE* (*ANS*), showed relatively higher expression in the pre-anthetic flowers compared to anthetic flowers (Fig. 5f). It is very likely that the *A. fimbriata* flowers lack delphinidin-based anthocyanins because none of the identified candidate genes encode for flavonoid 3′5′-hydroxylase (F3′5′H), a key enzyme for the synthesis of delphinidin-based lilac to blue anthocyanins^51,52^. In addition, the B-function genes (*AfAP3* and *AfPI*) are positively co-expressed with three structural genes (*F3H*, *DFR* and *ANS*) and a regulatory gene (*TRANSPARENT TESTA 8*, *TT8*), suggesting that they may regulate anthocyanin biosynthesis (Supplementary Note 5.5 and Fig. 5g). The observation that putative *AP3*/*PI*-specific binding motifs (CArG-box) can also be found in the promoter regions of the *F3H*, *DFR*, *ANS* and *TT8* genes further supports this idea (Supplementary Table 5.5). Further analysis of anthocyanin biosynthesis in the flowers of *A. fimbriata* is warranted.

### Terpenoid and AA biosynthesis in *A. fimbriata*

**Fig. 6:**
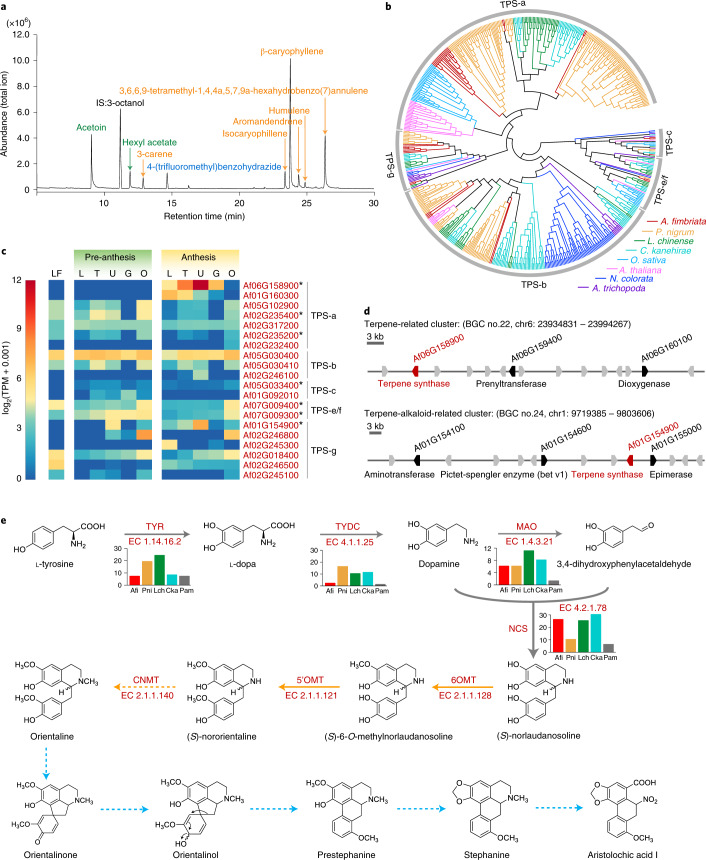
Terpenes and aristolochic acid I biosynthesis in *A. fimbriata*. **a**, Gas chromatogram of floral volatiles from anthetic flowers of *A. fimbriata*. The internal standard (IS) is 3-octanol. Fatty acid derivatives are coloured in green; benzenoid is coloured in blue; and terpenoids (sesquiterpenes and monoterpenoids) are coloured in orange. **b**, The phylogenetic inference of the TPS gene family using a maximum likelihood tree. Branches are coloured according to the species colour scheme on the bottom right. **c**, Expression patterns of the TPS genes in leaves and pre-anthetic and anthetic flowers. The TPS genes marked by stars were additionally annotated as occurring within terpene-related biosynthetic gene clusters. **d**, Two annotated terpene biosynthesis-related gene clusters in the *A. fimbriata* genome. An analysis integrating phylogenetic inference with expression pattern data suggests that *Af06G158900* and *Af01G154900* are functionally consequential sesquiterpene and monoterpene synthase genes, respectively. **e**, Our proposed aristolochic acid I biosynthesis pathway. The first four steps (grey arrows) are similar to the benzylisoquinoline alkaloid (BIA) biosynthesis pathway^121–123^; the subsequent two steps (orange solid arrows) are predicted and constructed on the basis of individual reactions in KEGG and previous studies^124–126^; the next step (orange dotted arrows) is predicted according to previous studies^127,128^; and the last five steps (blue dotted arrows) are predicted on the basis of previous tracer experiments^121^. Source data

Because of their enriched secondary metabolites, *Aristolochia* species have long been used in traditional pharmacopeias^27^. In the *A. fimbriata* genome, 1,803 genes belonging to ~20 secondary metabolism pathways (including isoquinoline alkaloid biosynthesis, tyrosine metabolism and other alkaloid biosynthesis pathways) were annotated (Supplementary Table 6.1). Thirty-three metabolic biosynthetic gene clusters (BGCs), which were annotated as alkaloid-, polyketide-, saccharide- and terpene-related clusters, were also found (Supplementary Fig. 6.1 and Supplementary Table 6.2). The large proportion of the annotated terpene (14/33) and alkaloid-related (9/33) BGCs appears to associate with the enriched production of terpenoid and alkaloid compounds in *A. fimbriata* (Fig. 6a)^21,27,53^.

Specifically, our GC–MS analyses detected complex volatile compounds, including fatty acid derivatives, benzenoids and two types of terpenoids (sesquiterpenoids and monoterpenoids) (Fig. 6a) but no diterpenoids in the *A. fimbriata* flowers. In the *A. fimbriata* genome, 41 putative terpene synthase (TPS) genes were identified and phylogenetic analyses further classified them into TPS-a, TPS-b, TPS-c, TPS-e/f and TPS-g subfamilies (Fig. 6b). TPS-a genes often encode sesquiterpene synthases^54^. Notably, the *Af06G158900* locus from the TPS-a clade exhibited extremely high expression in the utricle of anthetic flowers (Fig. 6c), which is consistent with the abundant component of sesquiterpene detected in anthetic flower volatiles (Fig. 6a). Because it was also annotated in the terpene-related gene cluster (BGC 22; Fig. 6d), it is very likely that *Af06G158900* is a main sesquiterpene synthase-coding gene in *A. fimbriata* (Supplementary Note 6.2 and Fig. 6a–d). The other gene that presents a similar case is *Af01G154900*, which codes for a monoterpene synthase (Fig. 6a–d). In contrast, the genes in the TPS-c and TPS-e/f clades, which are responsible for the biosynthesis of diterpenoids^54,55^, showed very low expression in both pre-anthetic and anthetic flowers (Fig. 6b,c). Presumably, it is the low expression of these genes that is responsible for the lack of diterpenoids in *A. fimbriata* flower volatile compounds.

**Extended Data Fig. 9:**
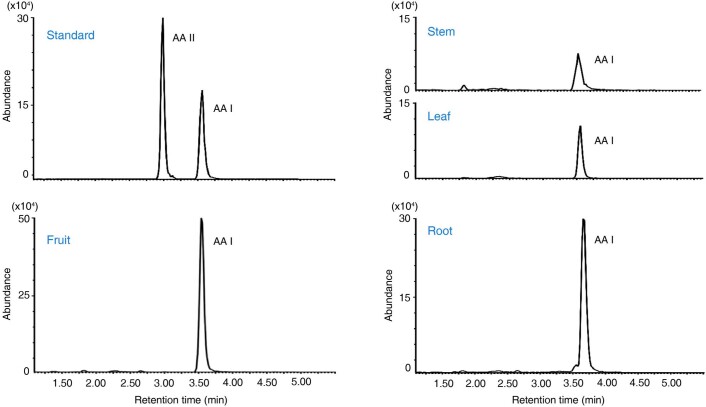
LC–MS analysis of aristolochic acid content in *A. fimbriata*. The purchased samples of AA I and AA II were used as standards, and the samples of fruit, stem, leaf, and root were analysed by LC–MS. Only AA I was detected in the investigated tissues of *A. fimbriata*.

**Extended Data Fig. 10:**
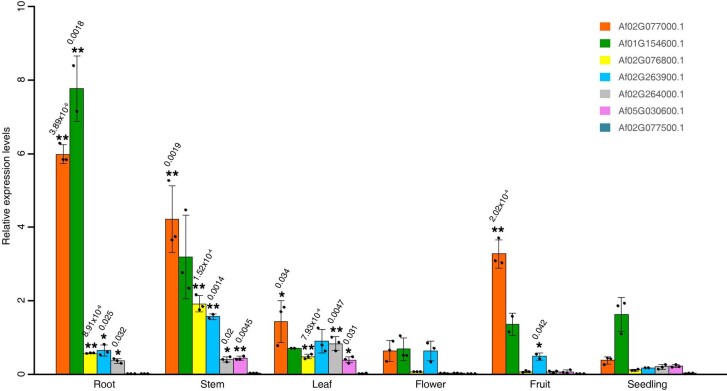
Gene expression quantification by qRT–PCR for the seven NCSI genes in *A. fimbriata*. All data are presented as the means ± s.d. (n = 3 biological replicates, as shown in solid black dots). The NCSI gene expression levels in five other tissues (root, stem, leaf, flower, and fruit) were compared with that in seedlings, and the two-tailed t tests were used to analyse the statistical significance of their expression levels. * indicates a significant difference at *P* value < 0.05, and ** indicates a significant difference at *P* value < 0.01, *P* values are shown above each bar chart. Source data

Given the widely known toxicity problems with AAs—major toxic alkaloid compounds present in many popular medicinal plants of Aristolochiaceae^27–29,53^—we also explored the *A. fimbriata* genome assembly to yield some insights into AA biochemistry. After liquid chromatography–mass spectrometry (LC–MS)-based confirmation of the accumulation of an AA compound (AA I) in *A. fimbriata* tissues (Extended Data Fig. 9), we constructed the AA I biosynthesis pathway on the basis of the previous studies (Supplementary Table 6.4) and identified the main enzymes involved (Supplementary Note 6.3 and Fig. 6e). Our extensive metabolic enzyme annotation, gene family phylogeny construction and key catalytic motif/residues investigations led to the putative identification of the main candidate genes encoding these associated enzymes (Supplementary Note 6.4). For example, norcoclaurine synthase (NCS) is crucial for the biosynthesis of benzylisoquinoline alkaloids (BIAs) in Ranunculaceae, Papaveraceae, Berberidaceae and Nelumbonaceae^56,57^. Phylogenetic analysis found seven *NCS* genes that were grouped together with the known alkaloid biosynthetic genes of opium poppy (*Papaver somniferum*) in the *NCS* *I* clade (Supplementary Fig. 6.8). Six of them (*Af02G077000*, *Af02G076800*, *Af02G263900*, *Af02G264000*, *Af01G154600* and *Af05G030600*) were annotated in alkaloid-associated gene clusters (BGC 1, 10, 24 and 25) (Supplementary Fig. 6.1 and Supplementary Table 6.2) and their amino acid sequences exhibit conserved catalytic residues (Supplementary Fig. 6.9). Notably, the expression levels of the two genes (*Af02G077000* and *Af01G154600*) were highly correlated with the concentration of AA I in the examined tissues (Extended Data Fig. 10), suggesting their roles in encoding the main functional norcoclaurine synthase in *A. fimbriata*.

## Discussion

The tremendous diversification of angiosperms can be at least partially attributed to prevalent WGDs throughout their evolutionary history^15,31,35,58–62^. Previously, *Amborella* was considered the sole angiosperm genome lacking a lineage-specific WGD, possessing only the single WGD event characteristic of all extant angiosperms^32^. Our work establishes that *A. fimbriata* is the second among the several hundred sequenced flowering plant genomes to retain this ancestral genomic condition; this genome sequence therefore offers exceptional opportunities for unravelling the WGD history and genomic changes of other lineages, especially other magnoliids. Moreover, genomic analysis anchored by *Amborella* and *A. fimbriata* can ultimately deepen our understanding of genome evolution across angiosperms^36^. The well-conserved synteny between *A. fimbriata* and *Amborella* also enables a more resolved reconstruction of the ancestral angiosperm genome and thus provides insights into the genomic features of the common ancestor of extant angiosperms.

The *A. fimbriata* genome may help to clarify early mesangiosperm diversification and the phylogenetic placement of magnoliids through analysis of the evolutionary history of genomic structural variations, as we demonstrate here. Recent studies have used a phylogenomic approach to determine the relationship among the monocot, eudicot and magnoliid clades^4–11,15–20^ but have often recovered different topologies (Supplementary Table 4.4). After comprehensively testing alternative taxon sampling and tree-constructing strategies, we also found it challenging to resolve with strong support the relationship among these three clades (Supplementary Note 4.2). Moreover, codon usage bias also affected the resolution of the tree as well as the topology (Supplementary Note 4.4 and Supplementary Figs. 4.12–4.15). The difficulty in resolving relationships among these clades may be due to the limited informative sequence divergence generated during their rapid diversification. In such cases, it is plausible that some rare genomic changes, such as genomic structural changes, may potentially have occurred in a very compressed evolutionary window. Because rare genomic changes have more alternative states and may be less vulnerable to the high frequency of reversals or parallel substitutions in sequence evolution, they can offer valuable insights into the phylogenetic relationships as proposed previously^63,64^. Although it remains hard to completely exclude the possibility of ancient hybridization, parallel evolution and ILS, the identified genome structural changes most parsimoniously imply a sister relationship between magnoliids and monocots, a relationship that has also been recovered in another study^42^. We stress however, that other key mesangiosperm lineages (Chloranthales and Ceratophyllales) are not included in these analyses and it will be crucial to investigate their patterns of genomic rearrangement.

The genome assembly of *A. fimbriata* also serves as a functional genomic resource for pinpointing the genetic bases for the origins and modifications of phenotypic traits, such as the highly modified flower and the enriched alkaloid chemistry of *A. fimbriata*. Gene duplication is considered to be a driving force for the evolution of phenotypical and functional novelty. Here, we found similar numbers of MADS-box genes, as well as other floral regulators, between *A. fimbriata* and *Amborella*, two species with dramatically different flower morphologies^23,65^. We also noted that alternative splicing variant forms for these genes are very rare in *A. fimbriata* (Supplementary Note 5.2 and Supplementary Fig. 5.3). At minimum, these findings suggest that MADS-box gene repertoire has not expanded in *A. fimbriata*, excluding one of the possible mechanisms of flower diversification via gene duplication and neofunctionalization^66,67^. The expanded regulatory networks involving the floral organ identity genes and genes associated with other developmental features identified in this study can help at least partially explain the morphogenesis of the highly modified flowers of *A. fimbriata*. Further comparative analyses of expression profiling and chromatin immunoprecipitation followed by sequencing (ChIP–seq) of MADS-box genes in *A. fimbriata* and *Amborella* could be used to better understand the evolutionary developmental mechanism of the distinct flowers in *A. fimbriata*.

In conclusion, the *A. fimbriata* genome lacks any additional WGDs beyond that shared by all extant angiosperms. Thus, it provides an outstanding new evolutionary reference for comparative genomics and for inferring the ancestral angiosperm genome and patterns and processes of genome evolution in other angiosperms. The *A. fimbriata* genome has also facilitated the identification of genomic structural changes, which is shared with other magnoliids and with monocots, suggesting a sister relationship between magnoliids and monocots, in contrast to many sequence-based analyses that have found monocots and eudicots to be sisters. Finally, the genome also provides insights into the genetic basis underlying both the highly specialized flower development and aristolochic acid biosynthesis. Given its low genetic redundancy and ease of large-scale cultivation, *A. fimbriata* could readily be developed into an important new genetic model species given its phylogenetic position as a member of the magnoliid clade; the species affords opportunities for further functional genomic studies, serving as an excellent system for studies of floral biology, developmental genetics, biochemical pathways and development of synthetic chemicals.

## Methods

### Plant materials and DNA sequencing

Fresh leaves were collected from the same individual of *A. fimbriata* plant for DNA extraction and sequencing. For Oxford Nanopore Technologies (ONT) sequencing, DNA was extracted from young leaves using QIAGEN Genomic Kits and libraries with an insert size of 20–40 kb were then prepared and sequenced on a GridION X5 instrument. For optical maps, DNA was extracted from young leaves according to a modified Bionano genomics protocol^68^. The long high-quality DNA was labelled by enzyme Nt.BspQI and then loaded into the Saphyr chip for scanning. To collect sufficient material for Hi-C sequencing, we cultivated the seedlings by tissue culture using stem cuttings from the same individual used for the above sequencing. The samples were processed and the DNA was extracted and crosslinked using the standard protocol. The Hi-C libraries were then amplified and sequenced with 150-bp paired-end reads using Illumina HiSeq.

### Genome assembly and assessment

ONT long reads were de novo assembled using minimap2 v.2.15-r914 (ref. ^69^) and miniasm v.0.3 (ref. ^70^). Then, three rounds of polishing with racon^71^ and one round of polishing with Pilon^72^ were applied to the assembled contigs. Optical molecules with length >180 kb or the molecule label number >9 were used for optical map assembly using the Bionano Solve Pipeline v.3.3 (https://bionanogenomics.com/support/software-downloads/) and hybrid scaffolds were generated by aligning the optical maps to ONT assembled genomic contigs using Bionano’s hybrid-scaffold software (https://bionanogenomics.com/support/software-downloads/). The hybrid scaffolds with length >100 kb were further anchored and oriented to seven pseudochromosomes on the basis of the Hi-C contact map between genomic loci using 3D-DNA v.180114 (ref. ^73^). We also manually corrected the order or orientation of several misassembled scaffolds on the basis of the Hi-C contact frequency using Juicebox Assembly Tools (JBAT v.1.8.8)^74^.

The quality and completeness of the *A. fimbriata* genome assembly were assessed from four aspects. First, we evaluated the mapping rates of the clean raw reads from transcriptomes and genomic DNA by TopHat2 (ref. ^75^) and BWA-MEM (ref. ^76^) with default parameters, respectively. We further used the ‘—vcf’ option in Pilon v.1.23 (ref. ^72^) to call single nucleotide polymorphisms from the Illumina genomic reads. Second, we investigated the BUSCO genes from Embryophyta in the final assembly^33^. Third, we used the LAI to infer the assembly continuity^34^. Finally, we aligned Bionano molecules back to the final *A. fimbriata* genome assembly to check the consistency between Bionano molecules and the final genome assembly using the RefAligner tool (https://bionanogenomics.com/support/software-downloads/) with default parameters. In addition, we also checked the consistency of the Bionano assembly consensus genome maps (CMAP) and the in-silico maps of the *A. fimbriata* genome assembly.

### Transcriptome sequencing

Several organs and tissues were sampled for total RNAs extraction and transcriptome sequencing, including leaves, seedlings under normal and low temperature (4 °C) conditions, roots and five different floral organs (limb, tube, utricle, gynostemium and ovary). For Illumina RNA-seq sequencing, total RNA from young leaves and five different floral organs at different developmental stages (stage 8 and anthesis flower) were separately extracted and processed using Trizol reagent (Invitrogen) following the manufacturer’s procedure. The paired-end complementary DNA libraries with insert size of 150 bp were constructed and sequenced using Illumina HiSeq4000 instrument. For full-length transcriptome sequencing, the samples from anthetic flowers, seedlings under normal growth conditions, seedlings treated with low temperature (4 °C) for 9 h and roots were collected and the extracted RNAs from the four samples were mixed together in equal amount to obtain transcriptomes from various plant tissues and treatments. The cDNA libraries were constructed using the SMARTer PCR cDNA Synthesis Kit. The full-length cDNA fragments were screened using a BluePippin instrument to construct cDNA libraries of different sizes (1–2, 2–3 and 3-6 kb) (Supplementary Fig. 2.2). The libraries were sequenced on a PacBio RS II instrument. In addition, we further collected and pooled the flower buds at different developmental stages (from stage 5 to anthesis)^50^ together in relatively equal amount to perform much deeper transcriptome sequencing to get the potential alternative splicing transcripts for floral genes. The extracted RNA from the mixed sample was used for isoform sequencing (Iso-seq) on the PacBio Sequel II platform.

### Repeat annotation

TEs were identified using a combination of evidence-based search and ab initio prediction approaches. For evidence-based search, *A. fimbriata* genome was searched against the Repbase database v.20.05 (ref. ^77^) using RepeatMasker v.4.0.7 (ref. ^78^) with default parameters. For ab initio prediction, a consensus sequence library was built using RepeatModeler v.1.0.10 (http://repeatmasker.org/RepeatModeler/) with the parameter ‘-engine ncbi’. Then, LTRharvest v.1.5.10 (ref. ^79^), LTR_FINDER v.1.05 (ref. ^80^) and LTR_retriever v.1.8.0 (ref. ^81^) were used to build an LTR library with default parameters. These two libraries were used to annotate the *A. fimbriata* genome using RepeatMasker and the detected TEs were then combined to obtain the final TE annotation. Results from these two runs of RepeatMasker were merged.

### Protein-coding gene prediction and functional annotation

The protein-coding genes were predicted using the well-developed combination strategies of transcriptome, homology-based annotation and ab initio gene prediction. For the ab initio prediction, Fgenesh^82^ and AUGUSTUS^83^ were run on the repeat-masked scaffolds. For the homology-based prediction, we used the inferred amino acid sequences from the *A. coerulea*, *A. comosus*, *Arabidopsis thaliana*, *A. trichopoda*, *P. somniferum* and *C. kanehirae* genomes. GeneWise^84^ and GeMoMa^85^ were used to annotate the gene models using alignments from amino acid sequence similarity against the *A. fimbriata* assembled sequences. For transcriptome-based prediction, PASA^86^ and GMAP^87^ were used to predict the gene models. If the transposable domain occupied >60% of the predicted gene length, the gene was removed using TransposonPSI (http://transposonpsi.sourceforge.net). Finally, the results from the three approaches were integrated to generate EVidenceModeler (EVM)^88^ gene models to obtain the final annotated protein-coding gene set.

The putative functions of the genes were predicted by searching the best-matched proteins in SwissProt (https://web.expasy.org/docs/swiss-prot_guideline.html), non-redundant (Nr) (https://ftp.ncbi.nlm.nih.gov/blast/db/FASTA/) and Eukaryotic Orthologous Groups (KOG) (https://hsls.pitt.edu/obrc/index.php?page=URL1144075392) databases using BLASTP (*E*-value ≤ 10^–6^). Gene ontology terms were also assigned to the genes by combining the results from Blast2GO v.5.2.5 (ref. ^89^) and eggNOG-mapper v.22 (ref. ^90^) annotations. We also used the KEGG database (https://www.genome.jp/kegg/) to obtain KEGG orthologues to infer putative gene pathways.

### Gene family classification and comparison

We selected 22 species to construct putative gene families (for detailed sampling information see Supplementary Table 2.8). The longest transcript isoform for each locus was selected for all-versus-all BLASTP^91^ with an *E*-value cutoff setting of 10^−5^. OrthoMCL v.2.0.9 (ref. ^92^) was used to identify gene clusters of putative gene families and the inflation parameter was set to 1.5 in the mcl process^93^. The output from OrthoMCL was summarized using a custom Python script to obtain the number of genes from each species belonging to the orthogroups. Venn diagrams of the selected taxa were generated using InteractiVenn (http://www.interactivenn.net/).

### Genome structural comparisons and polyploidization analysis

Except for *A. fimbriata*, seven other genomes were selected for polyploidization analysis: *A. trichopoda*, *N. colorata*, *P. nigrum*, *C. kanehirae*, *P. americana*, *L. chinense* and *V. vinifera*. For synteny analyses, we first performed all-against-all BALSTP (*E*-value < 10^-5^ and score > 100) within and between genomes. Then, the top ten BLAST matches are selected for inferring syntenic blocks within or between genomes. We used MCScanX^94^ to identify syntenic blocks by setting the maximum gap between the anchor genes to 25. We further plotted the syntenic gene pairs according to their genomic locations in dotplots and used different colour-coded dots to distinguished whether the anchor gene pairs are the best BLAST hit within/among the genomes. Finally, we inferred the WGD history by investigating the syntenic depth ratios within and among genomes.

The median Ks values of syntenic anchor genes were further used to determine the divergence degree of the identified syntenic blocks. First, Ks was estimated using the Nei–Gojobori approach^95^ implemented in the Bioperl Statistical module. Then, we adopted a kernel function analysis to obtain the Ks distribution, which was further simulated as a mixture of multiple normal distributions by the kernel smoothing density function (Ks density, width was set to 0.05). Lastly, we performed the Gaussian multipeak fitting of the curve by using the Gaussian approximation function (cftool) in MATLAB, and set the R-squared >95% which is a parameter to evaluate the fitting level. The smallest number of normal distributions was used to represent the multiple peaks of the Ks distribution.

To investigate the timing of previously identified WGDs in magnoliids, we used the integrated approaches of synteny, Ks and phylogenomic analyses similar to previous research^40,41^. Here, Ks correction was applied by using grape (*V. vinifera*) as a comparing reference to make its divergence (Ks) similar to the studied magnoliid genomes, similar as in the previous studies^96,97^.

To track the evolutionary history of the genomic rearrangement events, we first identified orthologous genomic regions on the basis of generated syntenic dotplots. Then, we defined the involved regions of the genomic rearrangements and revealed the connection pattern of these orthologous regions in each studied genome. Next, we reconstructed the ancestral connection pattern of these involved regions for the major clades of angiosperms on the basis of orthologous regions in living species. Finally, we compared the ancestral pattern of each clade with the predicted pattern of the most common ancestor of extant angiosperms and identified the shared genomic rearrangements of major clades that potentially occurred before their divergence.

### Phylogenetic analysis

To comprehensively analyse the phylogenetic position of magnoliids, we performed phylogenomic analyses using different datasets and approaches (Supplementary Table 4.1). Two strategies were used for screening orthogroups on the basis of gene copy number: the SSC and MSC gene families. For SSC gene families, because the genomes of *P. nigrum* and *P. somniferum* each experienced a very recent WGD event^4,98^, we allowed them two gene copies at most and the other 20 species strictly a single gene.

For phylogeny reconstruction, protein sequences from each gene family were aligned using MUSCLE v.3.8.31 (ref. ^99^) and nucleotide sequences were then forced to fit the amino acid alignments using PAL2NAL v.14 (ref. ^100^). We also forced nucleotide sequences on the amino acid alignments using a custom Python script to obtain codon-preserving alignments of nucleotide sequences. Finally, we retrieved four different alignments for each gene family to perform phylogenetic analyses: (1) amino acid (or peptide, pep) alignments; (2) nucleotide alignment (nucleotides forced to the amino acid alignment; or coding sequence, cds); (3) codon alignments with third-position removed (codon1&2); and (4) codon alignments with first- and second-position removed (codon3). For the concatenation-based analyses, gene alignments were concatenated as a single supermatrix and the tree was inferred under the ‘PROTGAMMAAUTO’ and ‘GTRGAMMA’ model of amino acid and nucleotide substitution using RAxML v.8.2.12 (ref. ^101^). For coalescent-based analyses, we constructed individual gene trees by 100 rapid bootstrapping replicates and searching for the best-scoring maximum likelihood (ML) tree in one single run (-f a option); we checked the bootstrap support (BS) values for the nodes associated with the phylogenetic relationship among monocots, eudicots and magnoliids and summarized the topologies with BS values ≥0, 10, 50 or 80%, respectively; the individual ML gene trees with different BS cutoff values were then used by ASTRAL-II v.5.5.11 (ref. ^102^) with local posterior probability (LPP). We also used another coalescent-based method, MP-EST, to carry out additional phylogenetic analyses. In addition, we used ASTRAL-Pro and STAG to perform a phylogenetic analysis of all gene families containing paralogue genes^103,104^.

To investigate the extent of incongruence that is present in the phylogenomic data matrix, we performed the following two assessments for ML trees on the basis of amino acid and nucleotide sequences, respectively. First, we used phyparts v.0.0.1 (ref. ^105^) to count the number of genes supporting certain topologies. Secondly, we used built-in LPPs of ASTRAL to estimate branch support and to test for polytomies^106,107^.

To investigate the impact of taxon sampling on phylogenomic analyses, we constructed datasets of differently selected species in eudicots, monocots, magnoliids and the *A. trichopoda* (sister to all other extant angiosperm). Associated single-copy gene families were extracted from orthoMCL results by custom Python scripts and the concatenation- and coalescent-based phylogenetic analyses were performed. All of these analyses were rooted with *Amborella*.

For chloroplast genes, we used the same set of 22 species in the above nuclear genome phylogenomic analyses as in Supplementary Note 4.1. Here, the chloroplast genome of *Nuphar advena* was used to represent Nymphaeales instead of *N. colorata*, because the chloroplast genome of *N. colorata* has not been fully annotated^38^. We manually checked the chloroplast genomes and extracted 79 protein-coding genes from the selected genomes. The concatenation-based analyses for amino acid, nucleotide, codon1&2 and codon3 sequences were performed with 1,000 bootstrap replicates respectively, as described above. In addition, the coalescent-based phylogeny was also inferred from the individual ML gene trees with BS ≥ 50% using ASTRAL-II v.5.5.11 (ref. ^102^).

### Estimation of divergence time

Divergence times of each tree node were inferred using the program MCMCTree in the PAML v.4.9e package^108^. The species tree constructed with the 98 SSC gene families from 22 species (T3 topology) and rooted with *S. moellendorffii* was used as the input tree. Following fossil dates were used for the calibration procedure: maximum age of 400 Ma for the divergence of *S. moellendorffii*^109^, a minimum age of 309 Ma for the crown-group seed plants^110^, a minimum age of 125 Ma for the eudicots^111^, a maximum age of 113 Ma for the monocots^112–114^ and a maximum age of 113 Ma for the magnoliids^115^. Branch lengths were estimated using BASEML from the PAML package under the GTR + G model (model = 7)^108^. The overall substitution rate (rgene gamma) and rate-drift parameter (sigma2 gamma) were set as G (1, 5.6) and G (1, 4.0) respectively. We ran all analyses twice to check for consistency and to ensure the effective sample size was >200 in Tracer v.1.7 (http://tree.bio.ed.ac.uk/software/tracer/).

### Transcriptomic data analyses

RNA-seq raw reads were preprocessed using Trimmomatic^116^ to remove adaptor sequences and low-quality reads. The clean reads were then mapped to the reference genome using HISAT2 with default parameters. The expression abundance values were calculated using Stringtie^117^ and we averaged the abundance values from the three biological replicates of each sample to obtain levels of gene expression.

For the Iso-seq data of mixed tissues sequenced on PacBio RS II instrument, the raw reads were processed using SMRT Link 5.0 software. First, the circular consensus sequences (CCSs) were generated from the subreads BAM files with parameters of ‘--minLength=300 --minPasses=1 minPredictedAccuracy=0.8’. Next, all the CCSs were further classified into full-length non-chimaeric (FLNC) and non-full-length (nFL) transcript sequences on the basis of whether the 5′-primers, 3′-primers and poly(A) tail could be detected. To improve consensus accuracy, we clustered and polished the FL sequences using an isoform-level clustering algorithm, iterative clustering for error correction (ICE) and the Quiver tool in the SRMT Link software. The FL reads were further corrected using RNA-seq reads using LoRDEC^118^ with the parameters of ‘-k 19 -s 3 -T 4’ and redundancy was removed using Cd-hit^119^ with the parameters of ‘-c 0.99 -T 10 -G 0 -aL 0 -aS 0.99 -AS 30 -d 0 -p 1’.

For the Iso-seq data of mixed flower buds sequenced on PacBio Sequel II platform, the raw sequence data were processed by SMRT Link v.8.0 software (https://www.pacb.com/support/software-downloads/). First, CCSs were generated from the raw subreads BAM file to identify full-length (FL) reads using CCS with parameters of ‘--min-passes 1 --min-length 100’. Then, FLNC reads were identified if they have the 5’-primer, 3’-primer and poly(A) tail. Lastly, FLNC reads from the same isoform were clustered and further polished using subreads.

### The construction of co-expression networks

For the construction of co-expression networks, we used all RNA-seq data from 14 samples described above (tissues of flowers at anthesis and pre-anthesis, leaves and seedlings with different treatment) and required genes with transcripts per million (TPM) ≥ 1 in at least one of the samples to be included in the analysis. Pearson correlation coefficients (PCCs) for each bidirectional gene pair were calculated to quantify the correlations. Then, we ranked the PCC values by mutual rank (MR) algorithm to identify the highly correlated gene pairs. Finally, gene pairs with MR ≤ 300 were referred to as co-expressed genes^120^.

### Floral scent measurement

To investigate the floral volatile production of *A. fimbriata*, we collected the newly opened flowers for gas chromatography–mass spectrometry (GC–MS) analysis, with the added 0.0825 μg of 3-octanol as an internal standard. Then, the samples were incubated at 40 °C for 30 min. The volatiles were further extracted using SPME fibre with 50/30 μm of divinylbenzene/carboxen/polydimethylsiloxane (DVB/CAR/PDMS) (Supelco Co.). Finally, GC–MS analysis was conducted on an Agilent 7890B gas chromatograph coupled to a mass spectrometer (Agilent 7000D) with a fused silica capillary column (HP-5MS) coated with polydimethylsiloxane (19091S-433UI) (30 m × 0.25 mm internal diameter, 0.25 μm film thickness). The oven temperature was programmed to start at 40 °C for 3 min and then ramped to 130 °C at a rate of 5 °C min^–1^, followed by a second ramp to 156 °C at a rate of 2 °C min^–1^ and the final ramp to 280 °C at a rate of 10 °C min^–1^. Three biological replicates were conducted for the GC–MC analysis.

### Aristolochic acid identification

We performed an LC–MS-based metabolomic analysis for the root, stem, leaf and fruit from one-year-old *A. fimbriata* plants. A total 50 mg of each dried tissue were processed for the HPLC-DAD-ESIMS/MS measurements. AAs were separated by UPLC (Waters, ACQUITY) equipped with an ACQUITY UPLC HSS T3 column (Waters) and detected by MS/MS using a Triple Quad Xevo TQ-S (Waters) mass spectrometer. The mobile phase consists of buffer A (5 mM ammonium acetate and 0.1% formic acid) and buffer B (100% acetonitrile). AAs were qualified using the ion mass transitions of *m/z* 324.1/237 and 324.1/280 for AA I and *m/z* 329/238 and 329/268 for AA II, respectively, and the base ions were ammonium adduct ions [M + NH^4^]^+^. For quantitative analysis, we used a higher abundance of the adduct ion mode. Standard curves were generated by running a concentration series of pure commercial AAs. The content of AAs in each sample was then calculated by fitting the peak areas to the standard curves.

### Reporting Summary

Further information on research design is available in the Nature Research Reporting Summary linked to this article.

## Supplementary information

Supplementary Information Supplementary Figs. 1.1–6.9, materials and methods, and detailed description of results and discussion.

Reporting Summary

Supplementary Tables Supplementary Tables 1.1–6.7.

## Data Availability

All of the raw sequence reads, nuclear and chloroplast genome assembly and annotations of *A. fimbriata* have been deposited in NCBI under the BioProject accession number PRJNA656149. The genome assembly and annotations have also been deposited in the BIG Data Center (https://ngdc.cncb.ac.cn/) as a BioProject PRJCA004207 and CoGe. The *Amborella* genome assembly and annotations used in this study are available from CoGe (https://genomevolution.org/coge/GenomeInfo.pl?gid=50948). Source data are provided with this paper.

## Source data

Source Data Fig. 1 The LAI for each 3M sliding window on different chromosome with the step of 50 kb. It is the source data used for generating Fig. 1c.
Source Data Fig. 5 The mean size of floral-related gene families in the 22 studied species. It is the source data used for generating the barplot in Fig. 5a. The expression values (logarithm to base 2 of TPM plus 0.001) of floral-related genes in different tissues. The source data used for generating the heatmaps in Fig. 5d–f. The genes co-expressed with *AP3* and *PI* and other floral-related co-expressed gene pairs. It is the source data used for generating the co-expression network in Fig. 5g.
Source Data Fig. 6 The expression values (logarithm to base 2 of TPM plus 0.001) of TPS genes in different tissues. The source data used for generating the heatmaps in Fig. 6c.
Source Data Extended Data Fig. 3 The Nanopore read depth for each 50-kb sliding window on different chromosomes. It is the source data used for generating the Extended Data Fig. 3a. The resulted Nt.BspQI restriction site information of each BioNano optical moleculars. It is the source data used for generating the Extended Data Fig. 3c.
Source Data Extended Data Fig. 10 The relative expression values of seven NCSI genes in different tissues which were examined by qRT–PCR and further calculated using the comparative CT method. It is the source data used for generating the Extended Data Fig. 10.

## Code availability

The main custom scripts have been deposited in Github (https://github.com/yihenghu/Aristolochia_fimbriata_genome_analysis).
