# Supplementary material for: Insights into angiosperm evolution, floral development and chemical biosynthesis from the Aristolochia fimbriata genome
Source: Nat Plants. 2021 Sep 2;7(9):1239–53. doi: 10.1038/s41477-021-00990-2 (PMC8445822; doi:10.1038/s41477-021-00990-2)
Supplement: Supplementary file 1 — Supplementary Figs. 1.1–6.9, materials and methods, and detailed description of results and discussion. [file 41477_2021_990_MOESM1_ESM.pdf]

nature portfolio

https://doi.org/10.1038/s41477-021-00990-2

# Supplementary information

# Insights into angiosperm evolution, floral development and chemical biosynthesis from the *Aristolochia fimbriata* genome

In the format provided by the authors and unedited

# Supplementary Information

## Insights into angiosperm evolution, floral development and chemical biosynthesis from the *Aristolochia fimbriata* genome

Liuyu Qin[1,2#], Yiheng Hu[1,2#], Jinpeng Wang[1,2,3#], Xiaoliang Wang[1,2#], Ran Zhao[1#], Hongyan Shan[1], Kunpeng Li[1,2], Peng Xu[1,2], Hanying Wu[1], Xueqing Yan[1,2], Lumei Liu[1,2], Xin Yi[1], Stefan Wanke[4], John E. Bowers[5,6], James H. Leebens-Mack[5], Claude W. dePamphilis[7], Pamela S. Soltis[8], Douglas E. Soltis[8,9], Hongzhi Kong[1,2], Yuannian Jiao[1,2*]

[1]State Key Laboratory of Systematic and Evolutionary Botany, Institute of Botany, the Chinese Academy of Sciences, Beijing 100093, China.
[2]University of Chinese Academy of Sciences, Beijing 100049, China.
[3]School of Life Sciences, and Center for Genomics and Computational Biology, North China University of Science and Technology, Tangshan, Hebei 063000, China.
[4]Institute of Botany, Dresden University of Technology, Dresden 01062, Germany.
[5]Department of Plant Biology, University of Georgia, Athens, Georgia 30602, USA.
[6]Plant Genome Mapping Laboratory, University of Georgia, Athens, Georgia 30602, USA.
[7]Department of Biology and Huck Institutes of the Life Sciences, The Pennsylvania State University, University Park, Pennsylvania 16802, USA.
[8]Florida Museum of Natural History, University of Florida, Gainesville, Florida 32611, USA.
[9]Department of Biology, University of Florida, Gainesville, Florida 32611, USA.
[#]These authors contributed equally: Liuyu Qin, Yiheng Hu, Jinpeng Wang, Xiaoliang Wang, Ran Zhao.
*e-mail: jiaoyn@ibcas.ac.cn.

**This PDF file includes:**

Supplementary Notes
Supplementary Figs. 1.1-6.9
Supplementary References

**Addational Supplementary Files for this manuscript include the following:**

Supplementary Tables 1.1-6.7

**Supplementary Notes and Figures** ........................................................................3
1 Introduction to *Aristolochia* and the genome sequencing........................................3
1.1 Genome survey and genome size estimation.....................................................3
1.2 Oxford Nanopore Technologies (ONT), optical maps, and Hi-C sequencing .4
1.3 Nuclear and chloroplast genome assemblies ...................................................5
1.4 Genome quality assessments..........................................................................9
2 Genome annotation and gene family classification................................................10
2.1 Repeat annotation and TE analyses...............................................................10
2.2 Transcriptome sequencing by Illumina and PacBio Iso-Seq...........................11
2.3 Gene prediction and functional annotation.....................................................13
2.4 Gene family classification and comparison ....................................................14
3 Comparative genomics and whole-genome duplication (WGD) events in magnoliids..........................................................................................15
3.1 Comparative genomic analyses indicate no WGD in *A. fimbriata* since the origin of extant angiosperms ..................................................................15
3.2 Three rounds of WGDs detected in black pepper genome using the reference genome of *A. fimbriata* ..........................................................................19
3.3 Timing of the previously identified WGDs in magnoliids.............................22
3.4 Genome structure comparisons identified genomic rearrangement events shared by magnoliids and monocots ..........................................................26
4 Phylogenomic investigation of the relationship among magnoliids, monocots, and eudicots ...............................................................................................42
4.1 Phylogenomic analyses of strictly and mostly single-copy gene families......42
4.2 The impact of taxon sampling on the resulting phylogenetic topology..........53
4.3 Phylogenetic analysis of chloroplast genes....................................................55
4.4 Codon usage bias and the most plausible phylogenetic topology...................58
4.5 Molecular dating and gene family evolution ..................................................63
5 High-specialized flower morphology and floral development in *A. fimbriata*......65
5.1 Phylogeny of the MADS-box gene family .....................................................66
5.2 Alternative splicing of MADS-box genes in *A. fimbriata*..............................69
5.3 Expression patterns of floral organ identity genes .........................................70
5.4 Genetic basis of the floral organ fusion and the bilaterally symmetry of flowers..........................................................................................72
5.5 Floral color and trichome formation genes in *A. fimbriata*............................75
6 Metabolic gene clusters in *A. fimbriata* and the biosynthesis of terpenoid and aristolochic acids...........................................................................79
6.1 Identification of metabolic gene clusters in the *A. fimbriata* genome ............79
6.2 Floral scent and terpenes ................................................................................87
6.3 LC-MS-based metabolomic analysis and reconstruction of the aristolochic acid I biosynthesis pathway.......................................................................89
6.4 Identifying the key candidate genes based on phylogenetic classification and the sequence conservation of key residues ...............................................91
**Supplementary References**.............................................................................**100**

## 1 Introduction to *Aristolochia* and the genome sequencing

*Aristolochia*, a genus in the magnoliid order Piperales with approximately 450 species, has long been famous for its complicated flower morphology, specialized pollination, and enriched alkaloid chemistry[1-4]. The flowers of *Aristolochia* species consist of a monosymmetric, often tubular, dull purple-brown, and insect-trapping perianth, a gynostemium formed by the congenital fusion between stamens and the stigmatic region of the carpels, and elaborated inner epidermis (Fig. 1a and Extended Data Fig. 1)[5]. The flowers often emit unusual flower scent, which together with the peculiar flower morphology, enables virtually all *Aristolochia* species to exhibit deceptive pollination strategies that include attraction, imprisonment, and release of specific pollinators[3,6-8]. In addition, *Aristolochia* has been widely used in traditional pharmacopeias[9]. However, a class of nitrophenanthrene carboxylic acids called aristolochic acids (AAs) are naturally synthesized in *Aristolochia*; these compounds have been demonstrated to be highly nephrotoxic and carcinogenic to humans[10-13]. Therefore, many herbal medicines are banned or restricted due to their AA content.

*Aristolochia fimbriata* is a typical member of *Aristolochia*, it has special features that differ from closely related *Aristolochia* species including the development of fimbriae and papillae. *A. fimbriata* has two genotypes (VL and NV) which possess a number of readily discernible traits (e.g., leaf variegation, perianth shape and color)[5]. Importantly, as we proposed previously[5], because of its short life cycle, ease of large-scale cultivation, and small genome size, *A. fimbriata* could become a fantastic model system in magnoliids to facilitate comparative studies and functional investigations on the evolution of angiosperms and flowers. Therefore, here we selected *A. fimbriata* as the first *Aristolochia* species to decipher the nuclear genome sequence.

### 1.1 Genome survey and genome size estimation

#### Materials and Methods

The *A. fimbriata* (VL genotype) used for sequencing has been propagated via selfing for approximately 20 years in the greenhouse of Pennsylvania State University, USA. We raised new individuals from cuttings of a single plant in Pennsylvania State University, and cultivated them in the greenhouse of Institute of Botany, Chinese Academy of Sciences in Beijing. Young leaves from one individual were collected for flow cytometry and genome survey sequencing. For flow cytometry experiment, nuclei were released by chopping the young leaves and analyzed with the Moflo XDP Cell Sorter (Beckman-Coulter) according to the manufacturer's instructions. The genome size was then estimated by Summit software using *Arabidopsis thaliana* (125 Mb/2C) as a reference.

We also estimated the genome size of *A. fimbriata* using a *k*-mer-based approach. DNA was extracted from young leaves of *A. fimbriata* for the genome survey. Paired-end (PE) libraries with an insert size of 500 bp were constructed and sequenced using the Illumina platform (Supplementary Table 1.1). The *k*-mer distribution was then investigated using Jellyfish v2.2.10 with the parameters of '-t 10 -C -m 19 -s 4G'[14]. Genome size (G) was estimated by $G = k_{num}/k_{depth}$, where the $k_{num}$ represents the total number of *k*-mers, and the $k_{depth}$ denotes the *k*-mer depth of the peak frequency of *k*-mer distribution. We also used GenomeScope[15] to estimate the overall genome characteristics (heterozygosity rate, haploid genome length, and unique content length) from the Illumina data using Jellyfish v2.2.10[14] with the parameters of '-t 10 -C -m 19 -s 4G'.

**Results and Discussion**

The flow cytometry experiments found the mean peak fluorescence of *A. thaliana* (R1) at 26.99 and *A. fimbriata* (R2) at 62.51 (Extended Data Fig. 2a); the genome size of *A. fimbriata* was estimated to be approximately 289.50 Mb. We generated ~31 Gb of paired-end clean reads from the Illumina genome survey (Supplementary Table 1.1). Using the *k*-mer approach, we estimated the genome size as approximately 251-271 Mb when using different *k*-mer lengths from 17 to 31 (Supplementary Table 1.2). If we set the *k*-mer length to 17, we found the total number of *k*-mer = 20,853,344,487 and the *k*-mer depth = 83, and the estimated genome size as 251 Mb (Extended Data Fig. 2b and Supplementary Table 1.2). Therefore, based on estimations from the above two approaches, we infer that the genome size of *A. fimbriata* is about 251-290 Mb. In addition, the sequenced individual of *A. fimbriata* has extremely low heterozygosity (~0.07%) (Fig. 1d), following ~20 years of inbreeding in cultivation.

## 1.2 Oxford Nanopore Technologies (ONT), optical maps, and Hi-C sequencing

**Materials and Methods**

The same individual of *A. fimbriata* used for the flow cytometry and genome survey was used for the following three different types of sequencing. For ONT sequencing, DNA was extracted from young leaves using QIAGEN® Genomic Kits, and its quality was then assessed by 0.75% agarose gel and Nanodrop. Libraries with an insert size of 20-40 Kb were then prepared and sequenced on a GridION X5 instrument. For optical maps, DNA was extracted from young leaves according to a modified Bionano genomics protocol[16]. The long high-quality DNA was labeled by enzyme Nt.BspQI, and then loaded into the Saphyr chip for scanning. The output of the Bionano single-molecule map files in bnx format was subjected to an initial quality check to obtain high-quality molecule maps. To collect sufficient material for

Hi-C sequencing, we cultivated the seedlings by tissue culture using stem cuttings from the same individual used for the above sequencing. The samples were processed, and the DNA was extracted and crosslinked using the standard protocol. The Hi-C libraries were then amplified and sequenced with 150 bp paired-end reads using Illumina HiSeq.

## Results and Discussion

We achieved about 34 Gb of Nanopore long reads after filtering out the runs with the mean Q-scores less than 7, and the final length of the clean reads is about 120× coverage of the estimated genome size of *A. fimbriata* (Supplementary Table 1.3). The maximum length of the long reads is about 167 Kb, and the reads N50 and average length are 23.5 Kb and 16.6 Kb, respectively (Supplementary Fig. 1.1). For the Bionano optical maps, we obtained 404,934 molecules with minimum length of 180 Kb, and a total length of ~142 Gb (552× coverage of the genome) (Supplementary Table 1.4). For the Hi-C sequencing, there is 45 Gb valid data, about 180× coverage of the genome (Supplementary Table 1.5).

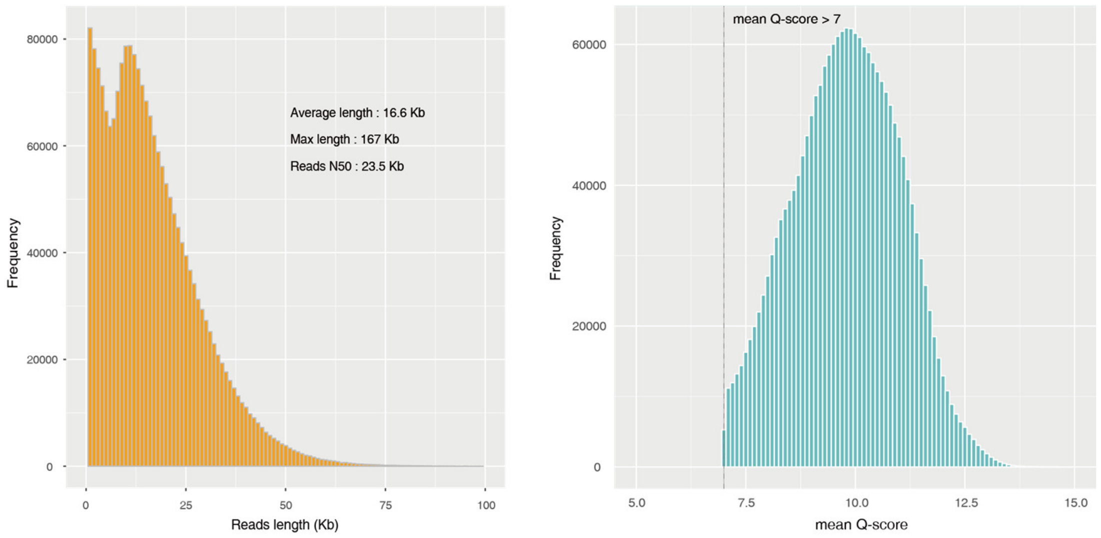


**Supplementary Fig. 1.1 | The frequency of ONT long reads length (left) and Q-score (right)**. The lowest mean Q-score value was setting to 7.

## 1.3 Nuclear and chloroplast genome assemblies

### Materials and Methods

**Nuclear genome *de novo* assembly.** The passed reads from GridION X5 runs were used to generate overlap maps using minimap2 v2.15-r914[17] with the parameter setting of '-x ava-ont'. The graphical fragment assembly format (GFA) file was then generated

by miniasm v0.3[18] with default parameters and was further converted into unitig sequences. We also performed three rounds of consensus correction using racon v1.3.3[19] based on minimap overlaps, and the consensus sequences were polished by Illumina reads using bwa-mem v0.7.12-r1039[20] and Pilon v1.22[21] with default parameters (Fig. 2b).

**Bionano optical maps and scaffold construction.** Low-quality optical molecules with length < 180 Kb or the molecule label number < 9 were removed first. The optical map assembly for *A. fimbriata* was generated using the Bionano Solve Pipeline v 3.3 with the parameter settings of '-i 0 -V 0 -A -z -u –m' (https://bionanogenomics.com/support/software-downloads/). The first assembly was reassembled by launching the settings of '-y -r (rough assembly cmap) -V 0 –m'. Hybrid scaffolds were generated by aligning the optical maps to ONT assembled genomic contigs using Bionano's hybrid-scaffold software (https://bionanogenomics.com/support/software-downloads/). Conflicting sites were further separated for both the contig sequences and optical maps, and the resulted maps and contigs were assembled again with the hybrid-scaffold parameters of '-B 2 -N 2'.

**Anchor scaffolds to pseudo-chromosomes using Hi-C.** Hybrid scaffolds with length > 100 Kb were used to construct pseudomolecules. A Hi-C contact map between genomic loci was computed using Juicer v1.7.6[22] with parameters of "-s MboI -r bwa mem" and visualized using Juicebox v1.8.8.8[23]. 3D-DNA v180114 was used to anchor and orient scaffolds[24] with following parameters: -m haploid -r 2. We also manually corrected the order or orientation of several misassembled scaffolds based on the Hi-C contact frequency and assembled the pseudo-chromosomes based on the Rabl configuration of the telomere-to-telomere contact enrichment using Juicebox Assembly Tools (JBAT version 1.8.8)[23].

**Chloroplast genome assembly.** To extract the reads from the chloroplast genome, we trimmed raw ONT reads and Illumina reads using Canu v1.8[25] and Trimmomatic v3.8[26] respectively, and then mapped them to the chloroplast genome of the *Aristolochia debilis* (downloaded from GenBank, accession NC_036153) using minimap2 v2.16-r922[17] and bowtie2 v2.3.4.1[27], respectively. These reads that mapped to the chloroplast genome were then extracted using samtools v1.9[28], and further *de novo* assembled with SPAdes v3.11.1[29]. The assembled scaffolds were aligned to the chloroplast genome of *A. debilis* using Geneious v8.0.2 to determine the order of the scaffolds[30], and MITObim v1.9 was used to fill gaps between the scaffolds based on the Illumina short reads[31]. Lastly, the final circular chloroplast genome was plotted using OGDRAW[32].

## Results and Discussion

We assembled the 120× ONT clean long reads into 378 contigs (contig N50 = 5.16 Mb), which have better contiguity than the other published magnoliid genomes (Fig. 2b and Supplementary Tables 1.6 and 1.7). The consensus genomic sequences of the contigs were then corrected using 100× Illumina reads and further used to assemble scaffolds by integrating the 351× valid optical map data, with 89.3% of the total length of the contig sequences and 97.4% of the genome maps incorporated (Supplementary Fig. 1.2 and Supplementary Table 1.8). We generated a hybrid assembly of 258 Mb of sequence in 283 scaffolds with an N50 of 12.89 Mb (Supplementary Table 1.6). The scaffolds were further clustered and ordered using Hi-C and assembled into 7 pseudo-chromosomes that covered ~95% of the assembled genome (Supplementary Fig. 1.3).

We also assembled the complete chloroplast genome of *A. fimbriata*, with length of 160,529 bp and an average GC content of 38.5%. This assembled circular molecule has a typical quadripartite structure containing a large single-copy region of 90,080 bp, an inverted region A (IRa) of 25,433 bp, a short single-copy region of 19,583 bp, and an inverted region B (IRb) of 25,433 bp. In total, 131 genes were annotated, including 86 protein-coding genes, 37 transfer RNA (tRNA), and 8 ribosomal RNA (rRNA) genes (Supplementary Fig. 1.4).

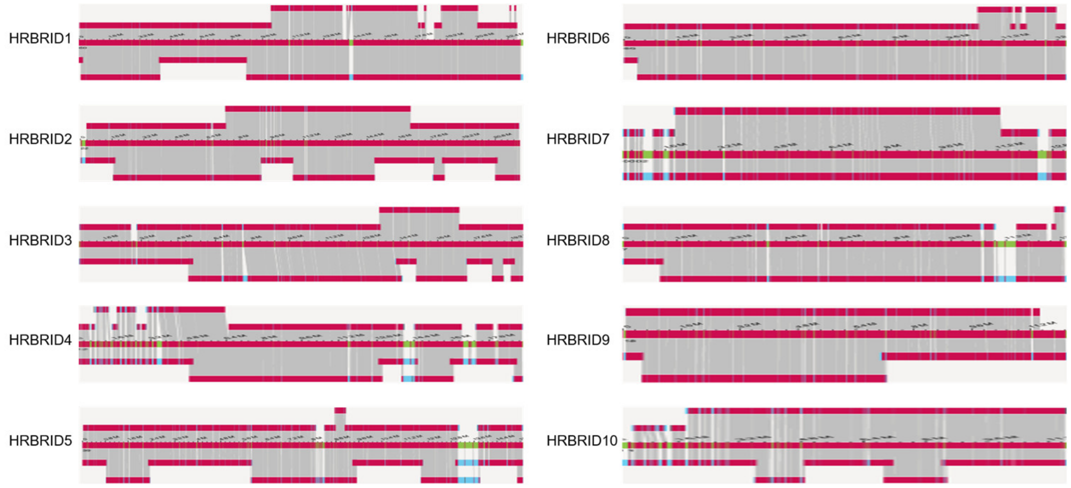


**Supplementary Fig. 1.2 | Alignments of the *A. fimbriata* Bionano optical maps against in-silico maps of the largest 10 hybrid scaffolds.** Alignment of hybrid scaffolds showed ONT contigs ordered and oriented after mapping to the Bionano optical maps. The ONT contigs are on the top track; the hybrid scaffolds are in the middle track; Bionano maps are on the bottom track. Collinear Nt.BspQI markers on the two maps are linked with gray lines. The green blocks mark the gap regions in the hybrid scaffolds.

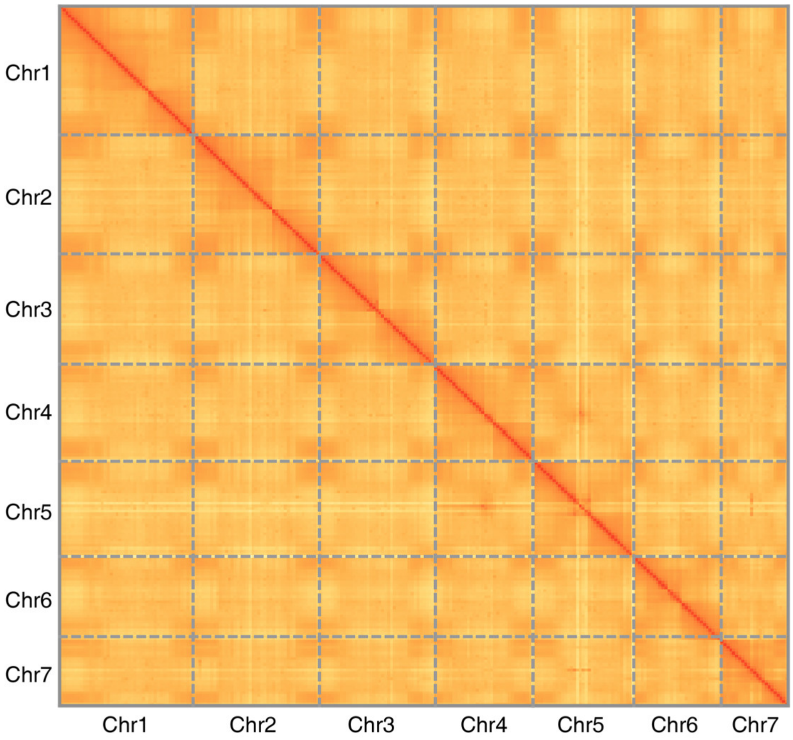


**Supplementary Fig. 1.3 | Hi-C contact matrices of the seven pseudo-chromosomes of the final *A. fimbriata* assembly.**

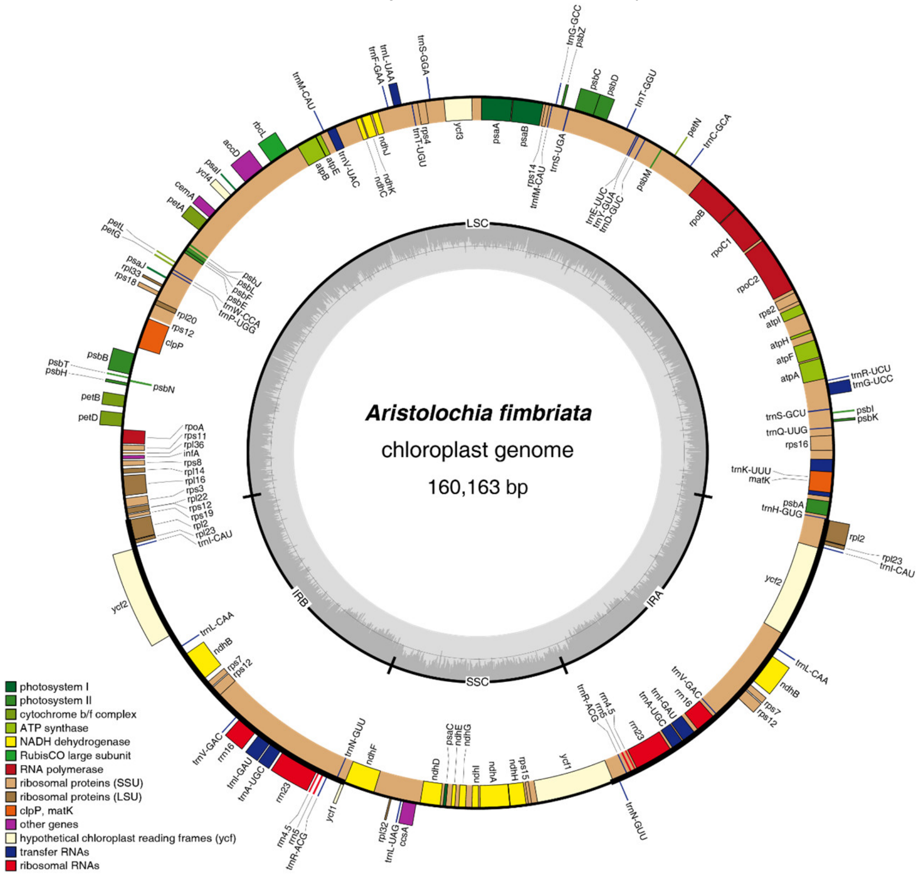


**Supplementary Fig. 1.4 | The assembled circular chloroplast genome of *A. fimbriata*.** In total, 131 genes were annotated, including 86 protein-coding genes, 37 transfer RNA (tRNA), and 8 ribosomal RNA (rRNA) genes.

## 1.4 Genome quality assessments

### Materials and Methods

The quality and completeness of the *A. fimbriata* genome assembly were assessed from four aspects. First, we evaluated the mapping rates of the clean raw reads from transcriptomes and genomic DNA by TopHat2[33] and BWA-MEM[20] with default parameters, respectively. We further used the "--vcf" option in Pilon v1.23[21] to call SNPs from the Illumina genomic reads with parameters of "--diploid --fix all --mindepth 0.1". Second, we investigated the Benchmarking Universal Single Copy Orthologs (BUSCO) genes from Embryophyta in the final assembly[34]. Third, we employed the LTR Assembly Index (LAI) to infer the assembly continuity[35] with parameters of "-step 50000 -t 8". Finally, we aligned Bionano molecules back to the final *A. fimbriata* genome assembly to check the consistency between Bionano molecules and the final genome assembly using the RefAligner tool (https://bionanogenomics.com/support/software-downloads/) with default parameters. In addition, we also checked the consistency of the Bionano assembly consensus genome maps (CMAP) and the in-silico maps of the *A. fimbriata* genome assembly.

### Results and Discussion

First, the average overall mapping rate of the nine transcriptomes was about 93.2%, and the average rate of the properly mapped reads was approximately 89% (Supplementary Table 1.9). The overall mapping rate of the 100× Illumina PE reads was 99.77%, and the rate of properly mapped reads was 95.96% (Supplementary Table 1.10). Very low heterozygosity (~0.07%) was identified based on the SNPs calling (Fig. 1d). The median depth of coverage of the ONT clean raw reads was about 80×, and only limited gaps were observed across the seven assembled pseudo-chromosomes (Extended Data Fig. 3a). Second, we identified 96.8% of the Plantae BUSCO genes in the assembled *A. fimbriata* genome; the other published magnoliid genomes identified 86.3% to 96.1% of the BUSCO genes (Supplementary Tables 1.11 and 1.12). Third, the LAI of the *A. fimbriata* assembly is about 21, similar to those of well assembled *Oryza sativa* (MSU7) and *Zea mays* (B73v4) genomes (Extended Data Fig. 3b,d), suggestive of high-quality assembly (Fig. 1c)[35]. Finally, we observed that the Bionano molecules uniformly and consistently mapped to the assembled genome (Extended Data Fig. 3c). Moreover, 97.5% of the Bionano consensus genome maps (CMAP) were uniquely aligned to the seven in-silico maps of the *A. fimbriata* reference assembly (Supplementary Table 1.13). We also successfully assembled the telomeric tandem repeat arrays (CCCTAAA/TTTAGGG repeats) at both distal ends of chromosomes 2, 3, 5, and 6, and at one distal end of chromosomes 1 and 7 (Supplementary Table 1.14).

## 2 Genome annotation and gene family classification

### 2.1 Repeat annotation and TE analyses

**Materials and Methods**

The repetitive sequences were identified using a combination of repeat similarity searching and *ab initio* prediction approaches. Repbase v20.05[36] was employed to search against the *A. fimbriata* genome using RepeatMasker v4.0.7[37] with default parameters. For *ab initio* prediction, a consensus sequence library was built using RepeatModeler v1.0.10 (http://repeatmasker.org/RepeatModeler/) with the parameter '-engine ncbi'. Then, LTRharvest v1.5.10[38], LTR_FINDER v1.05[39], and LTR_retriever v1.8.0[40] were used to build an LTR library with default parameters. These two libraries were used to annotate the *A. fimbriata* genome using RepeatMasker, and the detected transposable elements (TEs) were then combined to obtain the final TE annotation. We also downloaded the genomic sequences of *Amborella trichopoda*, four other magnoliids (*Piper nigrum*, *Cinnamomum kanehirae*, *Liriodendron chinense*, *Persea americana*), two eudicots (*Aquilegia coerulea*, *A. thaliana*), and one monocot (*O. sativa*) (Supplementary Table 2.1), and performed TE annotation for these genomes based on the same processes for comparison.

**Results and Discussion**

We found that 52.1% of the *A. fimbriata* genome sequence is composed of transposons (Supplementary Table 2.2). Long terminal repeat (LTR) retrotransposons are the most abundant transposable elements (Supplementary Fig. 2.1a), of which *Ty3*/*Gypsy*- and *Ty1*/*Copia-like* DNA sequences account for 21.3% and 4.6% of the genome assembly, respectively (Supplementary Table 2.2). The fractions of different types of TEs in the *A. fimbriata* genome are similar to those of other published magnoliid genomes and *A. trichopoda* (Supplementary Table 2.3). We plotted the density of the main TE types along each chromosome (Fig. 1d). The identified *Ty3*/*Gypsy* LTRs are clustered around the centromeric region of all chromosomes, while the LINE/L1 TEs tend to be located outside of the centromeric regions (Fig. 1d). Notably, the highest percent of LINE/L1 TEs occur in the introns of genic regions in *A. fimbriata*, compared to several other genomes, and genes with LINE/L1 TEs insertions tend to have relatively high expression levels (Supplementary Fig. 2.1). Furthermore, we also found that DNA transposon types *MULE-MuDR* and *CMC-EnSpm* are localized to centromeric regions but absent from the rest of the genome (Fig. 1d).

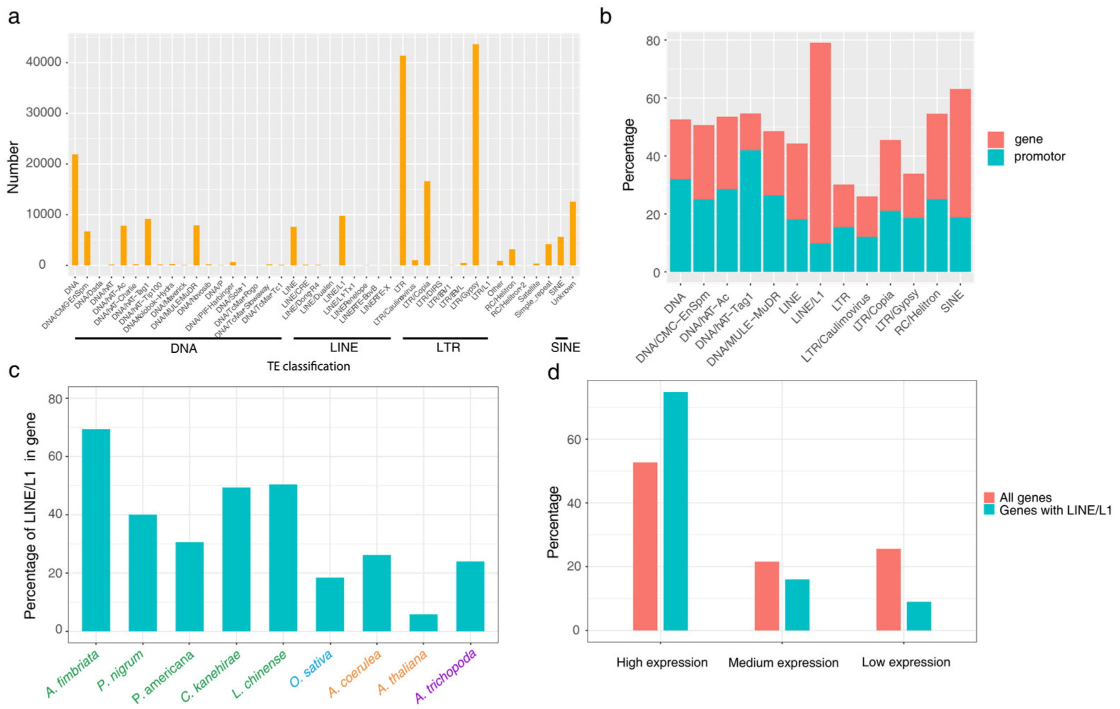


**Supplementary Fig. 2.1 | TE annotation in the genome of *A. fimbriata*.** (a) Number of different types of TEs identified in the *A. fimbriata* genome. (b) Percentage of different types of TEs located in gene or promoter regions. (c) Comparison of the percentage of LINE/L1 TEs located in gene regions of the *A. fimbriata* and eight other angiosperm genomes. (d) Expression levels of genes with LINE/L1 insertions in the intron regions. High expression: TPM⩾10; Medium expression: 1⩽TPM<10; Low expression: TPM <1.

## 2.2 Transcriptome sequencing by Illumina and PacBio Iso-Seq

### Materials and Methods

**Illumina RNA-Seq data.** Total RNAs were separately extracted and processed from leaves, seedlings, and five different floral organs (limb, tube, utricle, gynostemium, and ovary) using Trizol reagent (Invitrogen, CA, USA) following the manufacture's procedure. The paired-end cDNA libraries with insert size of 150 bp were constructed and sequenced using Illumina HiSeq4000 instrument. Low-quality reads were filtered out using Trimmomatic[26] with the parameters of 'PE -phred33 ILLUMINACLIP: Trimmomatic-0.36/adapters/TruSeq3-PE.fa: 2:30:10 LEADING: 20 TRAILING: 20 SLIDINGWINDOW: 4:20 MINLEN: 50'.

**PacBio Iso-Seq data.** We sequenced a library of full-length transcripts using PacBio Iso-Seq. Samples from anthetic flowers, seedlings under normal growth conditions, seedlings treated with low temperature (4°C) for 9 hours, and roots were collected,

and the extracted RNAs from the four samples were mixed together in equal amount to obtain transcriptomes from various plant tissues and treatments. The cDNA libraries were constructed using the SMARTer™ PCR cDNA Synthesis Kit. The full-length cDNA fragments were screened using a BluePippin instrument to construct cDNA libraries of different sizes (1-2 Kb; 2-3 Kb; and 3-6 Kb) (Supplementary Fig. 2.2). The libraries were sequenced on a PacBio RS II instrument, and the raw reads were processed using SMRT Link 5.0 software. First, the circular consensus sequences (CCSs) were generated from the subreads BAM files with parameters of "--minLength=300 --minPasses=1 minPredictedAccuracy=0.8". Next, all the CCSs were further classified into full-length non-chimeric (FLNC) and non-full-length (nFL) transcript sequences based on whether the 5'-primers, 3'-primers, and poly(A) tail could be detected. To improve consensus accuracy, we clustered and polished the FL sequences using an isoform-level clustering algorithm, iterative clustering for error correction (ICE), and the Quiver tool in the SRMT Link software. The FL reads were further corrected using RNA-Seq reads using LoRDEC[41] with the parameters of '-k 19 -s 3 -T 4', and redundancy was removed using Cd-hit[42] with the parameters of '-c 0.99 -T 10 -G 0 -aL 0 -aS 0.99 -AS 30 -d 0 -p 1'.

## Results and Discussion

For Illumina RNA-Seq, we obtained approximately 6 Gb clean data on average for each sample (Supplementary Table 2.4). For PacBio Iso-Seq, there were 4,418,800 raw subreads in total from the three libraries of different sizes, and we obtained 46,287 FLNC transcripts after multi-step processing (Supplementary Fig. 2.2). These transcriptome data were used for genome annotation.

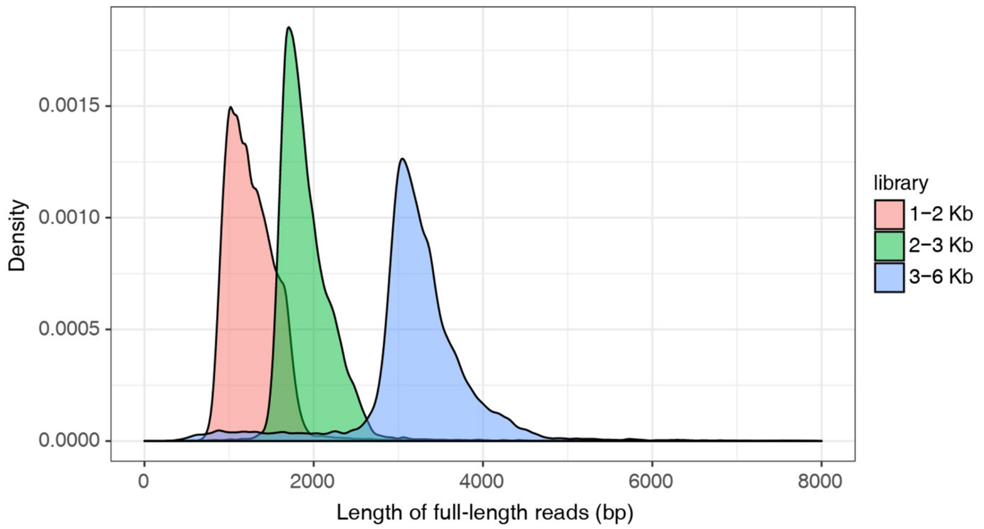


**Supplementary Fig. 2.2 | The length distribution of the full-length non-chimeric transcripts of PacBio Iso-Seq.** The output results are consistent with the three constructed cDNA libraries.

## 2.3 Gene prediction and functional annotation

### Materials and Methods

The protein-coding genes were predicted using the well-developed combination strategies of transcriptome, homology-based annotation, and *ab initio* gene prediction. For the *ab initio* prediction, the repeat-masked scaffolds were annotated using Fgenesh[43] and AUGUSTUS[44] with default parameters. For the homology-based prediction, we used the inferred amino acid sequences from the *A. coerulea, Ananas comosus, A. thaliana, A. trichopoda, Papaver somniferum,* and *C. kanehirae* genomes. GeneWise[45] and GeMoMa[46] were used to annotate the gene models using alignments from amino acid sequence similarity against the *A. fimbriata* assembled sequences. For transcriptome-based prediction, PASA[47] and GMAP[48] were used to predict the gene models. If the transposable domain occupied more than 60% of the predicted gene length, the gene was removed using TransposonPSI (http://transposonpsi.sourceforge.net). Finally, the results from the three approaches were integrated to generate EVidenceModeler (EVM)[49] gene models to obtain the final annotated protein-coding gene set.

The putative functions of the genes were predicted by searching the best matched proteins in SwissProt (https://web.expasy.org/docs/swiss-prot_guideline.html), non-redundant (Nr) (https://ftp.ncbi.nlm.nih.gov/blast/db/FASTA/) and Eukaryotic Orthologous Groups (KOG) (https://hsls.pitt.edu/obrc/index.php?page=URL1144075392) databases using BLASTP ($E$-value$\leqslant 10^{-6}$). Gene Ontology (GO) terms were also assigned to the genes by combining the results from Blast2GO v5.2.5[50] and eggNOG-mapper v22[51] annotations. We also used the KEGG database (https://www.genome.jp/kegg/) to obtain KEGG orthologs to infer putative gene pathways.

### Results and Discussion

For the *ab initio* gene annotation, we predicted 24,596 genes using AUGUSTUS[44], and 21,774 genes using Fgenesh[43]. For the protein similarity-based prediction, we obtained 14,868 to 18,677 genes using GeMoMa[46] and 17,504 genes using GeneWise[45]. For transcriptome-based predictions, we obtained 11,787 genes from the Illumina RNA-Seq and 12,091 genes from PacBio Iso-Seq. We combined these results together, and annotated 21,751 genes in total for the *A. fimbriata* genome, 19,582 of which were classified as high-confidence genes based on whether they have support from the aforementioned transcriptomes and whether they exhibit overlapping with TEs. The annotated genes were further compared among *A. fimbriata, A. trichopoda,* and representative genomes of magnoliids, eudicots, and monocots (Supplementary Table 2.6). In addition, we were able to functionally annotate 17,966

(82.6%) genes using the databases of SwissProt, KOG, KEGG, GO, and Nr. The remaining 3,785 genes were treated as “unknown function” (Supplementary Table 2.7).

### 2.4 Gene family classification and comparison

**Materials and Methods**

We selected 22 species to construct putative gene families, including five monocots (*O. sativa*, *Sorghum bicolor*, *Musa acuminata*, *Spirodela polyrhiza*, and *Phalaenopsis equestris*), seven eudicots (*Vitis vinifera*, *A. thaliana*, *Populus trichocarpa*, *Solanum lycopersicum*, *P. somniferum*, *A. coerulea*, and *Nelumbo nucifera*), five magnoliids (*L. chinense*, *C. kanehirae*, *P. americana*, *P. nigrum*, and *A. fimbriata*), two representatives of the ANA grade (*A. trichopoda* and *Nymphaea colorata*), two gymnosperms (*Ginkgo biloba* and *Picea abies*), and one lycophyte (*Selaginella moellendorffii*) (Supplementary Table 2.8). The longest transcript isoform for each locus was selected for all-vs-all BLASTP[52] with an *E*-value cut-off setting of $10^{-5}$. OrthoMCL v2.0.9[53] was used to identify gene clusters of putative gene families, and the inflation parameter was set to 1.5 in the mcl process[54]. The output from OrthoMCL was summarized using a custom Python script to obtain the number of genes from each species belonging to the orthogroups (OGs). Venn diagrams of the selected taxa were generated using InteractiVenn (http://www.interactivenn.net/).

**Results and Discussion**

In total, 577,294 genes (77.1% of the total sequences) from the selected 22 species were clustered into 51,315 OGs (Supplementary Table 2.9). 3,327 OGs contained genes from all 22 species, and 7,051 OGs were magnoliid-specific (Supplementary Table 2.10). For the annotated 21,751 genes in *A. fimbriata*, 17,995 were clustered into 12,054 OGs, 335 of which contained only *A. fimbriata* genes and could be species-specific (Supplementary Tables 2.9 and 2.10). The numbers of gene families shared among different clades of angiosperms, and among representative species, are shown in the Venn diagrams (Supplementary Fig. 2.3).

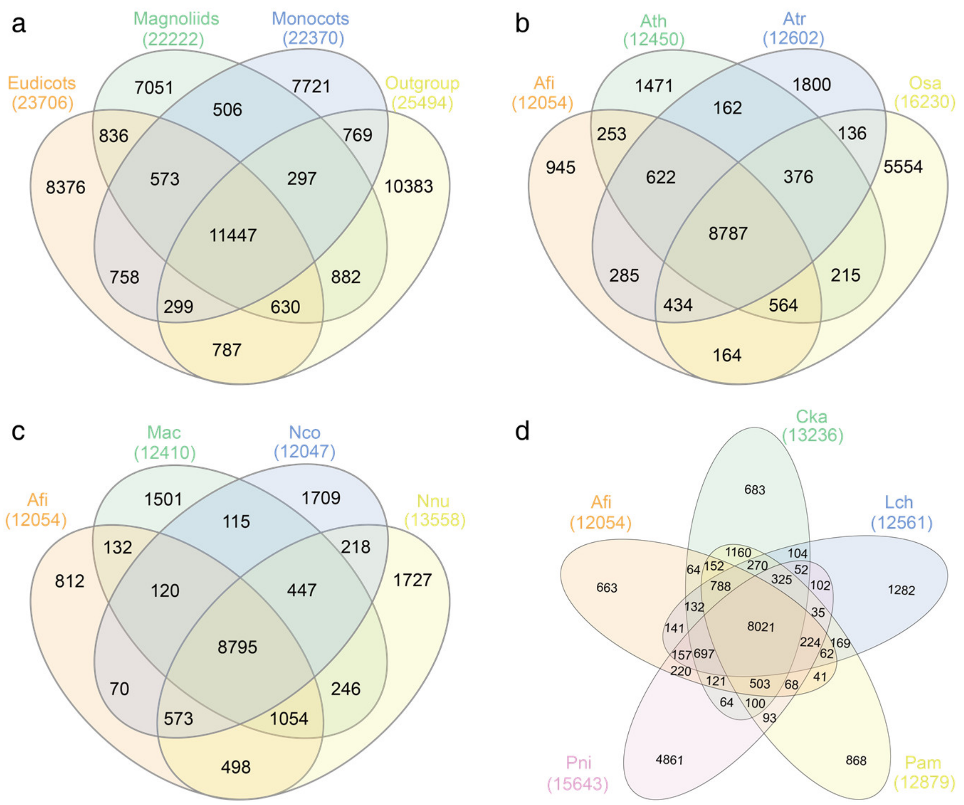


**Supplementary Fig. 2.3 | Venn diagrams showing the numbers of shared and species/clade specific OGs in the 22 selected species.** (a) The common and clade-specific OGs among magnoliids, eudicots, monocots, and outgroups (two gymnosperms and a lycophyte). (b) Venn diagram of OGs shared by *A. fimbriata* (Afi), *A. thaliana* (Ath), *O. sativa* (Osa), and *A. trichopoda* (Atr). (c) Venn diagram of OGs shared by *A. fimbriata* (Afi), *N. nucifera* (Nnu), *M. acuminata* (Mac), and *N. colorata* (Nco). (d) Venn diagram of OGs shared by the five magnoliid species, Afi, *P. nigrum* (Pni), *P. americana* (Pam), *L. chinense* (Lch), and *C. kanehirae* (Cka). Numbers in parentheses are the total number of OGs in that species or clade; numbers in the intersections show the number of OGs shared by the species or clade.

## 3 Comparative genomics and whole-genome duplication (WGD) events in magnoliids

### 3.1 Comparative genomic analyses indicate no WGD in *A. fimbriata* since the origin of extant angiosperms

#### Materials and Methods

Inter- and intragenomic comparisons were conducted to infer the WGD history in *A. fimbriata*. *Amborella*, sister to all other extant angiosperms[55-58], and water lily (*N. colorata* of Nymphaeales, the subsequent sister to all remaining angiosperms)[59] were

used as references to infer if any additional WGD events had occurred in the evolutionary history of *A. fimbriata*. It has been widely accepted that no additional WGDs occurred in the *Amborella* lineage after its split from the rest of extant angiosperms[55]. The *N. colorata* genome study revealed a recent WGD after the origin of angiosperms[59]. Both the *Amborella* and *Nymphaea* genomes, with their clear WGD history, can serve as important references to infer the evolutionary history of *Aristolochia*.

For the genome structural comparisons, we investigated the intra- and intergenomic syntenic blocks that were then shown in dotplots. First, we performed all-against-all BALSTP (*E*-value < $10^{-5}$, and Score > 100)[52] within and between genomes. Then, the top ten BLAST matches are selected for inferring syntenic blocks within or between genomes. We employed MCScanX[60] to identify syntenic blocks by setting the maximum gap between the anchor genes to 25, as commonly implemented in other studies[61,62]. We further plotted the syntenic gene pairs according to their genomic locations in dotplots, and used different color-coded dots to distinguished whether the anchor gene pairs are the best BLAST hit within/among the genomes. Finally, we inferred the WGD history by investigating the syntenic depth ratios within and among genomes.

The median synonymous substitution rate (*Ks*) values of syntenic anchor gene pairs were further employed to determine the divergence degree of the identified syntenic blocks. First, *Ks* was estimated using the Nei–Gojobori approach[63] implemented in the Bioperl statistical module. Then, we adopted a kernel function analysis to obtain the *Ks* distribution, which was then simulated as a mixture of multiple normal distributions by the kernel smoothing density function (*Ks* density, width was set to 0.05). Lastly, we performed the Gaussian multi-peak fitting of the curve by using the gaussian approximation function Gaussian in the fitting toolbox cftool. We set the R-squared above 95% which is a parameter to evaluate the fitting level. The smallest number of normal distributions was used to represent the multiple peaks of the *Ks* distribution.

**Results and Discussion**

Intragenomic comparison of *A. fimbriata* identified very sparse and weak syntenic blocks (Supplementary Fig. 3.1), which were potentially retained from the previously identified WGD characterizing all living angiosperms[64]. In the comparison of the *A. fimbriata* and *Amborella* genomes, we identified 450 microsyntenic blocks comprising 6,378 anchor genes in each genome, in which 207 and 10 syntenic blocks have more than 10 and 50 anchor gene pairs, respectively (Supplementary Fig. 3.2a and Supplementary Table 3.1). The longest syntenic block, which is between *A. fimbriata* chromosome 3 and *Amborella* chromosome 4, contains 77 syntenic anchor

gene pairs. We found that each *Amborella* genomic region only matched a single region in *A. fimbriata* (Supplementary Fig. 3.2a). The results clearly showed that the syntenic depth ratio between *Amborella* and *A. fimbriata* is 1:1, indicating that, as in *Amborella*, no independent WGD has occurred in *A. fimbriata* since the shared WGD that occurred in the common ancestor of all extant angiosperms. Moreover, intergenomic comparison between *A. fimbriata* and *N. colorata* revealed that their corresponding syntenic depth ratio is 1:2 (Supplementary Fig. 3.2b), which is consistent with an independent WGD having occurred in the *N. colorata* lineage[59]. Additionally, only 3/567 syntenic regions with more than 50 anchored gene pairs were detected (Supplementary Table 3.1), which again suggests extensive chromosomal rearrangements following each round of WGD[65]. Taking one microsyntenic comparison as an example, we identified the region of Chr1:6.61-7.12 Mb in *A. fimbriata* matching with two genomic regions, Chr8:32.66-33.38 Mb and Chr10:7.79-8.76 Mb, in *N. colorata*, and one region, Chr4:52.21-48.06 Mb, in *Amborella* (Supplementary Fig. 3.3). Therefore, our results clearly indicate that no WGD occurred in the history of *A. fimbriata* since its shared ancestry with *Amborella*. Importantly, the high degree of conservation between *A. fimbriata* and *Amborella* enables the construction of the ancestral angiosperm genome and also indicates that the *A. fimbriata* genome would be an exceptional evolutionary reference genome for flowering plants.

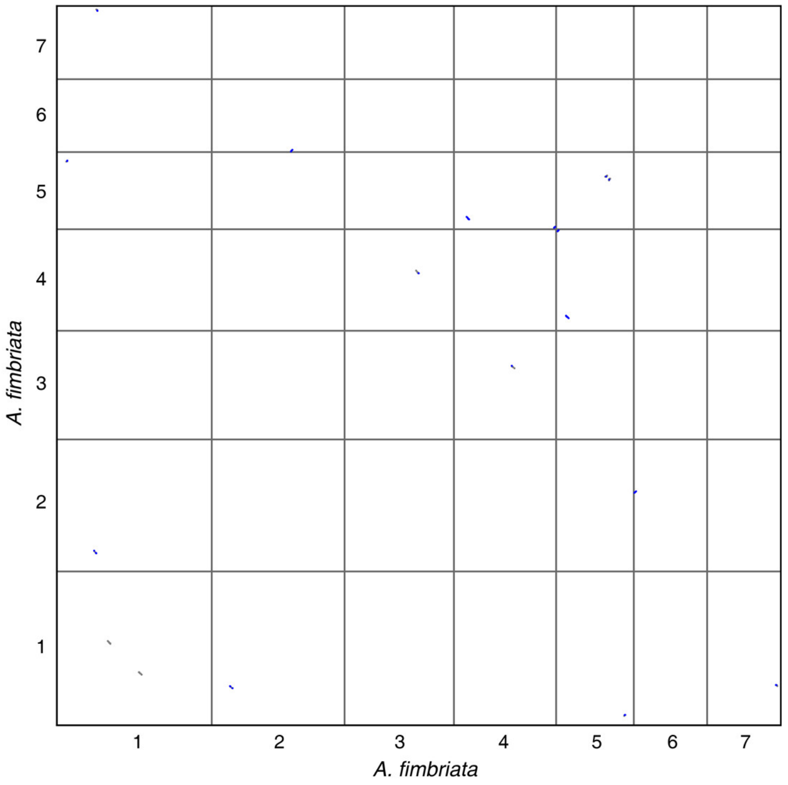


**Supplementary Fig. 3.1 | Synteny of the self-genomic comparison in *A. fimbriata*.** Genomic syntenic blocks (⩾5 anchor gene pairs) inferred from MCScanX were shown in dotplot according to their genomic locations in *A. fimbriata*.

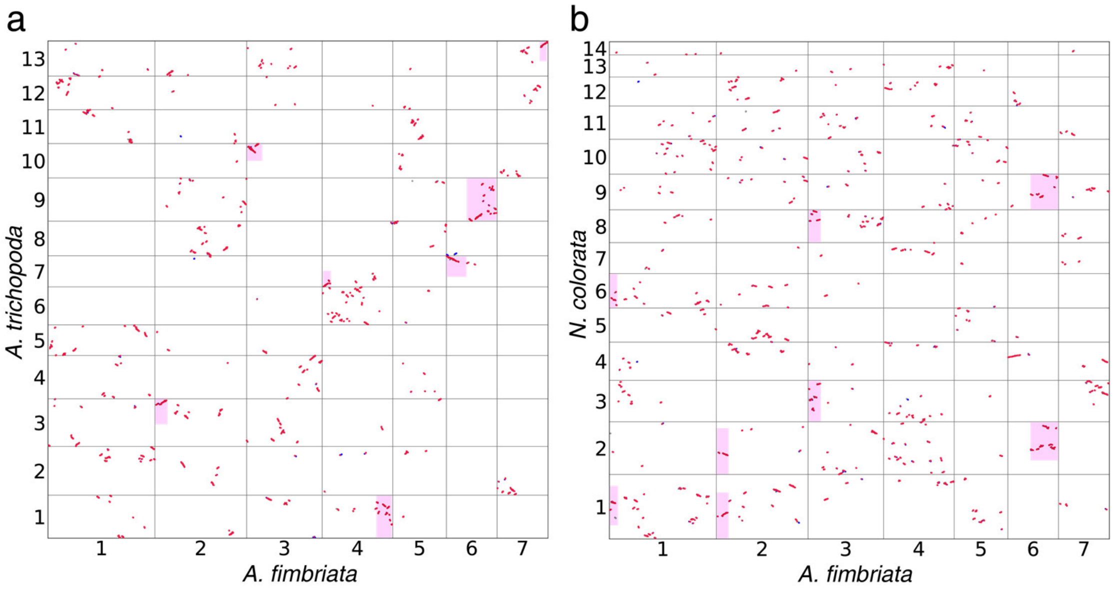


**Supplementary Fig. 3.2 | Intergenomic comparative analyses.** (a) Syntenic dotplot between the *A. fimbriata* and *A. trichopoda* genomes. (b) Syntenic dotplot between the *A. fimbriata* and *N. colorata* genomes. Genomic synteny blocks (⩾5 anchor gene pairs) were shown in dotplots according to their genomic locations in *A. fimbriata*, *A. trichopoda*, and *N. colorata*. If the anchor gene pairs are the best BLAST hit among the genomes, they are plotted as red dots; otherwise, they are shown in blue dots. Highlighted purple boxes indicate the selected orthologous regions between *A. fimbriata* and *A. trichopoda* with syntenic depth ratio of 1:1, and the syntenic regions between *A. fimbriata* and *N. colorata* with syntenic depth ratio of 1:2.

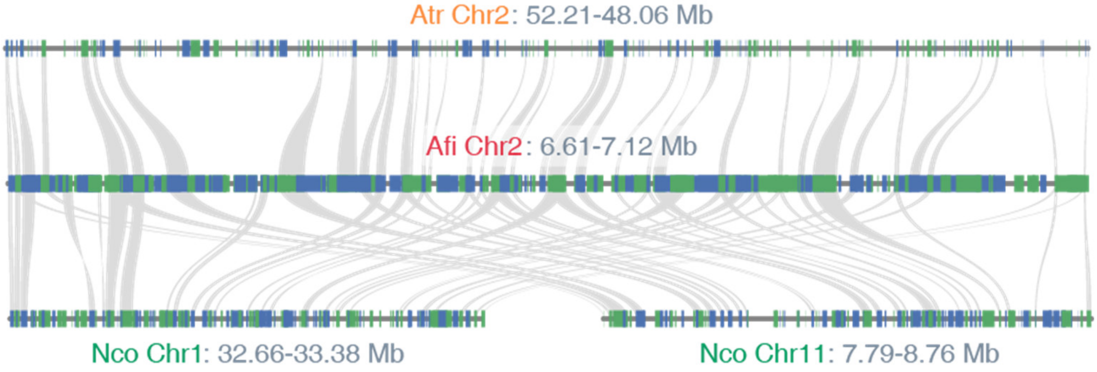


**Supplementary Fig. 3.3 | Representative microsynteny among the genomes of *A. trichopoda* (Atr), *A. fimbriata* (Afi), and *N. colorata* (Nco).** Rectangles represent predicted genes with orientation on the same strand (blue) or reverse strand (green). Gray lines link gene pairs with syntenic relationship. The syntenic depth ratio is clearly shown as 1 (Atr) : 1 (Afi) : 2 (Nco).

## 3.2 Three rounds of WGDs detected in the black pepper genome using the reference genome of *A. fimbriata*

### Materials and Methods

The high-quality genome assembly of *A. fimbriata*, lacking any additional WGD since the origin of extant angiosperms, is an excellent reference for comparative genomics in magnoliids, and for all angiosperms more generally. Using the *A. fimbriata* genome as a reference, we compared the genome of black pepper (*P. nigrum*) using MCScanX[60] with the same parameters described in methods Supplementary Note 3.1 and investigated the syntenic depth ratio to infer the extent of WGD in *P. nigrum*. For black pepper syntenic blocks, we also calculated the *Ks* values to identify their kernel density.

### Results and Discussion

Comparing the genomes of *A. fimbriata* and *P. nigrum*, we revealed well-preserved intergenomic homologous regions, with one *A. fimbriata* chromosomal region matching eight homologous regions in the black pepper genome (Fig. 2b,c and Supplementary Fig. 3.4). In total, 1,399 intergenomic syntenic regions were identified (107/1,399 syntenic blocks with >20 syntenic gene pairs), which contain 15,365 anchor gene pairs with 6,756 *A. fimbriata* genes and 14,080 black pepper genes. We further performed syntenic analysis of the self-comparison for black pepper (Supplementary Fig. 3.5), and estimated the median *Ks* values for the paralogous syntenic blocks. Three different duplication periods were identified with *Ks* around 0.11, 0.69, and 0.91, respectively (Supplementary Fig. 3.6). Our results suggested that three independent polyploidization events (Pn-α, Pn-β, and Pn-γ) occurred in the evolutionary history of black pepper, and the more ancient two events (Pn-β and Pn-γ) seem to have been overlooked in a previous study[66].

To further classify the syntenic blocks into three events, we carefully investigated the density and sequence similarity of the inter- and intragenomic synteny. For example, the *A. fimbriata* chromosome 6 matched to eight orthologous regions in the *P. nigrum* genome that could be further classified into four groups based on the block length and their median *Ks* values (Fig. 2b and Supplementary Fig. 3.4). The two regions in group_A (Pn1p-Pn13, *Ks* ~0.11, colored blue), group_B (Pn3-Pn15, *Ks* ~0.15, colored orange), group_C (Pn1q-Pn21, *Ks*~0.14, colored yellow), and group_D (Pn19-Pn20, *Ks* ~0.15, colored green) were duplicated from the most recent WGD Pn-α (Fig. 2b), and they also show great synteny length and density (Supplementary Fig. 3.5). The median *Ks* value of collinear genes between the group_A and group_B, and between the group_C and group_D are 0.71 and 0.64, suggesting they were duplicated from the Pn-β event. The *Ks* value between groups_A/B and groups_C/D is about 0.89,

indicating that they were duplicated from the most ancient Pn-γ event (Supplementary Fig. 3.6). Therefore, we found that three rounds of WGDs differentiate black pepper from *A. fimbriata* (and from the common ancestor of all extant angiosperms) instead of one WGD reported for black pepper previously[66]. Three WGDs in black pepper are also consistent with its high chromosome number of $2n = 52$, which is $4x$ based on a base chromosome number for *Piper* of $x = 13$[67], a number which itself can now be inferred to be polyploid.

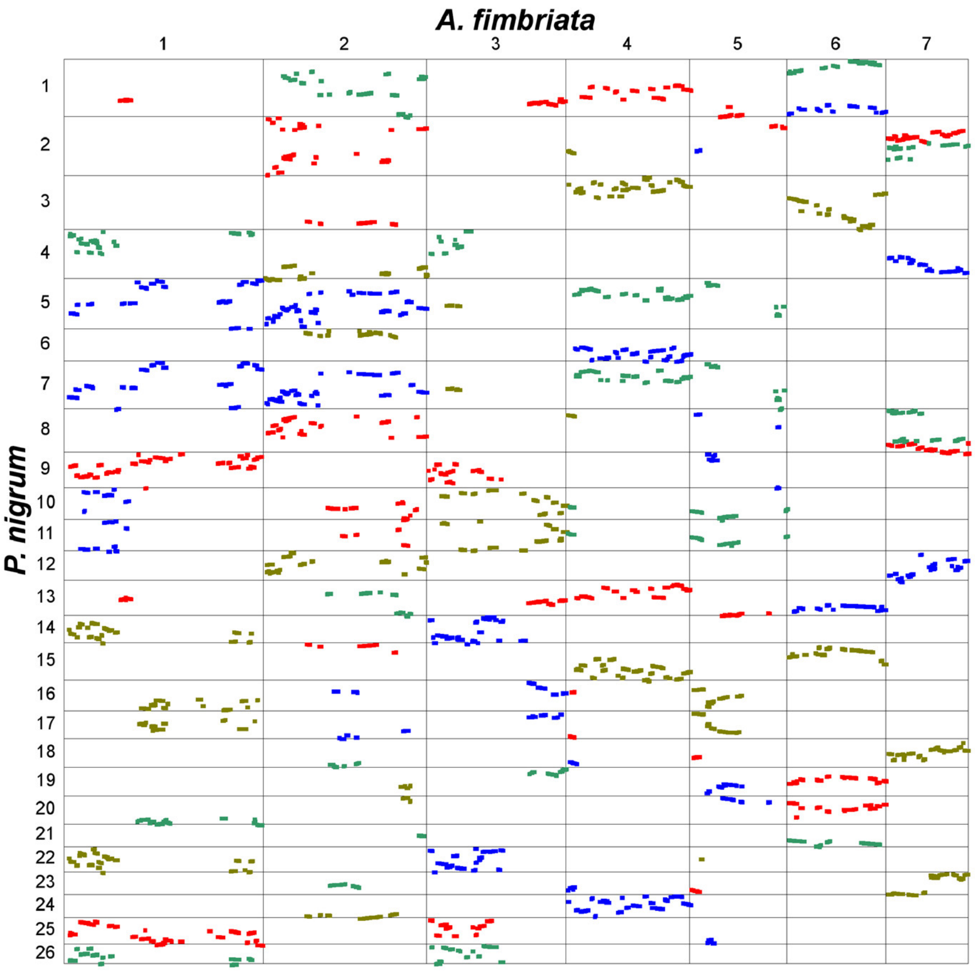


**Supplementary Fig. 3.4 | Syntenic blocks between *A. fimbriata* and *P. nigrum* genomes.** Dotplot shows the orthologous regions detected by MCScanX, and the same color-coded regions denote duplicated regions from Pn-α in *P. nigrum*.

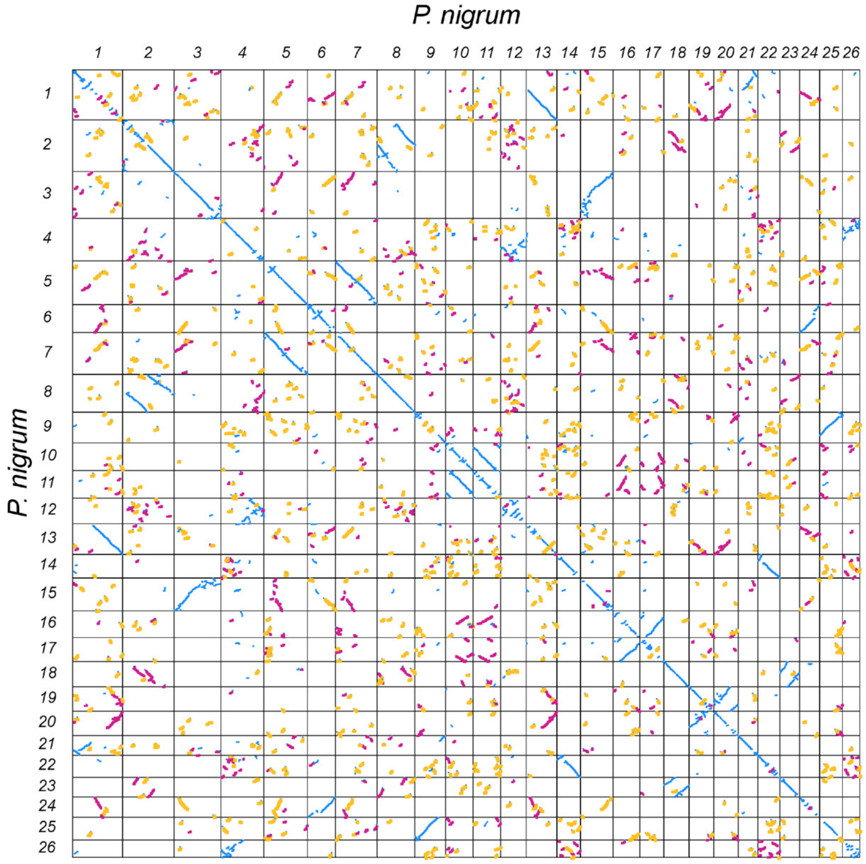


**Supplementary Fig. 3.5 | Intragenomic syntenic analysis in *P. nigrum*.** The median *Ks* values of the paralogous syntenic blocks were used to infer their duplication timing, and then the syntenic blocks were color-coded according to the median *Ks* value of the anchor gene pairs in the block. Blue means the *Ks* value ≤ 0.3; purple represents 0.3 < *Ks* ≤ 0.8; and orange indicates *Ks* > 0.8.

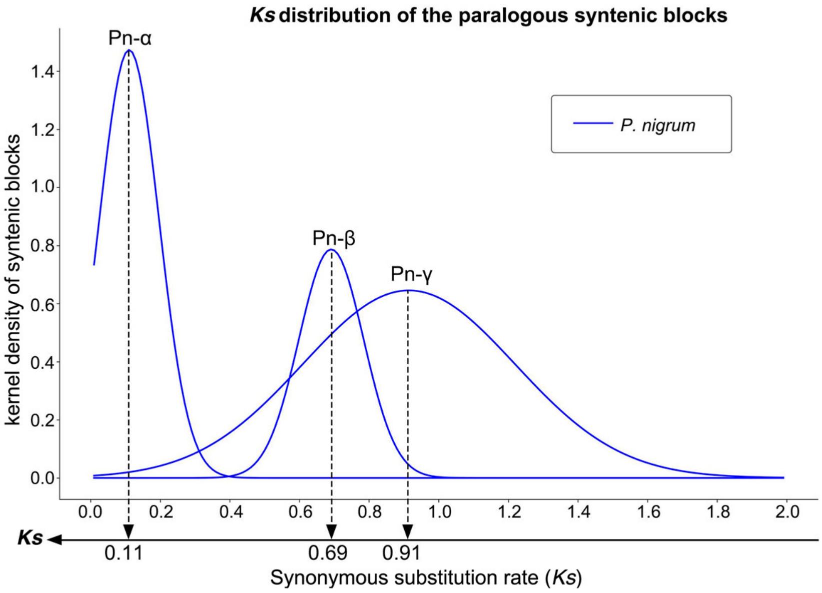


**Supplementary Fig. 3.6 | *Ks* distribution of the identified syntenic blocks in *P. nigrum*.** Pn-α, Pn-β, and Pn-γ represent the three rounds of WGDs detected here in *P. nigrum*.

### 3.3 Timing of the previously identified WGDs in magnoliids

**Materials and Methods**

Genomic studies of *C. kanehirae* and *P. americana* (Lauraceae, Laurales) have proposed two WGD events occurred in their evolutionary history, and *L. chinense* (Magnoliaceae, Magnoliales) has experienced one ancient WGD[68-70]. Interestingly, isozyme data suggested ancient WGD events in both Laurales and Magnoliales[71]. On the basis of EST analysis, Cui *et al.* (2006) determined that the WGD detected in *P. americana* and *Liriodendron tulipifera* was not shared[72]. Recently, the *Ks* analyses in the *Litsea* (Lauraceae) genome suggested that an ancient WGD occurred just before the divergence of Laurales and Magnoliales[73]. However, the wintersweet (*Chimonanthus praecox*; Calycanthaceae, Laurales) genome study proposed no WGD event was shared by *Cinnamomum* and *Liriodendron* using *Ks* approach dating these WGDs[74]. As yet, it remains unclear if these sister clades (Laurales, Magnoliales) in fact share any ancient WGD events. We employed the integrated approaches of synteny, *Ks*, and phylogenomic analyses to investigate the timing of these WGDs. *Ks* values were estimated using the Nei-Gojobori approach[63], and the *Ks* distribution was fitted as a mixture of multiple normal distributions. We also adopted a kernel function analysis to further classify the syntenic blocks based on the *Ks* values of anchor genes. Here, *Ks* correction was applied by using grape (*V. vinifera*) as a comparing reference to make its divergence (*Ks*) similar to the studied magnoliid genomes. Detailed methods were described in the previous studies[61,75].

**Results and Discussion**

The intergenomic syntenic depth ratio between *A. fimbriata* and *L. chinense* is 1:2, and the ratio between *A. fimbriata* and *C. kanehirae* is 1:4 (Fig. 2d and Supplementary Figs. 3.7 and 3.8). These results are consistent with the previously identified two WGDs in *C. kanehirae*[70] and one WGD in *L. chinense*[69]. We performed *Ks* analyses of the syntenic anchor gene pairs in these genomes. In *C. kanehirae* paralogous regions, the *Ks* distribution of anchor genes showed a clear bimodal pattern with *Ks* peaks at 0.535 (±0.06) and 0.858 (±0.09), which is consistent with the two WGDs (Supplementary Fig. 3.9a and Supplementary Table 3.2). The *Ks* distribution of *L. chinense* showed one major peak at 0.679 (±0.07) (Supplementary Fig. 3.9a and Supplementary Table 3.2). The *Ks* values of the orthologous gene pairs between *C. kanehirae* and *L. chinense* were also calculated to estimate the divergence between these genomes and the relative timing of their split. After evolutionary rate correction, the larger *Ks* peak of the *C. kanehirae* genome shows a little larger than the peak of the *C. kanehirae* - *L. chinense* divergence, but the evidence from the *Ks* analyses seems hard to clarify if the ancient WGD in *C. kanehirae* was shared by *L. chinense* (Supplementary Fig. 3.9b and Supplementary Table 3.3).

We further performed integrated phylogenomic and synteny analyses for the syntenic anchor gene pairs as described before[76,77]; 67.33% (1,350/2,005) of *C. kanehirae* gene pairs in 264 syntenic blocks were duplicated before the split of *C. kanehirae* and *P. americana*, and 20.45% (410/2,005) were duplicated before the divergence of *C. kanehirae* and *L. chinense* (Supplementary Table 3.4). Similarly, 70.33% (410/583) of the *P.americana* anchor gene pairs in 228 syntenic blocks were duplicated before the divergence of *C. kanehirae* and *P. americana* (Supplementary Table 3.5) and 13.55% (79/583) of the anchor gene pairs were duplicated before the divergence of *L. chinense* and *P.americana* (Supplementary Table 3.5). For 35 syntenic blocks of *L. chinense,* we found that 54.5% (462/848) of the anchor genes were duplicated before the split of *C. kanehirae* and *L. chinense* (Supplementary Table 3.6). These results strongly suggest that the identified WGD in *L. chinense* (named LCT) was shared with *C. kanehirae* and *P. americana*, and one additional WGD event (named CCT) occurred after the *C. kanehirae-L. chinense* divergence, and before the split of *P. americana* and *C. kanehirae*. Using the corrected *Ks* values, we inferred that the LCT event occurred approximately 74.09-83.75 million years ago (Ma), and the more recent CCT event occurred approximately 46.31-52.35 Ma (Supplementary Fig. 3.9b).

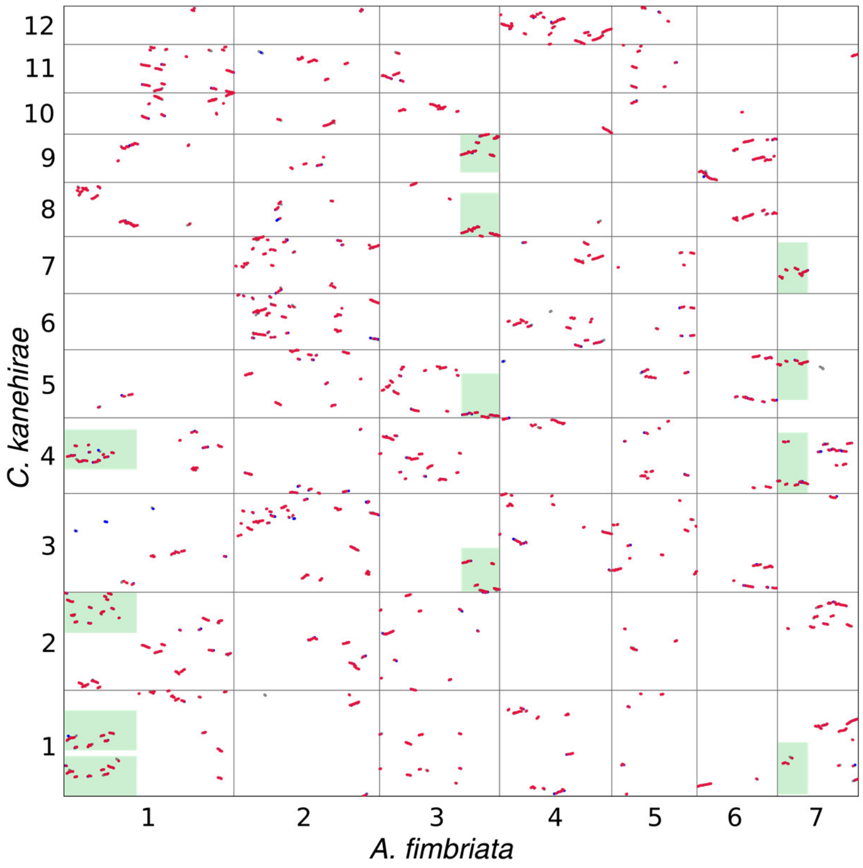


**Supplementary Fig. 3.7 | Synteny analysis between the *A. fimbriata* and *C. kanehirae* genomes.** Genomic syntenic blocks (⩾5 anchor gene pairs) were shown in dotplots according to their genomic locations in *A. fimbriata* and *C. kanehirae*. If the anchor gene pairs are the best BLAST hit among the genomes, they are plotted as red

dots; otherwise, they are shown in blue dots. Highlighted boxes indicate the selected orthologous regions between *A. fimbriata* and *C. kanehirae* with syntenic depth ratio of 1:4.

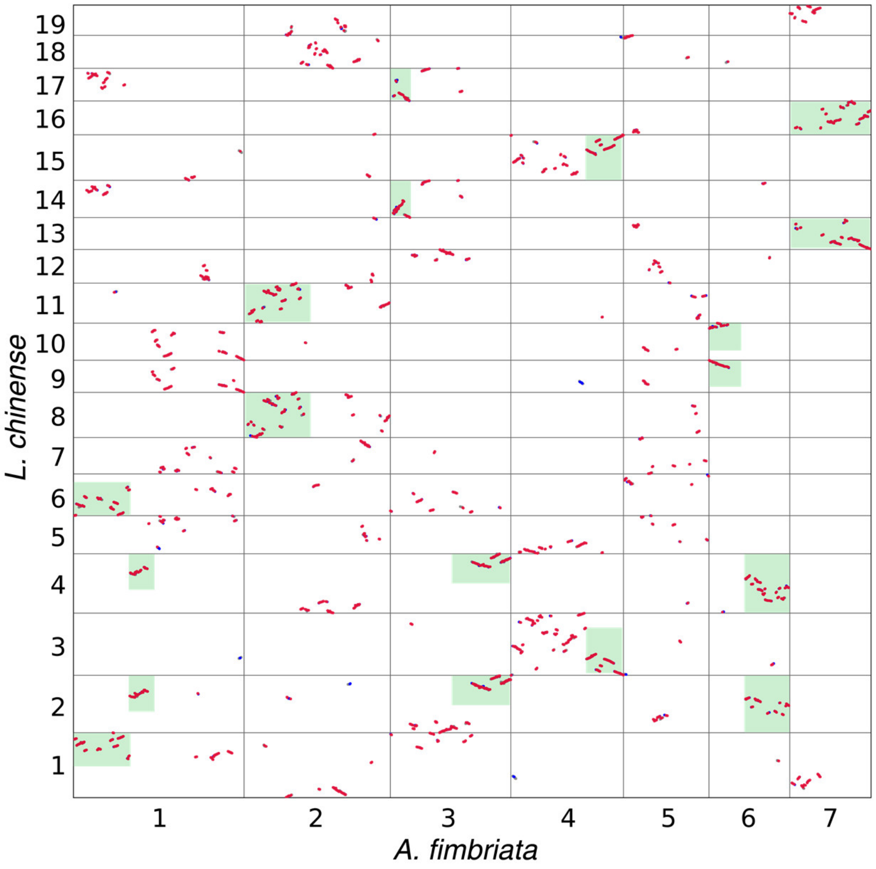


**Supplementary Fig. 3.8 | Synteny analysis between the *A. fimbriata* and *L. chinense* genomes.** Syntenic blocks (⩾5 anchor gene pairs) were shown in dotplots according to their genomic locations in *A. fimbriata* and *L. chinense*. If the anchor gene pairs are the best BLAST hit among the genomes, they are plotted as red dots; otherwise, they are shown in blue dots. Highlighted boxes indicate the selected orthologous regions between *A. fimbriata* and *L. chinense* that clearly show syntenic depth ratio of 1:2.

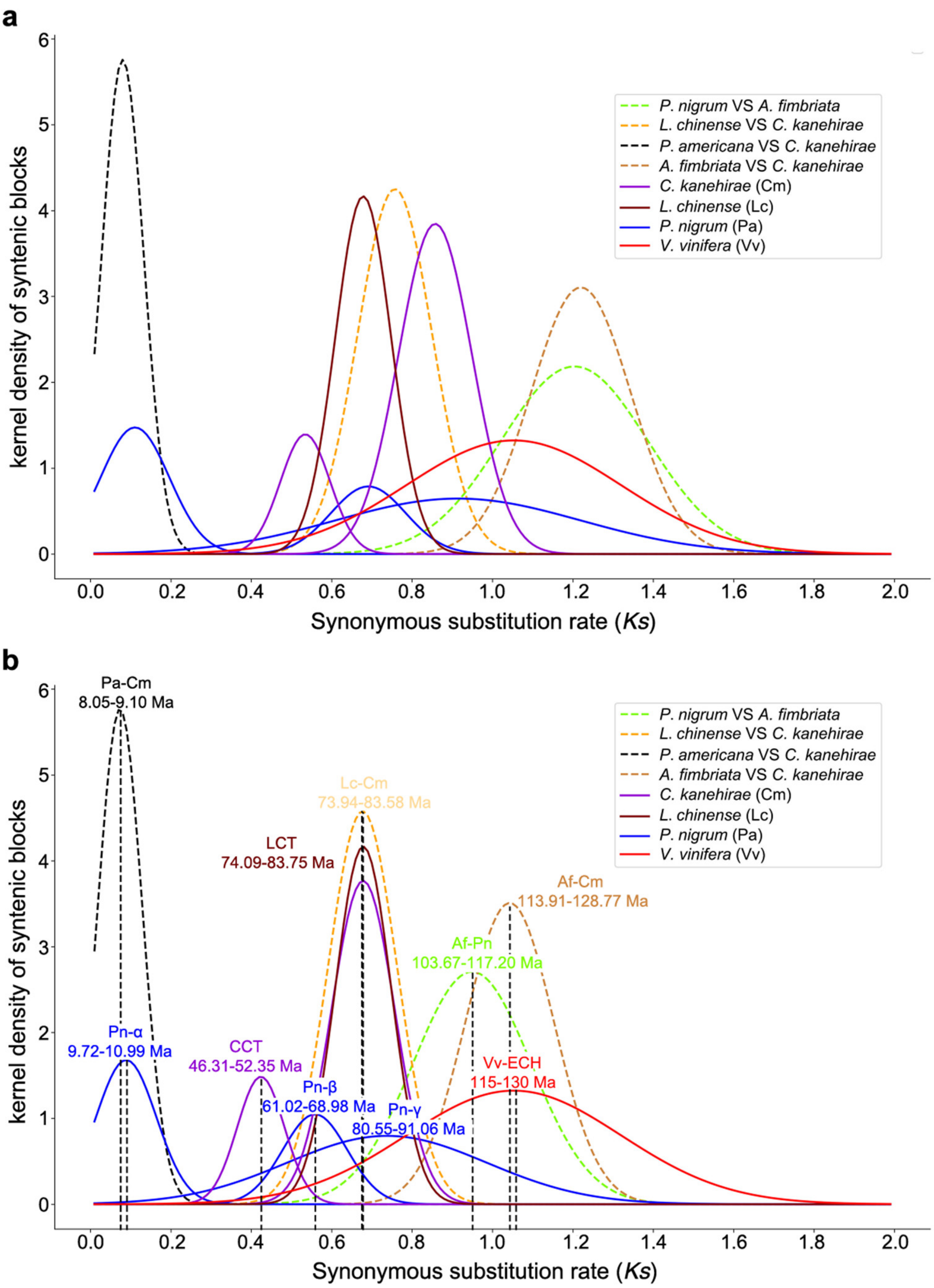


**Supplementary Fig. 3.9 | Original (a) and corrected (b) synonymous substitution rate (*Ks*) analyses.** *Ks* correction was applied by using the *V. vinifera* as a reference to standardize its divergence from magnoliid genomes; details of the *Ks* values are summarized in Supplementary Tables 3.2 and 3.3.

### 3.4 Genome structure comparisons identified genomic rearrangement events shared by magnoliids and monocots

**Materials and Methods**

The reference genome of *A. fimbriata* may be used to illustrate large genome structural rearrangements during the evolutionary history of angiosperms. In comparisons of the *A. fimbriata* genome with those of *A. trichopoda* and *N. colorata*, we identified several chromosome-level rearrangements between the former and these two angiosperms from the ANA grade, however, the timing of their occurrence requires further exploration. Comparisons among genomes of *A. fimbriata* and other magnoliids (*P. nigrum*, *L. chinense*, *Magnolia biondii*, *C. kanehirae*, and *Litsea cubeba*), the eudicots (*V. vinifera, Acer yangbiense*, *Tetracentron sinense*, and *A. coerulea*) as well as the monocots (*S. polyrhiza, A. comosus*, *Asparagus setaceus*, and *Elaeis guineensis*) could reveal whether or not the identified chromosomal rearrangements are shared among certain major groups of mesangiosperms.

Tracking the evolutionary history of the genomic rearrangement events include the following steps: 1) generating syntenic dotplots between the compared genomes and identifying orthologous genomic regions using aforementioned methods (Supplementary Note 3.1); 2) defining the involved regions of the genomic rearrangements and revealing the connection pattern of these orthologous regions in each studied genome; 3) reconstructing the ancestral connection pattern of these involved regions for the major clades of angiosperms based on orthologous regions in living species; 4) comparing the ancestral pattern of each clade with the predicted pattern of the most common ancestor of extant angiosperms and identifying the shared genomic rearrangements of major clades that potentially occurred before their divergence.

**Results and Discussion**

By comparing the *A. fimbriata* genome to the *A. trichopoda* and *N. colorata* genomes, we identified 1:1 and 1:2 syntenic depth ratios, respectively. We found that the chromosome 6 of *A. fimbriata* (Af6) separately matches chromosomes 7 and 9 of *A. trichopoda,* and chromosomes 4 and 12 and chromosomes 2 and 9 of *N. colorata* (Supplementary Fig. 3.10). The *N. colorata* genomic regions of chromosomes 4 and 12, as well as chromosomes 2 and 9, were generated by an independent WGD[59]. Similarly, we also found that chromosome 7 of *A. fimbriata* (Af7) has two separated orthologous syntenic regions in *A. trichopoda* and multiple regions in *N. colorata* (Fig. 3a and Supplementary Fig. 3.10). Given the *A. trichopoda* and *N. colorata* are two successively early diverging angiosperms, these syntenic results indicated that chromosomes 6 and 7 of *A. fimbriata* might be formed via two fusion events in an

ancestor of *A. fimbriata*, respectively. The entire Af6 and Af7 match with eight integrated orthologous regions in *P. nigrum*, respectively (Extended Data Fig. 4a). It confirms the correctness of the assembly of Af6 and Af7 in *A. fimbriata*, and further suggests the fusions occurred, at least, before the divergence of *A. fimbriata* and *P. nigrum*.

To get insights into the evolutionary history of these genomic rearrangements, we further compared the *A. fimbriata* genome with other representatives in the major clades of angiosperms. To better illustrate the genomic rearrangements related to chromosome 6 of *A. fimbriata*, we defined the L and R regions for the Af6 (Af6L and Af6R) according to the synteny relationship between *A. fimbriata* and *Amborella* (Supplementary Fig. 3.10a). When comparing the Af6 to the genomes of *C. kanehirae* and *L. cubeba* (Laurales), we identified two orthologous regions for the Af6L and four orthologous regions for the Af6R, in which only the syntenic region in the chromosome 9 of *C. kanehirae* (Supplementary Fig. 3.11a), as well as chromosome 11 of *L. cubeba*, shows an integrated L-R orthologous pattern (Supplementary Fig. 3.11c). When comparing to the genomes of *L. chinense* and *M. biondii* (Magnoliales), both of the orthologous regions for the Af6L and Af6R are located in separated chromosomes (Extended Data Fig. 4b,c). Given the WGD generating the two orthologous regions in the *L. chinense* genome shared with Laurales, we predict that the ancestral orthologous regions of the Af6L and Af6R might be separated in the common ancestor of Magnoliales and Laurales, and the integrated orthologous region in chromosome 9 of *C. kanehirae* might be resulted from a Laurales-specific fusion event. Interestingly, we also revealed that the entire Af6 matched the integrated orthologous regions in the studied four monocot genomes (*A. comosus*, *A. setaceus*, *S. polyrhiza* and *E. guineensis*) (Extended Data Fig. 5 and Supplementary Fig. 3.12). For example, the entire Af6 matches with chromosomes 4, 11, 12 and 16 in *E. guineensis*, which derived from the two WGDs[78-79]. However, when comparing to eudicot genomes, we found the orthologous region of Af6L and Af6R are located in different set of chromosomes, in which the syntenic relationships exhibit the Af6L matching to Vvi2, Vvi15, and Vvi16 and the Af6R matching to Vvi3, Vvi4, and Vvi18 in the *V. vinifera* (Vvi) genome (Supplementary Fig. 3.13a). Similarly in other studied eudicot genomes (*T. sinense*, *A. yangbiense*, and *A. coerulea*), the orthologous regions of Af6L and Af6R are also located in separated chromosomes (Supplementary Figs. 3.13 and 3.14). In summary, the orthologous regions of Af6L and Af6R are in different set of chromosomes in *Amborella*, *Nymphaea*, Laurales, Magnoliales, and eudicots, and the breakpoints between Af6L and Af6R are likely in similar genomic locations (Af6:7.22 Mb) when examined by microsynteny analyses (Supplementary Figs. 3.15 and 3.16). The integrated regions of L and R were revealed in Piperales and monocots, suggesting either a fusion event connected the L and R regions in a common ancestor of monocots and magnoliids (with a further fission event in the common ancestor of Laurales and Magnoliales) or a paralleling evolution led to the

"L-R" fusion in the Piperales and monocots (Extended Data Figs. 4a and 5 and Supplementary Fig. 3.12).

Similarly, for the chromosome 7 of *A. fimbriata*, we defined the ordered regions of E[A1]-A2-B1-B2 for the Af7 and as shown in the Fig. 3a, where E[A1] represents A1 region overlapped with the E region. Comparison between *A. fimbriata* and *L. chinense* (Lch), the orthologous regions of the A1-A2-B1-B2 of Af7 are integrated in *L. chinense* genome which located in Lch13 and Lch16 (Fig. 3a and Extended Data Fig. 4b), while the orthologous regions of the E part of the Af7 (Af7E) located in other set of chromosomes which are Lch1 and Lch19 (Fig. 3a and Extended Data Fig. 4b). Comparison between *A. fimbriata* and *C. kanehirae* (Ck) identified four orthologous regions of the A1-A2-B1-B2 in *C. kanehirae* (two on Ck1, Ck2, and Ck4, these generated from the two WGDs) and three orthologous regions of Af7E located in Ck4, Ck5, and Ck7 (Supplementary Fig. 3.11a and Extended Data Fig. 6). In addition, the same fused syntenic pattern of A1-A2-B1-B2 of Af7 also present in *M. biondii* and *L. cubeba* genomes, whereas the orthologous regions of Af7E are located in different chromosomes (Extended Data Fig. 4c and Supplementary Fig. 3.11c). Therefore, these results indicated that the fusion of A1-A2 and B1-B2 regions of Af7 occurred at least before the divergence of magnoliids, while a translocation event involving Af7E seems to be Piperales-specific.

Further integrating syntenic evidence from comparisons of the *A. fimbriata* genome with the two representatives of the ANA grade (*A. trichopoda* and *N. colorata*) and with other magnoliids, we found that the fusion event of A1-A2-B1-B2 was associated with two other genomic regions on Af1 (defined regions of D1, D2, and C2) and Af3 (defined C1 region) as illustrated in Fig. 3a. In *A. trichopoda* genome, the orthologous regions of A1, A2, and C1 of *A. fimbriata* are located on chromosome 10, while the orthologous regions of B1 and C2 of *A. fimbriata* are located on the chromosome 12 (Fig. 3a and Supplementary Fig. 3.10a). In the *L. chinense* and *M. biondii* (Magnoliales) and *P. nigrum* (Piperales), the orthologous regions of the C1 and C2 of *A. fimbriata* located in same chromosome (Fig. 3a and Extended Data Figs. 4a-c and 6). In the *C. kanehirae* and *L. cubeba* (Laurales) genomes, two of the orthologous regions of the C1 and C2 of *A. fimbriata* located in separated locations of the same chromosome (likely resulted from genomic inversion events), and the other orthologous region of the C1 and C2 located in different chromosomes (Supplementary Fig. 3.11a,c and Extended Data Fig. 6c,d). These results suggested that the translocation of C2 into the D1 and D2 regions was specifically occurred in *A. fimbriata* (Fig. 3b and Extended Data Figs. 4 and 6). Moreover, we noticed many Laurales-specific fusion and inversion events that led to the connection patterns of (C1-A1-A2-B1-B2-D1-D2-C2) and (A1-B1-B2-D1-D2, C1, C2) in *C. kanehirae* and *L. cubeba* (Fig. 3b, Supplementary Fig. 3.11a,c and Extended Data Fig. 6c,d), which are different from the pattern in Magnoliales (Fig. 3a,b and Extended Data Figs. 4b,c

and 6a). Notably, the two paralogous regions in *L. chinense* (as well as in *M. biondii*) exhibit exactly the same connection pattern (A1-A2-B1-B2, C1-C2, D1-D2) (Fig. 3 and Extended Data Figs. 4b,c and 6a). Again, considering the ancient WGD shared by Magnoliales and Laurales, we predict that the identified connection pattern in Magnoliales is the ancestral pattern of Magnoliales and Laurales, while many additional rearrangements occurred in the Laurales following the extra recent WGD. Therefore, the connection patterns of the involved genomic regions in the ancestral genome of magnoliids are predicted as (A1-A2-B1-B2, C1-C2, D1-D2, and E) (Fig. 3b). In *A. trichopoda* genome, the connection pattern exhibited as (A1-A2-C1, B1-C2, B2, D1-D2, and E) (Fig. 3a,c and Supplementary Fig. 3.10a). In *N. colorata*, we found the connection patterns exhibiting as (A1-A2-C1, B1-B2-C2, D1-D2, and E) and (A1-A2, C1, B1-B2-C2, D1-D2, and E) (Fig. 3c and Supplementary Fig. 3.10b).

Then, we compared the genome structure of *A. fimbriata* with four monocots (*A. comosus*, *A. setaceus*, *S. polyrhiza* and *E. guineensis*), four eudicots (*T. sinense*, *V. vinifera*, *A. yangbiense*, and *A. coerulea*), and *Ceratophyllum demersum*, respectively. To exclude the effect of the Piperales- and *A. fimbriata*-specific translocations (including the merge of E[A1] and the translocation of C2 into D1-D2 region), we also performed these intergenomic comparisons using *L. chinense* as another companion reference (Extended Data Figs. 4-6 and Supplementary Figs. 3.11-3.14), because these specific translocations are absent in the *L. chinense* genome. In the four monocot genomes, we found that the connection pattern of the involved genomic regions consistently shows as (A1-A2-B1-B2, C1-C2, D1-D2, and E), therefore such pattern could be referred as the ancestral pattern of monocots (Fig. 3b, Extended Data Fig. 5 and Supplementary Fig. 3.12). Notably, the ancestral connection pattern of monocots seems to be the same as that of magnollids, but different from those identified in *A. trichopoda* and *N. colorata* (Fig. 3c). In four eudicot genomes, the connection patterns of the involved genomic regions all show as (A1-A2-C1, B1-B2, D1-D2-C2, and E), which could represent the ancestral pattern of eudicots (Fig. 3b and Supplementary Figs. 3.13 and 3.14). The connection of A1-A2-C1 in eudicots is consistent with that in *A. trichopoda* and *N. colorata* genomes, but differ from the pattern in magnoliids and monocots (A1-A2-B1-B2) (Fig. 3c). Further microsynteny examinations revealed that the locations of the breakpoints (Af7:7.85 Mb) is similar when comparing *A. fimbriata* with these genomes of monocots, eudicots, *Amborella*, and *N. colorata* (Supplementary Fig. 3.17). We also compared the genomic structure of two monocot representatives (*A. comosus* and *S. polyrhiza*) with two eudicots (*T. sinense* and *V. vinifera*) to further confirmed these connection patterns (Supplementary Figs. 3.18-3.21).

In *C. demersum*, we found a few signals supporting the integrated orthologous regions of the *A. fimbriata* A1-A2-B1-B2 on chromosomes 2 and 5, and the integrated orthologous regions of the *A. fimbriata* C2-D1-D2 on chromosomes 1 and 3

(Supplementary Fig. 3.22). Given the three lineage-specific WGDs in *Ceratophyllum* and the followed extensive chromosome merges (currently with only 12 chromosomes), the identified connection pattern in *Ceratophyllum* could be recently derived rather than inherited from the common ancestor of the major clades of angiosperms. Therefore, we are not able to confidently suggest the chromosomal rearrangement history in *Ceratophyllum* using current genome, and excluded this lineage in the following investigations.

From the above analyses, the connection patterns of involved genomic regions in most recent common ancestor of extant angiosperms could be inferred as (A1-A2-C1, B1-B2-C2, D1-D2, and E) (Fig. 3c). We reconstructed the history of chromosomal rearrangements following the divergence of extant angiosperms (Fig. 3c). After its split from other angiosperms, the *Amborella* genome experienced a lineage-specific genomic translocation event involving the B2 region that was separated from the ancestral B1-B2-C2 region. In *N. colorata* genome, one set of the paralogous regions (from the recent WGD) remains as the ancestral angiosperm connection pattern, while the other set experienced a translocation event involving the C1 region that was separated from the ancestral A1-A2-C1 region. In the common ancestor of eudicots, the C2 region was split out of the B1-B2-C2 region, and connected with the D1-D2 to form a D1-D2-C2 connection pattern. In the common ancestor of monocots, a reciprocal genomic translocation occurred involving: two fissions resulted the split of the C1 region out of the ancestral A1-A2-C1 region and the C2 region out of the ancestral B1-B2-C2 region; two fusions resulted the fusion of the C1-C2 region and the A1-A2-B1-B2 region. Notably, such scenario was also identified in magnoliids, which may represent an important genomic synapomorphy between magnoliids and monocots.

Therefore, our results suggested that the chromosomal rearrangement events we detected here were shared by magnoliids and monocots, and likely not shared with eudicots. It seems hard to completely exclude the possibility that ancient eudicots also shared these rearrangements, but they were subsequently reversed these exchanges soon after the divergence of eudicots from magnoliids and monocots. However, given the similar genomic structure between eudicots and representatives of the ANA grade, we speculate the chance of such reverse scenario should be very small, because both of the fissions and fusions in the common ancestor of eudicots would have had to occur at the previous break points and also in a very limited time frame. Thus, we propose that the reciprocal genomic translocations only shared by magnoliids and monocots may represent an important genomic synapomorphy and that magnoliids might be sister to monocots while the clade of eudicots diverged earlier (Fig. 3c).

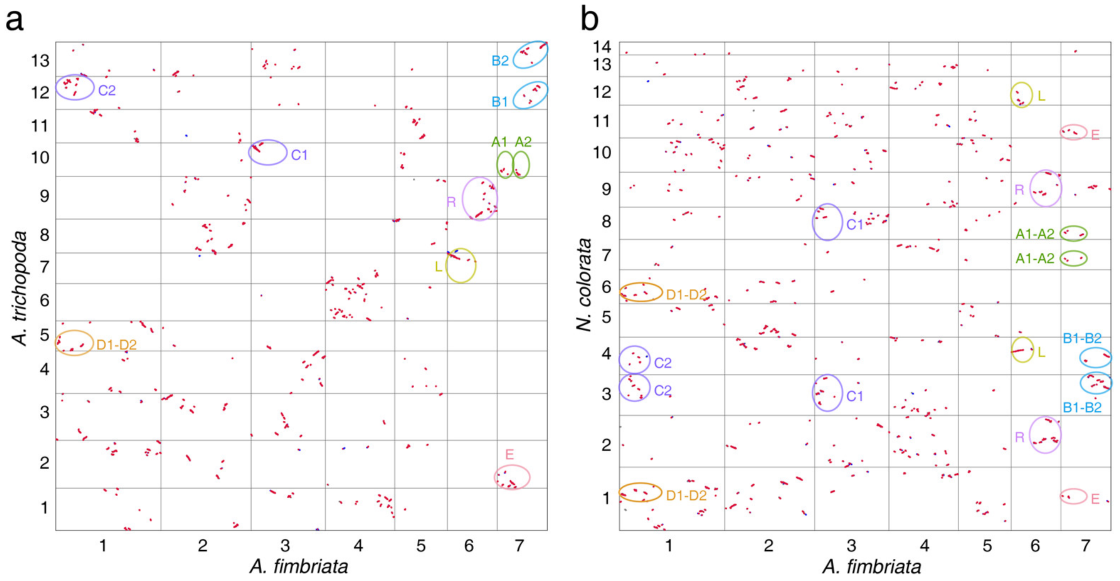


**Supplementary Fig. 3.10 | Genomic comparisons of the *A. fimbriata* with the *A. trichopoda* and *N. colorata* genomes.** (a) Syntenic dotplot between the *A. fimbriata* and *A. trichopoda* genomes. (b) Syntenic dotplot between the *A. fimbriata* and *N. colorata* genomes. Genomic syntenic blocks (⩾5 anchor gene pairs) were shown in dotplots according to their genomic locations in *A. fimbriata*, *A. trichopoda*, and *N. colorata*. If the anchor gene pairs are the best BLAST hit among the genomes, they are plotted as red dots; otherwise, they are shown in blue dots. The circled blocks indicate the syntenic blocks related to the rearranged genomic regions associated with the Chr6 and Chr7 in *A. fimbriata*, and they are marked with A1, A2, B1, B2, C1, C2, D1, D2, E, L, and R according to the defined regions in *A. fimbriata*.

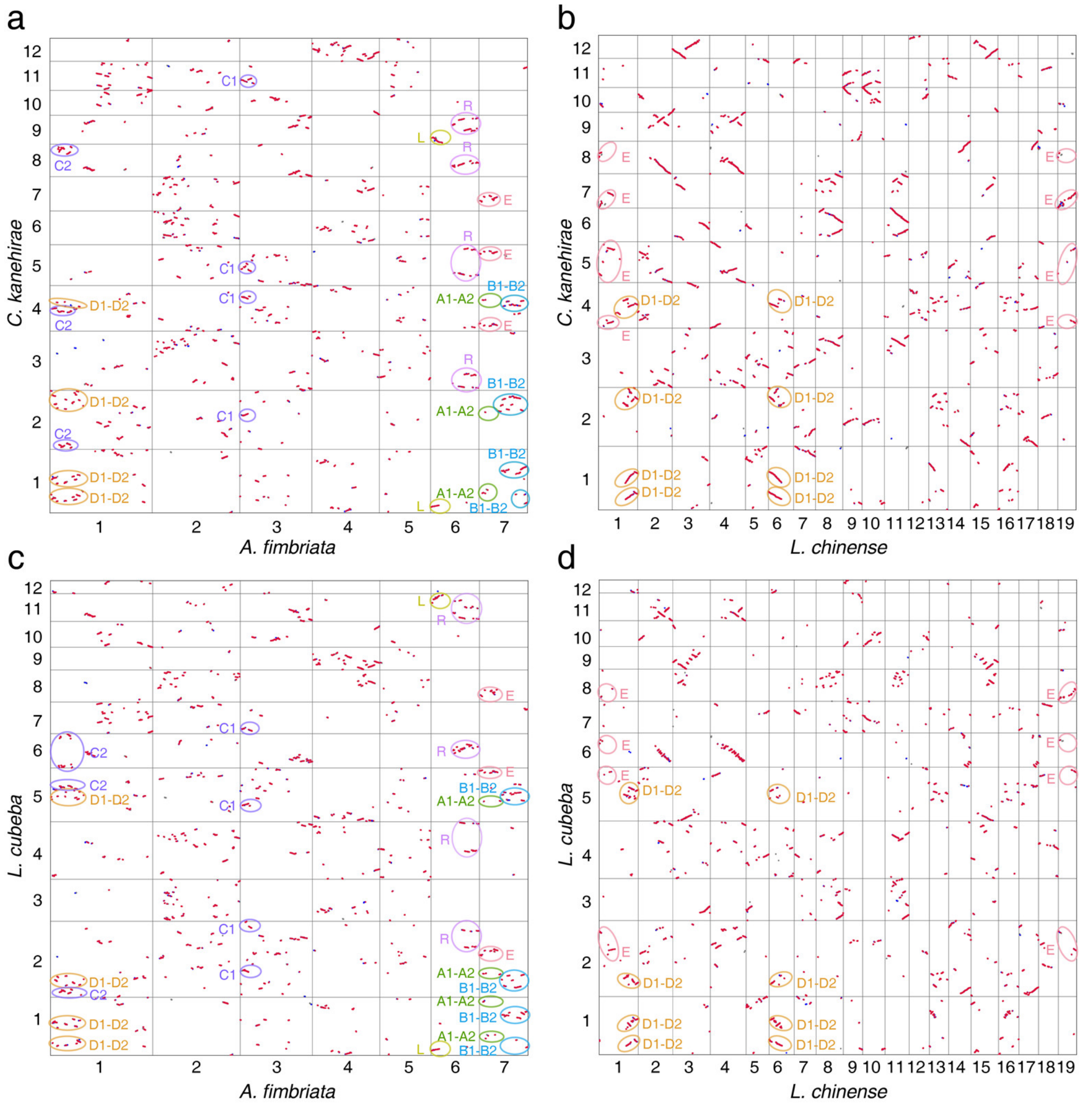


**Supplementary Fig. 3.11 | Genomic comparisons of the *A. fimbriata* and *L. chinense* with the *C. kanehirae* and *L. cubeba* genomes, respectively.** (a) Syntenic dotplot between the *A. fimbriata* and *C. kanehirae* genomes. (b) Syntenic dotplot between the *L. chinense* and *C. kanehirae* genomes. (c) Syntenic dotplot between the *A. fimbriata* and *L. cubeba* genomes. (d) Syntenic dotplot between the *L. chinense* and *L. cubeba* genomes. The orthologous region of the D1-D2 and E in *C. kanehirae* and *L. cubeba* could be further verified by the syntenic relationship to the corresponding D1-D2 and E regions in *L. chinense*.

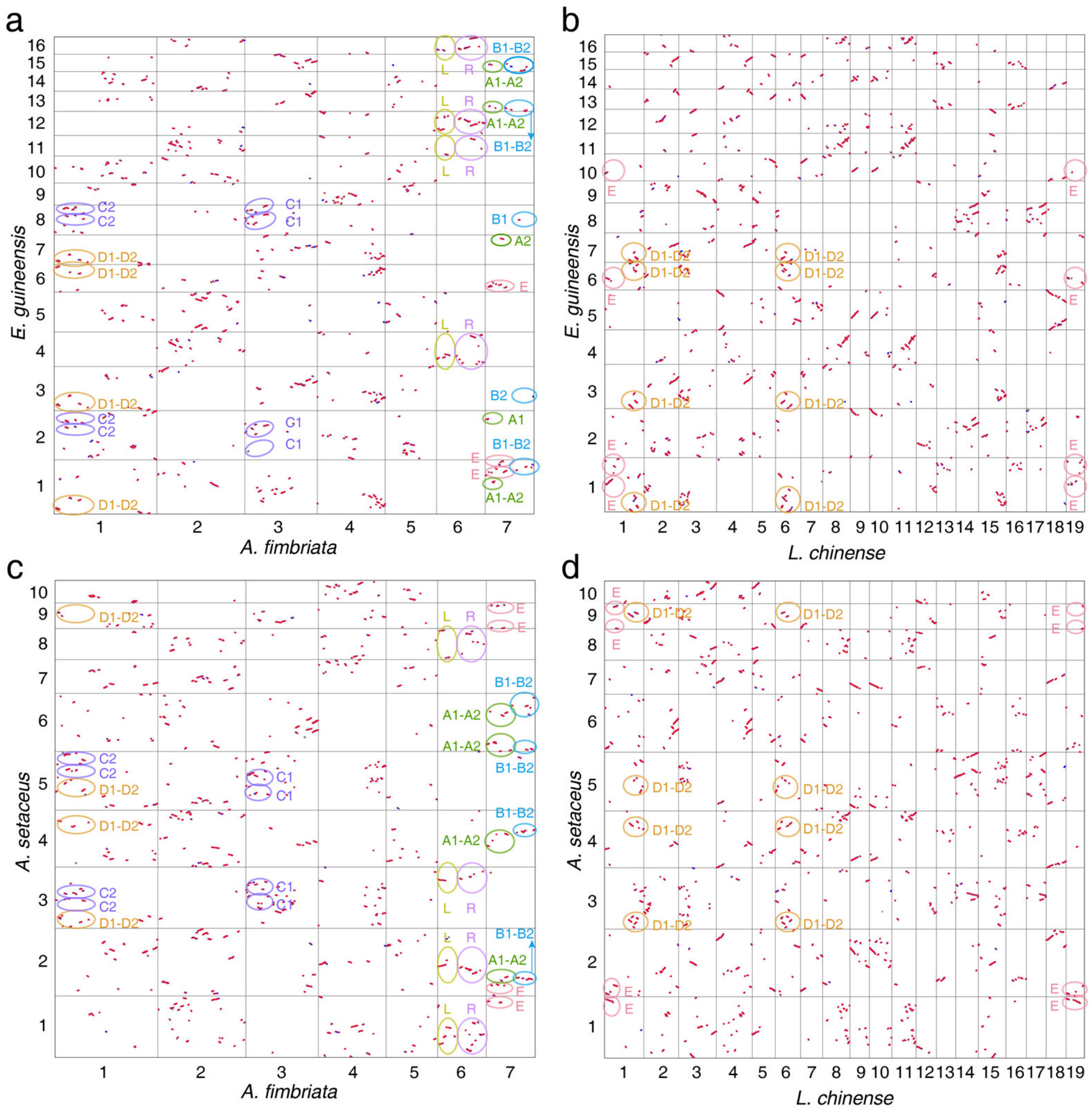


**Supplementary Fig. 3.12 | Genomic comparisons of the *A. fimbriata* and *L. chinense* with the *E. guineensis* and *A. setaceus* genomes.** (a) Syntenic dotplot between the *A. fimbriata* and *E. guineensis* genomes. (b) Syntenic dotplot between the *L. chinense* and *E. guineensis* genomes. (c) Syntenic dotplot between the *A. fimbriata* and *A. setaceus* genomes. (d) Syntenic dotplot between the *L. chinense* and *A. setaceus* genomes. The orthologous region of the D1-D2 and E in *E. guineensis* and *A. setaceus* could be further verified by the syntenic relationship to the corresponding D1-D2 and E regions in *L. chinense*.

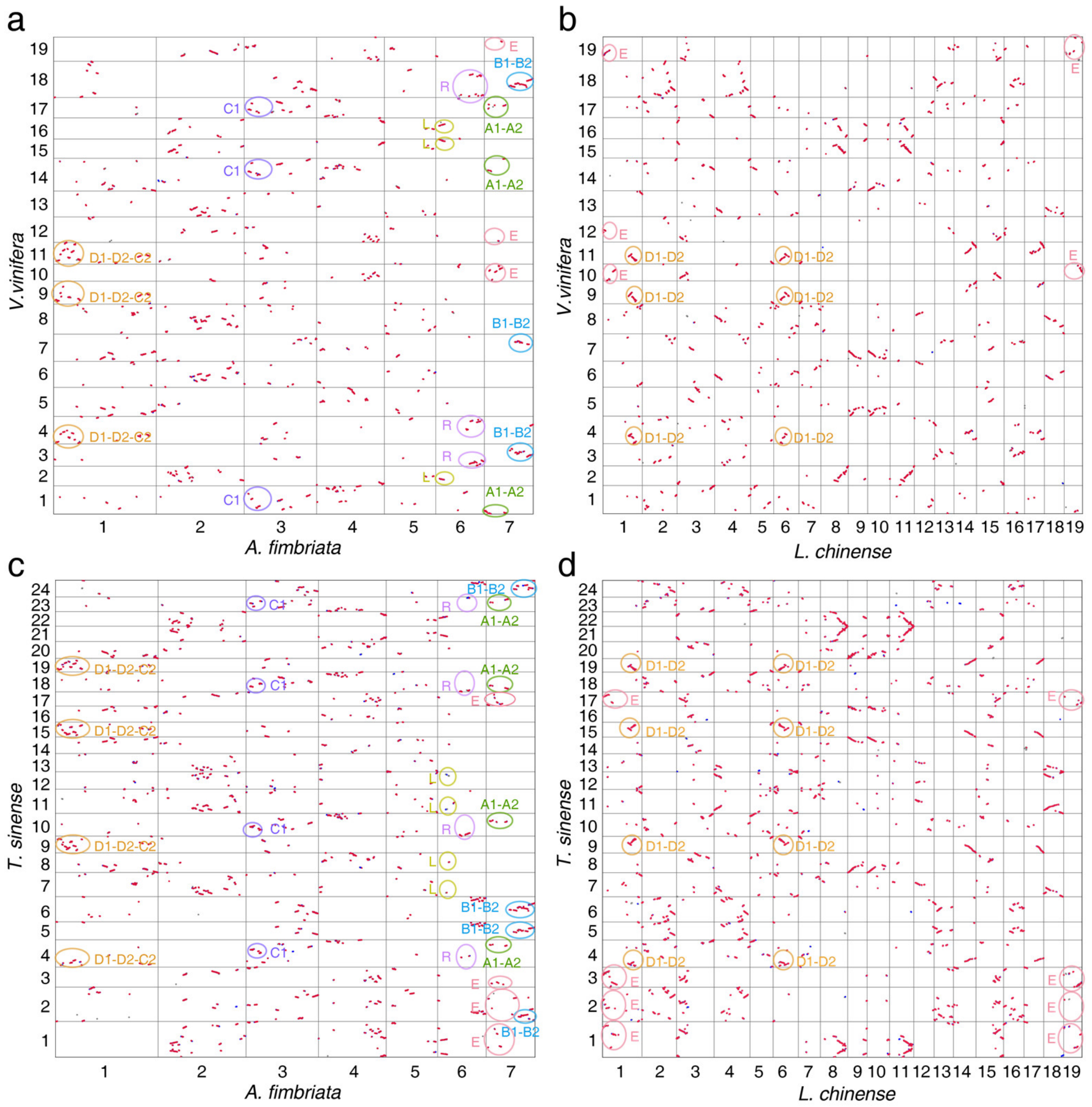


**Supplementary Fig. 3.13 | Genomic comparisons of the *A. fimbriata* and *L. chinense* with the *V. vinifera* and *T. sinense* genomes.** (a) Syntenic dotplot between the *A. fimbriata* and *V. vinifera* genomes. (b) Syntenic dotplot between the *L. chinense* and *V. vinifera* genomes. (c) Syntenic dotplot between the *A. fimbriata* and *T. sinense* genomes. (d) Syntenic dotplot between the *L. chinense* and *T. sinense* genomes. The orthologous region of the D1-D2 and E in *V. vinifera* and *T. sinense* could be further verified by the syntenic relationship to the corresponding D1-D2 and E regions in *L. chinense*.

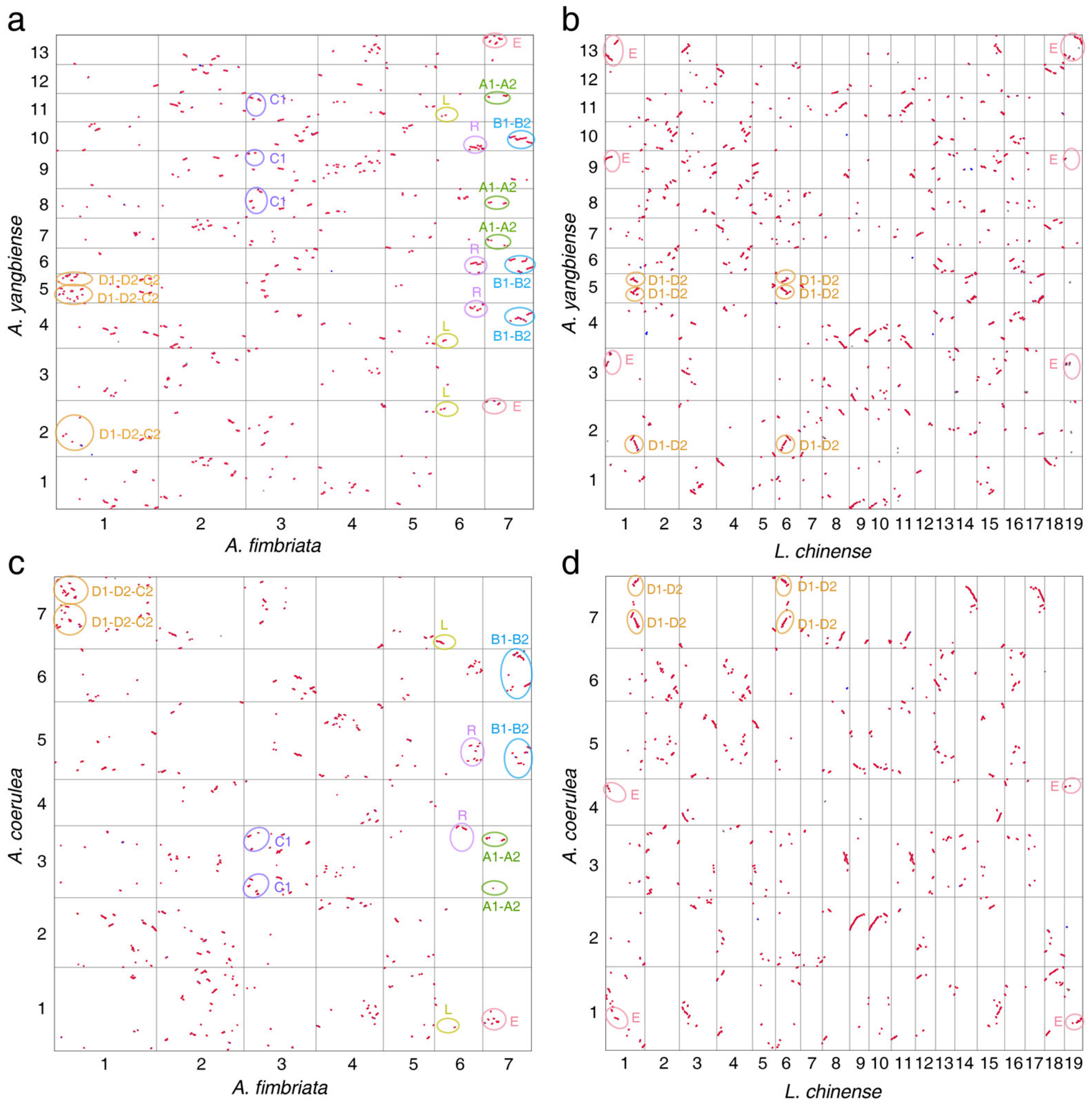


**Supplementary Fig. 3.14 | Genomic comparisons of the *A. fimbriata* and *L. chinense* with the *A. yangbiense*, and *A. coerulea* genomes.** (a) Syntenic dotplot between the *A. fimbriata* and *A. yangbiense* genomes. (b) Syntenic dotplot between the *L. chinense* and *A. yangbiense* genomes. (c) Syntenic dotplot between the *A. fimbriata* and *A. coerulea* genomes. (d) Syntenic dotplot between the *L. chinense* and *A. coerulea* genomes. The orthologous region of the D1-D2 and E in *A. yangbiense*, and *A. coerulea* could be further verified by the syntenic relationship to the corresponding D1-D2 and E regions in *L. chinense*.

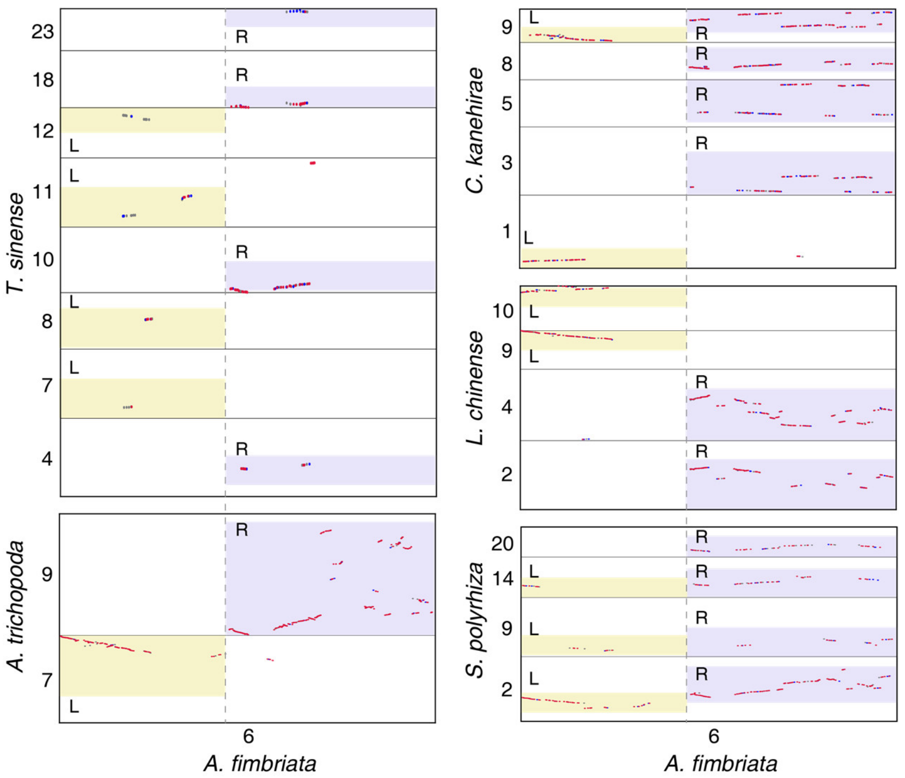


**Supplementary Fig. 3.15 | Local syntenic relationships among the selected genomic regions that associated with the structural rearrangements of *A. fimbriata* chromosome 6.** The local syntenic blocks identified between the *A. fimbriata* genome and the genomes of *A. trichopoda*, *T. sinense*, *S. polyrhiza*, *L. chinense*, and *C. kanehirae*. These syntenic blocks were highlighted and named according to the defined regions in *A. fimbriata*, similar to the Supplementary Fig. 3.10.

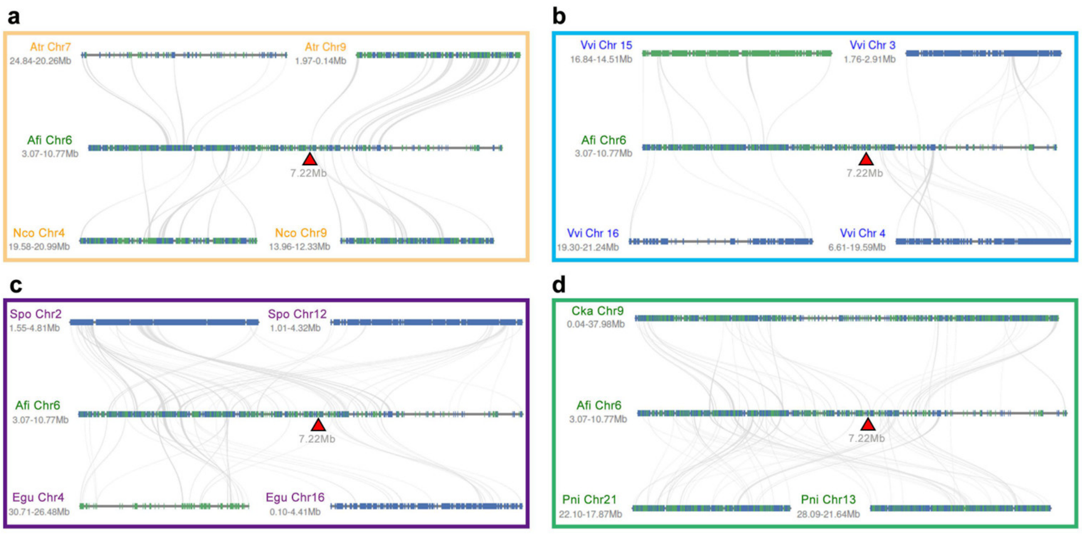


**Supplementary Fig. 3.16 | Intergenomic microsynteny using the chromosome 6 of *A. fimbriata* as a reference.** Syntenic relationships flanking the fusion point of

chromosome 6 of *A. fimbriata* (Afi) when comparing to the other genomes including (a) *A. trichopoda* (Atr) and *N. colorata* (Nco), (b) *V. vinifera* (Vvi), (c) *S. polyrhiza* (Spo) and *E. guineensis* (Egu), and (d) *P. nigrum* (Pni) and *C. kanehirae* (Cka). Rectangles represent annotated genes, and the grey lines connect syntenic gene pairs. The genomic fusion point is likely in the region around the locus indicated by the red triangle.

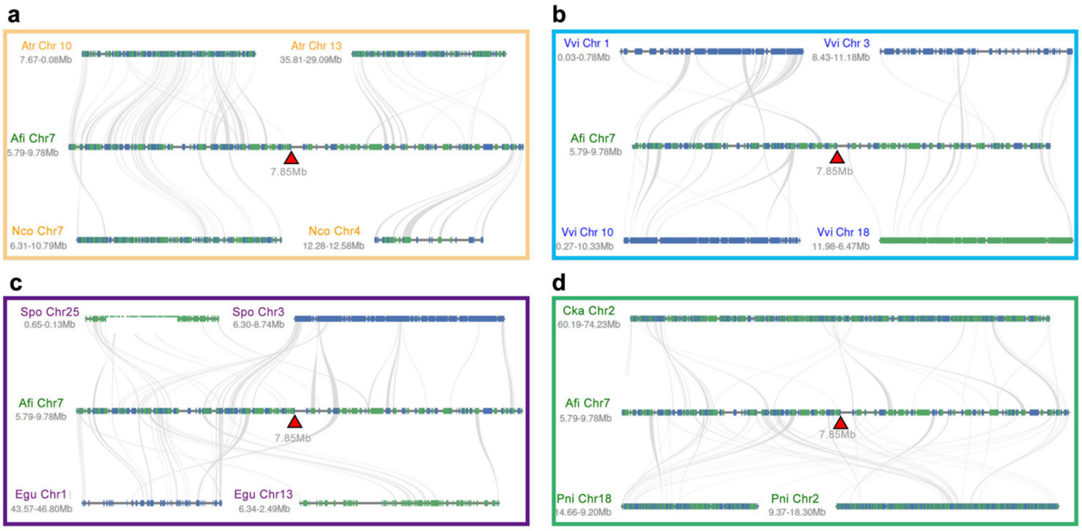


**Supplementary Fig. 3.17 | Intergenomic microsynteny using chromosome 7 of *A. fimbriata* as a reference.** Syntenic relationships flanking the fusion point of chromosome 7 of *A. fimbriata* (Afi) when compared to the other genomes including (a) *A. trichopoda* (Atr) and *N. colorata* (Nco), (b) *V. vinifera* (Vvi), (c) *S. polyrhiza* (Spo) and *E. guineensis* (Egu), and (d) *P. nigrum* (Pni) and *C. kanehirae* (Cka). Rectangles represent annotated genes, and the grey lines connect syntenic gene pairs. The genomic fusion point is likely in the region around the locus indicated by the red triangle.

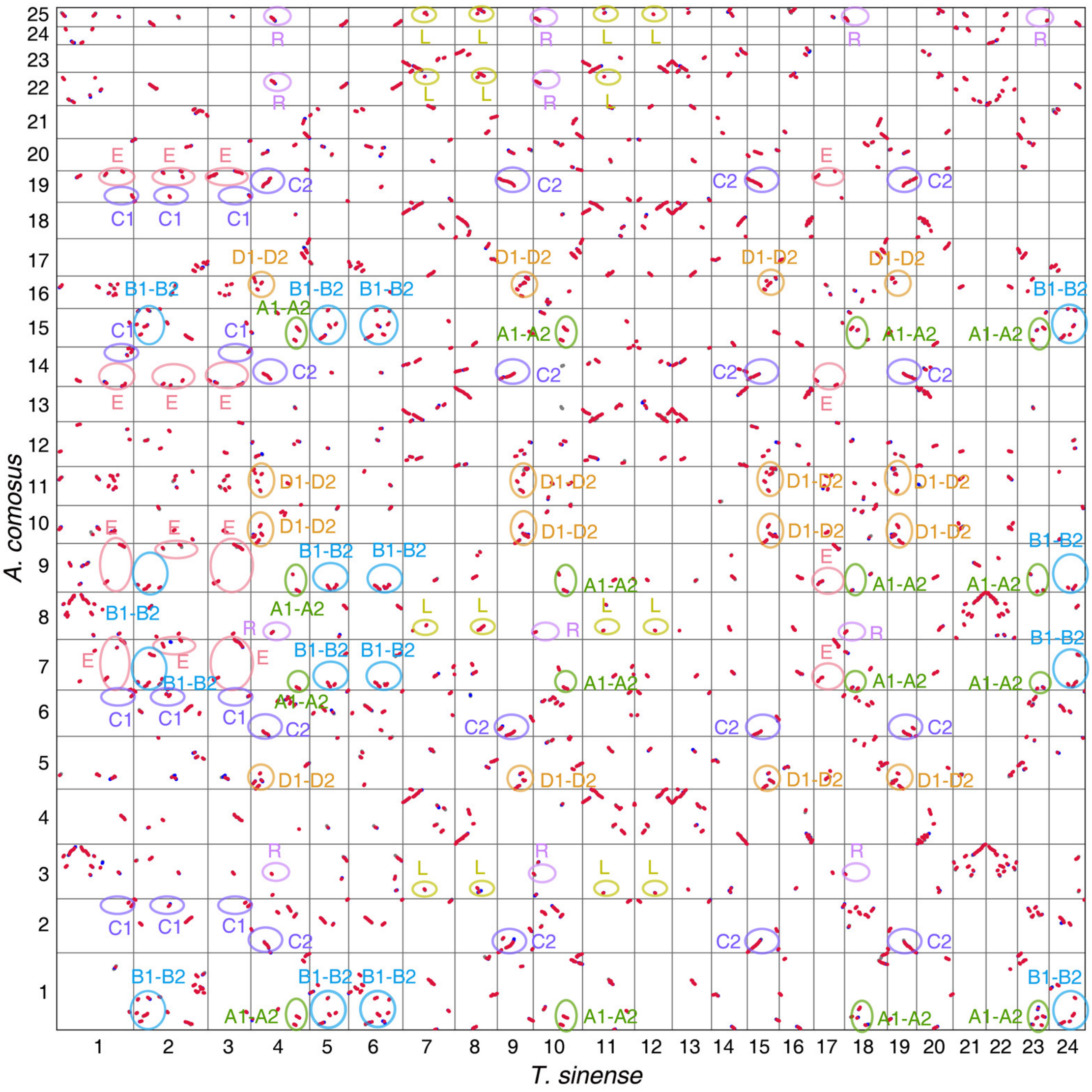


**Supplementary Fig. 3.18 | Genomic comparison of the *A. comosus* with the *T. sinense* genome.**

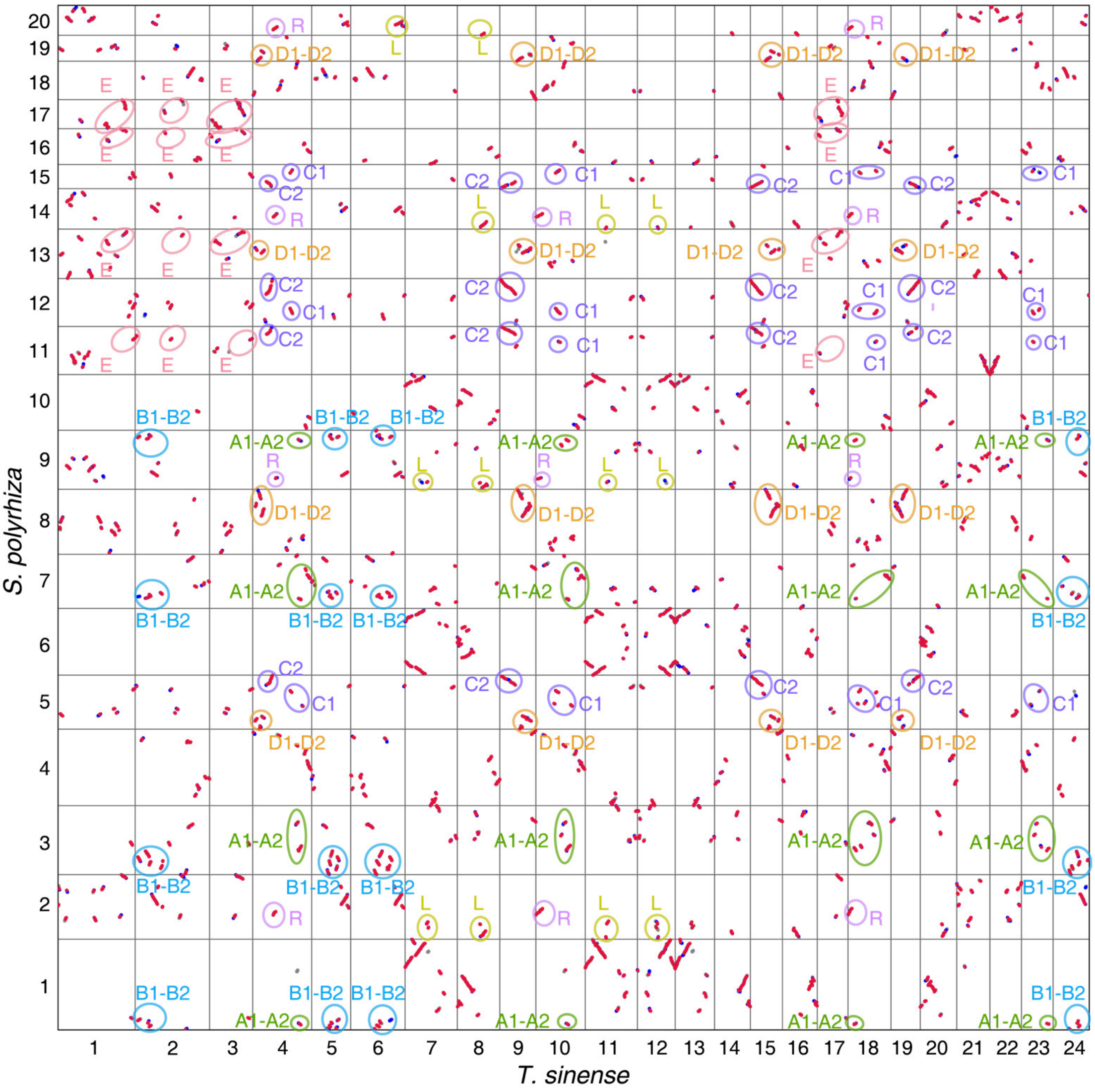


**Supplementary Fig. 3.19 | Genomic comparison of the *S. polyrhiza* with the *T. sinense* genome.**

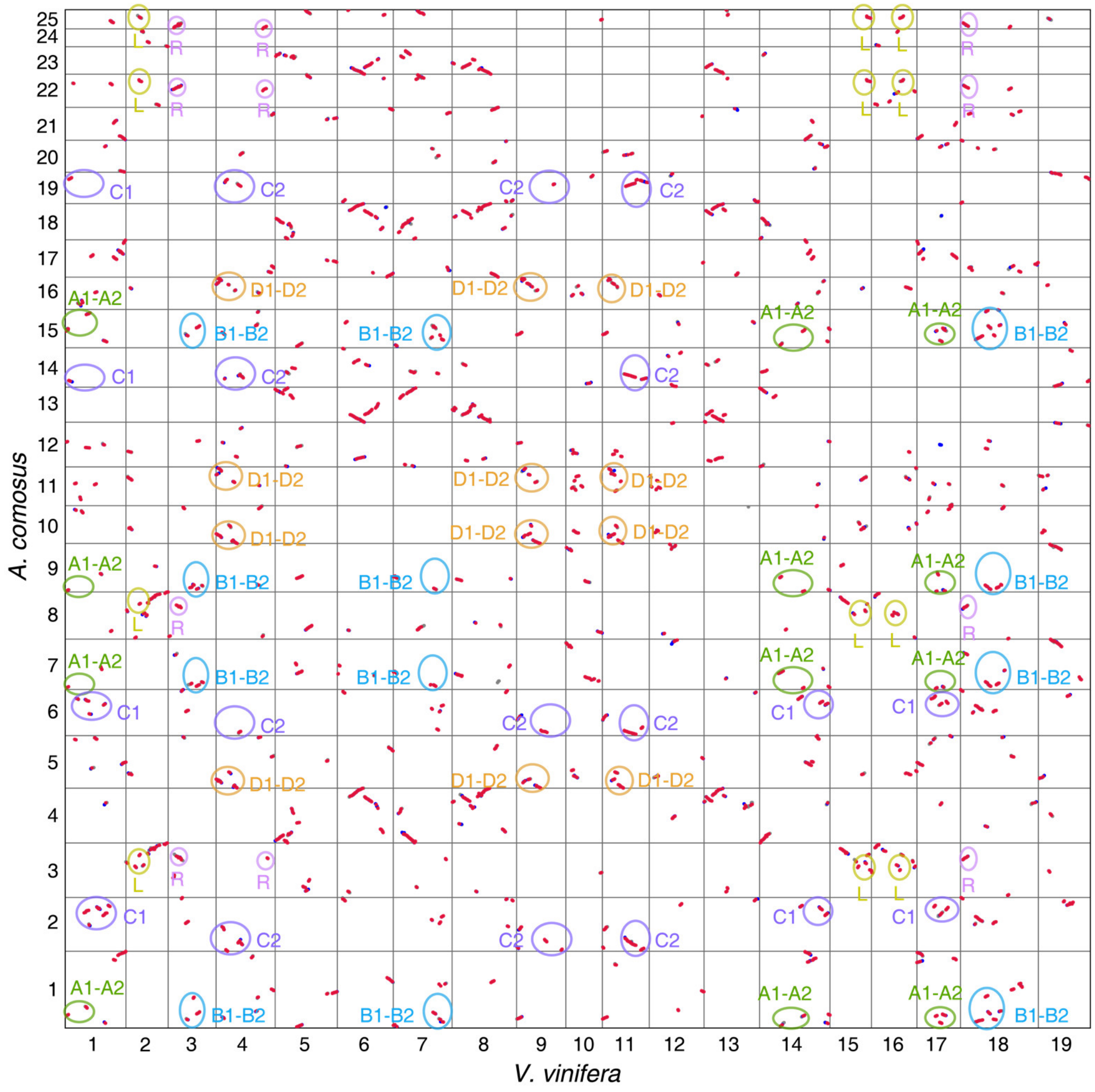


**Supplementary Fig. 3.20 | Genomic comparison of the *A. comosus* with the *V. vinifera* genome.**

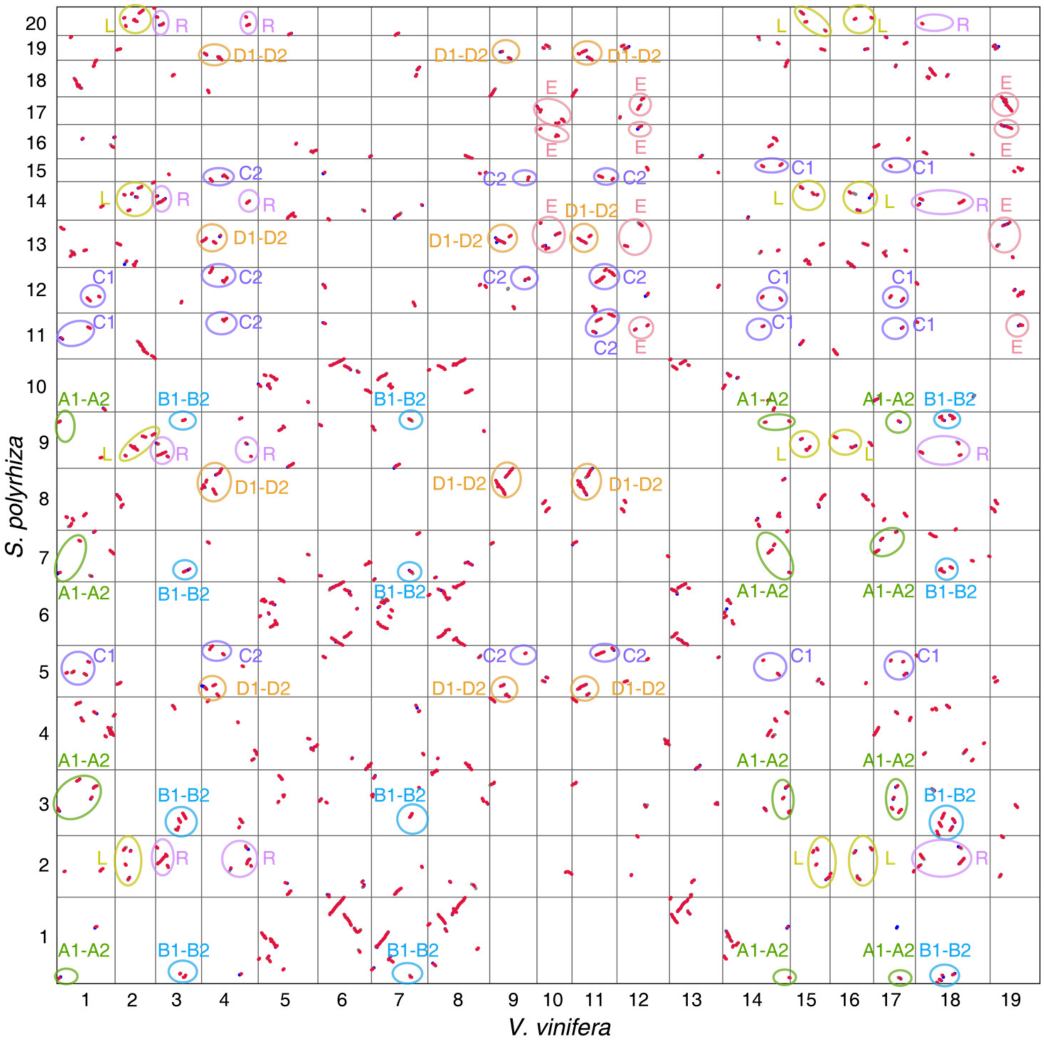


**Supplementary Fig. 3.21 | Genomic comparison of the *S. polyrhiza* with the *V. vinifera* genome.**

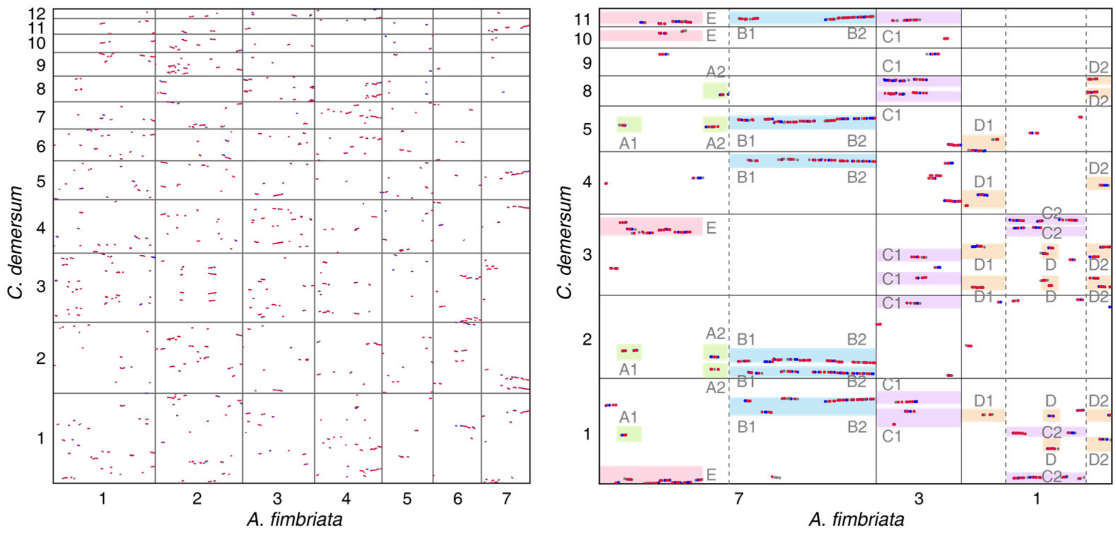


**Supplementary Fig. 3.22 | Genomic comparison of the *A. fimbriata* with the *C. demersum* genome.** (a) Syntenic dotplot between the *A. fimbriata* and the *C.*

*demersum* genome. (b) Local syntenic relationships among the selected genomic regions that associated with the structural rearrangements of *A. fimbriata* chromosome 7. Similar to the Fig. 3a, the specific genomic regions associated with the *A. fimbriata* chromosome 7 fusion were named regions of E, A1, A2, B1, B2, C1, C2, D, D1 and D2 as marked on top of the plot.

## 4 Phylogenomic investigation of the relationship among magnoliids, monocots, and eudicots

### 4.1 Phylogenomic analyses of strictly and mostly single-copy gene families

**Materials and Methods**

In order to clarify the phylogenetic position of magnoliids relative to eudicots and monocots, comprehensive phylogenomic analyses were performed using different datasets and approaches (Supplementary Table 4.1). Four main topologies were defined here as T1: ((magnoliids, (monocots, eudicots)), outgroup), T2: ((monocots, (eudicots, magnoliids)), outgroup), T3: ((eudicots, (monocots, magnoliids)), outgroup), and T4: ((eudicots, monocots, magnoliids), outgroup). Two strategies were used for screening OGs based on gene copy number: the strictly single-copy (SSC) and mostly single-copy (MSC) gene families.

**Strictly and mostly single-copy gene family identification**. Because the genomes of black pepper (*P. nigrum*) and opium poppy (*P. somniferum*) each experienced a very recent WGD event[66,80], we allowed them two gene copies at most, and the other 20 species strictly a single gene. A custom Python script was used to extract SSC gene families from the OrthoMCL results of 22 species (Supplementary Note 2.4). We also extracted MSC gene families which permitted some species to lack the homologous gene, but we still required the OGs with genes in at least 4 species (4/7) in eudicots, 3 species (3/5) in monocots, 3 species (3/5) in magnoliids, and 3 species (3/5) in outgroups (including 2 representatives from ANA grade, 2 gymnosperms, and one lycophyte).

**Phylogenetic analysis.** For each gene family, protein sequences were aligned using MUSCLE v3.8.31[81], and nucleotide sequences were then forced to fit the amino acid alignments using PAL2NAL v14[82]. We also forced nucleotide sequences on the amino acid alignments using a custom Python script to obtain codon-preserving alignments of nucleotide sequences. Finally, we retrieved four different alignments for each gene family: (1) amino acid (or peptide, pep) alignments; (2) nucleotide alignment (nucleotides forced to the amino acid alignment; or coding sequence, cds); (3) codon alignments with third-position removed (codon1&2); and (4) codon

alignments with first- and second-position removed (codon3). Here we specifically investigated the phylogeny using the alignment from codon3, because previous report showed apparently decreased supporting values for the sister relationship of magnoliids and eudicots when using alignment of codon1&2 comparing to using the cds alignment[83].

For the concatenation-based phylogeny, gene alignments were concatenated as a single supermatrix, and the tree was inferred under the "PROTGAMMAAUTO" and "GTRGAMMA" model of amino acid and nucleotide substitution using RAxML v8.2.12[84]. We also constructed individual gene trees by 100 rapid bootstrapping replicates and searching for the best-scoring maximum likelihood (ML) tree in one single run (-f a option). We checked the bootstrap support (BS) values for the nodes associated with the phylogenetic relationship among monocots, eudicots, and magnoliids, and summarized the topologies with BS value greater than or equal to 0%, 10%, 50%, or 80%, respectively. Coalescent-based phylogeny was inferred from the individual ML gene trees with different BS cutoff values using ASTRAL-II v5.5.11[85]. The reliability of each internal branch in the coalescent-based species tree was further evaluated using the local posterior probability (LPP) measurement. In addition, we also used another coalescent-based phylogenetic method, MP-EST (https://github.com/lliu1871/mp-est), to carry out additional phylogenetic analyses.

We selected 35,944 OGs with more than 4 members from OrthoMCL results, and used the above method to construct the gene tree. ASTRAL-Pro (https://github.com/chaoszhang/A-pro) and STAG (https://github.com/davidemms/STAG) method were used for species trees estimated from multi-copy gene families.

**Assessing conflict at specific nodes.** To investigate the extent of incongruence that is present in the phylogenomic data matrix, we performed the following two assessments for ML trees based on amino acid and nucleotide sequences, respectively. First, we used phyparts v0.0.1[86] to count the number of genes supporting certain topologies. Secondly, we used built-in LPPs of ASTRAL to estimate branch support and to test for polytomies[87,88].

**Results and Discussion**

In total, we identified 98 SSC and 535 MSC gene families from the orthogroups of the selected 22 species (Supplementary Note 2.4). For the analyses using coding sequence (cds) and amino acid sequence (pep) of the 98 SSC gene families, no individual trees from cds/pep alignments supported magnoliids sister to a clade consisting of eudicots and monocots (T1); 8% of cds trees and 4% of pep trees supported magnoliids as the sister lineage to eudicots (T2); 3% of cds trees and 2% of pep trees supported

magnoliids as sister lineage to monocots (T3); and the other trees (~90%) show no resolution (T4) (Fig. 4b and Supplementary Table 4.2). For the dataset 535 MSC gene families, 1% of the cds and pep trees supported that magnoliids are at the basal position of the angiosperms (T1); 5% of cds trees and 2% of pep trees supported magnoliids as sister lineage to eudicots (T2); 6% of cds trees and 2% of pep trees supported that magnoliids as sister lineage to monocots (T3); and the other trees (~90%) also show no resolution (T4) (Fig. 4b and Supplementary Table 4.2). In conclusion, most of the individual gene trees show weak support for relationships or no resolution regarding the phylogenetic relationship of magnoliids, monocots, and eudicots, resulting in the T4 topology (Fig. 4b and Supplementary Table 4.2).

The concatenation-based phylogenetic inferences were performed for the 98 SSC and 535 MSC gene families using nucleotide and amino acid sequences, respectively. The resulting phylogenetic trees all supported magnoliids and eudicots as sister lineages (T2; Supplementary Fig. 4.1), which is consistent with previous reports[89-91].

Then, we performed ASTRAL phylogenetic analyses using different datasets of input gene trees which have different BS cutoff values for the nodes of the magnoliids, monocots, and eudicots lineages (BS⩾0%, BS⩾10%, BS⩾50%, and BS⩾80%). Coalescent trees using all 98 SSC seem to support T2 with magnoliids and eudicots as sister groups with low LPP (T2; details in https://itol.embl.de/shared/Berlin). In addition, we found that including the low BS input trees of the 535 MSC gene families resulted in T2 topology, but with low LPP support (Supplementary Fig. 4.2a,b). Notably, if only inputting good support trees (BS⩾50% or BS⩾80%), we obtained phylogenies supporting T3 with high LPP values (Supplementary Fig. 4.2c,d).

We further performed the coalescent-based phylogenetic analyses inputting good support trees (the relevant node with BS⩾50%) of the 98 SSC and 535 MSC datasets using nucleotide and amino acid sequences respectively. The 98 SSC dataset moderately supported the T2 topology, with magnoliids sister to eudicots (Supplementary Fig. 4.3a,b). However, the 535 MSC dataset strongly supported T3, with magnoliids sister to monocots (Supplementary Fig. 4.3c,d). We used the MP-EST method to analyze the amino acid and nucleotide sequence data of 535 MSC. Since the input file of MP-EST does not support gene trees for non-binary analysis, the bootstrap of gene trees is not collapsed. These results show that the amino acid sequence support magnoliids and monocots are sister groups (T3), but the nucleotide sequence support magnoliids and eudicots are sister groups (T2) (Supplementary Fig. 4.4a,b). In addition, we use the bootstrap of RAxML to conduct bootstrap analysis on the MP-EST, and the results are consistent with the above results (Supplementary Fig. 4.4c,d).

In addition, we reconstructed phylogenies by partitioned codons employing both concatenation- and ASTRAL-based approaches. The resulted concatenation-based trees using the codon1&2 of the 98 SSC and 535 MSC gene families highly supported the magnoliids sister to eudicots (T2; Supplementary Fig. 4.5a,c), while using the codon3 resulted in low resolution and inconsistent topologies (Supplementary Fig. 4.5b,d). Coalescent analyses from codon1&2 of the 98 SSC gene families supported T2 but with moderate resolution (Supplementary Fig. 4.6a). Notably, the coalescent analyses using codon1&2 from the 535 MSC gene families suggested the T3 with full support (Supplementary Fig. 4.6c). When using codon3, both 98 SSC and 535 MSC gene families moderately and highly supported T3, respectively, while the placement of *S. moellendorffii* within the monocots seems to be incorrect (Supplementary Fig. 4.6b,d).

We also explored the impact of incomplete lineage sorting (ILS) on the phylogenomic incongruence. The quartet score values were investigated for the variations among the gene trees and species trees. If we input gene trees directly, the resulted quartet frequencies are similar for the three topologies (Supplementary Figs. 4.7b-e and Extend Data Fig. 7a-d), which seems consistent to those of previous studies[73,83]. However, if we collapse the tree nodes with BS value lower than 50%, the gene tree quartet frequencies of the 98 SSC gene families provide support for T2 with magnoliids as sister lineage to eudicots (Supplementary Fig. 4.7f-i and Supplementary Table 4.3). However, the quartet frequency analyses of the 535 MSC gene families with collapsed tree nodes support the T3 topology with magnoliids sister to monocots (Extended Data Fig. 7e-h and Supplementary Table 4.3). In addition, we used the SSC and MSC OGs for polytomy testing, which could not reject the null hypothesis that the node of magnoliids, eudicots, and monocots is a polytomy (T4) (Fig. 4 and Supplementary Table 4.3).

SSC/MSC gene families greatly limit the availability of genomic data. In order to use more genomic data, we used multicopy gene tree summary methods ASTRAL-Pro and STAG to analyze the 22,563 OGs of 22 species (gene family members greater than 4). These results show that ASTRAL-Pro and STAG both support magnoliids and eudicots as sister groups (T2), but the support value of STAG is low (0.24) (Supplementary Fig. 4.8).

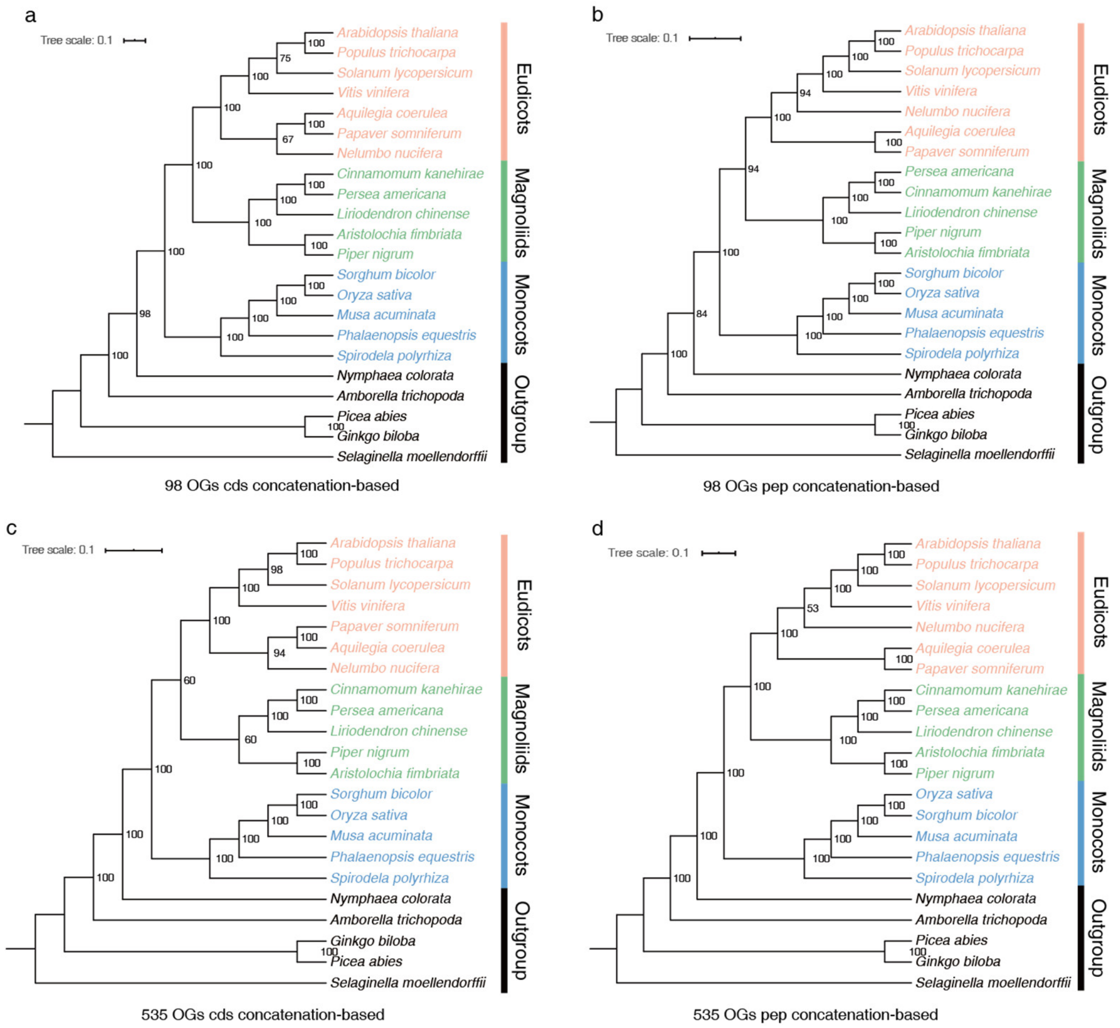


**Supplementary Fig. 4.1 | Phylogenetic relationships inferred from the 98 SSC and 535 MSC gene families using concatenation-based ML approaches.** Bootstrap support (BS) values for the concatenated-based analyses are shown on the branch to each node.

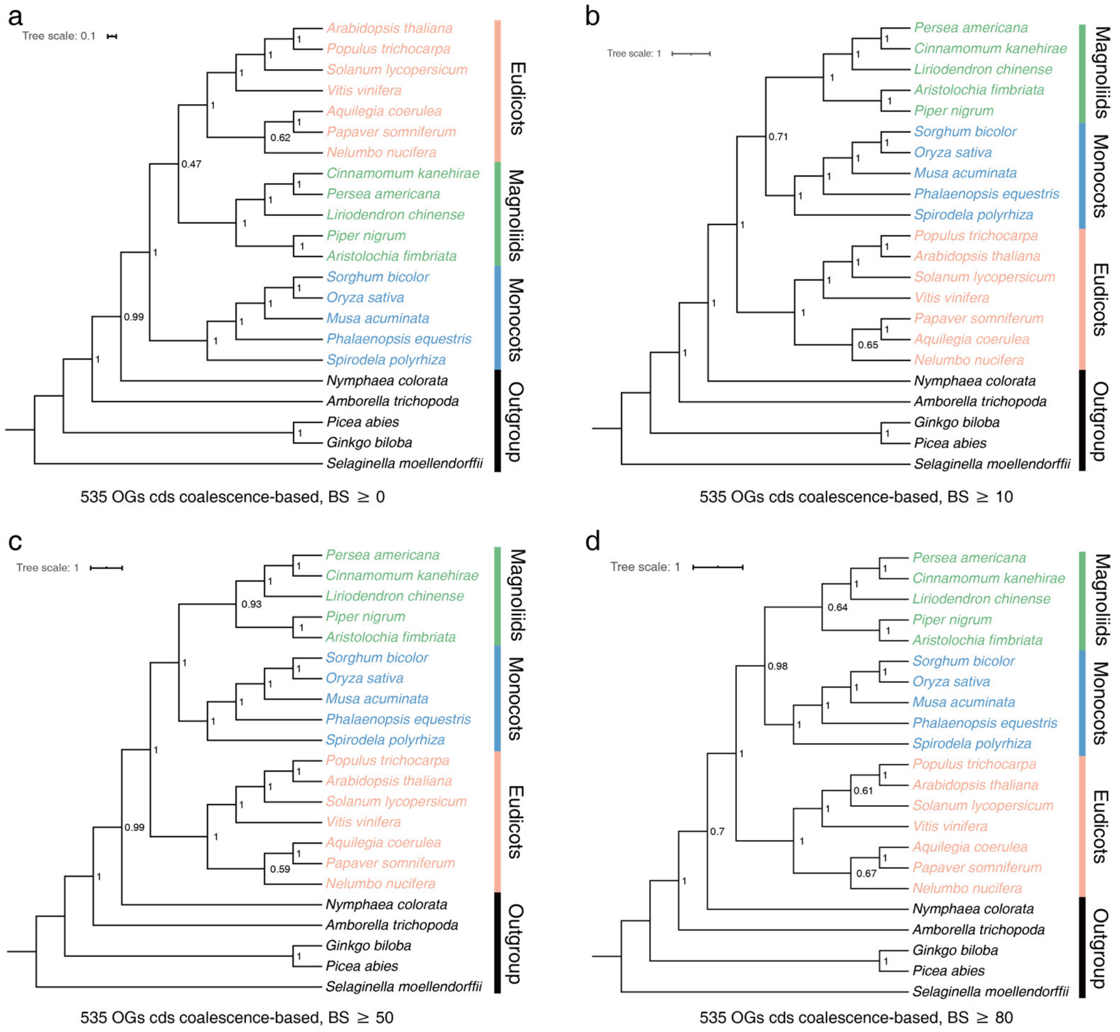


**Supplementary Fig. 4.2 | Effect of BS values of the input individual gene trees for coalescent-based phylogenetic analyses.** Numbers denote LPP values as percentages. When including weakly supported input trees, the resulting phylogenies supported T2 but with low support as in (a) and (b). When filtering out the low-BS trees, the resulting phylogenies supported T3 with high LPP values as in (c) and (d).

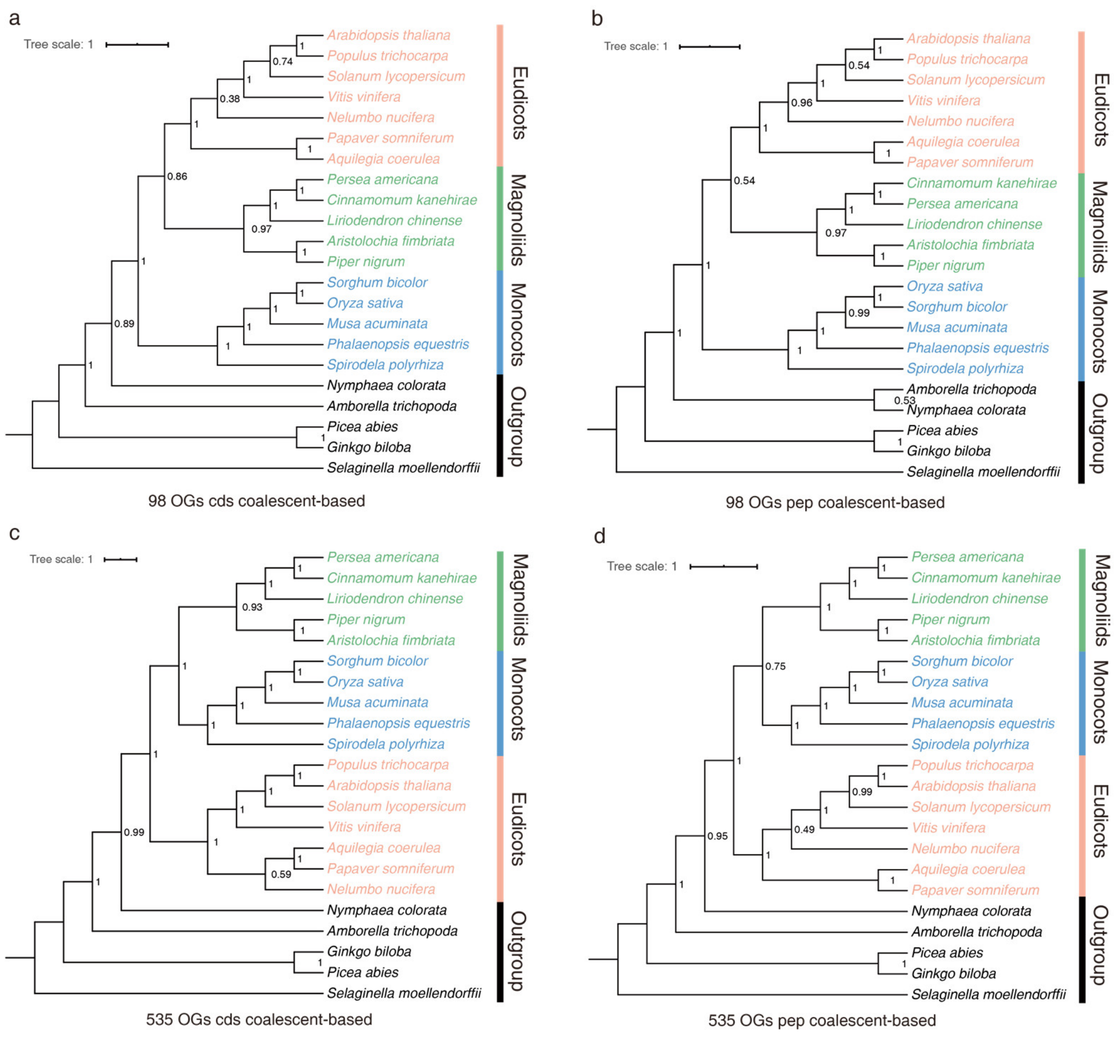


**Supplementary Fig. 4.3 | Coalescent-based inferences using well-supported (the relevant node with BS⩾50%) ML trees of the 98 SSC and 535 MSC gene families.** LPP values for the coalescent-based analyses are shown at each node.

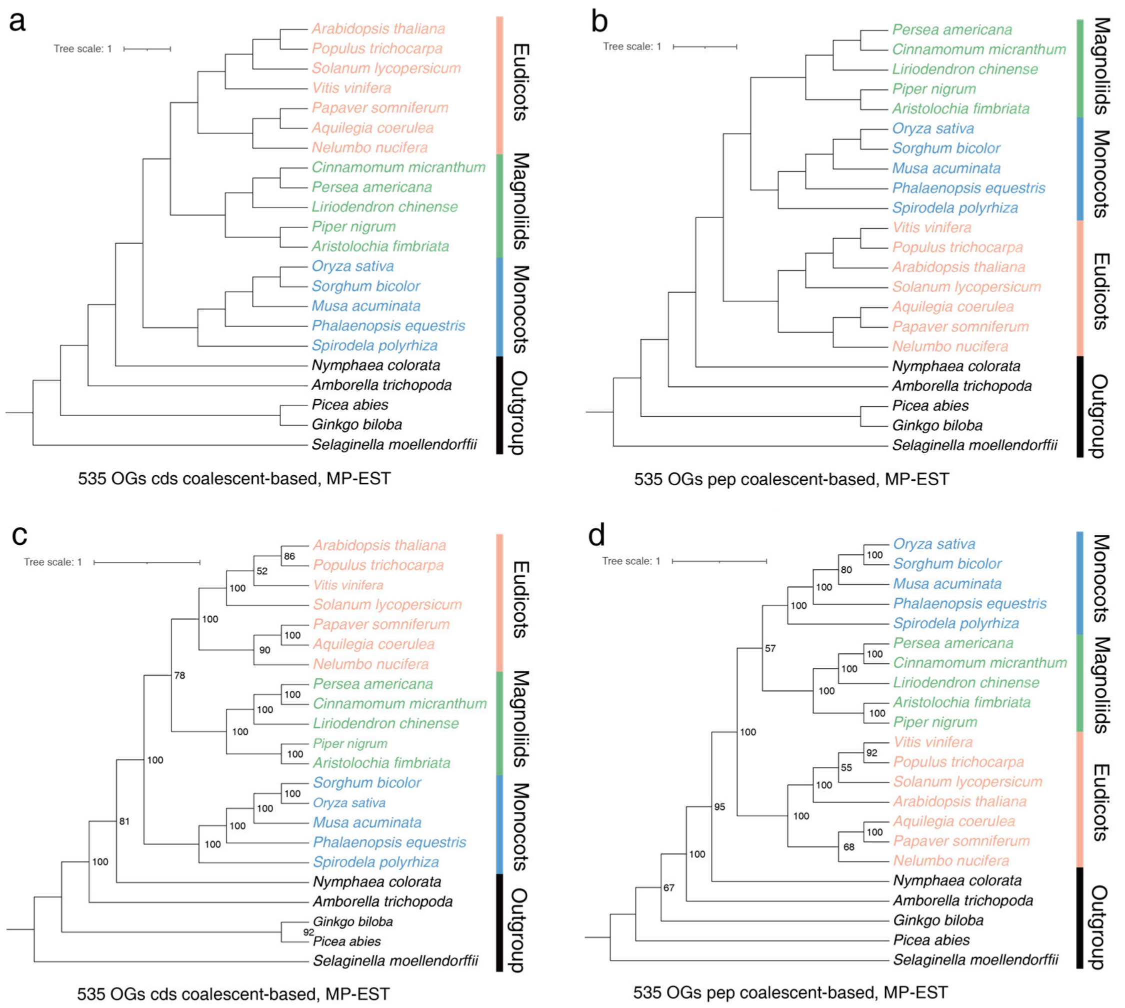


**Supplementary Fig. 4.4 | Coalescent species tree inferred by MP-EST using nucleotide and amino acid sequences of 535 genes.** BS values for the coalescent-based analyses are shown at each node in (c) and (d).

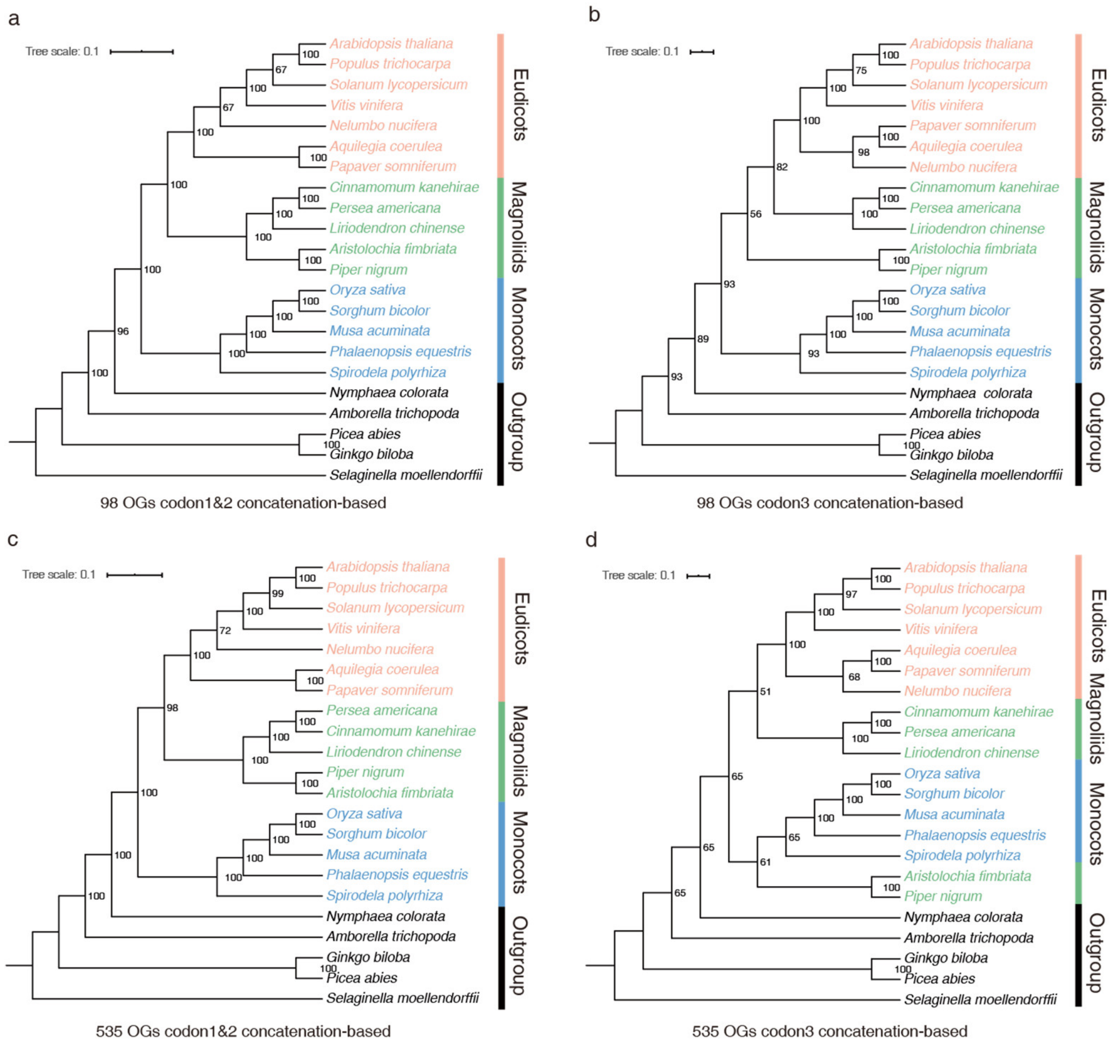


**Supplementary Fig. 4.5 | Concatenation-based phylogenetic relationships inferred from the partitioned codons.** BS values for the concatenation-based analyss are shown at each node.

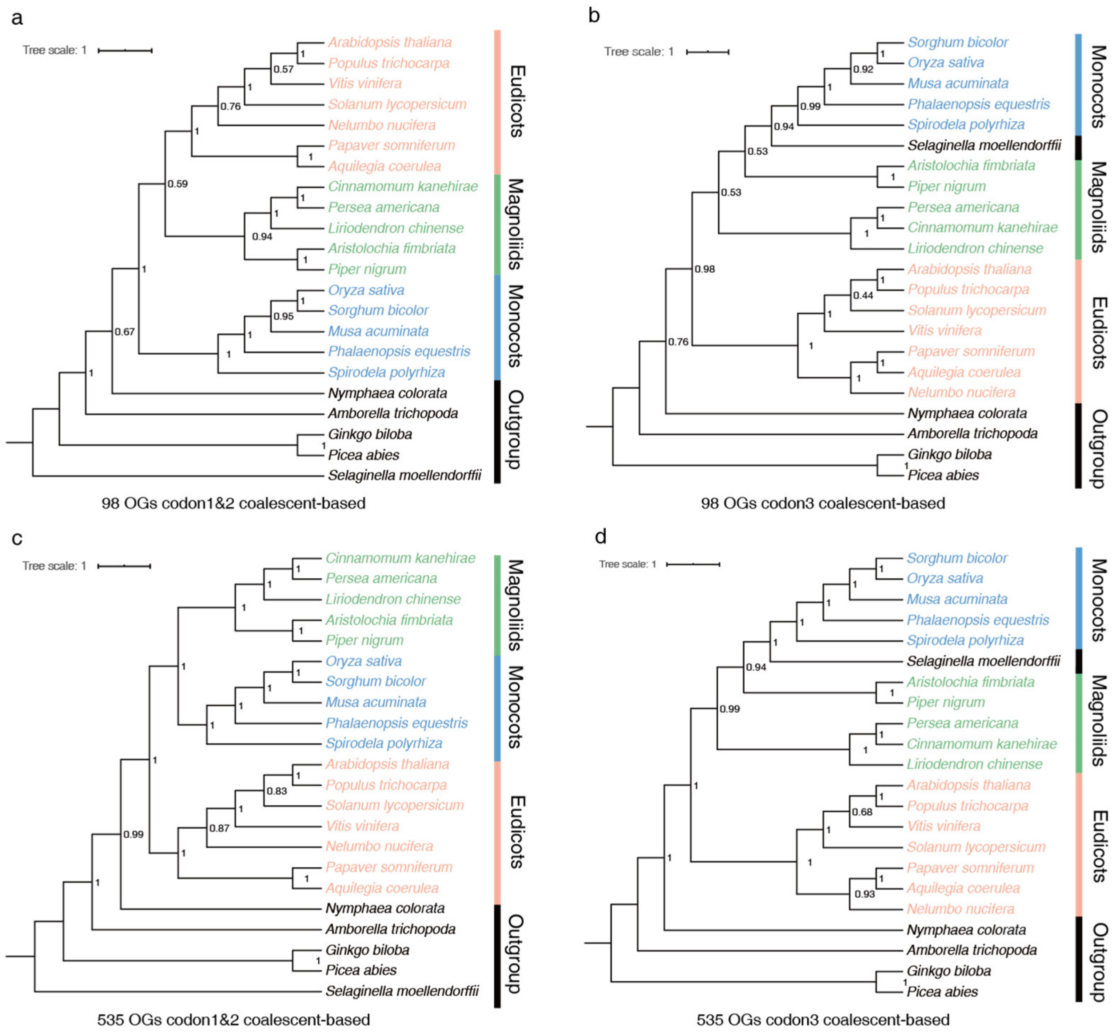


**Supplementary Fig. 4.6 | Coalescent-based phylogenetic relationships inferred from partitioned codons**. LPP values are shown for each node.

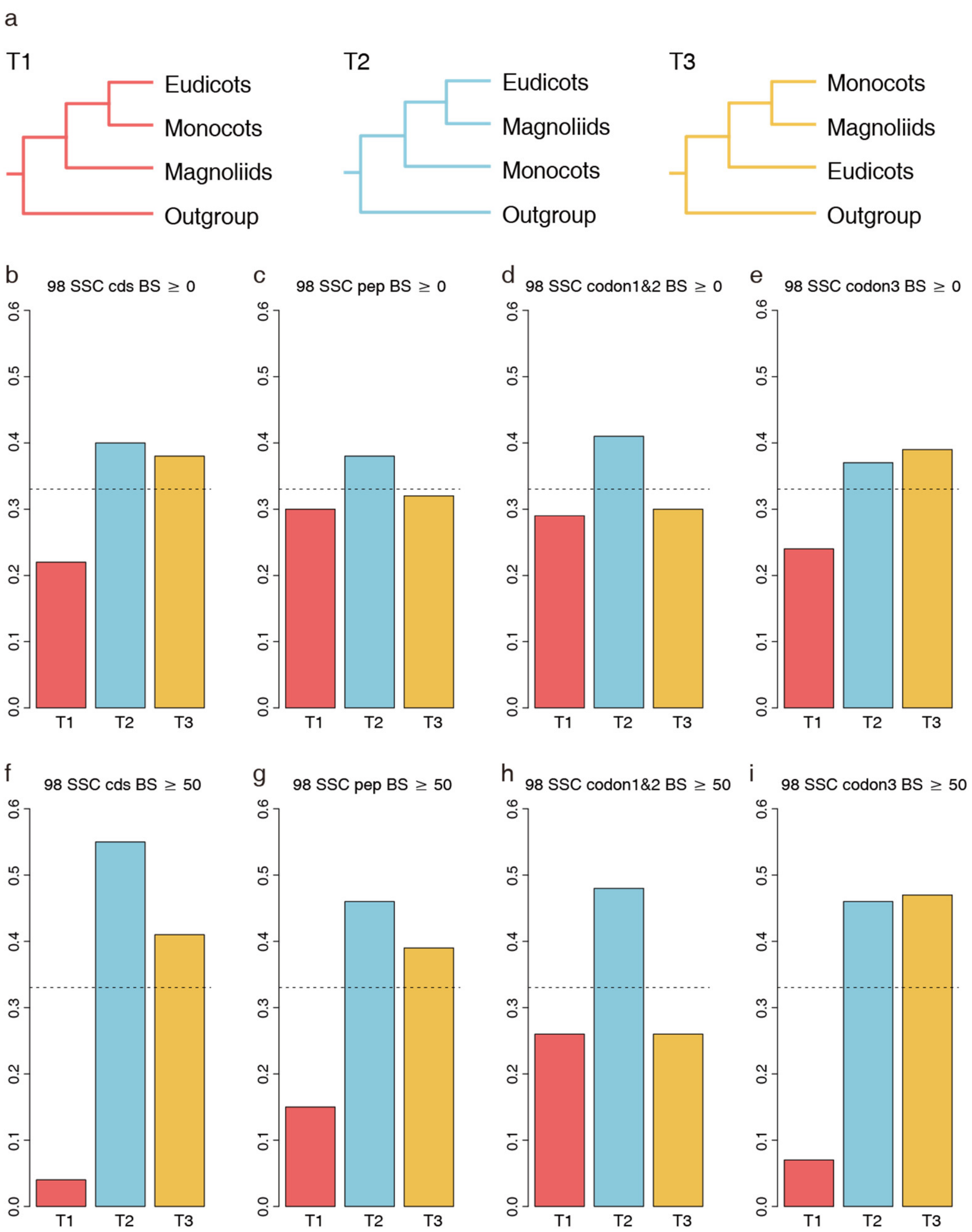


**Supplementary Fig. 4.7 | Gene tree quartet frequencies of 98 SSC gene families for different topologies.** (a) Three main topologies of magnoliids, monocots, and eudicots in angiosperms. Here we inputted individual genes trees (b-e), and also ran with collapsed trees if BS was less than 50% (f-i). The x-axis labels T1, T2, and T3 refer to the quartet support for the topologies of T1 (red), T2 (blue), and T3 (yellow) in panel (a), respectively. The dashed line refers to a proportion of 0.33.

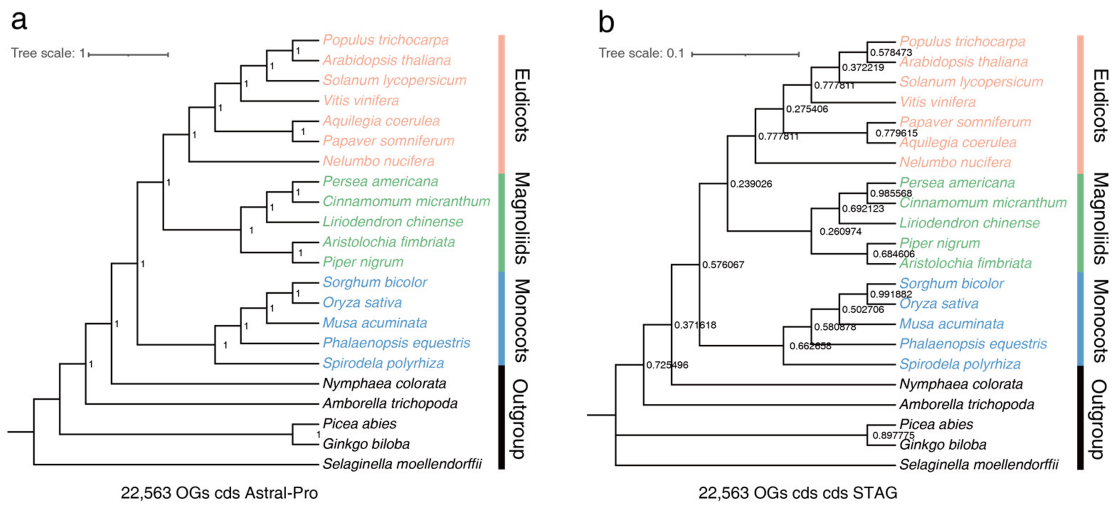


**Supplementary Fig. 4.8 | Species tree inferred by ASTRAL-Pro and STAG method using all gene family trees.** LPP and support values for the ASTRAL-Pro and STAG analysis are shown at each node.

## 4.2 The impact of taxon sampling on the resulting phylogenetic topology

### Materials and Methods

To investigate the impact of taxon sampling on phylogenomic analyses, we constructed datasets of (i) four species including one eudicot (*A. thaliana*), one monocot (*O. sativa*), one angiosperm from the ANA grade (*A. trichopoda*), and one of three magnoliid taxa (*L. chinense*, *C. kanehirae*, *A. fimbriata*), (ii) six species including *A. thaliana*, *O. sativa*, *A. trichopoda*, and three magnoliid plants (*L. chinense*, *C. kanehirae*, *A. fimbriata*), and (iii) 16 species including one angiosperm from the ANA grade (*A. trichopoda*), seven eudicots (*A. thaliana*, *P. trichocarpa*, *S. lycopersicum*, *V. vinifera*, *N. nucifera*, *A. coerulea*, and *P. somniferum*), five monocots (*O. sativa*, *S. bicolor*, *M. acuminata*, *P. equestris*, *S. polyrhiza*), and three magnoliids (*L. chinense*, *C. kanehirae*, *A. fimbriata*). Associated single-copy gene families were extracted from OrthoMCL results by custom Python scripts, and the concatenation- and coalescent-based phylogenetic analyses were performed. All of these analyses were rooted with *Amborella*, which has been well-supported as the single living sister lineage to all other extant angiosperms[55-59].

### Results and Discussion

We identified 4,041 SSC gene families for phylogenetic analysis of the four-species dataset and found that approximately 40% (1,614 out of 4,041) of the individual trees support the T1 topology with BS values greater than 50%, and 22%, 21%, and 18% of the 4,041 individual gene trees supported T2, T3, and T4, respectively (Fig. 4b and

Supplementary Table 4.2). The concatenation- and coalescent-based (BS⩾50) phylogenetic analyses all supported magnoliids as the sister lineage to the clade of eudicots and monocots (Supplementary Fig. 4.9a,b). When replacing *A. fimbriata* with the other two magnoliids, *C. kanehirae* and *L. chinense*, we obtained similar results (Fig. 4b, Supplementary Fig. 4.9c-f and Supplementary Table 4.2). For the six-species dataset, we obtained 2,673 SSC gene families, and found that both concatenation- and coalescent-based phylogenies strongly support the T1 topology as well (Supplementary Fig. 4.9g,h). When we selected 16 species, we only found 513 SSC gene families. The concatenation-based analyses fully supported T2 (Supplementary Fig. 4.10a), while the coalescent-based phylogeny supported T3 with high LPP (Supplementary Fig. 4.10b).

It is generally a tradeoff between the number of species and the number of SSC gene families for phylogenomic investigation of the species tree. A large number of the SSC gene families might better represent the genome-scale divergence, while limited taxon sampling may yield incorrect relationships, due to long-branch attraction and other artifacts. In addition, alternative phylogenetic approaches may produce different topologies. Thus, our study suggests that the discordant topologies among eudicots, monocots, and magnoliids proposed by previous studies result, at least in part, from different taxon sampling strategies and phylogenetic approaches (Supplementary Table 4.4).

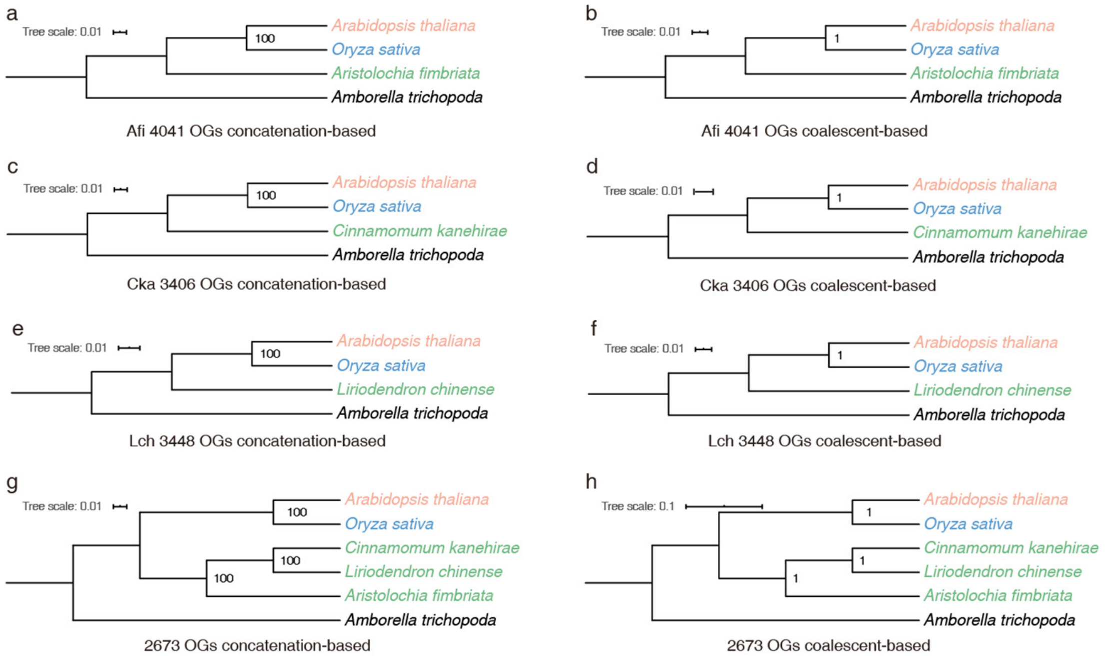


**Supplementary Fig. 4.9 | Alternative topologies resulting from the four- and six-species datasets**. The number of corresponding orthogroups (OGs) and the phylogenetic approaches are shown under the trees. Concatenation- and coalescent-based approaches were employed for phylogenomic analysis. The resulting topologies

all supported the magnoliids as sister to a clade of eudicots and monocots. *A. fimbriata* (Afi), *C. kanehirae* (Cka), *L. chinense* (Lch).

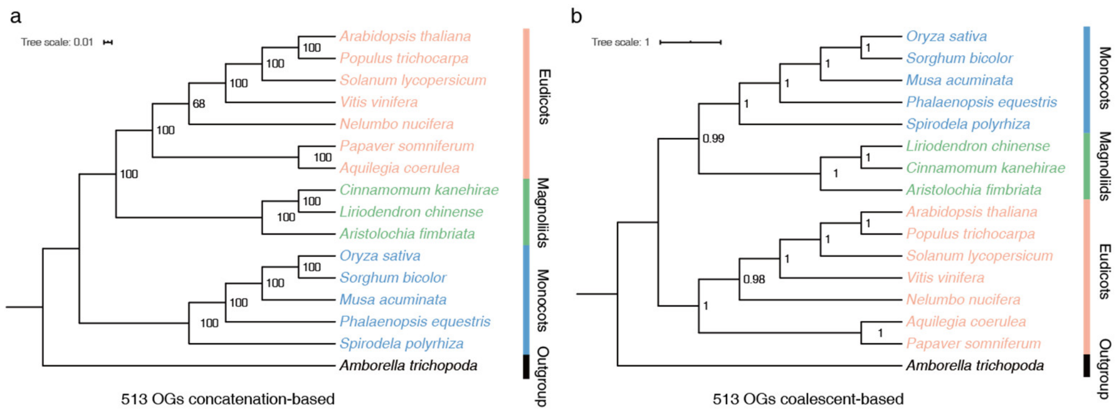


**Supplementary Fig. 4.10 | Discordant topologies from concatenation- and coalescent-based approaches for the selected 16-species dataset**. The number of corresponding OGs and phylogenetic approaches are shown under the trees.

### 4.3 Phylogenetic analysis of chloroplast genes

**Materials and Methods**

We obtained the well-annotated chloroplast genome of *A. fimbriata*, and also downloaded the chloroplast genomes of the other 21 land plants used for the gene family analyses in Supplementary Note 2.4 from NCBI. Together, we used the same set of 22 species in the above nuclear genome phylogenomic analyses as in Supplementary Note 4.1. Here, we used the chloroplast genome of *Nuphar advena* to represent Nymphaeales instead of *N. colorata*, because the chloroplast genome of *N. colorata* has not been fully annotated[59]. We manually checked the chloroplast genomes and extracted 79 protein-coding genes from the selected genomes. After concatenating the 79 gene alignments into a supermatrix, the ML tree was constructed using RAxML v8.2.12 under the “PROTGAMMAAUTO” and “GTRGAMMA” model for amino acid and nucleotide substitution, respectively, with 1000 bootstrap replicates[84]. Single gene trees were also constructed. In addition, we also used the first and second codon (codon1&2) and the third codon (codon3) for the above analyses. Even though coalescent methods are not really appropriate for plastid genomes because they are uniparentally inherited, as discussed in Stull et al. 2020[92]. We still performed the coalescent-based phylogeny inferred from the individual ML gene trees with BS$\geq$50% using ASTRAL-II v5.5.11[85].

**Results and Discussion**

Among the 79 chloroplast gene families, 7, 2, 2, and 68 gene trees supported T1, T2, T3, and T4, respectively (Fig. 4b and Supplementary Tables 4.2 and 4.3), therefore, the majority of the individual trees also show weak or no resolution about the phylogenetic relationship of monocots, eudicots, and magnoliids. Concatenation- and coalescent-based analyses of nucleotide sequences of the 79 protein-coding genes mostly supported magnoliids as the sister clade to the highly supported clade of eudicots and monocots (T1; Supplementary Fig. 4.11a-c), which is consistent with previous reports (Supplementary Table 4.4)[93,94]. The coalescent-based tree using amino acid sequences placed magnoliids as sister to eudicots with 40% LPP (T2; Supplementary Fig. 4.11d). We also performed concatenation- and coalescent-based analyses on 79 chloroplast genes for codon1&2 and codon3 sequences, respectively. Surprisingly, we found poor support for alternative relationships among monocots, eudicots, and magnoliids based on concatenation- and coalescent-based analyses for codon1&2 (Supplementary Fig. 4.11e,f). However, both codon3-based analyses highly supported magnoliids as sister to the clade of monocots and eudicots (T1; Supplementary Fig. 4.11g,h). These results seem to be consistent with the previous study using nuclear genomes that removed codon3 and decreased the support value of magnoliids + eudicots[83]. We speculate that this could be due to the biased codon usage among the different groups of angiosperms (Discussed more below).

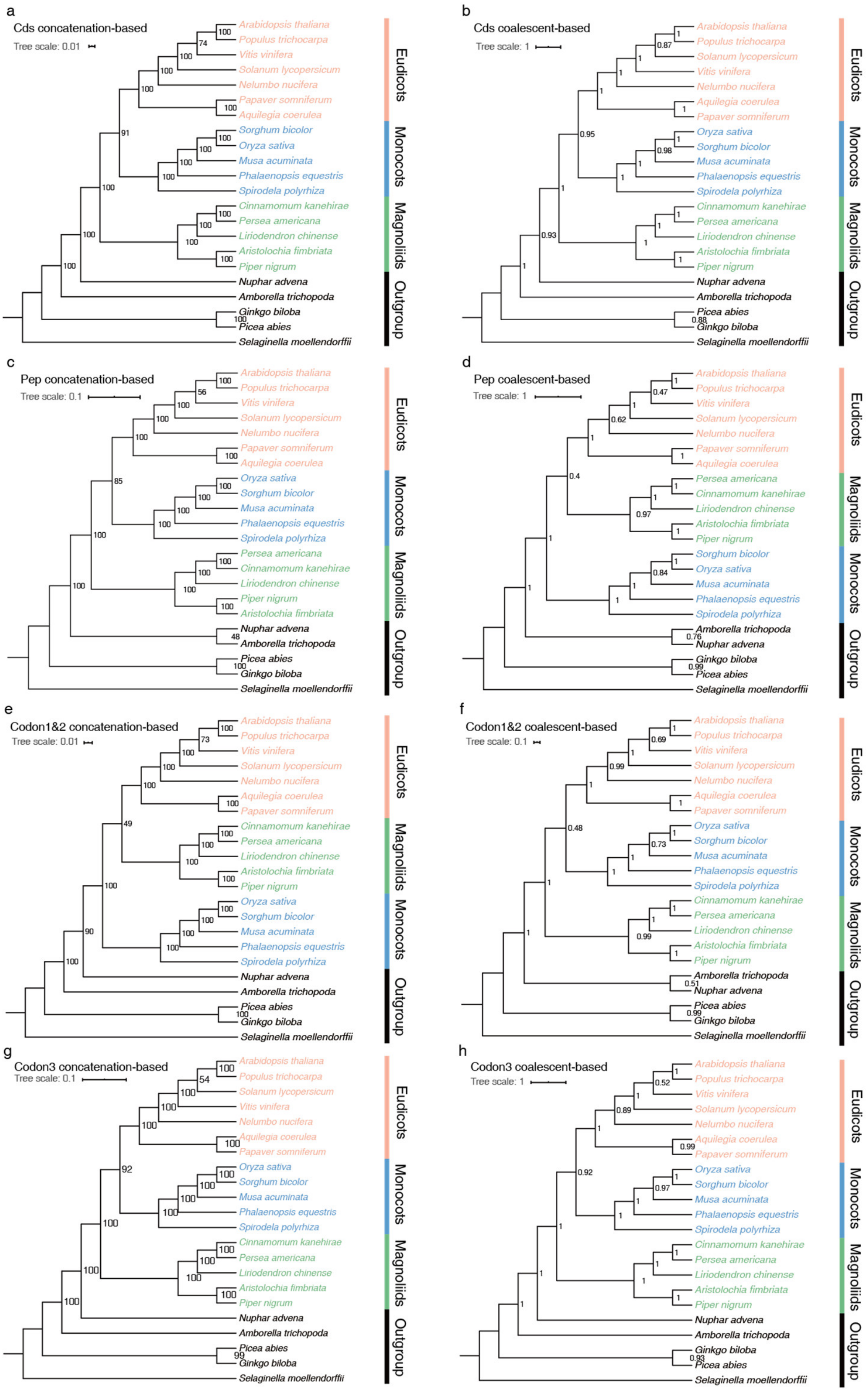


**Supplementary Fig. 4.11 | Phylogenetic relationships inferred from 79 chloroplast protein-coding genes using concatenated- and coalescent-based ML approaches.** BS and LPP values for the concatenated- and coalescent-based analysis, respectively, are shown on the branch to each node.

## 4.4 Codon usage bias and the most plausible phylogenetic topology

### Materials and Methods

The phenomenon that synonymous codons are not used at equal frequencies in coding sequences in many organisms is referred as codon usage bias. To investigate the codon usage patterns in the 22 species included in our phylogenetic analyses, we performed codon usage analyses for all of nuclear and chloroplast genes in the genome, and the MSC nuclear genes and chloroplast genes used for phylogenetic analyses, respectively. First, gene sequences with an internal stop codon in the reading frame were removed. Then, using CodonW v1.4.2 (http://codonw.sourceforge.net) with parameters of "-totals -all_indices", three indices of codon usage bias – i.e., relative synonymous codon usage (RSCU), effective number of codons (ENC), and the frequency of the nucleotides G+C at the third position (GC3s) – were calculated. Two codon composition indices, GC content of the entire gene (GC) and the frequency of each individual base at the third position of codons (A3s, T3s, G3s, C3s), were also tabulated. Differences in codon usage patterns among these species were shown using pheatmap package in R (www.R-project.org).

### Results and Discussion

The nuclear genomes of four species (*O. sativa*, *S. bicolor*, *S. polyrhiza* and *S. moellendorffii*) exhibited different codon usage patterns from the remaining 18 genomes included here. These four species tend to use purine-rich codons more frequently than pyrimidine-rich codons, and they also have relatively higher GC and GC3s content compared to other species (Supplementary Fig. 4.12a-c). Consistently, the RSCU-based cluster analysis also revealed that the preference of synonymous codon usage in these four species is different from that in the other analyzed species (Supplementary Fig. 4.12d). This might explain the unexpected placement of *S. moellendorffii* in some of the phylogenetic analyses (Supplementary Fig. 4.6b,d). We found less variation among species in codon usage in chloroplast genes than in nuclear genes. Only *S. moellendorffii* shows different codon usage than the other 21 species in chloroplast genes (Supplementary Fig. 4.13), consistent with at least some previous analyses[95].

We therefore removed *S. moellendorffii*, *O. sativa*, *S. bicolor*, and *S. polyrhiza* and performed the phylogenetic analyses of the 535 MSC nuclear gene families using concatenation- and coalescent-based approaches. The concatenation-based phylogenies still supported T2 with magnoliids sister to eudicots (Supplementary Fig. 4.14a,c). However, the coalescent analyses using cds, codon1&2, and codon3 sequences highly supported T3, with magnoliids as sister lineage to monocots (Supplementary Fig. 4.14b,d,f).

We also excluded the four codon-biased species (*S. moellendorffii*, *O. sativa*, *S. bicolor* and *S. polyrhiza*) in the analyses of chloroplast genes. Both the concatenation- and coalescent-based analyses using cds or codon3 seem to high/good supported T1 (Supplementary Fig. 4.15a,b,e,f). However, the concatenation- and coalescent-based analyses of the codon1&2 data produced different topologies, but with weak BS/LPP support (Supplementary Fig. 4.15c,d).

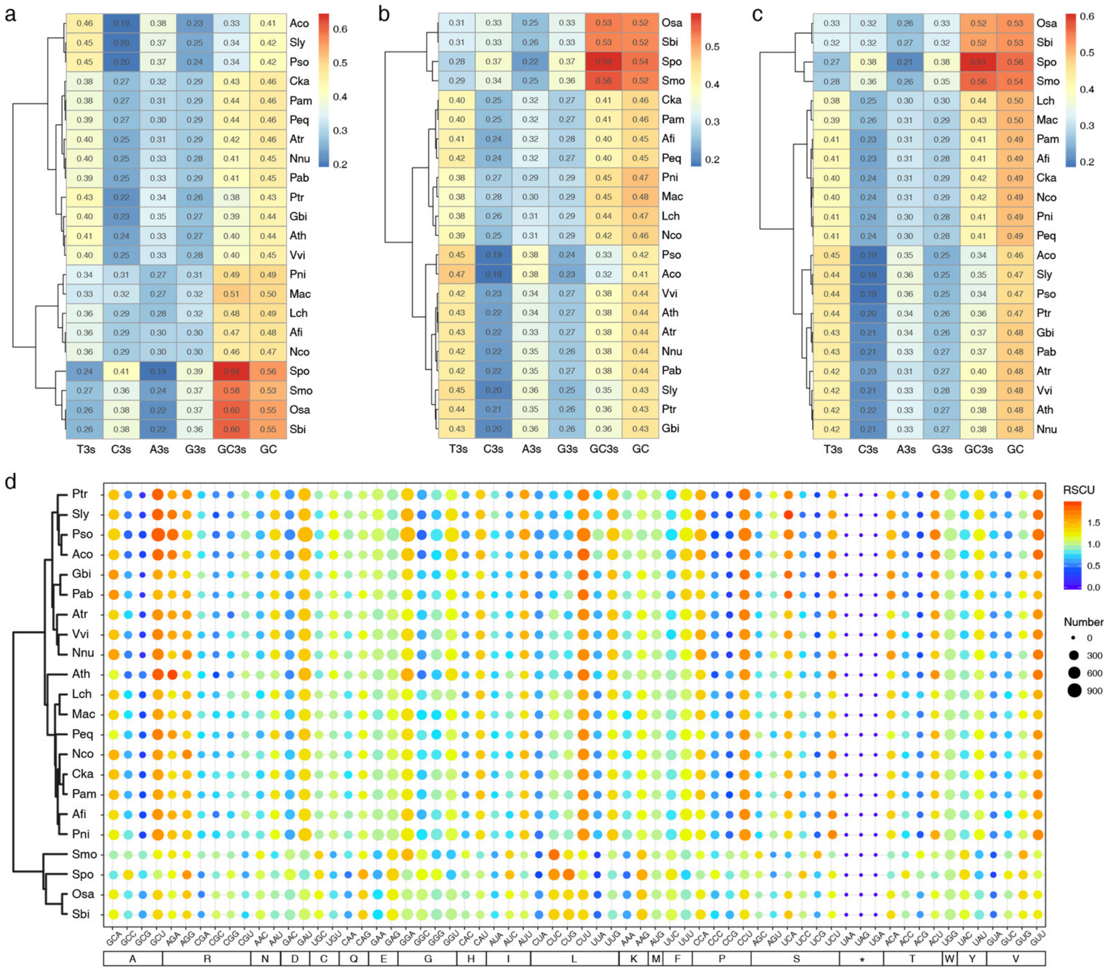


**Supplementary Fig. 4.12 | Codon composition and usage bias of nuclear genes in the 22 analyzed species.** (a) Comparisons of the third codon composition, GC3s, and GC content of genome-level nuclear genes. (b) Comparisons of the third codon composition, GC3s, and GC content of the mostly single-copy (MSC) gene families used for phylogenetic analyses. (c) Comparisons of the third codon composition, GC3s, and GC content of the codons of MSC genes which encode the same amino acids in the 22 species. (d) Cluster analysis of the analyzed 22 species based on the RSCU for the codons of MSC genes which encode the same amino acids in the 22 species. Numbers refer to the number of codons used for each species. *A. thaliana* (Ath), *P. trichocarpa* (Ptr), *S. lycopersicum* (Sly), *V. vinifera* (Vvi), *P. somniferum* (Pso), *A. coerulea* (Aco), *N. nucifera* (Nnu), *O. sativa* (Osa), *P. equestris* (Peq), *S. polyrhiza* (Spo), *S. bicolor* (Sbi), *M. acuminata* (Mac), *A. fimbriata* (Afi), *P. nigrum*

(Pni), *P. americana* (Pam), *C. kanehirae* (Cka), *L. chinense* (Lch), *N. colorata* (Nco), *A. trichopoda* (Atr), *G. biloba* (Gbi) and *S. moellendorffii* (Smo).

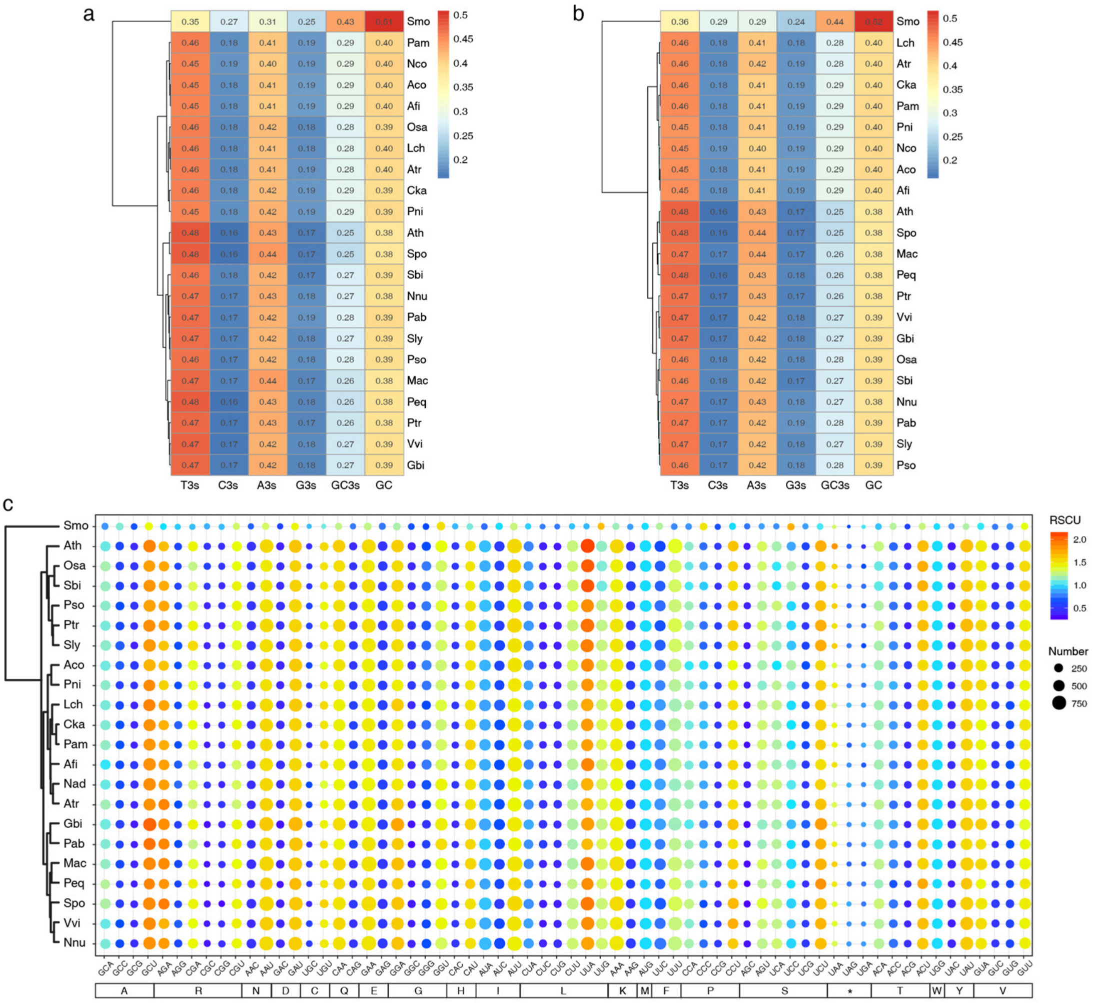


**Supplementary Fig. 4.13 | Codon composition and usage bias of the chloroplast genes in the 22 analyzed species.** (a) Comparisons of the third codon composition, GC3s, and GC content of all of the chloroplast genes. (b). Comparisons of the third codon composition, GC3s, and GC content of the 79 chloroplast gene families used for phylogenetic analyses. (c). Cluster analysis of the 22 analyzed species based on RSCU for the 79 chloroplast gene families. Numbers refer to the number of used codons in each species. *A. thaliana* (Ath), *P. trichocarpa* (Ptr), *S. lycopersicum* (Sly), *V. vinifera* (Vvi), *P. somniferum* (Pso), *A. coerulea* (Aco), *N. nucifera* (Nnu), *O. sativa* (Osa), *P. equestris* (Peq), *S. polyrhiza* (Spo), *S. bicolor* (Sbi), *M. acuminata* (Mac), *A. fimbriata* (Afi), *P. nigrum* (Pni), *P. americana* (Pam), *C. kanehirae* (Cka), *L. chinense* (Lch), *N. colorata* (Nco), *A. trichopoda* (Atr), *G. biloba* (Gbi) and *S. moellendorffii* (Smo).

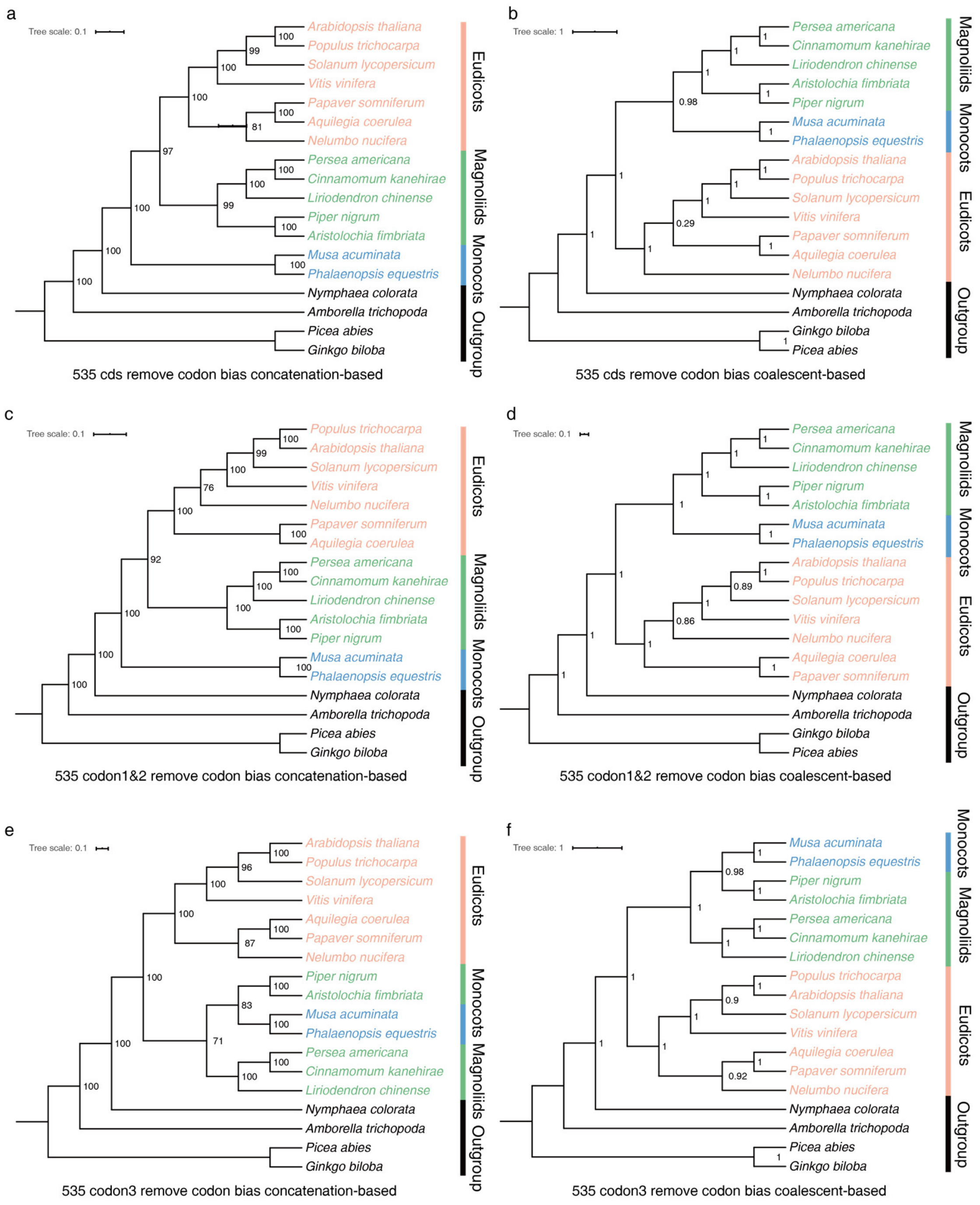


**Supplementary Fig. 4.14 | Phylogenetic relationships inferred from the 535 MSC gene families after removing the species with codon biases (*S. moellendorffii*, *O. sativa*, *S. bicolor* and *S. polyrhiza*), using concatenated- and coalescent-based approaches.** BS and LPP values for the concatenated- and coalescent-based analyses, respectively, are shown on branches leading to nodes.

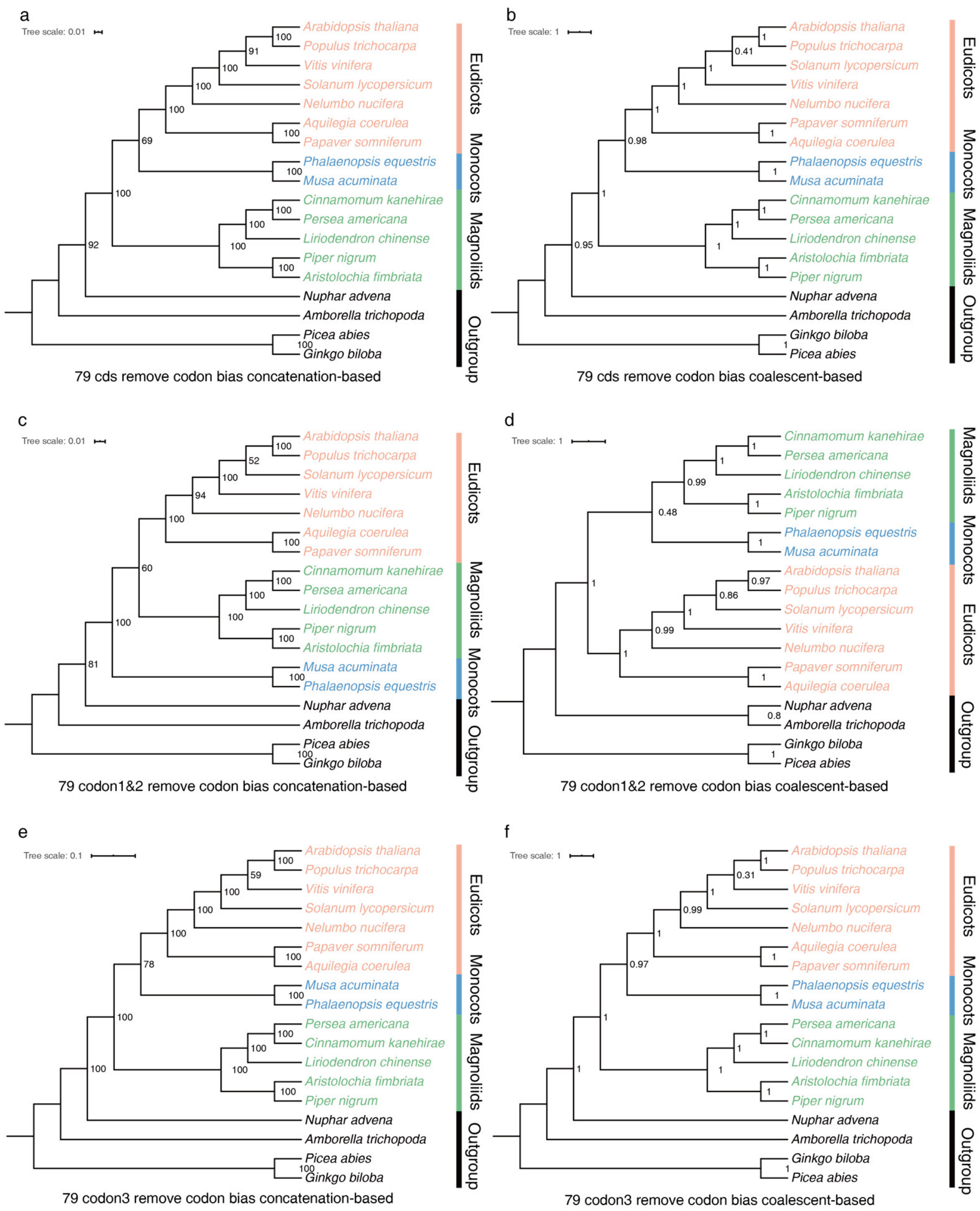


**Supplementary Fig. 4.15 | Phylogenetic relationships inferred from the 79 protein-coding chloroplast genes after removing the species with codon biases (*S. moellendorffii*, *O. sativa*, *S. bicolor* and *S. polyrhiza*), using concatenated- and coalescent-based approaches.** BS and LPP values for the concatenated- and coalescent-based analyses, respectively, are shown on branches leading to nodes.

## 4.5 Molecular dating and gene family evolution

### Materials and Methods

The Bayesian method MCMCTree in the PAML v4.9e package[96] was employed to estimate the divergence times using the T3 topology that was constructed with the 98 SSC gene families from 22 species and rooted with *S. moellendorffii* as the input tree. Following fossil dates were used for the calibration procedure: maximum age of 400 Ma for the divergence of *S. moellendorffii*[97], a minimum age of 309 Ma for the crown-group seed plants[98], a minimum age of 125 Ma for the eudicots[99], a maximum age of 113 Ma for the monocots[100-102], and a maximum age of 113 Ma for the magnoliids[103]. Branch lengths were estimated using BASEML from the PAML package under the GTR + G model (model = 7)[96]. The overall substitution rate (rgene gamma) and rate-drift parameter (sigma2 gamma) were set as G (1, 5.6) and G (1, 4.0) respectively. We ran all analyses twice to check for consistency and to ensure the effective sample size was above 200 in Tracer v1.7 (http://tree. bio.ed.ac.uk/software/tracer/).

Orthogroups constructed above (Supplementary Note 2.4) were used to investigate the expansion and contraction of gene families during evolutionary history using CAFÉ v4.1[104]. As instructed in the CAFÉ manual, we removed the 375 gene families that have more than 100 genes in each orthogroup, and the other 51,388 gene families were used for the gene family evolution analysis (automatic $\lambda$ and $\mu$ estimation, significance level for fast-evolving families 5%).

Due to the crucial roles of transcription factor (TF) for biological processes, we also specifically identified their family members in *A. fimbriata* and compared with other representative angiosperms. For each TF family, the *A. thaliana* genes were obtained from TAIR (https://www.arabidopsis.org/), and the corresponding OG containing these genes were retrieved. If multiple OGs were identified for the same TF family, we further combined them together as one family.

### Results and Discussion

Molecular dating of angiosperm lineages, using the stringent set of 98 SSC genes and with age calibrations based on 5 fossil dates, inferred the crown age of angiosperms at ~250 Ma (Fig. 4d). The split between monocots and magnoliids was estimated at ~186 Ma, and the divergence time between magnoliid + monocot clade and eudicots was at ~193 Ma. The rapid divergence among the magnoliids, monocots, and eudicots is likely responsible for the great difficulty in reconstructing relationships among these clades[56].

In addition, we found that 348 gene families expanded in the common ancestor of angiosperms, whereas 1,958 gene families contracted (Supplementary Fig. 4.16). In the common ancestor of eudicots, 942 gene families expanded, and 7,484 gene families contracted (Supplementary Fig. 4.16). In magnoliids, the black pepper genome has the largest number of expanded orthogroups (5,737), while only 567 orthogroups expanded in the *A. fimbriata* lineage. We also found that 2,380, 1,457, and 1,963 orthogroups expanded in the *L. chinense, P. americana,* and *C. kanehirae* lineages, respectively (Supplementary Fig. 4.16). *A. fimbriata* shows the least gene family expansion among magnoliids, which may be due to its lack of lineage-specific WGDs.

We found increasing numbers of TFs during land plant evolution, and the *P. somniferum*, *P. trichocarpa*, *A. thaliana*, *P. nigrum*, and *M. acuminata* genomes have larger mean family size of TF than the other species. Notably, the *A. fimbriata* genome, as well as *Amborella*, exhibit the lowest numbers of TFs among the angiosperms (Supplementary Fig. 4.17). The family sizes of TFs are also well correlated with the number and timing of WGDs.

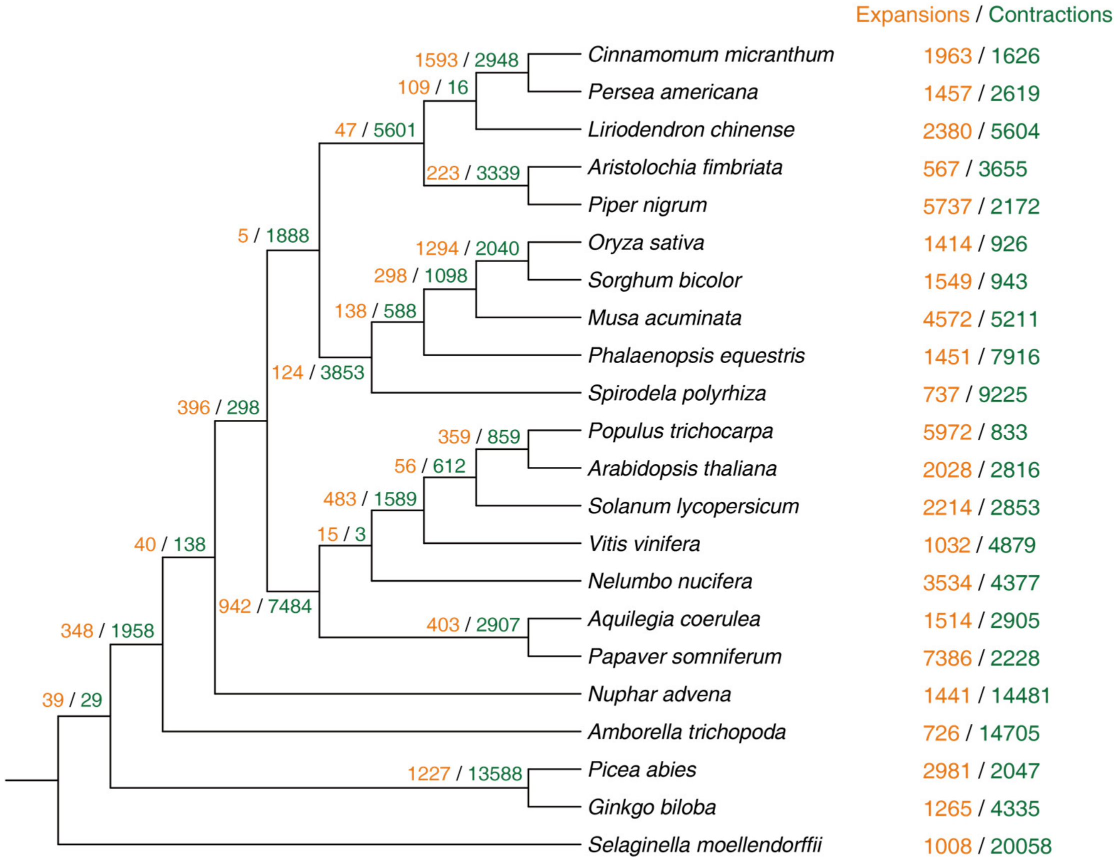


**Supplementary Fig. 4.16 | Estimation of gene family expansions and contractions**. Orange/green numbers on each branch indicate the numbers of gene family expansions and contractions, respectively.

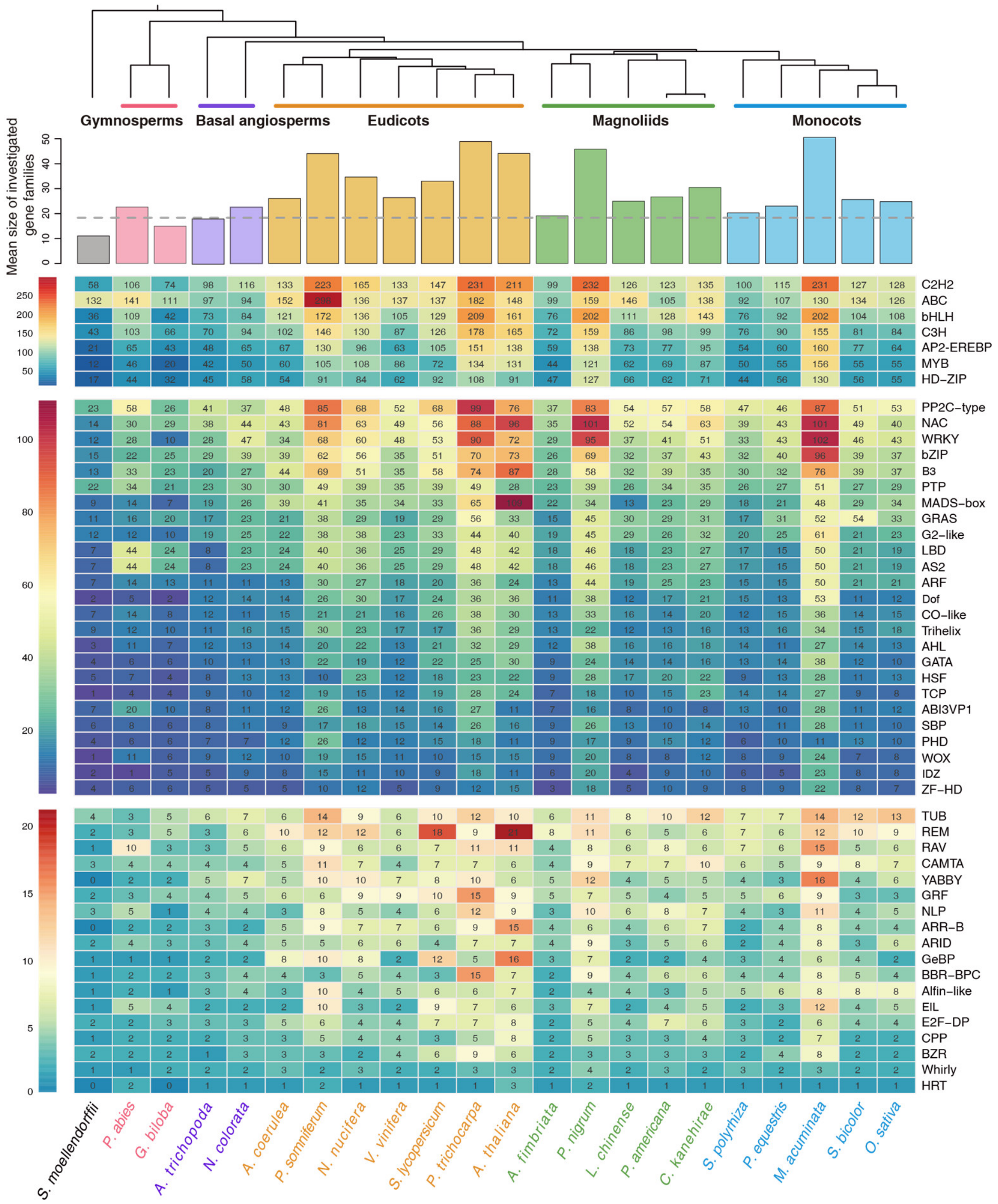


**Supplementary Fig. 4.17 | Variation in gene copy numbers of transcription factors during land plant evolution.** The *A. fimbriata* genome exhibits low mean size of gene families, similar to that of *A. trichopoda*. Transcription factors were classified into three main groups based on their total number of homologs in the 22 selected species.

## 5 High-specialized flower morphology and floral development in *A. fimbriata*

As described above, the *Aristolochia* flowers exhibits unique morphology and highly specialized modifications (Extended Data Fig. 1). In the absence of lineage-specific

WGDs, the newly sequenced genome of *A. fimbriata* offers an opportunity to study the genetic basis underlying the flower development and modifications in contrast to other angiosperms. It might shed new insights on the evolution of floral genes and regulatory networks that ultimately help us to understand the developmental mechanisms that contribute to the highly modified floral features.

**5.1 Phylogeny of the MADS-box gene family**

MADS-box genes encode a large family of transcription factors that are involved in various developmental processes in plants, animals, and fungi[105-107]. In green plants, the MADS-box gene family has been classified into type I and type II. Type I contains Mα, Mβ, and Mγ subfamilies, while type II has been further divided into $MIKC^C$ and MIKC* groups[105,108,109]. The $MIKC^C$ genes play essential roles in the flower development and floral regulatory networks, thus they are the most widely studied MADS-box genes[108-110].

**Materials and Methods**

To identify MADS-box genes in *A. fimbriata*, we first retrieved the well-studied and annotated subtypes of MADS-box genes in *A. thaliana*[111], *O. sativa*[112]*, Amborella*[55] and *N. colorata*[59]. The above constructed orthogroups (Supplementary Note 2.4) that contain these well-annotated MADS-box genes were obtained. To avoid any missing putative homologs, we further performed the BLASTP analysis using these well-annotated MADS-box genes as queries, as well as the profile hidden Markov model (HMM) searches using SPF-TF (PF00319) from the Pfam database[113] as seed, against the genome-wide amino acid sequences in *A. fimbriata*, employing the BLASTP[52] (*E*-value < $10^{-3}$) and the hmmsearch in HMMER v3.3[114] (--domE 0.001) respectively. The identified putative MADS-box genes were further manually inspected and screened using InterProScan[115]. We also identified the MADS-box genes in three other magnoliids (*C. kanehirae*, *P. nigrum* and *L. chinense*) using the same process. Due to the large number of MADS-box gene family members, we mainly focused on these eight species representing major clades of angiosperms for phylogenetic analysis.

To construct the phylogeny of these MADS-box genes, amino acid sequences were aligned first using MAFFT v7.312[116], and the nucleotide sequences were forced to fit the amino acid alignment using PAL2NAL v14[82]. The poorly aligned regions were removed using trimAL v3[117] with parameter of "-gt 0.3". Phylogenetic tree was constructed using maximum likelihood in RAxML v8.2.12[84] under the "GTRGAMMA" model with 100 bootstrap replicates.

**Results and Discussion**

Based on the phylogeny and the well-annotated genes in the above mentioned four species, 18 type I and 17 type II MADS-box genes were identified in *A. fimbriata* (Supplementary Tables 5.1 and 5.2 and Supplementary Figs. 5.1 and 5.2). We further specifically examined the floral organ identity determination program. Surprisingly, we found that the *A. fimbriata* genome contains only one homologue for each of the eight classes of floral organ identity genes including: *APETALA1/FRUITFULL* and *AGAMOUS-LIKE6* (*AP1/FUL* and *AGL6*, A function for sepals and petals), *APETALA3* and *PISTILLATA* (*AP3* and *PI*, B function for petals and stamens), *AGAMOUS* (*AG*, C function for stamens and carpels), *SEEDSTICK* (*STK*, D function for ovule), and *SEPALLATA* (*SEP1* and *SEP2*, E function for interacting with ABCD-function proteins) (Supplementary Figs. 5.1 and 5.2 and Fig. 5b). This is likely due to the lack of any additional WGD in *A. fimbriata* since the origin of extant angiosperms. In addition, we found high conservation of gene structure and exon sequences of floral organ identity genes between *A. fimbriata* and *Amborella*, while the intron length of *Amborella* genes are generally longer than that of *A. fimbriata* orthologues (Extended Data Fig. 8).

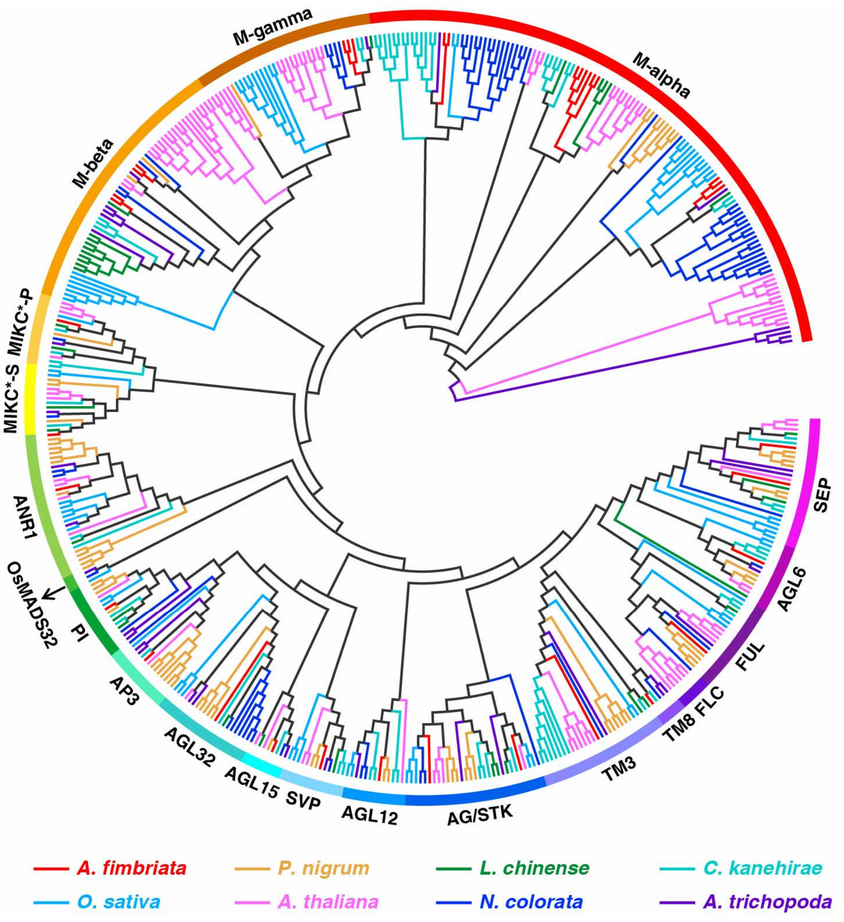


**Supplementary Fig. 5.1 | Phylogeny of MADS-box genes from the selected eight species.** The phylogenetic tree was constructed using maximum likelihood in RAxML, and the MADS-box genes are classified into 20 clades. Branches are colored according to the species color scheme under the tree.

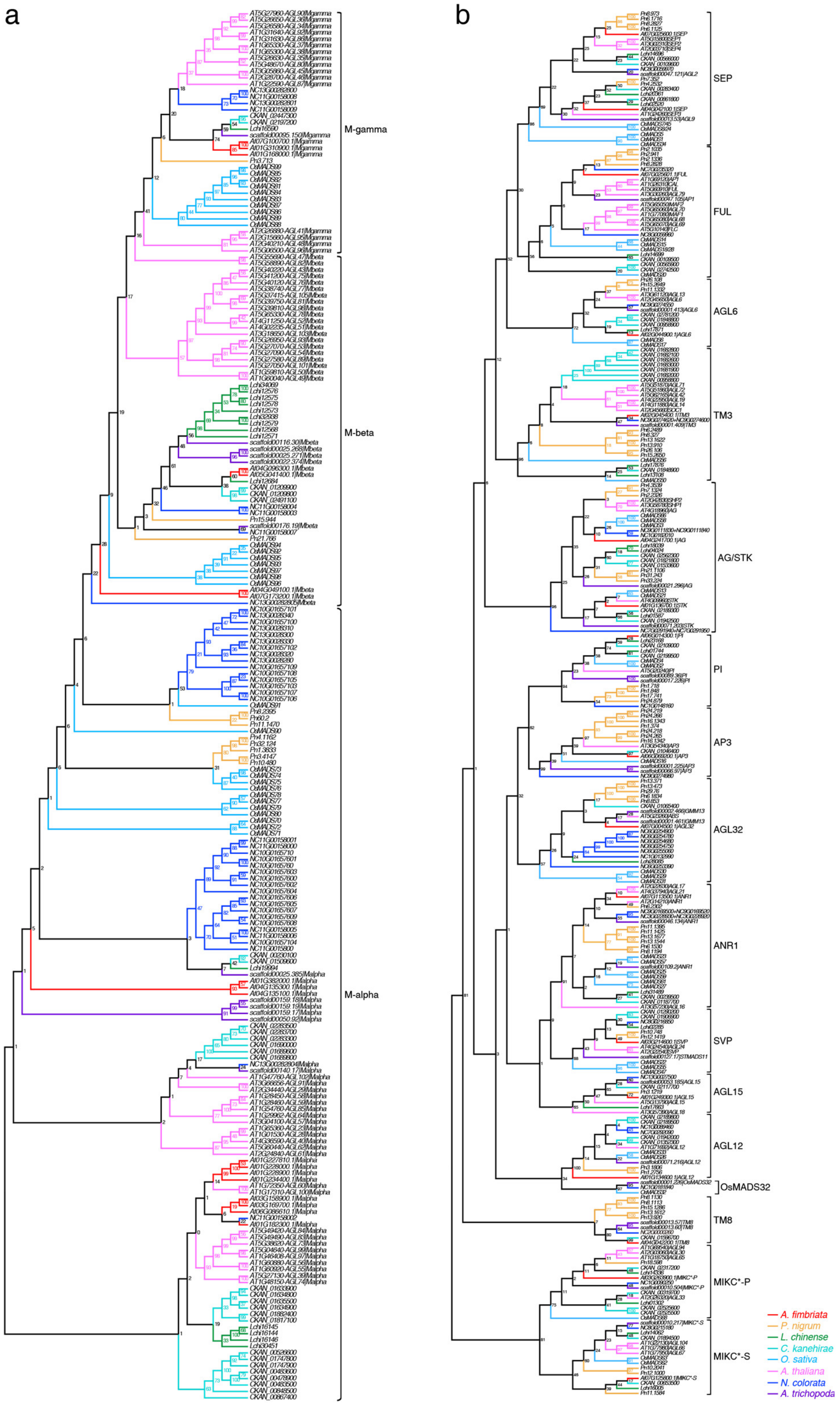


**Supplementary Fig. 5.2 | Phylogenetic trees of type I (a) and type II (b) MADS-box genes from eight species with their gene names.**

## 5.2 Alternative splicing of MADS-box genes in *A. fimbriata*

Alternative splicing (AS) is a key post-transcriptional processing step of pre-mRNA, which is prevalent in eukaryotic organisms and sometimes can widely lead to great complexity of the functional transcripts from a single gene locus[118,119]. Previous studies have illustrated that AS plays crucial roles in the processes of floral development[120], vernalization, and biotic and abiotic stress responses[121]. Given the relatively small number of MADS-box genes and other floral regulatory TFs in *A. fimbriata* (Fig. 5a), we particularly investigated AS events of MADS-box genes in *A. fimbriata*, to explore whether AS contributes to the development of the highly modified flower structure of *A. fimbriata*.

### Materials and Methods

In addition to the full-length transcript sequencing in the annotation section (Supplementary Note 2), we further collected and pooled the flower buds at different developmental stages (from stage 5 to anthesis)[122] together in relatively equal amount to perform much deeper transcriptome sequencing to get the potential AS for floral genes. The extracted RNA from the mixed sample was used for isoform sequencing (Iso-Seq) on the PacBio Sequel II platform. The raw sequence data were processed by SMRT Link v8.0 software (https://www.pacb.com/support/software-downloads/). Circular consensus sequences (CCSs) were generated from the raw subreads BAM file to identify full-length (FL) reads using ccs with parameters of "--min-passes 1 --min-length 100". Then, full-length non-chimeric (FLNC) reads were identified if they have the 5'-primer, 3'-primer, and poly(A) tail. Lastly, FLNC reads from the same isoform were clustered and further polished using subreads.

The high-quality and full-length consensus sequences were aligned to the *A. fimbriata* genome using the Genome Mapping and Alignment Program (GMAP) software[48] with parameters of "-S -A -f sampe --max-intronlength-middle 5000 -t 15". SpliceGrapher v0.2.7 software[123] was used to identify AS events in *A. fimbriata*, in which the models of splice sites were designed as donors of GT or GC and acceptor of AG, respectively.

### Results and Discussion

The quality control assessments indicated the extracted RNA was of high quality, with the RNA integrity number (RIN) = 9.7 and the 28S/18S ribosomal ratio = 1.6. The generated raw subreads data were approximately 133.09 Gb in size, and the average length of subreads was approximately 2,126 bp. After processing with SMRT Link, we obtained 132,090 high-quality and polished full-length consensus sequences, which were further mapped to the *A. fimbriata* reference genome. We found 97.8% of

all annotated genes (21,282/21,751) with at least one transcript, and all of the MADS-box genes were detected. These results indicate that the Iso-Seq data set has very high sequencing depth and a wide range of coverage.

In total, we identified 3,539 AS events from 2,678 genes in *A. fimbriata* genome, including intron retention (IR; 2,940, 83.1%), exon skipping (ES; 126, 3.6%), alternative 3' splice sites (A3SS; 280, 7.9%), and alternative 5' splice sites (A5SS; 193, 5.5%). IR was the most abundant type of AS event. Notably, we found no AS event for 33 of the 35 annotated MADS-box genes, and only two (*AG* and *AGL15*) were detected to have potential AS events (both with IR type) (Supplementary Fig. 5.3). *A. fimbriata*, with a relatively small number of MADS-box genes, might employ mechanisms other than multiple alternatively spliced isoforms to generate its complex floral morphology.

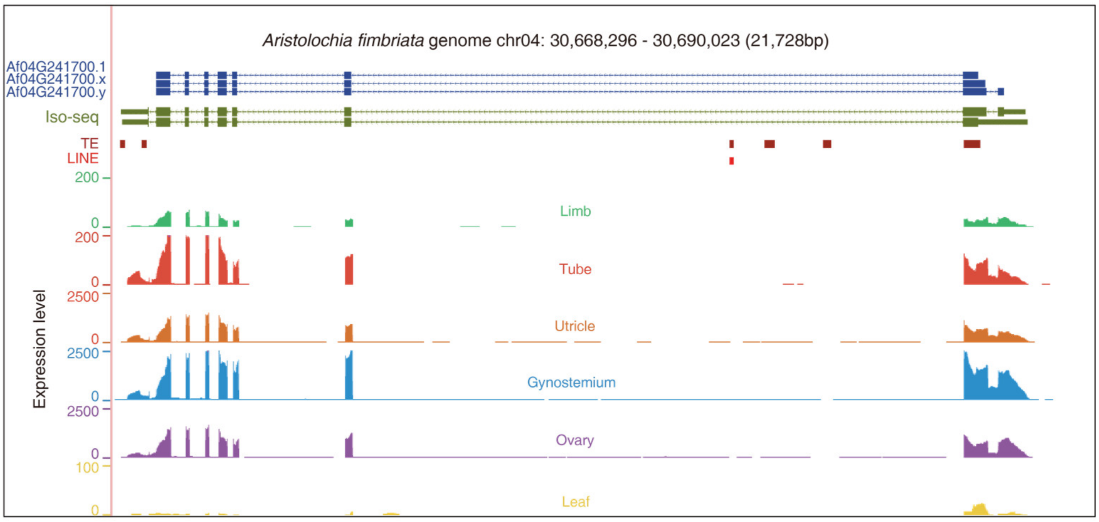


**Supplementary Fig. 5.3 | Genomic locations, overlapped TE/LINE elements, and expression levels of the *AG* gene in *A. fimbriata.***

## 5.3 Expression patterns of floral organ identity genes

### Materials and Methods

In this study, we wanted to obtain a general idea about the expression patterns of these floral organ identity genes, with the understanding that the early stage flower buds are usually used for studying the role of these genes in specifying floral organs. Here, the flowers at anthesis and late pre-anthesis (developmental stages 7 and 8) from living plants were collected and further dissected into 5 different tissue types (limb, tube, utricle, gynostemium, and ovary). Young leaves were also collected. Each sample had three biological replicates. Total RNA was extracted and sequenced on an Illumina

HiSeq4000 instrument. RNA-Seq raw reads from three replicates of the 11 samples were preprocessed using Trimmomatic[26] to remove adaptor sequences and low-quality reads with the parameters of "PE -phred33 ILLUMINACLIP: Trimmomatic-0.36/adapters/TruSeq3-PE.fa: 2:30:10 LEADING: 20 TRAILING: 20 SLIDINGWINDOW: 4:20 MINLEN: 50". The clean reads were then mapped to the reference genome using HISAT2 with default parameters. The expression abundance values were calculated using Stringtie[124] with options "-e -A", and we averaged the abundance values from the three biological replicates to obtain levels of gene expression.

**Results and Discussion**

We found that all of the floral organ identity genes exhibited relatively stable expression patterns during the pre-anthesis and anthesis stages (Fig. 5c), which were largely consistent with those of other well-studied species, such as *A. thaliana*[125]. For the A-class genes, we found that *AfFUL* has relatively low expression levels, while *AfAGL6* is highly expressed in the perianth (limb, tube, and utricle) as well as in the ovary (Fig. 5c and Supplementary Fig. 5.4). For B-class genes, *AfAP3* and *AfPI* are widely expressed in all studied floral organs, and their overlapping expression pattern in the sepal-derived perianth might be associated with the petaloidy of the perianth (Fig. 5c). The *AfAG* gene is mainly expressed in the gynostemium, ovary, and utricle (Fig. 5c and Supplementary Fig. 5.3), and *AfSTK* is highly expressed in the ovary (Fig. 5c). Despite their wide ranges of expression in the pre-anthetic and anthetic flowers compared to the more restricted patterns in *A. thaliana*[126,127], we found that the relatively high expression in specified floral organs largely agrees with their putative ascribed roles in floral organ patterning (Fig. 5c). Further expression profiling and functional studies using earlier developmental stage flowers are needed to confirm the function of these floral organ identity genes.

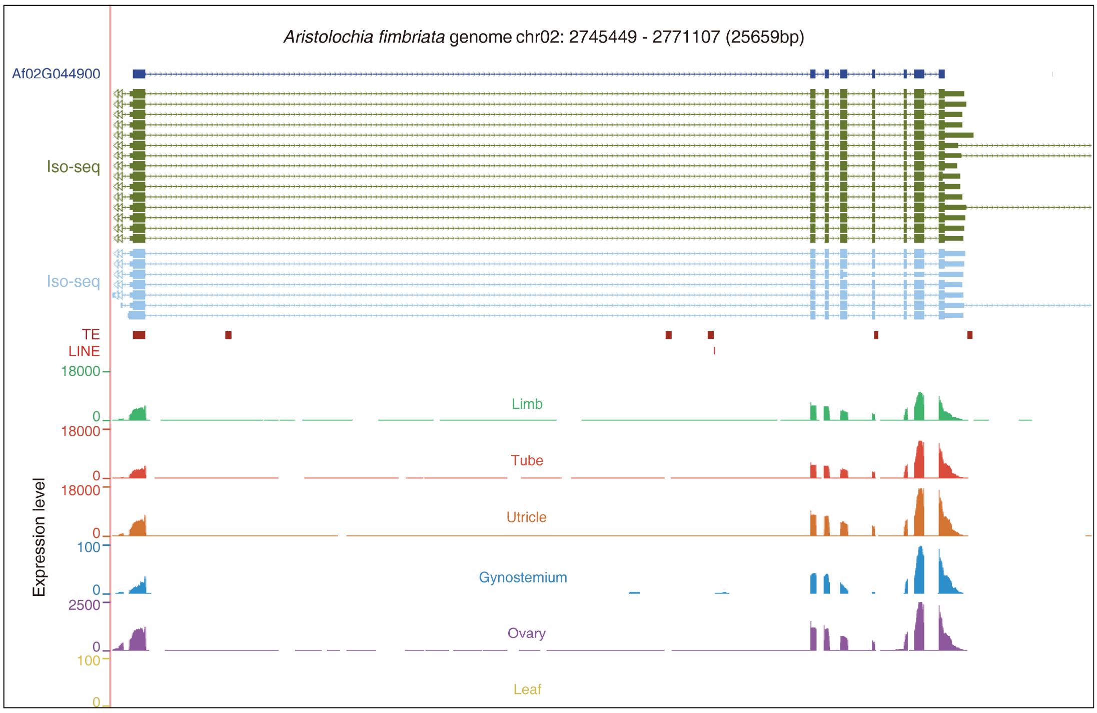


**Supplementary Fig. 5.4 | Genomic locations, overlapped TE/LINE elements, and expression levels of the *AGL6* gene in *A. fimbriata.***

## 5.4 Genetic basis of the floral organ fusion and the bilaterally symmetry of flowers

Floral organ fusion is a peculiar feature of the *A. fimbriata* flower. The stamens and stigmatic lobes are fused to form the gynostemium, and the sepals are fused to form a calyx consisting of an inflated utricle, a narrow tube, and a limb[2,5]. It has been suggested that the boundary-specification genes, *CUP-SHAPED COTYLEDON* (*CUC*) genes belonging to NAC transcription factors, are the key determination factor for the fusion of floral organs[128-130].

In addition, *Aristolochia* flowers exhibit monosymmetric perianth, which is rare in magnoliids and also rare in angiosperms in general[131,132]. It has been suggested that floral symmetry may impact specialized interactions between plants and their insect pollinators[133]. Previous studies have proposed the *CYCLOIDEA* (*CYC*)/*TEOSINTE BRANCHED1* (*TB1*) class and *CINCINNATA* (*CIN*) class of Teosinte branched1/Cycloidea/Proliferating cell factor (TCP) transcription factors as key regulators of flower symmetry establishment and morphogenesis of leaf-like organs in model species *Antirrhinum majus* and some other species[134-139].

**Materials and Methods**

The associated homologous genes for specific flower features were examined in the *A. fimbriata* genome. To identify the *CUC* genes in *A. fimbriata*, the well-annotated *CUC* genes in *A. thaliana* were firstly retrieved from previous studies[129,130] and TAIR (https://www.arabidopsis.org/). Then, similar to the identification of MADS-box genes in the above section (Supplementary Note 5.1), we integrated and screened the results from the associated OG(s), BLASTP and HMM searches to identify the *CUC* homologs in *A. fimbriata*, *A. trichopoda*, *N. colorata*, *O. sativa*, *C. kanehirae*, *P. nigrum* and *L. chinense*. Similarly, using the well-studied *CYC* and *CIN* genes in *A. majus* and *A. thaliana* as references[136-138], the *CYC* and *CIN* homologous genes were also comprehensively identified. Phylogenetic trees were then constructed using the methods described above (Supplementary Note 5.1). The expression patterns of these identified *A. fimbriata* homologous genes were also examined from the aforementioned transcriptomes in Supplementary Note 5.3.

**Results and Discussion**

Two *CUC* genes (named *AfCUC2* and *AfCUC3*) were identified in *A. fimbriata*, which are orthologous to the boundary-specification genes in *A. thaliana* (*CUC1/2* and *CUC3*, respectively) (Supplementary Fig. 5.5). Our RNA-Seq data showed that both of them exhibit nearly no expression in various floral organs and leaf tissue during both pre-anthesis and anthesis stages, except for the weak expression of *CUC2* in ovary (Fig. 5d). Given the previous knowledge of *CUC* genes in controlling the specification of organ boundaries[129], their lack of expression is consistent with the formation of tubular perianth and the fusion of stamens and the stigmatic region of carpels.

We identified five TCP genes in *A. fimbriata*. Phylogenetic analysis showed that the *CYC/TB1* clade includes one *A. fimbriata* gene (named *AfCYC*), all three *CYC* genes (*CYC1*, *CYC2*, and *CYC3*) in *A. thaliana* and all functional *CYC* genes in *A. majus*, and that three *A. fimbriata CIN* genes (named *AfCIN1*, *AfCIN2*, and *AfCIN3*) were clustered in three groups, respectively (Supplementary Fig. 5.6). The RNA-Seq data showed that the *AfCYC* gene is only expressed in the limb of the anthetic flower and in leaf (Fig. 5e). In contrast, all three *CIN* genes are widely expressed in all floral organs and the leaves, and exhibit differential expression in perianth basipetally with the highest expression in the limb region (Fig. 5e). These results, together with their previously known expression patterns at early developmental stages in *Aristolochia arborea* and *A. fimbriata*[132,135], further suggest that both *CYC* and *CIN* genes contribute to the heterogeneous growth and morphological deformation of the monosymmetric perianth in *Aristolochia*. Again, further functional genomic studies in the early flower developmental stages are needed to confirm the roles of these genes in the establishment of the bilateral symmetry of the perianth in *Aristolochia*.

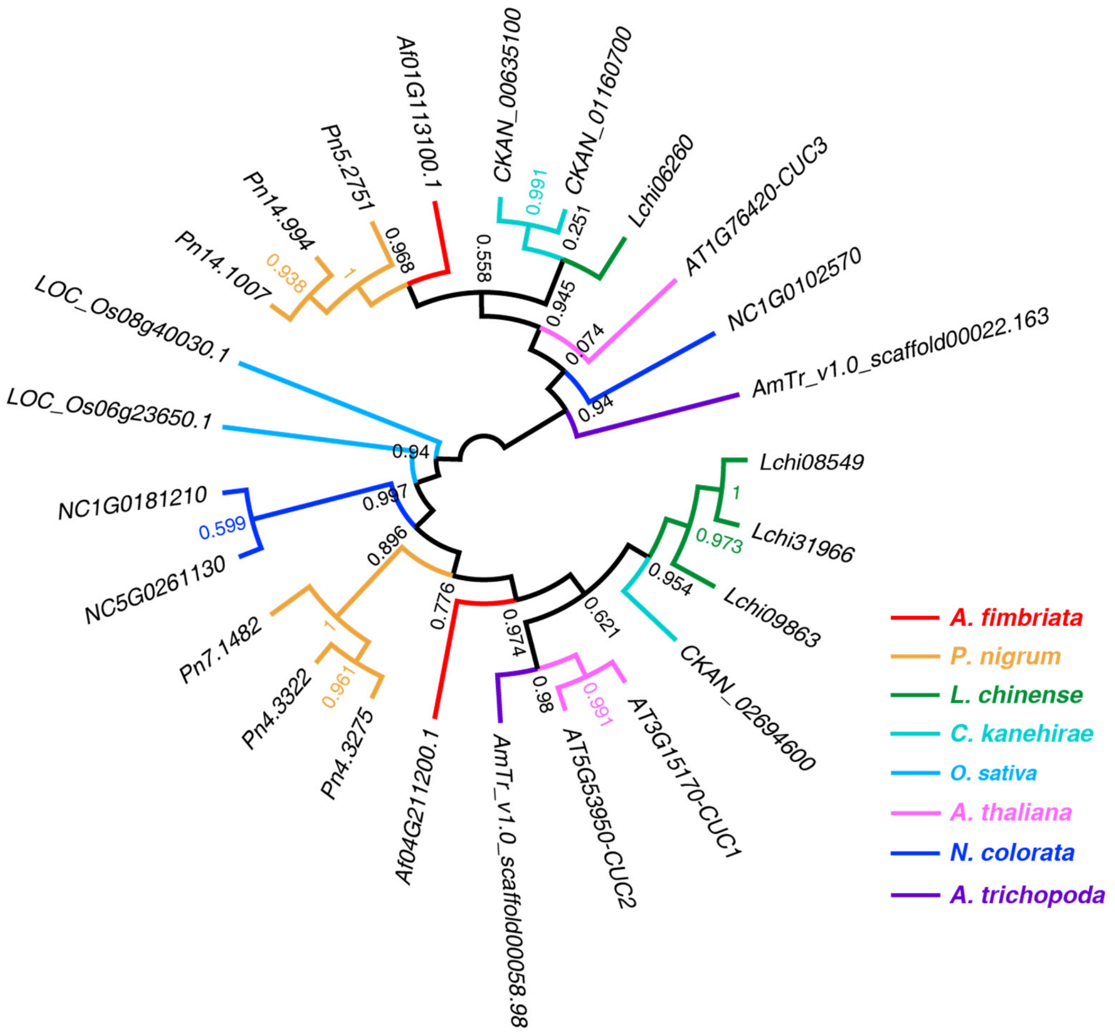


**Supplementary Fig. 5.5 | Phylogenetic tree of *CUC* genes from eight species.**
Branches were colored based on the species color scheme on the right of the tree.

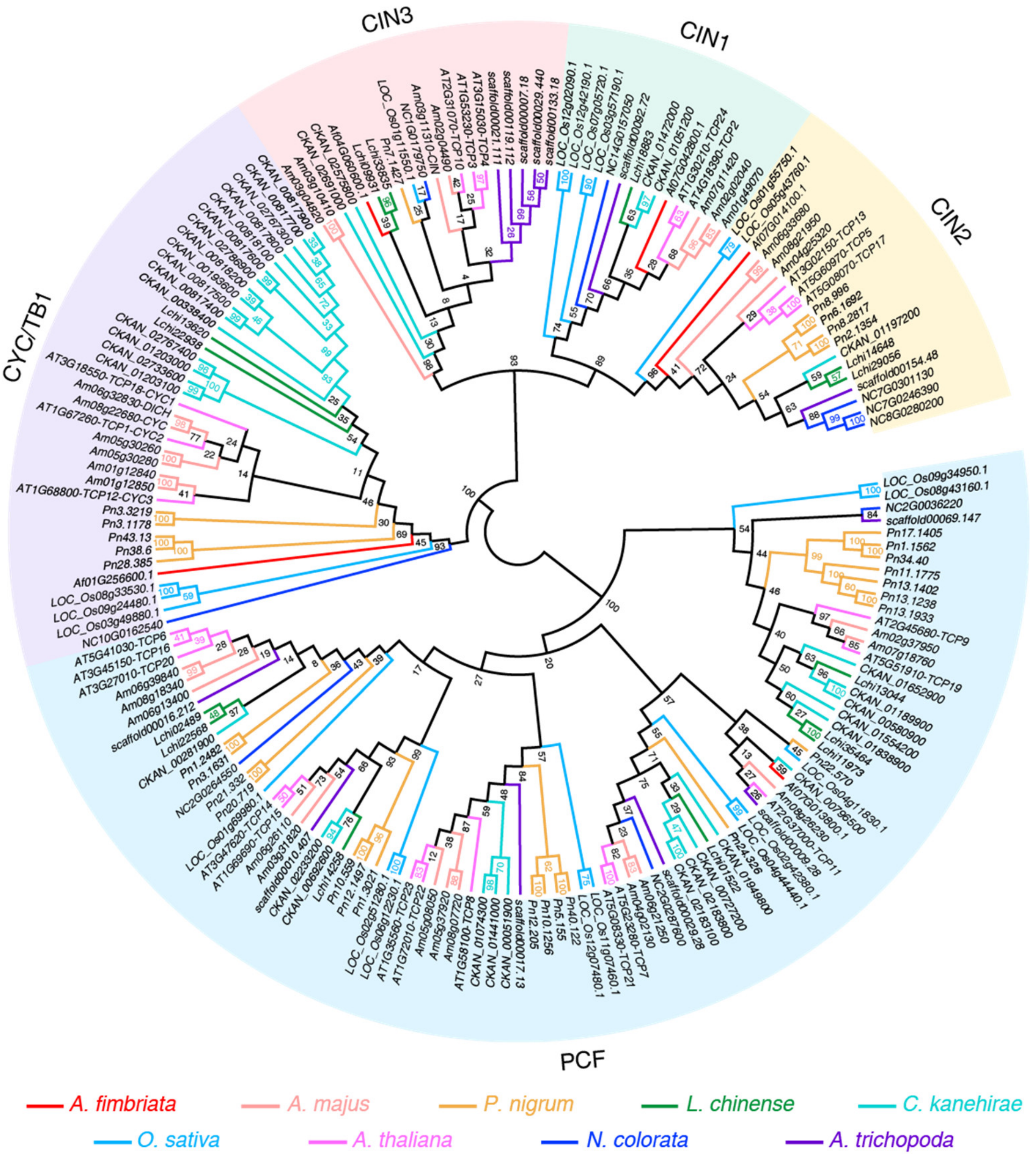


**Supplementary Fig. 5.6 | Maximum likelihood tree of TCP genes from nine species.** The phylogenetic tree was constructed using RAxML. Previously recognized clades were shaded by different colors. Branches were colored based on the species color scheme below the tree.

### 5.5 Floral color and trichome formation genes in *A. fimbriata*

*Aristolochia* flowers often exhibit dull purple-brown color and elaborated inner epidermal modification[3,6,8]. To explore the underlying genetic control of dull purple-brown perianth and a plethora of trichomes in inner perianth epidermis, we explored the putative homologs of the previously acknowledged anthocyanin biosynthetic

genes and trichome developmental genes, and investigated their expression patterns and co-expression networks.

**Materials and Methods**

To identify candidate genes responsible for the development of trichomes in the *A. fimbriata* flower, we first cataloged homologs of the genes involved in trichome formation that were previously identified in other species, such as *A. thaliana*, *S. lycopersicum* and *Cucumis sativus*[140-143]. The related protein sequences of the genes in these species were used as queries to search against the annotated *A. fimbriata* proteins using BLASTP with *E*-value cut-off of $10^{-3}$. The best BLASTP hit in *A. fimbriata* was designated as a homologous candidate gene. Then, phylogenetic analysis was performed based on orthogroup classification (Supplementary Note 2.4) to identify the closely related *A. fimbriata* gene with known *A. thaliana* genes. Finally, the putative genes associated with trichome development in *A. fimbriata* were inferred based on the integrated evidence from the sequence similarity and phylogenetic classification.

Anthocyanins are the main floral pigments that give rise to colors ranging from red to blue[144]. To understand the molecular basis of the unique flower color of *A. fimbriata*, the well-studied anthocyanin biosynthetic genes in *A. thaliana*, *S. lycopersicum* and *Petunia hybrida*[144-148] were retrived. Then, candidate genes involved in anthocyanin biosynthesis in *A. fimbriata* were also identified using the same processes as above (processes used for the identification of trichome development associated genes in *A. fimbriata*).

For the construction of co-expression networks, we used all RNA-Seq data from 14 samples described above (tissues of flowers at anthesis and pre-anthesis, leaves, and seedlings with different treatment) and required genes with TPM$\geqslant$1 in at least one of the samples to be included in the analysis. Pearson correlation coefficients (PCCs) for each bidirectional gene pair were calculated to quantify the correlations. Then, we ranked the PCC values by mutual rank (MR) algorithm to identify the highly correlated gene pairs. Finally, gene pairs with MR$\leqslant$300 were referred to as co-expressed genes[149].

**Results and Discussion**

In *A. fimbriata,* we identified 12 putative homologs of the previously acknowledged trichome developmental genes[140,141], including the trichome initiation and developmental genes, such as *GLABRA3* (*GL3*), *TRANSPARENT TESTA GLABRA1* (*TTG1*), *TRANSPARENT TESTA GLABRA2* (*TTG2*), *TRANSPARENT TESTA8* (*TT8*) and *CAPRICE* (*CPC*), and multicellular trichome formation genes, such as *WIN1*,

*WOOLLY*, and *TRICHOME-LESS* (*TRIL*) (Supplementary Fig. 5.7). Nearly all of these genes are single-copy in *A. fimbriata* (Supplementary Table 5.3), suggesting gene duplication and sub-functionalization are not responsible for the trichome development in the *A. fimbriata* flowers. The transcriptome data show that most of these genes show higher expression at pre-anthesis compared to anthesis (Supplementary Fig. 5.7).

Similarly, fourteen putative anthocyanin biosynthetic genes were identified in *A. fimbriata*, including several critical regulators such as *CHALCONE SYNTHASE* (*CHS*), *FLAVANONE 3-HYDROXYLASE* (*F3H*), *CHALCONE ISOMERASE* (*CHI*), *DIHYDROFLAVONOL 4-REDUCTASE* (*DFR*), *ANTHOCYANIDIN SYNTHASE* (*ANS*), *PRODUCTION OF ANTHOCYANIN PIGMENT 1* (*PAP1*), *TT8*, and *GL3*. We found that most of these genes are also single-copy in *A. fimbriata* (Supplementary Table 5.4). However, no homolog was identified to encode the flavonoid 3’5’-hydroxylase (F3’5’H), a key enzyme for the synthesis of delphinidin-based lilac to blue anthocyanins, indicating the absence of delphinidin-based anthocyanins in *A. fimbriata* flowers. The genes encoding key enzymes (e.g., *CHS*, *F3H*, *DFR*, and *ANS*) and several positive regulators show higher expression in perianth (limb, tube, and utricle) of the flower at the pre-anthesis (developmental stage 8) compared to anthesis (Fig. 5f). The stage with relatively higher gene expression is same as the previously known pigmentation stage (flower developmental stage 8) in *A. fimbriata*[122]. These results suggest these candidate genes are likely involved in the biosynthesis of cyanidin and/or pelargonidin-based anthocyanins, and therefore may be responsible for the dull purple-brown color in the perianth of *A. fimbriata* flowers.

By investigating co-expression networks, we found that the MADS-box B-function genes in *A. fimbriata*, *AP3* and *PI*, are positively co-expressed with three key anthocyanin biosynthesis candidate genes (*F3H*, *DFR*, *ANS*)[146] and a bHLH transcription factor (*TT8*) that has been reported to function in both anthocyanin biosynthesis and trichome formation (Fig. 5g and Supplementary Fig. 5.8)[150]. We also identified CArG-box motifs (MADS-box binding sites) in the promoter regions of *F3H*, *DFR*, *ANS*, and *TT8* genes (Supplementary Table 5.5). In addition, it also has been proposed that the expression of *PI* correlates with the occurrence of conical cellular differentiation and pigment accumulation in *Aristolochia* and other species[151,152]. Thus, the results suggest that novel regulatory relationships between the MADS-box B-function genes and the anthocyanin biosynthesis genes (as well as several trichome formation genes) might have contributed to the development of the showy color and elaborated epidermis of the *A. fimbriata* flowers.

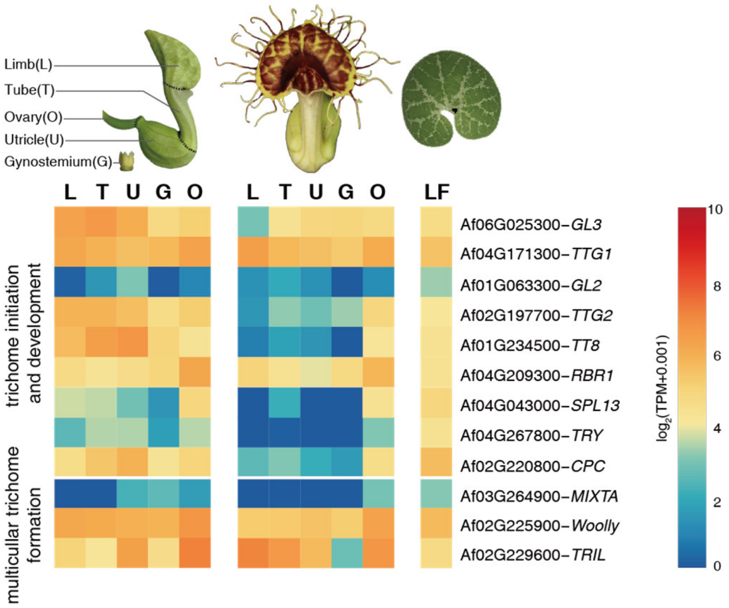


**Supplementary Fig. 5.7 | Expression patterns of the putative candidate genes involved in trichome formation in the late pre-anthetic and anthetic flower and leaf.**

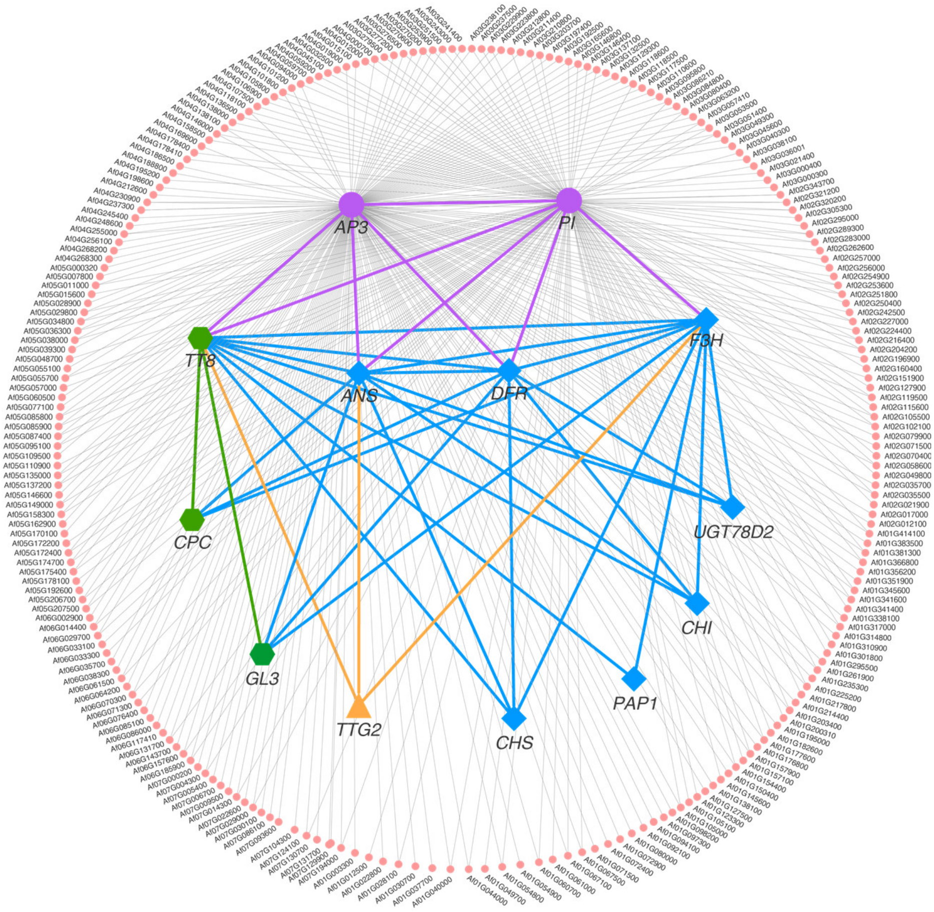


**Supplementary Fig. 5.8 | The details of the co-expression network containing MADS-box B-class genes, genes involved in anthocyanin biosynthesis, and several trichome formation genes.**

## 6 Metabolic gene clusters in *A. fimbriata* and the biosynthesis of terpenoid and aristolochic acids

*Aristolochia* is well-known for its production of a complex mixture of secondary metabolites and has been extensively used for traditional medicines[9]. However, a class of nitrophenanthrene carboxylic acids are naturally synthesized in *Aristolochia* called aristolochic acids (AAs), which have been demonstrated to be highly nephrotoxic and carcinogenic to humans[10-13]. Thusly, many herbal medicines are banned or restricted due to their AAs content. It has been postulated that aporphine alkaloids may be biogenetical precursors to AAs[153]. Here, we explored the utility of our genome assembly as a functional genomics resource to investigate the molecular basis of the secondary metabolites, especially the terpenoids and aristolochic acid.

### 6.1 Identification of metabolic gene clusters in the *A. fimbriata* genome

**Materials and Methods**

First, we annotated genes that were predicted to encode enzymes involving in the biosynthesis of common secondary metabolites using the KEGG automatic annotation server (https://www.genome.jp/kaas-bin/kaas_main) with the assignment method of BBH (bi-directional best hit). In addition, we also employed a metabolic database to annotate putatively related enzyme genes by the E2P2 package v3.1 using default parameter settings (https://gitlab.com/rhee-lab/E2P2/tree/master). Third, we searched for potential biosynthetic gene clusters in the *A. fimbriata* genome that are associated with secondary metabolite biosynthesis using plantiSMASH[154] with default parameters. Lastly, for the functionally important genes, we further identified the other gene family members by investigating the orthogroups we constructed in the above gene family analyses (Supplementary Note 2.4) and requiring these genes with corresponding Pfam models. Phylogenetic analyses were performed using the methods described in Supplementary Note 5.1. Amino acid sequence alignments for the orthologous genes between *A. fimbriata* and the functionally well-studied species were generated, and the conservation level of previously characterized functional active residues were carefully examined to better predict the candidates in *A. fimbriata.*

**Results and Discussion**

We annotated 1,803 genes across the *A. fimbriata* genome into 20 secondary metabolism pathways (Supplementary Table 6.1). Four alkaloid biosynthesis pathways were annotated: ‘Isoquinoline alkaloid biosynthesis’, ‘Indole alkaloid biosynthesis’, ‘Indole diterpene alkaloid biosynthesis’ and ‘Tropane, piperidine and

pyridine alkaloid biosynthesis'. Two tyrosine anabolism pathways were also predicted: 'Tyrosine metabolism' and 'Phenylalanine, tyrosine and tryptophan biosynthesis'. It has been proposed that the potential precursor compounds and reaction processes related to AAs were in the isoquinoline alkaloid biosynthesis (ko00950) and tyrosine metabolism (ko00350) pathways[155]. Here, we annotated 164 genes in the *A. fimbriata* genome belonging to these two pathways (Supplementary Table 6.1).

It also has been shown that genes involved in secondary metabolic pathways sometimes locate together as biosynthetic gene clusters (BGCs) in plant genomes[156,157]. By using the computational toolkit plantiSMASH[154], we detected 33 BGCs related to various plant secondary metabolic pathways (Supplementary Fig. 6.1 and Supplementary Table 6.2), which included five alkaloid-, two polyketide-, three saccharide-, two saccharide-alkaloid-, four saccharide-terpene-, seven terpene-, two terpene-alkaloid-related, and eight putative gene clusters. The genomic regions of these BGCs spanned from 19.47 to 358.74 Kb and contained multiple core protein domains related to various secondary metabolism. The large proportion of the annotated alkaloid (9/33) and terpene-related (14/33) BGCs might be associated with the enriched production of alkaloid and terpenoid compounds in *A. fimbriata*.

Polyketides are compounds that possess various critical biological activities, including antibiotic, anticancer, antifungal, immunosuppressive, and neurotoxic[158-160] properties, and have been considered as a useful source of pharmaceutical agents[159,161]. The polyketides could be one of the key compounds for its pharmaceutical usage. Polyketide synthases (PKSs) are the key enzymes for the biosynthesis of a large class of pharmacologically valuable compounds, which have been classified into three types[160]. Based on the annotated Pfam model and conserved domains, we found four genes belonging to type III PKS in *A. fimbriata*, and the Af04G164800 gene was annotated in the polyketide gene cluster (BGC #6) which also showed the highest sequence similarity to the chalcone synthase (CHS) gene AT5G13930 in *Arabidopsis* (Supplementary Fig. 6.3). Phylogenetic analysis further confirmed the close relationship of *Af04G164800* and the known *CHS* gene (*AT5G13930*) in *Arabidopsis* in Clade P1 (Supplementary Fig. 6.2). The sequence alignment showed that the previously characterized active sites of the Cys-His-Asn (CHN) catalytic triad and Phe in *Medicago sativa* are conserved in *A. fimbriata* and *A. thaliana*[162] (Supplementary Fig. 6.3). Based on these results, we propose that the *Af04G164800* gene is mostly likely the candidate gene encoding chalcone synthase in *A. fimbriata*, which could be functionally tested further if interested.

1. chr02 - Gene Cluster 1. Type = alkaloid. Location: 4804428 - 4831122 nt.

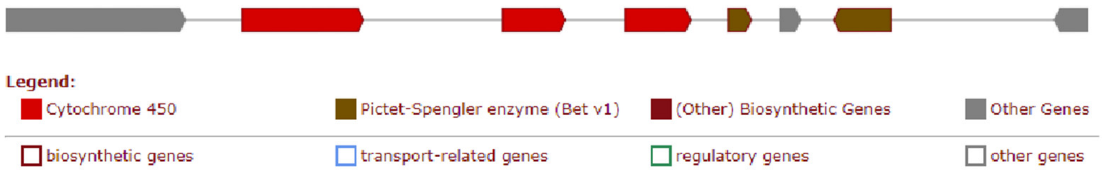


2. chr02 - Gene Cluster 2. Type = alkaloid. Location: 40032211 - 40062418 nt.

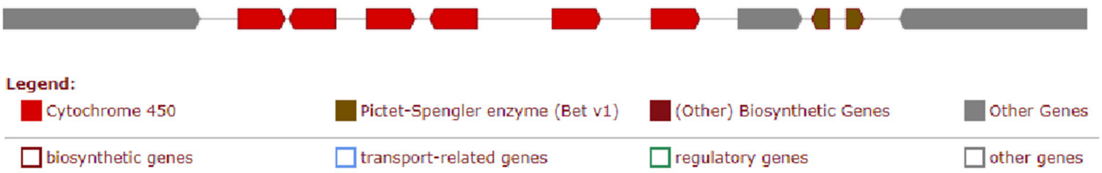


3. chr03 - Gene Cluster 3. Type = alkaloid. Location: 31357932 - 31632713 nt.

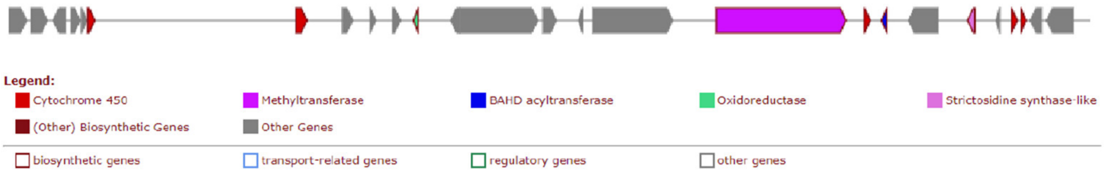


4. chr03 - Gene Cluster 4. Type = alkaloid. Location: 31671702 - 31730008 nt.

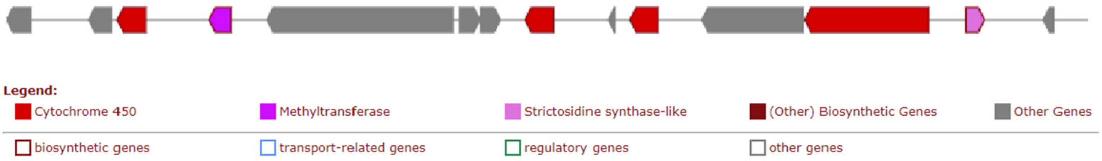


5. chr05 - Gene Cluster 5. Type = alkaloid. Location: 2443496 - 2539325 nt.

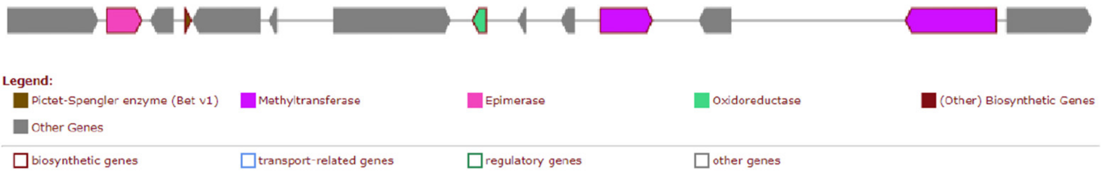


6. chr04 - Gene Cluster 6. Type = polyketide. Location: 24900798 - 24945969 nt.

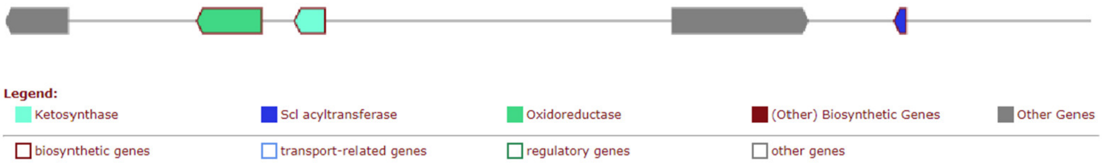


7. chr07 - Gene Cluster 7. Type = polyketide. Location: 21455618 - 21533274 nt.

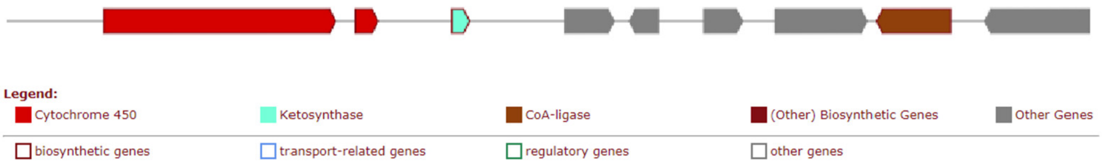


8. chr02 - Gene Cluster 8. Type = saccharide. Location: 1397150 - 1485140 nt.

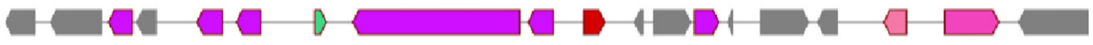

Legend:
Cytochrome 450 | Glycosyltransferase | Methyltransferase | Epimerase | Oxidoreductase
(Other) Biosynthetic Genes | Other Genes
biosynthetic genes | transport-related genes | regulatory genes | other genes

**9. chr07 - Gene Cluster 9. Type = saccharide. Location: 18562708 - 18582182 nt.**

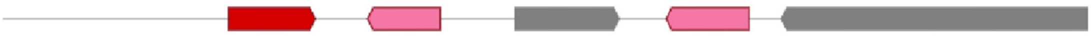

Legend:
Cytochrome 450 | Glycosyltransferase | (Other) Biosynthetic Genes | Other Genes
biosynthetic genes | transport-related genes | regulatory genes | other genes

**10. chr02 - Gene Cluster 10. Type = saccharide-alkaloid. Location: 35476718 - 35678337 nt.**

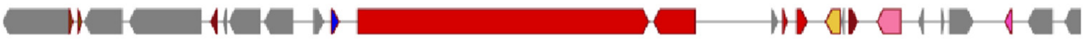

Legend:
Cytochrome 450 | Copper amine oxidase | Pictet-Spengler enzyme (Bet v1) | Glycosyltransferase | BAHD acyltransferase
Epimerase | (Other) Biosynthetic Genes | Other Genes
biosynthetic genes | transport-related genes | regulatory genes | other genes

**11. chr06 - Gene Cluster 11. Type = saccharide-alkaloid. Location: 4570830 - 4644656 nt.**

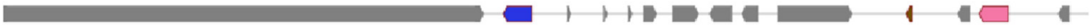

Legend:
Pictet-Spengler enzyme (Bet v1) | Glycosyltransferase | Scl acyltransferase | (Other) Biosynthetic Genes | Other Genes
biosynthetic genes | transport-related genes | regulatory genes | other genes

**12. chr03 - Gene Cluster 12. Type = saccharide-terpene. Location: 1454222 - 1490131 nt.**

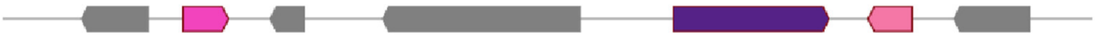

Legend:
Glycosyltransferase | Epimerase | Prenyltransferase | (Other) Biosynthetic Genes | Other Genes
biosynthetic genes | transport-related genes | regulatory genes | other genes

**13. chr06 - Gene Cluster 13. Type = saccharide-terpene. Location: 3350754 - 3416888 nt.**

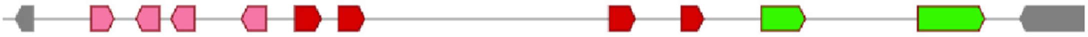

Legend:
Cytochrome 450 | Terpene synthase | Glycosyltransferase | (Other) Biosynthetic Genes | Other Genes
biosynthetic genes | transport-related genes | regulatory genes | other genes

**14. chr06 - Gene Cluster 14. Type = saccharide-terpene. Location: 19861542 - 19948891 nt.**

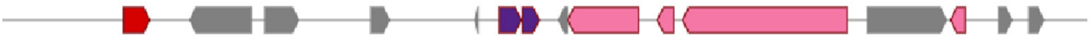

Legend:
Cytochrome 450 | Glycosyltransferase | Prenyltransferase | (Other) Biosynthetic Genes | Other Genes
1738 biosynthetic genes | transport-related genes | regulatory genes | other genes

15. chr07 - Gene Cluster 15. Type = saccharide-terpene. Location: 19755066 - 19856792 nt.

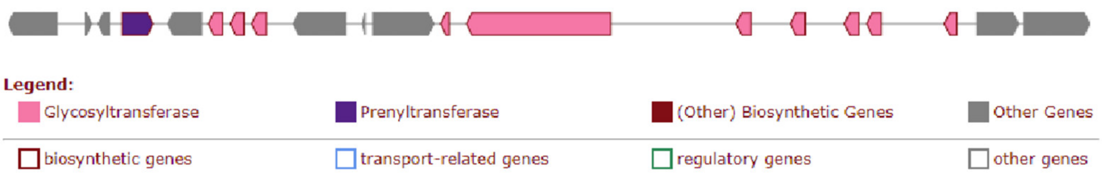


16. chr01 - Gene Cluster 16. Type = terpene. Location: 5547305 - 5586781 nt.

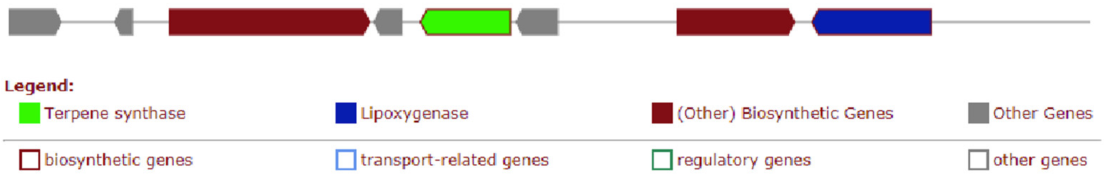


17. chr02 - Gene Cluster 17. Type = terpene. Location: 29635093 - 29814343 nt.

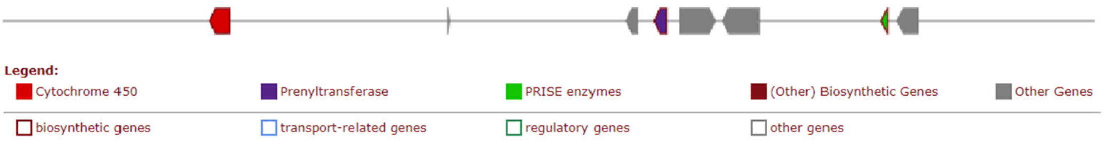


18. chr02 - Gene Cluster 18. Type = terpene. Location: 31658540 - 31727527 nt.

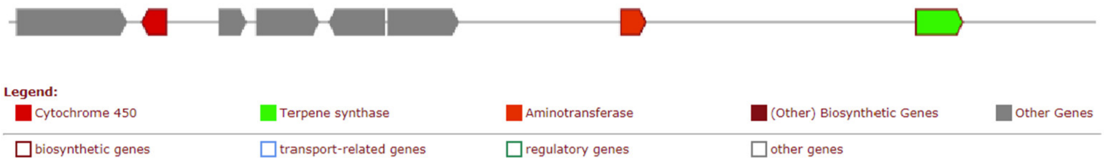


19. chr02 - Gene Cluster 19. Type = terpene. Location: 32427377 - 32579422 nt.

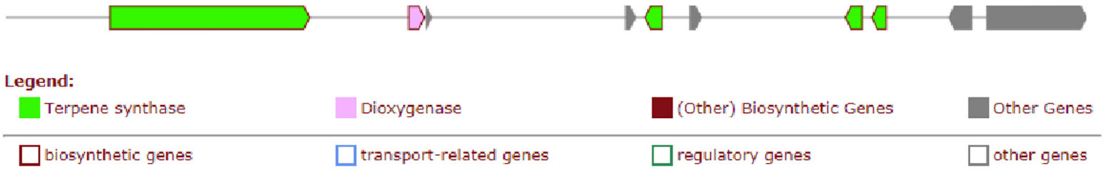


20. chr02 - Gene Cluster 20. Type = terpene. Location: 32662073 - 32803745 nt.

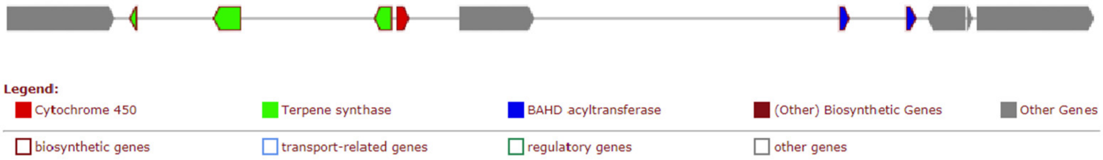


21. chr05 - Gene Cluster 21. Type = terpene. Location: 2122765 - 2187644 nt.

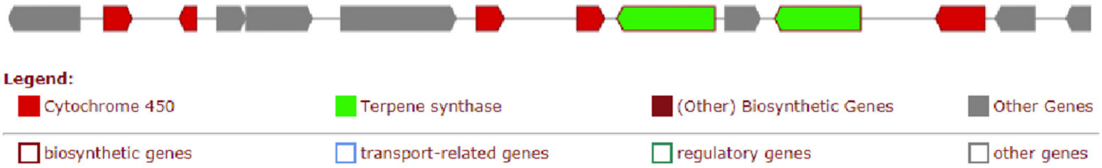


22. chr06 - Gene Cluster 22. Type = terpene. Location: 23934688 - 23994410 nt.

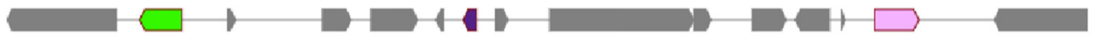

**Legend:**
Terpene synthase | Dioxygenase | Prenyltransferase | (Other) Biosynthetic Genes | Other Genes
biosynthetic genes | transport-related genes | regulatory genes | other genes

## 23. chr07 - Gene Cluster 23. Type = terpene. Location: 585971 - 638309 nt.

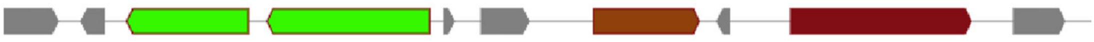

**Legend:**
Terpene synthase | CoA-ligase | (Other) Biosynthetic Genes | Other Genes
biosynthetic genes | transport-related genes | regulatory genes | other genes

## 24. chr01 - Gene Cluster 24. Type = terpene-alkaloid. Location: 9719385 - 9803606 nt.

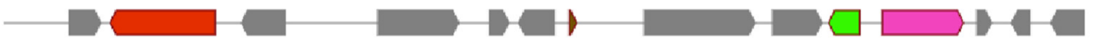

**Legend:**
Terpene synthase | Pictet-Spengler enzyme (Bet v1) | Epimerase | Aminotransferase | (Other) Biosynthetic Genes
Other Genes
biosynthetic genes | transport-related genes | regulatory genes | other genes

## 25. chr05 - Gene Cluster 25. Type = terpene-alkaloid. Location: 1938278 - 2004826 nt.

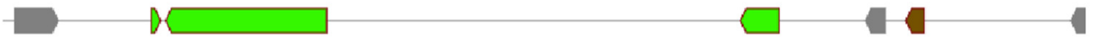

**Legend:**
Terpene synthase | Pictet-Spengler enzyme (Bet v1) | (Other) Biosynthetic Genes | Other Genes
biosynthetic genes | transport-related genes | regulatory genes | other genes

## 26. chr02 - Gene Cluster 26. Type = putative. Location: 126101 - 277600 nt.

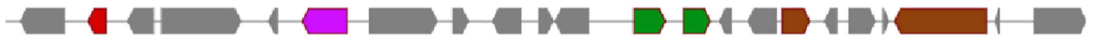

**Legend:**
Cytochrome 450 | Methyltransferase | CoA-ligase | Cellulose synthase-like | (Other) Biosynthetic Genes
Other Genes
biosynthetic genes | transport-related genes | regulatory genes | other genes

## 27. chr02 - Gene Cluster 27. Type = putative. Location: 982170 - 1088396 nt.

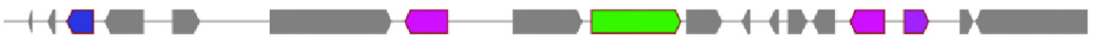

**Legend:**
Terpene synthase | COesterase | Methyltransferase | Scl acyltransferase | (Other) Biosynthetic Genes
Other Genes
biosynthetic genes | transport-related genes | regulatory genes | other genes

## 28. chr03 - Gene Cluster 28. Type = putative. Location: 3558163 - 3658935 nt.

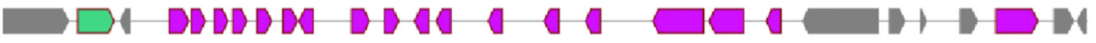

**Legend:**
Methyltransferase | Oxidoreductase | (Other) Biosynthetic Genes | Other Genes
biosynthetic genes | transport-related genes | regulatory genes | other genes

## 29. chr03 - Gene Cluster 29. Type = putative. Location: 23666975 - 24006353 nt.

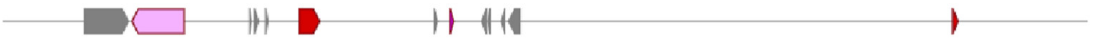

**Legend:**
Cytochrome 450 | Methyltransferase | Dioxygenase | (Other) Biosynthetic Genes | Other Genes
biosynthetic genes | transport-related genes | regulatory genes | other genes

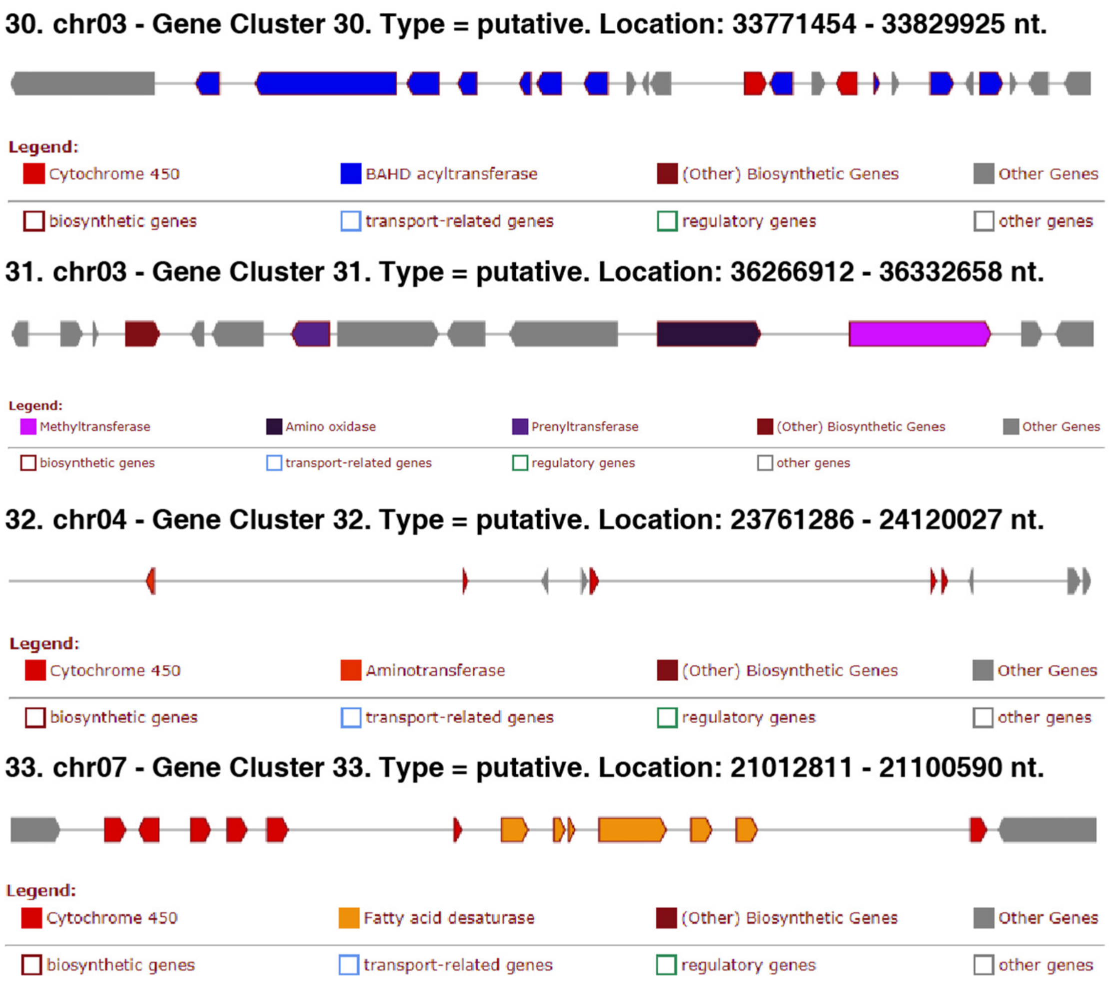


**Supplementary Fig. 6.1 | Visualization of 33 BGCs identified by plantiSMASH.** Various core enzymes were annotated, and the related genes were colored according to the enzymatic classes.

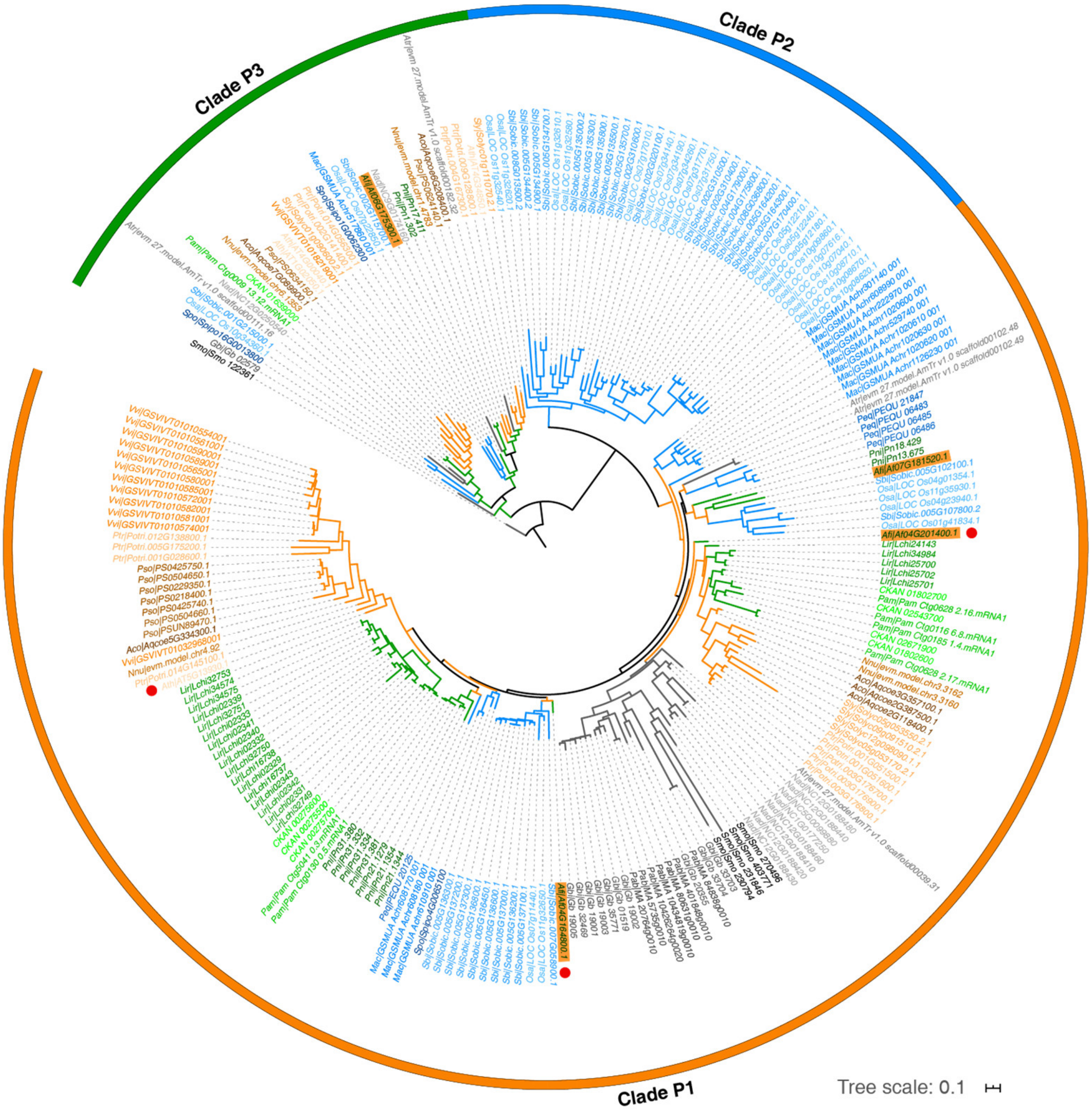


**Supplementary Fig. 6.2 | Phylogeny of the type III PKS gene family.** Branches of the phylogeny were colored: orange-eudicots; blue-monocots; green-magnoliids; gray-ANA grade, gymnosperms and lycophyte. The previously identified functional gene in *A. thaliana* and the two *A. fimbriata* candidate genes are marked with red dots. The *A. fimbriata* genes are highlighted with an orange background.

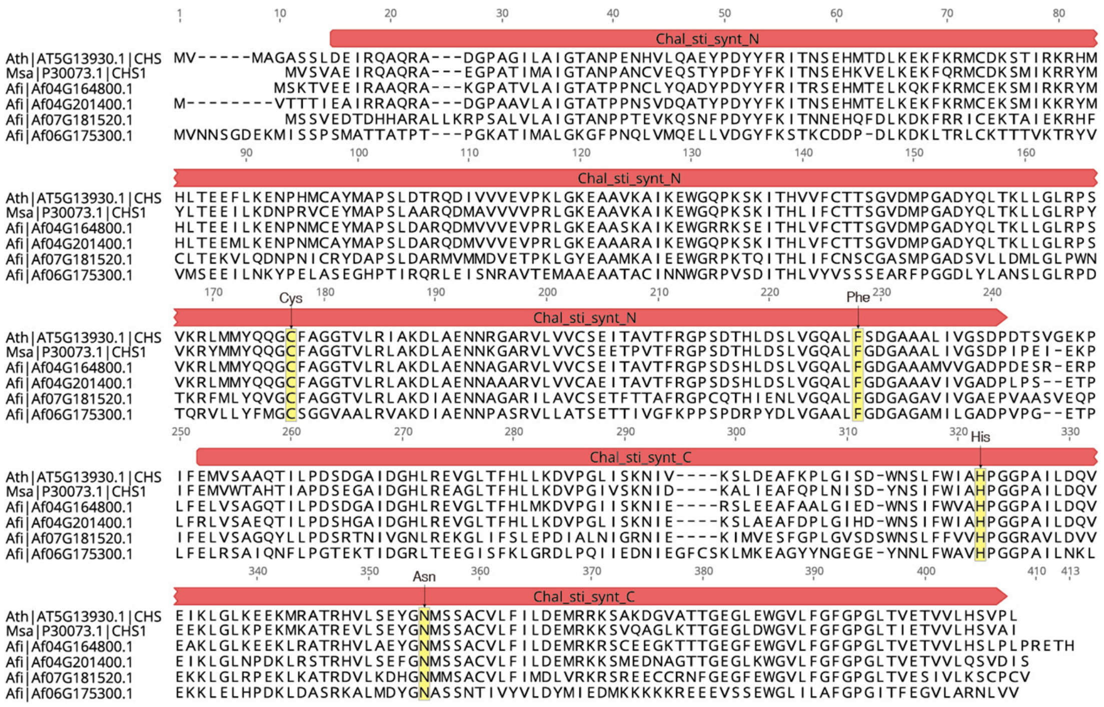


**Supplementary Fig. 6.3 | Multiple sequence alignment of CHS proteins in *M. sativa*, *A. thaliana,* and *A. fimbriata*.** The previously characterized active catalytic residues (Cys-His-Asn triad and Phe) are highlighted in yellow. The identified four *Aristolochia* protein sequences seem to be well-conserved compared to their orthologs in *M. sativa* and *A. thaliana*.

## 6.2 Floral scent and terpenes

### Materials and Methods

The complex and diverse floral scent composition is crucial for the deceptive pollination systems in *Aristolochia*[3,7,163]. To investigate the floral volatile production of *A. fimbriata*, we collected the newly opened flowers for gas chromatography-mass spectrometry (GC-MS) analysis, with the added 0.0825 µg of 3-Octanol as an internal standard. Then, the samples were incubated at 40 °C for 30 min. The volatiles were further extracted using SPME fiber with 50/30 µm divinylbenzene/carboxen/polydimethylsiloxane (DVB/CAR/PDMS) (Supelco Co., Bellefonte, PA, USA). Finally, GC-MS analysis was conducted on an Agilent 7890B gas chromatograph coupled to a mass spectrometer (Agilent 7000D, Santa Clara, CA, USA) with a fused silica capillary column (HP-5MS) coated with polydimethylsiloxane (19091S-433UI) (30m × 0.25 mm internal diameter, 0.25 µm film thickness). The oven temperature was programmed to start at 40 °C for 3 min, and then ramped to 130 °C at a rate of 5 °C $min^{-1}$, followed by a second ramp to

156 °C at a rate of 2 °C $min^{-1}$, the final ramp to 280 °C at a rate of 10 °C $min^{-1}$. Three biological replicates were conducted for the GC-MC analysis.

Terpenes are one of the major groups of flower volatile compounds in numerous plants[164,165], and monoterpenoids and sesquiterpenoids have also been commonly detected in several *Aristolochia* species[7,166,167]. Terpene synthases (TPSs) are pivotal enzymes for the biosynthesis of terpenes[164,165], and here we explored the number and expression of TPS genes to infer their putative roles in *A. fimbriata*. To identify TPS genes in *A. fimbriata* be consistent with the other studies, we performed profile HMM searches against the predicted protein sequences by using hmmsearch in HMMER v3.3[114] (--domE 0.001). To be selected, putative TPS genes were required to match at least one of the Pfam models (PF01397 and PF03936), which correspond to the conserved domains localized at the N and C termini of known terpene synthases (TPSs) respectively[168]. We also identified TPS genes in *A. trichopoda*, *N. colorata*, *O. sativa* and three other magnoliids (*C. kanehirae*, *P. nigrum*, and *L. chinense*) and compared them with the genes in previously published reports[55,59,70,168]. The well-studied and annotated subfamilies of TPS genes in *A. thaliana*[168] were used as reference for phylogenetically classifying these TPS genes into corresponding subfamilies. In addition, the TPS genes located in the annotated biosynthetic gene clusters were further identified, and their expression patterns were investigated as in Supplementary Note 5.3.

**Results and Discussion**

GC-MS analyses revealed a complex of *A. fimbriata* flower scent, including terpenoids (sesquiterpenes and monoterpenoids), fatty acid derivatives and benzenoids. Among the terpenoids, the sesquiterpenes are the most abundant volatile compounds (Fig. 6a), while the diterpenoids were not detected in the *A. fimbriata* flower volatiles. Then, the candidate TPS genes responsible for the biosynthesis of sesquiterpenes and monoterpenoids in *A. fimbriata* were further identified based on comprehensive analyses of phylogeny, associated biosynthetic gene clusters, and spatio-temporal expression patterns.

After comprehensive TPS family annotation, 41 genes were identified in the *A. fimbriata* genome, and 16 of them were located in the 13 terpene-related biosynthetic gene clusters noted above (Supplementary Fig. 6.1 and Supplementary Table 6.2). The TPS genes identified in four of six other species (*A. trichopoda*, *N. colorata*, *O. sativa* and *C. kanehirae*) were largely consistent with previous report[55,59,70,168]. The TPS genes in the other two species (*L. chinense* and *P. nigrum*) were newly annotated. Phylogenetic analysis classified the TPS genes into five previously recognized subfamilies, representing TPS-a, TPS-b, TPS-c, TPS-e/f, and TPS-g based on the well-studied *A. thaliana* TPS genes (Fig. 6b and Supplementary Table 6.3).

There are 21 genes in *A. fimbriata* clustered in subfamily of TPS-a, which are usually encoding sesquiterpene synthases[164]. Notably, the *Af06G158900* gene in the TPS-a subfamily showed extremely high and increased expression in the utricle of anthetic flowers compared to pre-anthetic flowers and leaves, which is consistent with the abundant component of sesquiterpenes in the newly opened flower volatiles (Fig. 6c). Moreover, the *Af06G158900* gene was also identified in the terpene-related gene cluster (BGC #22), consisting of genes encoding terpene synthase, dioxygenase, and prenyltransferase (Fig. 6d). Another gene is *Af06G160300* which also showed high expression in the limbs of flowers at anthesis, while almost no expression in the pre-anthetic floral organs and leaves. Together with the previous knowledge that the osmophores in the limb and utricle may be the major floral fragrance glands emitting floral scents[3,122,169], the *Af06G158900* and *Af06G160300* genes, especially the former, are likely the main sesquiterpene synthase genes in *A. fimbriata*.

We also examined the monoterpene synthase genes for the other main flower volatile compounds in *A. fimbriata*, and the subfamilies of TPS-g and TPS-b were proposed encoding monoterpene synthases[164,168,170]. We found the subfamily TPS-g has recently expanded in *A. fimbriata*, resulting in 9 TPS-g genes (Fig. 6b). The *Af01G154900* and *Af06G245300* among these nine TPS-g genes showed increased expression in the utricle and limb of anthetic flowers, respectively, compared to the counterparts of pre-anthetic flowers and the leaves (Fig. 6c). Notably, the *Af01G154900* gene in the TPS-g clade was also annotated in BGC #24 (Fig. 6d). These results indicated that the *Af01G154900* gene might be one of the key candidate genes encoding monoterpene synthases in *A. fimbriata*. Another subfamily encoding monoterpene synthases is TPS-b, and 6 genes in *A. fimbriata* were identified. A pair of tandem duplicates (*Af05G030400* and *Af05G030410*) shows relatively stable expression in leaf tissue and pre-anthetic and anthetic flowers (Fig. 6c). Functional studies could be employed in the future to validate the function of these putative candidate genes, and ultimately help to better understand the deceptive pollination system in *Aristolochia*.

It has been suggested that the genes in TPS-c and TPS-e/f clades are responsible for the biosynthesis of diterpenoids[164,168]. We found that the *A. fimbriate* genes in these clades showed very weak expression at both pre-anthetic and anthetic flowers (Fig. 6b,c). This finding suggests a lack of diterpenoids in *A. fimbriata* flower volatile compounds, which is consistent with the result of GC-MS analysis.

### 6.3 Liquid Chromatograph Mass Spectrometer (LC-MS)-based metabolomic analysis and reconstruction of aristolochic acid I biosynthesis pathway

**Materials and Methods**

AA I and II were found as the most common AA analogues, and these are also responsible for the nephrotoxic and carcinogenic effects[4]. We performed an LC-MS-based metabolomic analysis for the root, stem, leaf, and fruit from one-year-old *A. fimbriata* plants. 50 mg of each dried tissue were processed for the HPLC-DAD-ESIMS/MS measurements. AAs were separated by UPLC (Waters, ACQUITY) equipped with an ACQUITY UPLC HSS T3 column (Waters) and detected by MS/MS using a Triple Quad Xevo TQ-S (Waters) mass spectrometer. The mobile phase consists of buffer A (5 mM ammonium acetate and 0.1% formic acid) and buffer B (100% acetonitrile). AAs were qualified using the ion mass transitions of m/z 324.1/237 and 324.1/280 for AA I and m/z 329/238 and 329/268 for AA II, respectively, and the base ions were ammonium adduct ions $[M + NH_4]^+$. For quantitative analysis, we used a higher abundance of the adduct ion mode. Standard curves were generated by running a concentration series of pure commercial aristolochic acids. The content of aristolochic acids in each sample was then calculated by fitting the peak areas to the standard curves.

**Results and Discussion**

From the LC-MS investigation, we found an AA I peak at a retention time of 3.56 minute and the AA II peak at retention time of 2.99 minute in the standard reference curves. For our experimental samples, we observed the AA I peak at retention time of 3.56 minute in the tissues of root, stem, leaf, and fruit of *A. fimbriata*, but no AA II peak was observed (Extended Data Fig. 9). The results indicated that *A. fimbriata* contains AA I, but likely no AA II, which is consistent with a previous report[4]. Therefore, we mainly focused on the reconstruction of the biosynthetic pathway of AA I.

It has been indicated that several key intermediate such as benzylisoquinoline from norlaudanosoline to orientaline, orientalinone, orientalinol, prestephanine, stephanine may be involved in the biosynthesis of AA I[153,171,172]. Norlaudanosoline could be formed via the condensation of dopamine and 3,4-dihydroxyphenylacetaldehyde by norcoclaurine synthase (NCS), while the incorporation of tyrosine, dopa, dopamine, and 3,4-dihydroxyphenylacetaldehyde to norlaudanosoline could be similar to the first part of the benzylisoquinoline alkaloids (BIAs) biosynthetic pathway (tyrosine metabolism pathway: ko00350 and isoquinoline alkaloid biosynthesis pathway: ko00950)[173]. Through integrating the previous hypothesis[153,171,172] and the KEGG annotations, we reconstructed the AA I biosynthesis pathway with the potential biogenetic route of L-tyrosine→L-dopa→dopamine→3,4-dihydroxyphenylacetaldehyde→(S)-norlaudanosoline→(S)-6-O-methylnorlaudanosoline→(S)-nororientaline→orientaline→orientalinone→ orientalinol→prestephanine→stephanine→aristolochic acid I (Fig. 6e).

Furthermore, we identified the putative genes encoding key enzymes involved in the AA I biosynthesis pathway based on previous studies[173,174], including tyrosinases (TYR), tyrosine/dopa decarboxylases (TYDC), monoamine oxidases (MAO), norcoclaurine synthase (NCS), norcoclaurine 6-O-methyltransferase (6OMT), 6-O-methylnorlaudanosoline 5'-O-methyltransferase (5'OMT), coclaurine N-methyltransferase (CNMT), and possibly cytochrome P450s (CYPs). Those enzymes in other species mostly were verified to assign their functions using heterologous expression of microbial systems (Supplementary Table 6.4). Based on seed motif from the Pfam database[113], we identified the candidate genes encoding key enzymes involved in the AA I biosynthesis pathway, including 7 TYRs, 2 TYDCs, 6 MAOs, 26 NCSs, 29 OMTs and 8 CNMT (Supplementary Table 6.5). In addition, three cytochrome P450 (CYPs) subfamilies including CYP80, CYP719, and CYP82 might play key oxidative roles in alkaloid metabolism[174-178], which could catalyze the formation of C–C or C–O bonds in the BIA pathway[173,179]. We speculate that they could be involved in the reactions of orientaline→orientalinone and prestephanine→ stephanine in the aristolochic acid I pathway. Here, we identified 7 CYP80 genes, 11 CYP719 genes, and 23 CYP82 genes, which are potentially involved in alkaloid biosynthesis in *A. fimbriata* (Supplementary Table 6.6).

## 6.4 Identifying the key candidate genes based on phylogenetic classification and the sequence conservation of key residues

### Materials and Methods

To predict key candidate genes encoding the associated enzymes, we integrated phylogeny, gene expression, conservation of critical enzyme residue analyses. We first performed profile hidden Markov model (HMM) searches based on the seed motif from the Pfam database[113] against the annotated amino acid sequences using hmmsearch in HMMER v3.3[114] (--domE 0.001). Then, sequence alignments and phylogenetic analysis were constructed as described in the above sections (Supplementary Note 5.1). In the following analyses, we specifically compared the candidate genes in *A. fimbriata* to opium poppy (*P. somniferum*), because *P. somniferum* has emerged as a model system to study BIA metabolism. Many functional genes and their corresponding key residues for BIA biosynthesis in *P. somniferum* have been well characterized[180-183], and thus they were used as references to infer the associated candidate genes in *A. fimbriata*.

Expression profiles of key genes were further examined by qPCR. The samples of root, stem, leaf, flower, and fruit collected from 1-year-old *A. fimbriata* plants and one-month-old seedlings were used for gene expression profiling using qRT-PCR. Three independent plants and seedlings were used as biological replicates. Total

RNAs were isolated using an RNA Extraction Kit (Qiagen, Germany), and cDNAs were synthesized using the FastKing RT Kit (TIANGEN BIOTECH, China). Primers were designed and further checked for their specificity (Supplementary Table 6.7). qPCRs were performed on a QTOWER (Jena, Germany) using Real Master Mix SYBR Green II dye (Takara, Japan) according to the manufacturer's instructions. Actin was used as the internal reference gene for normalization. Relative gene expression was performed using the comparative $2^{-\triangle\triangle Ct}$ method[184]. Three technical replicates and three independent biological experiments were performed.

## Results and Discussion

**Coclaurine N-Methyltransferase (CNMT).** The intermediate part of the AA biosynthetic pathway comprises several key steps from norlaudanosoline to orientaline, which seem to involve methylation by an N-methyltransferase (NMT). Coclaurine N-methyltransferases (CNMTs) are a subclass of (S)-adenosyl-L-methionine (SAM)-dependent NMTs [E.C.2.1.1.140], which can catalyze the transfer of a methyl group from SAM to the amino group of coclaurine and several structurally similar alkaloids such as (S)-norcoclaurine, (R, S)-6-O-methylcoclaurine and (R, S)-norlaudanosoline[185-187]. Therefore, we predicted that CNMTs may catalyze the reactions from (S)-nororientaline to orientaline.

Eight putative genes encoding CNMTs were identified in the *A. fimbriata* genome, and a set of six tandem duplicates that phylogenetically clustered with a previously acknowledged functional gene of *P. somniferum* (Supplementary Fig. 6.4). Notably, these six genes were also annotated in a saccharide-type gene cluster (BGC #8). In addition, we further investigated the amino acid sequence alignment of these six *A. fimbriata* genes with the functional *P. somniferum* gene and found they all exhibit high conservation for the functional motif A of plant-SAM and activation sites[188] (Supplementary Fig. 6.5). Our recent study isolated and molecularly characterized a putative CNMT from *A. fimbriata*[188], which is the same gene as *Af02G021500* identified here. In particular, we found that the Af02G022300 gene has the highest sequence conservation for functional sites (Supplementary Fig. 6.5), which should be included for functional validations in the future.

**Norcoclaurine 6-O-methyltransferase (6OMT).** Plant O-methyltransferases (OMTs) constitute a large family of enzymes that can methylate the oxygen atom of a variety of secondary metabolites, including phenylpropanoids, flavonoids, and alkaloids[189]. It has been reported OMTs could be classified into two major groups, including PL-OMT I comprising the caffeoyl CoA OMTs and carboxylic acid OMTs, and PL-OMT II acting on a diverse group of metabolites such as hydroxycinnamic acids, flavonoids, and alkaloids[189]. Here, we mainly focused on PL-OMT II group and the alkaloid-related genes that were previously characterized in BIA pathways in

*Papaver*[180]. Based on the reference genome annotation of *A. fimbriata* and the known KEGG pathways, we predicted that 6OMT [EC 2.1.1.128] might be involved in the biosynthetic pathway of AA I, which could catalyze the reaction of S-Adenosyl-L-methionine + (S)-Norlaudanosoline ⇌ S-Adenosyl-L-homocysteine + (S)-6-O-Methylnorlaudanosoline.

Based on the reconstructed orthogroups and screening for the genes with conserved Pfam model, we identified 478 OMT genes belonging to PL-OMT II in the 22 species, in which the *A. fimbriata* genome contains 29 genes. Phylogenetic analysis classified them into four subclades (II-A, II-B, II-C, II-D) (Supplementary Fig. 6.6), which is consistent with previous reports, and the genes in subclades were involved in different biosynthesis[189-191]. It has been suggested that majority of BIA OMTs fall within subclade II-D (e.g., 6OMT, 4OMT, SOMT)[189-191]. Here, we found that nine genes in *A. fimbriata* and the previously characterized 6OMT gene in *P. somniferum* were classified within the PL-OMT II-D subclade (Supplementary Fig. 6.6). Notably, three of nine genes in *A. fimbriata* were also annotated in three associated BGCs (*Af03G181700* in BGC #29; *Af03G218000* in BGC #3; *Af03G219800* in BGC #4), that were annotated as putative-, alkaloids-, and alkaloids-type clusters, respectively.

In addition, the amino acid sequence alignment revealed that these nine genes all exhibit high sequence similarity to the previously characterized *P. somniferum* gene for the motif regions and the activation sites (Supplementary Fig. 6.7). In particular, the amino acid sequence, the position of motifs A, B, and C, and the activation sites (e.g., $I_{285}$, $K_{288}$, $P_{311}$) in Af04G180700, Af03G181700, Af03G218000 and Af03G219800 genes are highly conserved with that of the *P. somniferum*[181,182]. Together, our results indicate that these four genes (*Af04G180700*, *Af03G181700*, *Af03G218000*, and *Af03G219800*) are likely the functional genes encoding 6OMTs.

**Norcoclaurine synthase (NCS).** NCS catalyzes the first committed step in the biosynthesis of BIAs in plants[192]. NCS genes have been acknowledged to have evolved from the pathogenesis-related 10 (PR10)/Bet v 1 ancestor, and their biochemical activities have been characterized in opium poppy[193,194] and yellow meadow-rue (*Thalictrum flavum*)[195]. Two subfamilies of NCSI and NCSII have been defined[196]. The NCSII genes are universal in plants, while NCSI genes are crucial for BIA biosynthesis in certain families, including Ranunculaceae, Papaveraceae, Berberidaceae, and Nelumbonaceae[192,196].

We found 26 genes belonging to the PR10/Bet v1 family in *A. fimbriata*, and several genes are located closely in the chromosomes and likely resulted from tandem duplications (*Af02G076800*, *Af02G077000*, *Af02G077200*, *Af02G077300*, *Af02G077500*; *Af02G263900*, *Af02G264000*; *Af05G030500*, *Af05G030600*, *Af05G030700*, *Af05G030800*). Based on phylogenetic analysis, we identified 7 NCSI,

17 NCSII, and 2 PR10/Bet v1 genes (Supplementary Fig. 6.8). Consistent with previous reports, a highly conserved P-loop sequence motif could be identified in the NCSI and NCSII genes, but it is absent in PR10/Bet v1 genes[193,196].

NCSI genes are considered to be crucial for BIA biosynthesis. We found that the genes of *Af02G077000* and *Af02G076800* in the NCSI clade were annotated in an alkaloid-associated gene cluster (BGC #1), the *Af02G263900* and *Af02G264000* gene were annotated in a saccharide-alkaloid-associated gene cluster (BGC #10), and the *Af01G154600* and *Af05G030600* genes were located in terpene-alkaloid-associated gene clusters (BGC #24 and #25) (Supplementary Fig. 6.1 and Supplementary Table 6.2). Sequence alignment of the key NCSI amino acid sequences in *A. fimbriata* and *P. somniferum* showed these three genes from *A. fimbriata* (Af01G154600, Af02G077000, and Af02G076800) have conserved catalytic residues with those of *P. somniferum*[183] (Supplementary Fig. 6.9).

In addition, we performed qPCR to quantify the expression levels of the 7 NCSI genes in seedlings and the root, stem, leaf, flower, and fruit of mature plants (Extended Data Fig. 10). In general, most NCS genes have lower expression levels in seedlings than in tissues of mature plants. Two genes (*Af02G077000* and *Af01G154600*) previously identified in BGCs show high expression across root, stem, and fruit tissues in *A. fimbriata,* which seems to be consistent with the amount of their AA I production (Extended Data Fig. 10). Together, these results suggest that *Af02G077000* and *Af01G154600* are likely the main NCS candidate genes in *A. fimbriata*. Further integrated metabolomics, transcriptomics, and functional genetic analyses could be used to validate their roles in the aristolochic acid I biosynthesis.

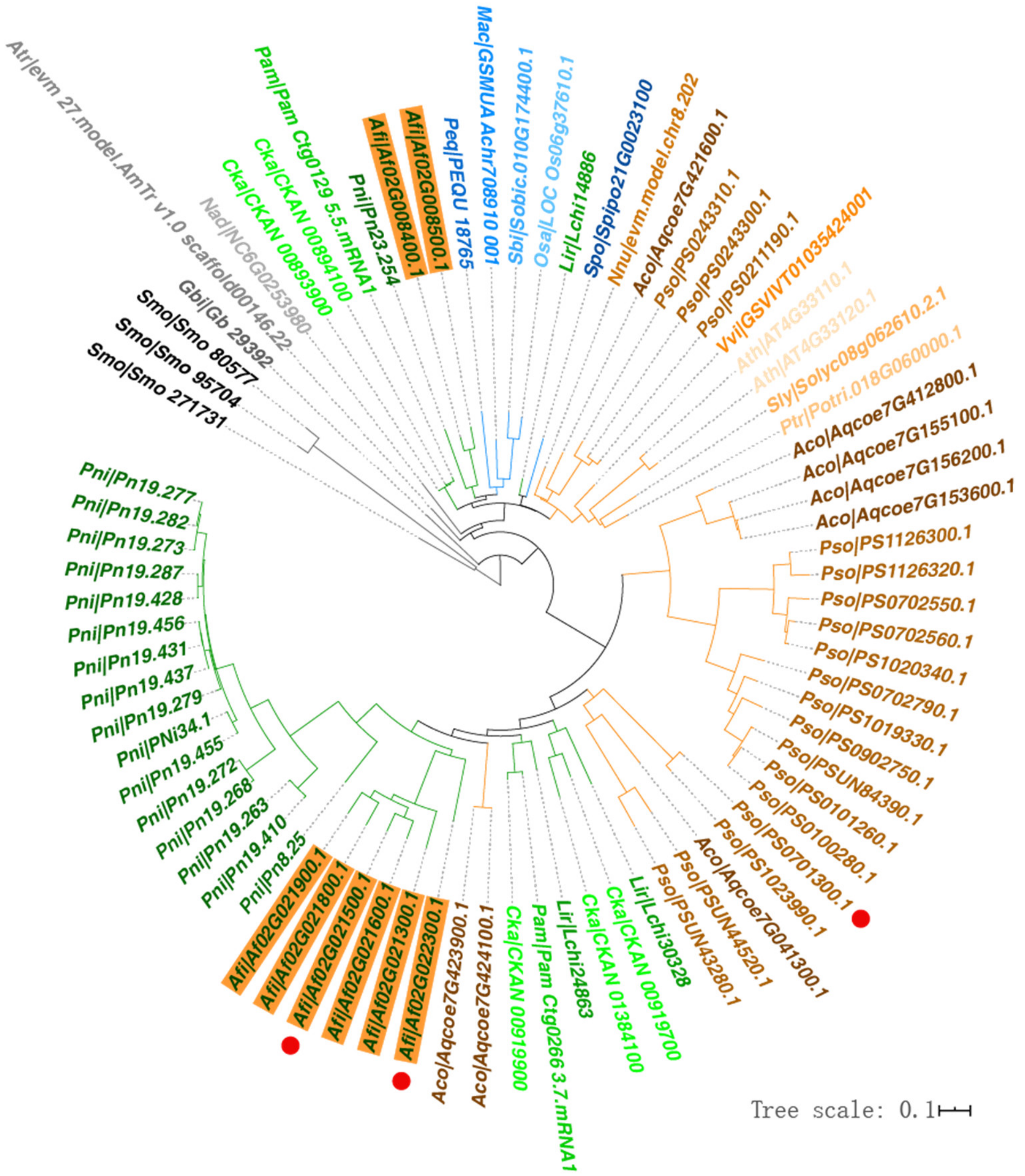


**Supplementary Fig. 6.4 | Phylogeny of the CNMT gene family.** The phylogenetic tree was constructed using RAxML. The *A. fimbriata* genes are highlighted in orange. The previously identified functional gene in *P. somniferum* and the two mentioned *A. fimbriata* genes in text are marked with red dots. Branches of the phylogeny were colored: orange-eudicots; blue-monocots; green-magnoliids; gray-ANA grade, gymnosperms and lycophyte. The *A. fimbriata* genes are highlighted with an orange background.

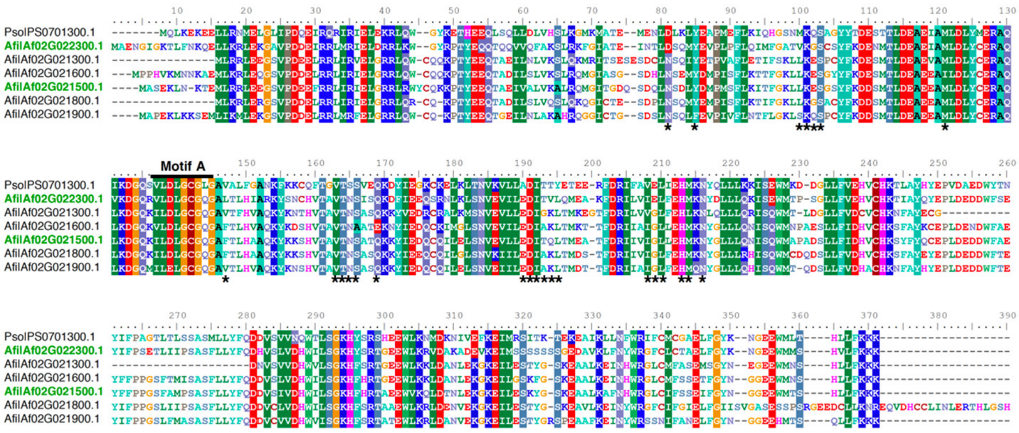


**Supplementary Fig. 6.5 | Alignment of the CNMT protein sequences in *P. somniferum* and *A. fimbriata*.** The black bar shows the key motif region, and asterisks represent the key functional residues previously identified in *P. somniferum*[180].

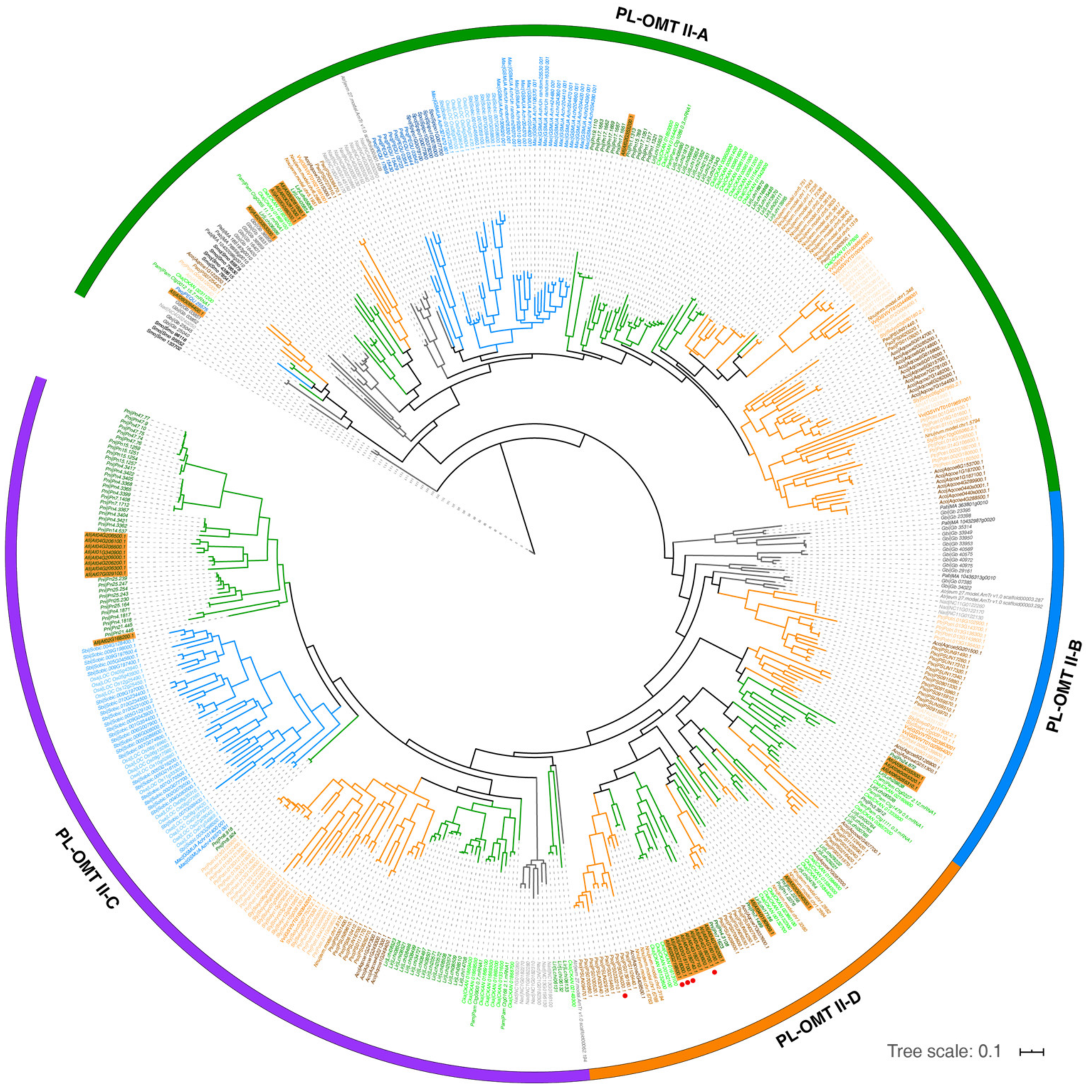


**Supplementary Fig. 6.6 | Phylogeny of the 6OMT gene family.** The phylogenetic tree was constructed using RAxML. Branches of the phylogeny were colored: orange-

eudicots; blue-monocots; green-magnoliids; gray-ANA grade, gymnosperms and lycophyte. The previously identified functional genes in *P. somniferum* and the closely related *A. fimbriata* genes in the PL-OMT II-D clade are marked with red dots. The *A. fimbriata* genes are highlighted with an orange background.

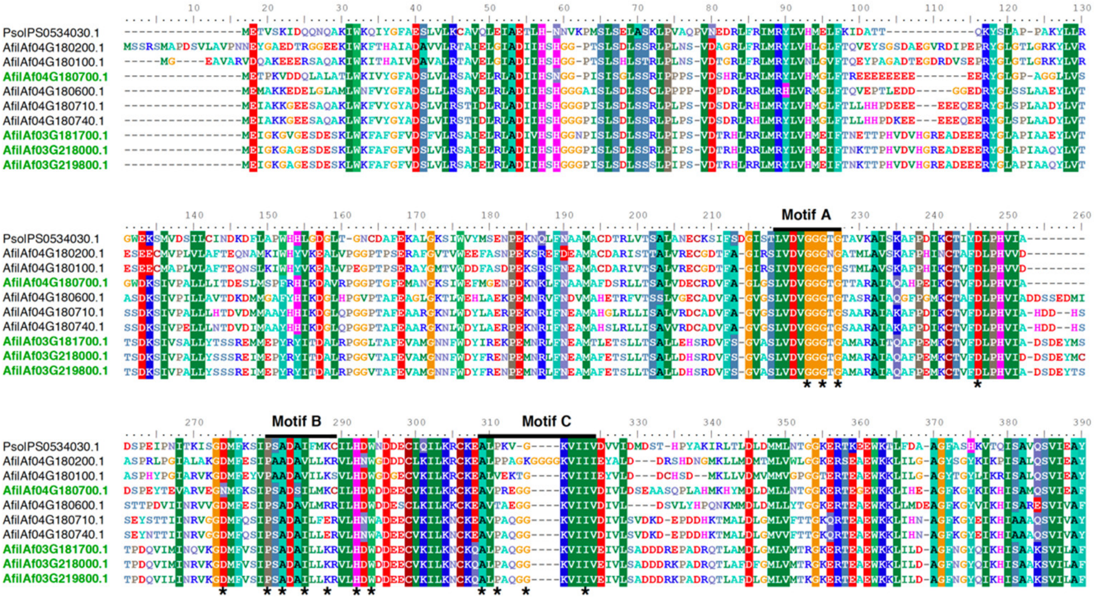


**Supplementary Fig. 6.7 | Alignment of the 6OMT amino acid sequences in *P. somniferum* and *A. fimbriata*.** The black bars show the regions of three key motifs, and asterisks represent the key enzyme activation residues that were identified in *P. somniferum* previously[181,182].

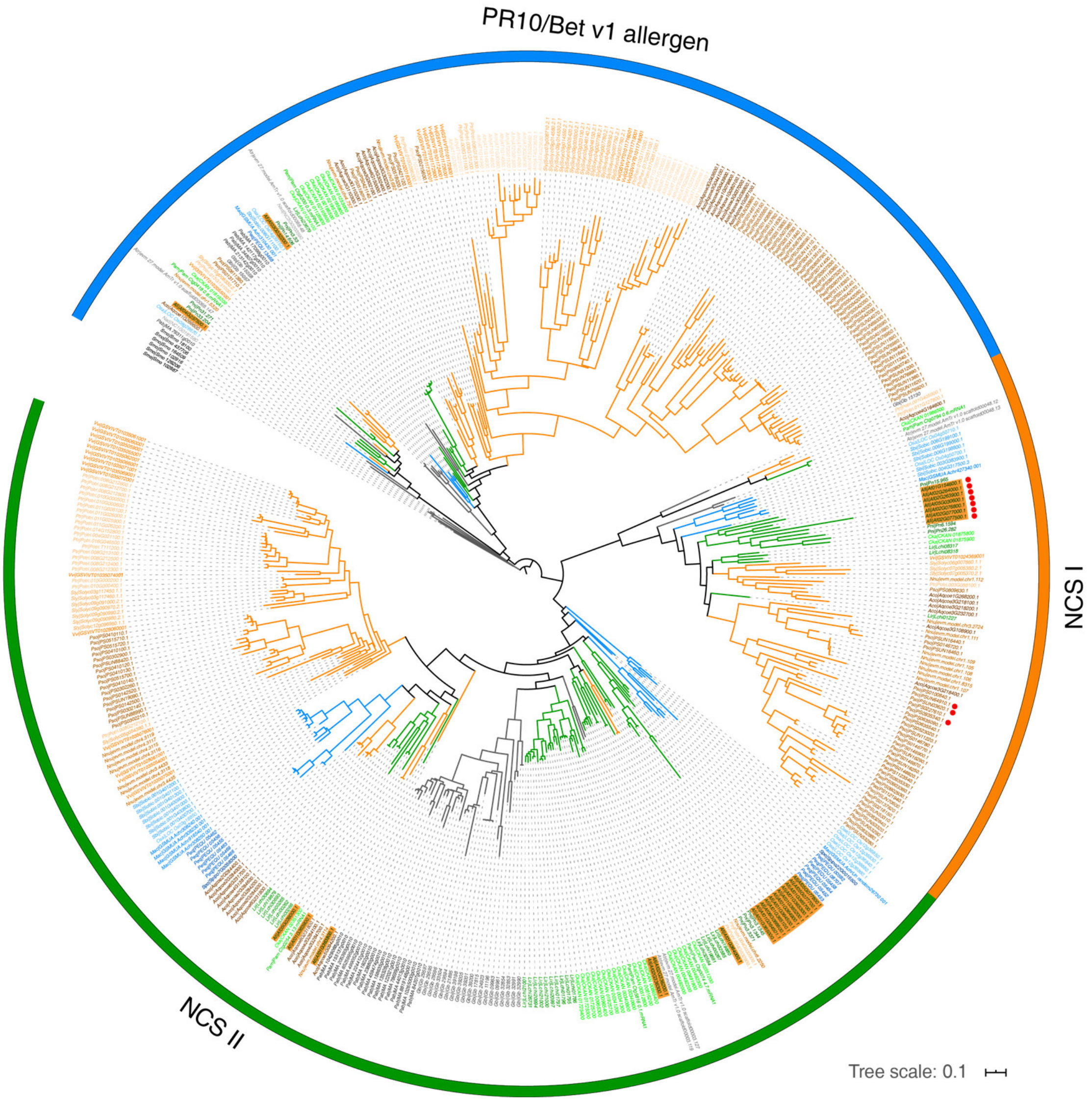


**Supplementary Fig. 6.8 | Phylogeny of the NCS gene family.** The phylogenetic tree was constructed using RAxML. Branches and genes were colored as follows: orange-eudicots; blue-monocots; green-magnoliids; gray-ANA grade, gymnosperms and lycophyte. The *A. fimbriata* genes are highlighted with an orange background. Seven *A. fimbriata* genes and three *P. somniferum* genes in the NCSI clade are marked with red dots. The *A. fimbriata* genes are highlighted with an orange background.

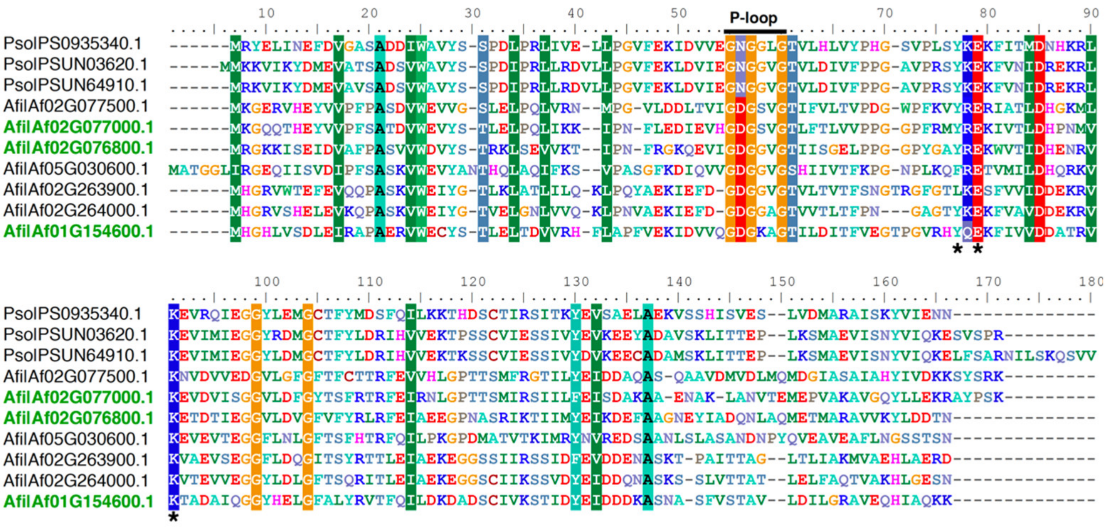


**Supplementary Fig. 6.9 | Alignment of NCSI amino acid sequences in *P. somniferum* and *A. fimbriata*.** The black bar depicts the conserved P-loop motif, and asterisks represent the catalytic residues.

**Supplementary References:**


1 Wagner, S. T. et al. Major trends in stem anatomy and growth forms in the perianth-bearing Piperales, with special focus on *Aristolochia*. *Ann. Bot.* **113**, 1139-1154 (2014).

2 González, F. & Stevenson, D. W. Perianth development and systematics of *Aristolochia*. *Flora* **195**, 370-391 (2000).

3 Gonzalez, F. & Pabon-Mora, N. Trickery flowers: the extraordinary chemical mimicry of *Aristolochia* to accomplish deception to its pollinators. *New Phytol.* **206**, 10-13 (2015).

4 Michl, J. et al. LC-MS- and (1)H NMR-based metabolomic analysis and in vitro toxicological assessment of 43 *Aristolochia* species. *J. Nat. Prod.* **79**, 30-37 (2016).

5 Bliss, B. J. et al. Characterization of the basal angiosperm *Aristolochia fimbriata*: a potential experimental system for genetic studies. *BMC Plant Biol.* **13**, 13 (2013).

6 Oelschlagel, B. et al. Spatio-temporal patterns in pollination of deceptive *Aristolochia rotunda* L. (Aristolochiaceae). *Plant Biol.* **18**, 928-937 (2016).

7 Martin, K. R. et al. Spatial and temporal variation in volatile composition suggests olfactory division of labor within the trap flowers of *Aristolochia gigantea*. *Flora* **232**, 153-168 (2017).

8 Oelschlagel, B., Gorb, S., Wanke, S. & Neinhuis, C. Structure and biomechanics of trapping flower trichomes and their role in the pollination biology of *Aristolochia* plants (Aristolochiaceae). *New Phytol.* **184**, 988-1002 (2009).

9 Heinrich, M., Chan, J., Wanke, S., Neinhuis, C. & Simmonds, M. S. Local uses of *Aristolochia* species and content of nephrotoxic aristolochic acid 1 and 2--a global assessment based on bibliographic sources. *J. Ethnopharmacol.* **125**, 108-144 (2009).

10 Nortier, J. L. et al. Urothelial carcinoma associated with the use of a Chinese herb (*Aristolochia fangchi*). *N. Engl. J. Med.* **342**, 1686-1692 (2000).

11 Ng, A. W. T. et al. Aristolochic acids and their derivatives are widely implicated in liver cancers in Taiwan and throughout Asia. *Sci. Transl. Med.* **9**, eaan6446 (2017).

12 Krell, D. & Stebbing, J. Aristolochia: the malignant truth. *Lancet Oncol.* **14**, 25-26 (2013).

13 Poon, S. L. et al. Genome-wide mutational signatures of aristolochic acid and its application as a screening tool. *Sci. Transl. Med.* **5**, 197ra101 (2013).

14 Marcais, G. & Kingsford, C. A fast, lock-free approach for efficient parallel counting of occurrences of k-mers. *Bioinformatics* **27**, 764-770 (2011).

15 Vurture, G. W. et al. GenomeScope: fast reference-free genome profiling from short reads. *Bioinformatics* **33**, 2202-2204 (2017).

16 Michael, T. P. et al. High contiguity *Arabidopsis thaliana* genome assembly with a single nanopore flow cell. *Nat. Commun.* **9**, 541 (2018).
17 Li, H. Minimap2: pairwise alignment for nucleotide sequences. *Bioinformatics* **34**, 3094-3100 (2018).
18 Li, H. Minimap and miniasm: fast mapping and de novo assembly for noisy long sequences. *Bioinformatics* **32**, 2103-2110 (2016).
19 Vaser, R., Sović, I., Nagarajan, N. & Šikić, M. Fast and accurate de novo genome assembly from long uncorrected reads. *Genome Res.* **27**, 737-746 (2017).
20 Li, H. Aligning sequence reads, clone sequences and assembly contigs with BWA-MEM. Preprint at https://arxiv.org/abs/1303.3997 (2013).
21 Walker, B. J. et al. Pilon: an integrated tool for comprehensive microbial variant detection and genome assembly improvement. *PLoS ONE* **9**, e112963 (2014).
22 Durand, N. C. et al. Juicer provides a one-click system for analyzing loop-eesolution Hi-C experiments. *Cell Syst.* **3**, 95-98 (2016).
23 Dudchenko, O. et al. The Juicebox Assembly Tools module facilitates de novo assembly of mammalian genomes with chromosome-length scaffolds for under $1000. Preprint at https://www.biorxiv.org/content/10.1101/254797v1 (2018).
24 Dudchenko, O. et al. De novo assembly of the *Aedes aegypti* genome using Hi-C yields chromosome-length scaffolds. *Science* **356**, 92-95 (2017).
25 Koren, S. et al. Canu: scalable and accurate long-read assembly via adaptive k-mer weighting and repeat separation. *Genome Res.* **27**, 722-736 (2017).
26 Bolger, A. M., Lohse, M. & Usadel, B. Trimmomatic: a flexible trimmer for Illumina sequence data. *Bioinformatics* **30**, 2114-2120 (2014).
27 Langmead, B. & Salzberg, S. L. Fast gapped-read alignment with Bowtie 2. *Nat. Methods* **9**, 357-359 (2012).
28 Li, H. et al. The sequence alignment/map format and SAMtools. *Bioinformatics* **25**, 2078-2079 (2009).
29 Bankevich, A. et al. SPAdes: a new genome assembly algorithm and its applications to single-cell sequencing. *J. Comput. Biol.* **19**, 455-477 (2012).
30 Kearse, M. et al. Geneious Basic: an integrated and extendable desktop software platform for the organization and analysis of sequence data. *Bioinformatics* **28**, 1647-1649 (2012).
31 Hahn, C., Bachmann, L. & Chevreux, B. Reconstructing mitochondrial genomes directly from genomic next-generation sequencing reads--a baiting and iterative mapping approach. *Nucleic Acids Res.* **41**, e129 (2013).
32 Lohse, M., Drechsel, O. & Bock, R. OrganellarGenomeDRAW (OGDRAW): a tool for the easy generation of high-quality custom graphical maps of plastid and mitochondrial genomes. *Curr. Genet.* **52**, 267-274 (2007).

33 Kim, D. et al. TopHat2: accurate alignment of transcriptomes in the presence of insertions, deletions and gene fusions. *Genome Biol.* **14**, R36 (2013).
34 Simao, F. A., Waterhouse, R. M., Ioannidis, P., Kriventseva, E. V. & Zdobnov, E. M. BUSCO: assessing genome assembly and annotation completeness with single-copy orthologs. *Bioinformatics* **31**, 3210-3212 (2015).
35 Ou, S., Chen, J. & Jiang, N. Assessing genome assembly quality using the LTR Assembly Index (LAI). *Nucleic Acids Res.* **46**, e126 (2018).
36 Bao, W., Kojima, K. K. & Kohany, O. Repbase Update, a database of repetitive elements in eukaryotic genomes. *Mob. DNA* **6**, 11 (2015).
37 Tarailo-Graovac, M. & Chen, N. Using RepeatMasker to identify repetitive elements in genomic sequences. *Curr. Protoc. Bioinformatics* **4**, 10 (2009).
38 Ellinghaus, D., Kurtz, S. & Willhoeft, U. LTRharvest, an efficient and flexible software for de novo detection of LTR retrotransposons. *BMC Bioinformatics* **9**, 18 (2008).
39 Xu, Z. & Wang, H. LTR_FINDER: an efficient tool for the prediction of full-length LTR retrotransposons. *Nucleic Acids Res.* **35**, W265-W268 (2007).
40 Ou, S. & Jiang, N. LTR_retriever: A highly accurate and sensitive program for identification of long terminal repeat retrotransposons. *Plant Physiol.* **176**, 1410-1422 (2018).
41 Salmela, L. & Rivals, E. LoRDEC: accurate and efficient long read error correction. *Bioinformatics* **30**, 3506-3514 (2014).
42 Li, W. & Godzik, A. Cd-hit: a fast program for clustering and comparing large sets of protein or nucleotide sequences. *Bioinformatics* **22**, 1658-1659 (2006).
43 Salamov, A. A. & Solovyev, V. V. Ab initio gene finding in *Drosophila* genomic DNA. *Genome Res.* **10**, 516-522 (2000).
44 Hoff, K. J. & Stanke, M. Predicting genes in single genomes with AUGUSTUS. *Curr. Protoc. Bioinformatics* **65**, e57 (2019).
45 Birney, E. & Durbin, R. Using GeneWise in the *Drosophila* annotation experiment. *Genome Res.* **10**, 547-548 (2000).
46 Keilwagen, J., Hartung, F. & Grau, J. GeMoMa: homology-based gene prediction utilizing intron position conservation and RNA-seq data. *Methods Mol. Biol.* **1962**, 161-177 (2019).
47 Xu, Y., Wang, X., Yang, J., Vaynberg, J. & Qin, J. PASA--a program for automated protein NMR backbone signal assignment by pattern-filtering approach. *J. Biomol. NMR.* **34**, 41-56 (2006).
48 Wu, T. D. & Watanabe, C. K. GMAP: a genomic mapping and alignment program for mRNA and EST sequences. *Bioinformatics* **21**, 1859-1875 (2005).
49 Haas, B. J. et al. Automated eukaryotic gene structure annotation using EVidenceModeler and the program to assemble spliced alignments. *Genome Biol.* **9**, R7 (2008).

50 Conesa, A. et al. Blast2GO: a universal tool for annotation, visualization and analysis in functional genomics research. *Bioinformatics* **21**, 3674-3676 (2005).
51 Huerta-Cepas, J. et al. Fast genome-wide functional annotation through orthology assignment by eggNOG-mapper. *Mol. Biol. Evol.* **34**, 2115-2122 (2017).
52 Camacho, C. et al. BLAST+: architecture and applications. *BMC Bioinformatics* **10**, 421 (2009).
53 Li, L., Stoeckert, C. J., Jr. & Roos, D. S. OrthoMCL: identification of ortholog groups for eukaryotic genomes. *Genome Res.* **13**, 2178-2189 (2003).
54 Enright, A. J., Van Dongen, S. & Ouzounis, C. A. An efficient algorithm for large-scale detection of protein families. *Nucleic Acids Res.* **30**, 1575-1584 (2002).
55 Amborella Genome Project. The *Amborella* genome and the evolution of flowering plants. *Science* **342**, 1241089 (2013).
56 Moore, M. J., Bell, C. D., Soltis, P. S. & Soltis, D. E. Using plastid genome-scale data to resolve enigmatic relationships among basal angiosperms. *Proc. Natl. Acad. Sci. USA* **104**, 19363-19368 (2007).
57 Soltis, D. E. et al. Angiosperm phylogeny: 17 genes, 640 taxa. *Am. J. Bot.* **98**, 704-730 (2011).
58 Jansen, R. K. et al. Analysis of 81 genes from 64 plastid genomes resolves relationships in angiosperms and identifies genome-scale evolutionary patterns. *Proc. Natl. Acad. Sci. USA* **104**, 19369-19374 (2007).
59 Zhang, L. et al. The water lily genome and the early evolution of flowering plants. *Nature* **577**, 79-84 (2020).
60 Wang, Y. et al. MCScanX: a toolkit for detection and evolutionary analysis of gene synteny and collinearity. *Nucleic Acids Res.* **40**, e49 (2012).
61 Wang, J. et al. An overlooked paleotetraploidization in Cucurbitaceae. *Mol. Biol. Evol.* **35**, 16-26 (2018).
62 Wang, J. et al. Hierarchically aligning 10 legume genomes establishes a family-level genomics platform. *Plant Physiol.* **174**, 284-300 (2017).
63 Nei, M. & Gojobori, T. Simple methods for estimating the numbers of synonymous and nonsynonymous nucleotide substitutions. *Mol. Biol. Evol.* **3**, 418-426 (1986).
64 Jiao, Y. et al. Ancestral polyploidy in seed plants and angiosperms. *Nature* **473**, 97-100 (2011).
65 Jiao, Y. & Paterson, A. H. Polyploidy-associated genome modifications during land plant evolution. *Philos. Trans. R. Soc. B Biol. Sci.* **369**, 20130355 (2014).
66 Hu, L. et al. The chromosome-scale reference genome of black pepper provides insight into piperine biosynthesis. *Nat. Commun.* **10**, 4702 (2019).
67 Samuel, R. Chromosome numbers in *Piper*. *Kew Bulletin* **42**, 465-470 (1987).

68 Rendon-Anaya, M. et al. The avocado genome informs deep angiosperm phylogeny, highlights introgressive hybridization, and reveals pathogen-influenced gene space adaptation. *Proc. Natl. Acad. Sci. USA* **116**, 17081-17089 (2019).

69 Chen, J. et al. *Liriodendron* genome sheds light on angiosperm phylogeny and species-pair differentiation. *Nat. Plants* **5**, 18-25 (2019).

70 Chaw, S. M. et al. Stout camphor tree genome fills gaps in understanding of flowering plant genome evolution. *Nat. Plants* **5**, 63-73 (2019).

71 Soltis, D. E. & Soltis, P. S. Isozyme evidence for ancient polyploidy in primitive angiosperms. *Syst. Bot.* **15**, 328-337 (1990).

72 Cui, L. et al. Widespread genome duplications throughout the history of flowering plants. *Genome Res.* **16**, 738-749 (2006).

73 Chen, Y. C. et al. The *Litsea* genome and the evolution of the laurel family. *Nat. Commun.* **11**, 1675 (2020).

74 Shang, J. et al. The chromosome-level wintersweet (*Chimonanthus praecox*) genome provides insights into floral scent biosynthesis and flowering in winter. *Genome Biol.* **21**, 200 (2020).

75 Wang, J. et al. Recursive paleohexaploidization shaped the durian genome. *Plant Physiol.* **179**, 209-219 (2019).

76 Jiao, Y. et al. A genome triplication associated with early diversification of the core eudicots. *Genome Biol.* **13**, R3 (2012).

77 Jiao, Y., Li, J., Tang, H. & Paterson, A. H. Integrated syntenic and phylogenomic analyses reveal an ancient genome duplication in monocots. *Plant Cell* **26**, 2792-2802 (2014).

78 Tang, H., Bowers, J. E., Wang, X. & Paterson, A. H. Angiosperm genome comparisons reveal early polyploidy in the monocot lineage. *Proc. Natl. Acad. Sci. USA* **107**, 472-477 (2010).

79 Singh, R. et al. Oil palm genome sequence reveals divergence of interfertile species in Old and New worlds. *Nature* **500**, 335-339 (2013).

80 Guo, L. et al. The opium poppy genome and morphinan production. *Science* **362**, 343-347 (2018).

81 Edgar, R. C. MUSCLE: multiple sequence alignment with high accuracy and high throughput. *Nucleic Acids Res.* **32**, 1792-1797 (2004).

82 Suyama, M., Torrents, D. & Bork, P. PAL2NAL: robust conversion of protein sequence alignments into the corresponding codon alignments. *Nucleic Acids Res.* **34**, W609-612 (2006).

83 Yang, Y. et al. Prickly waterlily and rigid hornwort genomes shed light on early angiosperm evolution. *Nat. Plants* **6**, 215-222 (2020).

84 Stamatakis, A. RAxML version 8: a tool for phylogenetic analysis and post-analysis of large phylogenies. *Bioinformatics* **30**, 1312-1313 (2014).

85 Mirarab, S. & Warnow, T. ASTRAL-II: coalescent-based species tree estimation with many hundreds of taxa and thousands of genes. *Bioinformatics* **31**, i44-i52 (2015).
86 Smith, S. A., Moore, M. J., Brown, J. W. & Yang, Y. Analysis of phylogenomic datasets reveals conflict, concordance, and gene duplications with examples from animals and plants. *BMC Evol. Biol.* **15**, 150 (2015).
87 Sayyari, E. & Mirarab, S. Fast coalescent-based computation of local branch support from quartet frequencies. *Mol. Biol. Evol.* **33**, 1654-1668 (2016).
88 Sayyari, E. & Mirarab, S. Testing for polytomies in phylogenetic species trees using quartet frequencies. *Genes* **9**, 132 (2018).
89 One Thousand Plant Transcriptomes Initiative. One thousand plant transcriptomes and the phylogenomics of green plants. *Nature* **574**, 679-685 (2019).
90 Yang, L. et al. Phylogenomic insights into deep phylogeny of angiosperms based on broad nuclear gene sampling. *Plant Comm.* **1** (2020).
91 Wickett, N. J. et al. Phylotranscriptomic analysis of the origin and early diversification of land plants. *Proc. Natl. Acad. Sci. USA* **111**, E4859-4868 (2014).
92 Stull, G. W., Soltis, P. S., Soltis, D. E., Gitzendanner, M. A. & Smith, S. A. Nuclear phylogenomic analyses of asterids conflict with plastome trees and support novel relationships among major lineages. *Am. J. Bot.* **107**, 790-805 (2020).
93 Li, H. T. et al. Origin of angiosperms and the puzzle of the Jurassic gap. *Nat. Plants* **5**, 461-470 (2019).
94 Gitzendanner, M. A., Soltis, P. S., Wong, G. K., Ruhfel, B. R. & Soltis, D. E. Plastid phylogenomic analysis of green plants: a billion years of evolutionary history. *Am. J. Bot.* **105**, 291-301 (2018).
95 Ruhfel, B. R., Gitzendanner, M. A., Soltis, P. S., Soltis, D. E. & Burleigh, J. G. From algae to angiosperms-inferring the phylogeny of green plants (Viridiplantae) from 360 plastid genomes. *BMC Evol. Biol.* **14**, 23 (2014).
96 Yang, Z. PAML 4: phylogenetic analysis by maximum likelihood. *Mol. Biol. Evol.* **24**, 1586-1591 (2007).
97 Kenrick, P. & Crane, P. R. The origin and early evolution of plants on land. *Nature* **389**, 33-39 (1997).
98 Miller, C. N. Implications of fossil conifers for the phylogenetic relationships of living families. *Bot. Rev.* **65**, 239-277 (1999).
99 Doyle, J. A. & Hotton, C. L. in *Pollen and Spores, Patterns of Diversification* (eds Blackmore, S. & Barnes, S. H.) 169-195 (Clarendon Press, Oxford, 1991).
100 Doyle, J. A. & Robbins, E. I. Angiosperm pollen zonation of the continental cretaceous of the Atlantic coastal plain and its application to deep wells in the Salisbury embayment. *Palynology* **1**, 43-78 (1977).

101 Hickey, L. J. & Doyle, J. A. Early cretaceous fossil evidence for angisperm evolution. *Bot. Rev.* **43**, 3-104 (1977).

102 Doyle, J. A. & Hickey, L. J. in *Origin and Early Evolution of Angiosperms* (eds Beck, C. B.) 139-206 (Columbia University Press, New York, 1976).

103 Mohr, B. A. R. & Bernardes-de-Oliveira, M. E. C. *Endressinia brasiliana*, a magnolialean angiosperm from the Lower Cretaceous Crato Formation. *Int. J. Plant Sci.* **165**, 1121-1133 (2004).

104 De Bie, T., Cristianini, N., Demuth, J. P. & Hahn, M. W. CAFE: a computational tool for the study of gene family evolution. *Bioinformatics* **22**, 1269-1271 (2006).

105 Gramzow, L. & Theissen, G. A hitchhiker's guide to the MADS world of plants. *Genome Biol.* **11**, 214 (2010).

106 Becker, A., Winter, K. U., Meyer, B., Saedler, H. & Theissen, G. MADS-box gene diversity in seed plants 300 million years ago. *Mol. Biol. Evol.* **17**, 1425-1434 (2000).

107 De Bodt, S., Raes, J., Van de Peer, Y. & Theissen, G. And then there were many: MADS goes genomic. *Trends Plant Sci.* **8**, 475-483 (2003).

108 Ng, M. & Yanofsky, M. F. Function and evolution of the plant MADS-box gene family. *Nat. Rev. Genet.* **2**, 186-195 (2001).

109 Smaczniak, C., Immink, R. G., Angenent, G. C. & Kaufmann, K. Developmental and evolutionary diversity of plant MADS-domain factors: insights from recent studies. *Development* **139**, 3081-3098 (2012).

110 Theissen, G. & Saedler, H. Floral quartets. *Nature* **409**, 469-471 (2001).

111 Parenicova, L. et al. Molecular and phylogenetic analyses of the complete MADS-box transcription factor family in *Arabidopsis*: new openings to the MADS world. *Plant Cell* **15**, 1538-1551 (2003).

112 Arora, R. et al. MADS-box gene family in rice: genome-wide identification, organization and expression profiling during reproductive development and stress. *BMC Genomics* **8**, 242 (2007).

113 Finn, R. D. et al. The Pfam protein families database: towards a more sustainable future. *Nucleic Acids Res.* **44**, D279-285 (2016).

114 Potter, S. C. et al. HMMER web server: 2018 update. *Nucleic Acids Res.* **46**, W200-W204 (2018).

115 Jones, P. et al. InterProScan 5: genome-scale protein function classification. *Bioinformatics* **30**, 1236-1240 (2014).

116 Katoh, K. & Standley, D. M. MAFFT multiple sequence alignment software version 7: improvements in performance and usability. *Mol. Biol. Evol.* **30**, 772-780 (2013).

117 Capella-Gutierrez, S., Silla-Martinez, J. M. & Gabaldon, T. trimAl: a tool for automated alignment trimming in large-scale phylogenetic analyses. *Bioinformatics* **25**, 1972-1973 (2009).

118 Chaudhary, S., Jabre, I., Reddy, A. S. N., Staiger, D. & Syed, N. H. Perspective on alternative splicing and proteome complexity in plants. *Trends Plant Sci.* **24**, 496-506 (2019).

119 Filichkin, S., Priest, H. D., Megraw, M. & Mockler, T. C. Alternative splicing in plants: directing traffic at the crossroads of adaptation and environmental stress. *Curr. Opin. Plant Biol.* **24**, 125-135 (2015).

120 Lee, J. H. et al. Regulation of temperature-responsive flowering by MADS-box transcription factor repressors. *Science* **342**, 628-632 (2013).

121 Lin, F., Zhang, Y. & Jiang, M. Y. Alternative splicing and differential expression of two transcripts of nicotine adenine dinucleotide phosphate oxidase B gene from *Zea mays*. *J. Integr. Plant Biol.* **51**, 287-298 (2009).

122 Pabón-Mora, N., Suárez-Baron, H., Ambrose, B. A. & González, F. Flower development and perianth identity candidate genes in the basal angiosperm *Aristolochia fimbriata* (Piperales: Aristolochiaceae). *Front. Plant Sci.* **6**, 1095 (2015).

123 Rogers, M. F., Thomas, J., Reddy, A. S. & Ben-Hur, A. SpliceGrapher: detecting patterns of alternative splicing from RNA-Seq data in the context of gene models and EST data. *Genome Biol.* **13**, R4 (2012).

124 Pertea, M., Kim, D., Pertea, G. M., Leek, J. T. & Salzberg, S. L. Transcript-level expression analysis of RNA-seq experiments with HISAT, StringTie and Ballgown. *Nat. Protoc.* **11**, 1650-1667 (2016).

125 Wellmer, F., Graciet, E. & Riechmann, J. L. Specification of floral organs in *Arabidopsis*. *J. Exp. Bot.* **65**, 1-9 (2014).

126 Theißen, G., Melzer, R. & Rümpler, F. MADS-domain transcription factors and the floral quartet model of flower development: linking plant development and evolution. *Development* **143**, 3259-3271 (2016).

127 Irish, V. The ABC model of floral development. *Curr. Biol.* **27**, R887-R890 (2017).

128 Specht, C. D. & Howarth, D. G. Adaptation in flower form: a comparative evodevo approach. *New Phytol.* **206**, 74-90 (2015).

129 Aida, M., Ishida, T., Fukaki, H., Fujisawa, H. & Tasaka, M. Genes involved in organ separation in *Arabidopsis*: an analysis of the cup-shaped cotyledon mutant. *Plant Cell* **9**, 841-857 (1997).

130 Baker, C. C., Sieber, P., Wellmer, F. & Meyerowitz, E. M. The early extra petals1 mutant uncovers a role for microRNA miR164c in regulating petal number in *Arabidopsis*. *Curr. Biol.* **15**, 303-315 (2005).

131 Endress, P. K. The immense diversity of floral monosymmetry and asymmetry across angiosperms. *Bot. Rev.* **78**, 345-397 (2012).

132 Horn, S., Pabon-Mora, N., Theuss, V. S., Busch, A. & Zachgo, S. Analysis of the *CYC/TB1* class of TCP transcription factors in basal angiosperms and magnoliids. *Plant J.* **81**, 559-571 (2015).

133 Dilcher, D. Toward a new synthesis: major evolutionary trends in the angiosperm fossil record. *Proc. Natl. Acad. Sci. USA* **97**, 7030-7036 (2000).

134 Martin-Trillo, M. & Cubas, P. TCP genes: a family snapshot ten years later. *Trends Plant Sci.* **15**, 31-39 (2010).

135 Pabón-Mora, N. et al. Evolution of Class II TCP genes in perianth bearing Piperales and their contribution to the bilateral calyx in *Aristolochia*. *New Phytol.* **228**, 752-769 (2020).

136 Luo, D., Carpenter, R., Vincent, C., Copsey, L. & Coen, E. Origin of floral asymmetry in *Antirrhinum*. *Nature* **383**, 794-799 (1996).

137 Luo, D. et al. Control of organ asymmetry in flowers of *Antirrhinum*. *Cell* **99**, 367-376 (1999).

138 Nath, U., Crawford, B. C., Carpenter, R. & Coen, E. Genetic control of surface curvature. *Science* **299**, 1404-1407 (2003).

139 Hileman, L. C. Bilateral flower symmetry--how, when and why? *Curr. Opin. Plant Biol.* **17**, 146-152 (2014).

140 Ishida, T., Kurata, T., Okada, K. & Wada, T. A genetic regulatory network in the development of trichomes and root hairs. *Annu. Rev. Plant Biol.* **59**, 365-386 (2008).

141 Yang, C. & Ye, Z. Trichomes as models for studying plant cell differentiation. *Cell. Mol. Life Sci.* **70**, 1937-1948 (2013).

142 Liu, X., Bartholomew, E., Cai, Y. & Ren, H. Trichome-related mutants provide a new perspective on multicellular trichome initiation and development in cucumber (*Cucumis sativus* L). *Front. Plant Sci.* **7**, 1187 (2016).

143 Yang, C. et al. A regulatory gene induces trichome formation and embryo lethality in tomato. *Proc. Natl. Acad. Sci. USA* **108**, 11836-11841 (2011).

144 Tanaka, Y. & Ohmiya, A. Seeing is believing: engineering anthocyanin and carotenoid biosynthetic pathways. *Curr. Opin. Biotechnol.* **19**, 190-197 (2008).

145 Albert, N. W. et al. A conserved network of transcriptional activators and repressors regulates anthocyanin pigmentation in eudicots. *Plant Cell* **26**, 962-980 (2014).

146 Dixon, R. A., Liu, C. & Jun, J. H. Metabolic engineering of anthocyanins and condensed tannins in plants. *Curr. Opin. Biotechnol.* **24**, 329-335 (2013).

147 Olsen, K. M. et al. Identification and characterisation of *CYP75A31*, a new flavonoid 3'5'-hydroxylase, isolated from *Solanum lycopersicum*. *BMC Plant Biol.* **10**, 21 (2010).

148 Holton, T. A. et al. Cloning and expression of cytochrome P450 genes controlling flower colour. *Nature* **366**, 276-279 (1993).

149 Da, L. et al. AppleMDO: a multi-dimensional omics database for apple co-expression networks and chromatin states. *Front. Plant Sci.* **10**, 1333 (2019).

150 Maes, L., Inzé, D. & Goossens, A. Functional specialization of the *TRANSPARENT TESTA GLABRA1* network allows differential hormonal control of laminal and marginal trichome initiation in *Arabidopsis* rosette leaves. *Plant Physiol.* **148**, 1453-1464 (2008).

151 Jaramillo, M. A. & Kramer, E. M. *APETALA3* and *PISTILLATA* homologs exhibit novel expression patterns in the unique perianth of *Aristolochia* (Aristolochiaceae). *Evol. Dev.* **6**, 449-458 (2004).

152 Mara, C. D. & Irish, V. F. Two GATA transcription factors are downstream effectors of floral homeotic gene action in *Arabidopsis*. *Plant Physiol.* **147**, 707-718 (2008).

153 Schutte, H. R., Orban, U. & Mothes, K. Biosynthesis of aristolochic acid. *Eur. J. Biochem.* **1**, 70-72 (1967).

154 Kautsar, S. A., Suarez Duran, H. G., Blin, K., Osbourn, A. & Medema, M. H. plantiSMASH: automated identification, annotation and expression analysis of plant biosynthetic gene clusters. *Nucleic Acids Res.* **45**, W55-W63 (2017).

155 Wang, X., Hui, F., Yang, Y. & Yang, S. Deep sequencing and transcriptome analysis to identify genes related to biosynthesis of aristolochic acid in *Asarum heterotropoides*. *Sci. Rep.* **8**, 17850 (2018).

156 Nutzmann, H. W. & Osbourn, A. Gene clustering in plant specialized metabolism. *Curr. Opin. Biotechnol.* **26**, 91-99 (2014).

157 Nutzmann, H. W., Huang, A. & Osbourn, A. Plant metabolic clusters - from genetics to genomics. *New Phytol.* **211**, 771-789 (2016).

158 Austin, M. B. & Noel, J. P. The chalcone synthase superfamily of type III polyketide synthases. *Nat. Prod. Rep.* **20**, 79-110 (2003).

159 Xie, L. et al. Phylogeny and expression analyses reveal important roles for plant PKS III Family during the conquest of land by plants and angiosperm diversification. *Front. Plant Sci.* **7**, 1312 (2016).

160 Shimizu, Y., Ogata, H. & Goto, S. Type III polyketide synthases: functional classification and phylogenomics. *ChemBioChem.* **18**, 50-65 (2017).

161 Pandith, S. A., Ramazan, S., Khan, M. I., Reshi, Z. A. & Shah, M. A. Chalcone synthases (CHSs): the symbolic type III polyketide synthases. *Planta* **251**, 15 (2019).

162 Ferrer, J. L., Jez, J. M., Bowman, M. E., Dixon, R. A. & Noel, J. P. Structure of chalcone synthase and the molecular basis of plant polyketide biosynthesis. *Nat. Struct. Biol.* **6**, 775-784 (1999).

163 Oelschlagel, B. et al. The betrayed thief - the extraordinary strategy of *Aristolochia rotunda* to deceive its pollinators. *New Phytol.* **206**, 342-351 (2015).

164 Chen, F., Tholl, D., Bohlmann, J. & Pichersky, E. The family of terpene synthases in plants: a mid-size family of genes for specialized metabolism that is highly diversified throughout the kingdom. *Plant J.* **66**, 212-229 (2011).

165 Tholl, D. Terpene synthases and the regulation, diversity and biological roles of terpene metabolism. *Curr. Opin. Plant Biol.* **9**, 297-304 (2006).
166 Johnson, S. D. & Jürgens, A. Convergent evolution of carrion and faecal scent mimicry in fly-pollinated angiosperm flowers and a stinkhorn fungus. *S. Afr. J. Bot.* **76**, 796-807 (2010).
167 Wang, X. et al. A new sesquiterpene, a new monoterpene and other constituents with anti-inflammatory activities from the roots of *Aristolochia debilis*. *Nat. Prod. Res.* **34**, 351-358 (2020).
168 Jiang, S. Y., Jin, J., Sarojam, R. & Ramachandran, S. A comprehensive survey on the terpene synthase gene family provides new insight into its evolutionary patterns. *Genome Biol. Evol.* **11**, 2078-2098 (2019).
169 Tolke, E. D. et al. Osmophores and floral fragrance in *Anacardium humile* and *Mangifera indica* (Anacardiaceae): an overlooked secretory structure in Sapindales. *AoB Plants* **10**, ply062 (2018).
170 Dudareva, N. et al. (E)-beta-ocimene and myrcene synthase genes of floral scent biosynthesis in snapdragon: function and expression of three terpene synthase genes of a new terpene synthase subfamily. *Plant Cell* **15**, 1227-1241 (2003).
171 Comer, F., Tiwari, H. P. & Spenser, I. D. Biosynthesis of aristolochic acid. *Can. J. Chem.* **47**, 481-487 (1969).
172 Sharma, V., Jain, S., Bhakuni, D. & Kapil, R. Biosynthesisof aristolochic acid. *J. Chem. Soc. Perkin Trans.* **1**, 1153-1155 (1982).
173 Hagel, J. M. & Facchini, P. J. Benzylisoquinoline alkaloid metabolism: a century of discovery and a brave new world. *Plant Cell Physiol.* **54**, 647-672 (2013).
174 Ziegler, J. & Facchini, P. J. Alkaloid biosynthesis: metabolism and trafficking. *Annu. Rev. Plant Biol.* **59**, 735-769 (2008).
175 Takemura, T., Ikezawa, N., Iwasa, K. & Sato, F. Molecular cloning and characterization of a cytochrome P450 in sanguinarine biosynthesis from *Eschscholzia californica* cells. *Phytochemistry* **91**, 100-108 (2013).
176 Ikezawa, N., Iwasa, K. & Sato, F. Molecular cloning and characterization of CYP80G2, a cytochrome P450 that catalyzes an intramolecular C-C phenol coupling of (S)-reticuline in magnoflorine biosynthesis, from cultured *Coptis japonica* cells. *J. Biol. Chem.* **283**, 8810-8821 (2008).
177 Ikezawa, N. et al. Molecular cloning and characterization of CYP719, a methylenedioxy bridge-forming enzyme that belongs to a novel P450 family, from cultured *Coptis japonica* cells. *J. Biol. Chem.* **278**, 38557-38565 (2003).
178 Ikezawa, N., Iwasa, K. & Sato, F. Molecular cloning and characterization of methylenedioxy bridge-forming enzymes involved in stylopine biosynthesis in *Eschscholzia californica*. *FEBS J.* **274**, 1019-1035 (2007).
179 Mizutani, M. & Ohta, D. Diversification of P450 genes during land plant evolution. *Annu. Rev. Plant Biol.* **61**, 291-315 (2010).

180 Ounaroon, A., Decker, G., Schmidt, J., Lottspeich, F. & Kutchan, T. M. (R,S)-Reticuline 7-O-methyltransferase and (R,S)-norcoclaurine 6-O-methyltransferase of *Papaver somniferum* - cDNA cloning and characterization of methyl transfer enzymes of alkaloid biosynthesis in opium poppy. *Plant J.* **36**, 808-819 (2003).

181 Meelaph, T. et al. Coregulation of biosynthetic genes and transcription factors for aporphine-type alkaloid production in wounded lotus provides insight into the biosynthetic pathway of nuciferine. *ACS omega* **3**, 8794-8802 (2018).

182 Robin, A. Y., Giustini, C., Graindorge, M., Matringe, M. & Dumas, R. Crystal structure of norcoclaurine-6-O-methyltransferase, a key rate-limiting step in the synthesis of benzylisoquinoline alkaloids. *Plant J.* **87**, 641-653 (2016).

183 Li, J., Lee, E. J., Chang, L. & Facchini, P. J. Genes encoding norcoclaurine synthase occur as tandem fusions in the Papaveraceae. *Sci. Rep.* **6**, 39256 (2016).

184 Livak, K. J. & Schmittgen, T. D. Analysis of relative gene expression data using real-time quantitative PCR and the 2(-Delta Delta C(T)) Method. *Methods* **25**, 402-408 (2001).

185 Yang, L., Zhu, J., Sun, C., Deng, Z. & Qu, X. Biosynthesis of plant tetrahydroisoquinoline alkaloids through an imine reductase route. *Chem. Sci.* **11**, 364-371 (2020).

186 Choi, K. B., Morishige, T. & Sato, F. Purification and characterization of coclaurine N-methyltransferase from cultured *Coptis japonica* cells. *Phytochemistry* **56**, 649-655 (2001).

187 Malakar, D., Chaudhuri, P., Dutta, T. & Ghosh, A. K. S-adenosyl-L-methionine, trehalose and oleanolic acid in few plants. *Health* **2**, 968-972 (2010).

188 Ali, R. et al. In silico identification and structure function analysis of a putative coclaurine N-methyltransferase from *Aristolochia fimbriata*. *Comput. Biol. Chem.* **85**, 107201 (2020).

189 Morris, J. S. & Facchini, P. J. Molecular origins of functional diversity in benzylisoquinoline alkaloid methyltransferases. *Front. Plant Sci.* **10**, 1058 (2019).

190 Salim, V., Jones, A. D. & DellaPenna, D. *Camptotheca acuminata* 10-hydroxycamptothecin O-methyltransferase: an alkaloid biosynthetic enzyme co-opted from flavonoid metabolism. *Plant J.* **95**, 112-125 (2018).

191 Lam, K. C., Ibrahim, R. K., Behdad, B. & Dayanandan, S. Structure, function, and evolution of plant O-methyltransferases. *Genome* **50**, 1001-1013 (2007).

192 Lee, E. J. & Facchini, P. Norcoclaurine synthase is a member of the pathogenesis-related 10/Bet v1 protein family. *Plant Cell* **22**, 3489-3503 (2010).

193 Liscombe, D. K., Macleod, B. P., Loukanina, N., Nandi, O. I. & Facchini, P. J. Evidence for the monophyletic evolution of benzylisoquinoline alkaloid biosynthesis in angiosperms. *Phytochemistry* **66**, 1374-1393 (2005).

194 Samanani, N. & Facchini, P. J. Isolation and partial characterization of norcoclaurine synthase, the first committed step in benzylisoquinoline alkaloid biosynthesis, from opium poppy. *Planta* **213**, 898-906 (2001).

195 Samanani, N. & Facchini, P. J. Purification and characterization of norcoclaurine synthase: the first committed enzyme in benzylisoquinoline alkaloid biosynthesis in plants. *J. Biol. Chem.* **277**, 33878-33883 (2002).

196 Vimolmangkang, S. et al. Evolutionary origin of the *NCSI* gene subfamily encoding norcoclaurine synthase is associated with the biosynthesis of benzylisoquinoline alkaloids in plants. *Sci. Rep.* **6**, 26323 (2016).
